# Infection Characteristics and Transcriptomics of African Swine Fever Virus in Bama Minipigs

**DOI:** 10.1128/spectrum.03834-22

**Published:** 2022-11-29

**Authors:** Changjie Lv, Jingyu Yang, Li Zhao, Chao Wu, Chao Kang, Qiang Zhang, Xiaomei Sun, Xi Chen, Zhong Zou, Meilin Jin

**Affiliations:** a College of Veterinary Medicine, Huazhong Agricultural University, Wuhan, China; b The Cooperative Innovation Center for Sustainable Pig Production, Wuhan, China; c State Key Laboratory of Biocatalysis and Enzyme Engineering, College of Life Sciences, Hubei University, Wuhan, China; d Research Institute of Wuhan Keqian Biology Co., Ltd., Wuhan, China; e College of Biomedicine and Health, Huazhong Agricultural University, Wuhan, China; University of Sussex

**Keywords:** African swine fever virus, virus isolation, Bama minipig, host-virus infection

## Abstract

Animal experiments on African swine fever virus (ASFV) are vital to the study of ASFV; however, ASFV can only infect pigs, and animal experiments need to be performed in animal biosafety level 3 (ABSL-3) laboratories, meaning that many small ABSL-3 laboratories are unable to carry out *in vivo* ASFV experiments. Therefore, miniaturized experimental animals for ASFV infection are urgently needed. Here, we successfully isolated genotype II of ASFV SY-1 from wild boars and evaluated ASFV-infected Bama minipigs in a negative-pressure isolator of a small ABSL-3 laboratory. The pathological changes of ASFV-infected Bama minipigs were consistent with characteristic lesions of ASFV-infected domestic pigs and wild boars. All pigs died 5 to 14 days postinfection (dpi) through intramuscular injection. Viral genomic DNA from nasal, oral, and rectal swab samples was first detectable at 2 to 4 dpi. The common differentially expressed genes were clustered in the immune-related, metabolic, and inflammatory response pathways from the spleen and inguinal lymph node samples comparing infected to mock. In summary, these results demonstrated that the Bama minipig was an appropriate model for ASFV infection in small ABSL-3 laboratories that can accelerate the research of vaccines and antiviral drugs and uncover pathogenic mechanisms of ASFV infection.

**IMPORTANCE** African swine fever virus (ASFV) can only infect pigs rather than other animals. However, the domestic pigs cannot be kept in small ABSL-3 laboratories for a long time due to the characteristics of rapid growth and large size, which hinder ASFV research, including research of vaccines, antiviral drugs, and mechanisms. In contrast, Bama minipigs have unique advantages consisting of low growth and small size. In the research, Bama minipigs were used to evaluate the characteristics of ASFV infection in small ABSL-3 laboratories. The pathological changes, viral shedding, and gene regulation were consistent with those of domestic pigs infected with ASFV. Therefore, Bama minipigs can be a suitable model for ASFV infection in small ABSL-3 laboratories.

## INTRODUCTION

African swine fever (ASF), a highly lethal hemorrhagic disease of domestic pigs and wild boars, causes enormous economic losses worldwide (1). African swine fever virus (ASFV), the causative agent of ASF, is the only member of the family *Asfarviridae* and the only known DNA arbovirus to date (2). The virus contains a linear double-stranded DNA genome of 170 to 190 kb in length, depending on the strain, which encodes more than 150 open reading frames (ORFs). These ORFs play vital roles in viral assembly, replication, and evasion of host defenses, including cell death and type I interferon pathways (3, 4). Although the functions of ASFV proteins have been studied, there are no effective and safe vaccines or antiviral drugs available against ASFV infection so far.

ASFV was first discovered and isolated in Kenya in 1921, followed by its discovery in other countries outside Africa (5). In 2007, the outbreak of genotype II ASFV was reported in Georgia and Armenia (6). ASF was first discovered in China in 2018 and subsequently spread across China, followed by outbreaks in Mongolia and Vietnam in 2019 (7, 8). Various natural mutational ASFV strains have also emerged that have fast viral spread, which has produced a greater challenge for the prevention and control of ASFV in domestic pigs and wild boars (9). Meanwhile, wild boars are free-moving animals that are more susceptible to ASFV infection and could pose a constant risk of transmission to domestic pigs. Thus, it is important to monitor and isolate ASFV from wild boars, which can lead to development of more effective strategies for viral control.

Animal experiments of ASFV infection need to be implemented in laboratories of at least animal biosafety level 3 (ABSL-3). However, domestic pigs are too large and grow rapidly, meaning that many small ABSL-3 laboratories are unsuitable for animal experiments on ASFV infection. The Bama minipig, a unique breed of miniature pig, is an excellent indigenous breed for this purpose from Bama County, China. Since Bama minipigs are more docile than wild boars but grow more slowly than domestic pigs, Bama minipigs are widely used as experimental animals for drug evaluation and gene editing. A previous study reported using CRISPR/Cas9 technology-generated growth hormone receptor (*GHR*) knockout Bama minipigs and *GHR* knockout Bama fibroblast cells (10). Bama minipigs have been used as an experimental model for Staphylococcus aureus hepatic abscesses (11). Bama minipigs have also been used to estimate low-dose sustained-release deoxycorticosterone acetate-induced hypertension (12). Therefore, Bama minipigs can also be raised in negative-pressure isolators of ABSL-3 laboratories as a model for severe infectious diseases owing to specific advantages.

Here, we isolated a strain of ASFV from wild boars and analyzed its viral genotype and gene sequences. Furthermore, Bama minipigs were used as animal models to evaluate ASFV infection at different doses. The survival rate, viremia, disease signs, lesions, and viral loads in various tissues and organs were recorded. The transcriptomes of spleens and inguinal lymph nodes after ASFV infection were studied to identify the relevant pathways that were enriched in the infected Bama minipigs.

## RESULTS

### Isolation of ASFV and characteristic sequence analyses.

**FIG 1:**
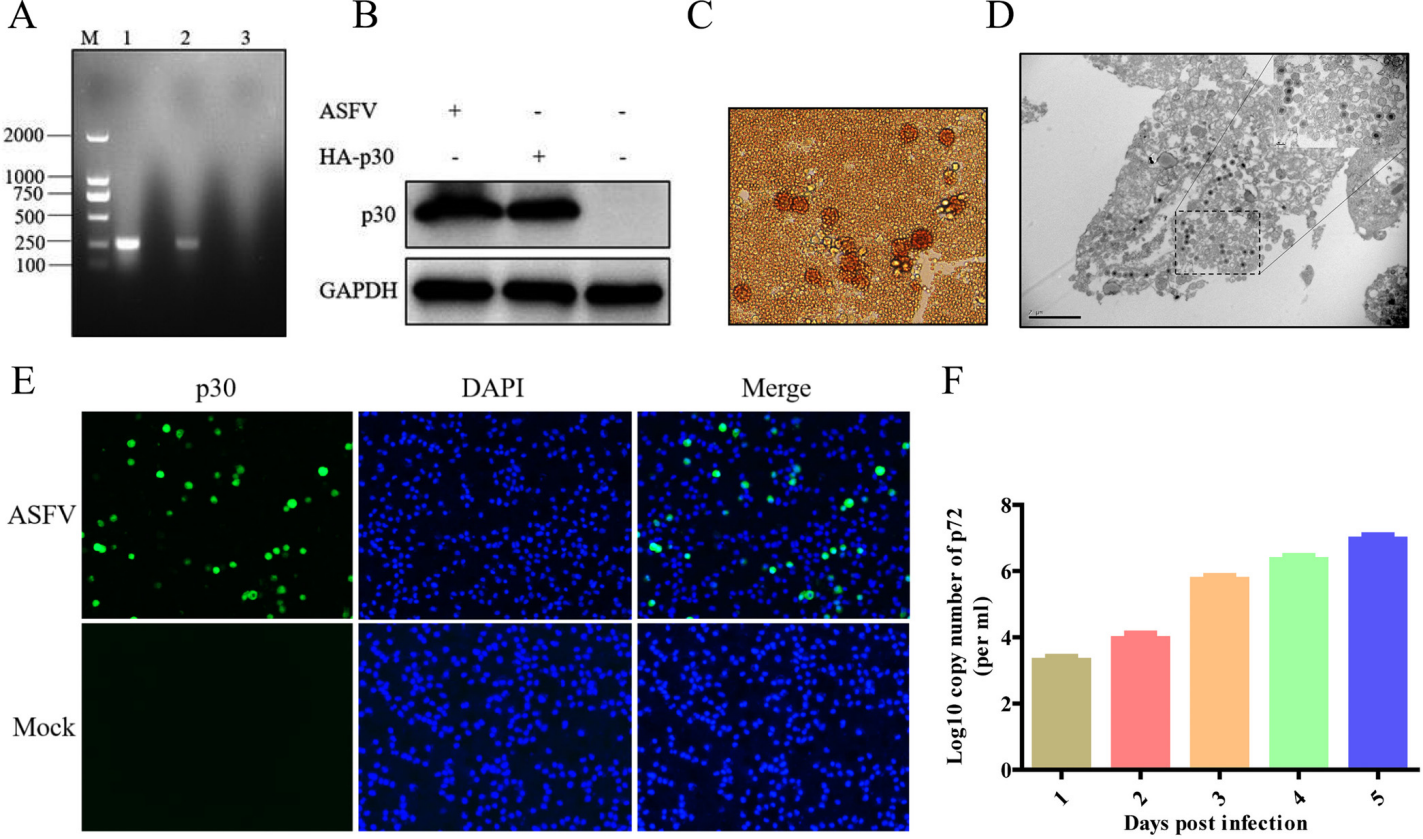
Isolation and identification of African swine fever virus (ASFV) SY-1 and phylogenetic analysis. (A) The homogenate of a spleen sample was tested using the OIE-recommended PCR method. M, marker; lane 1, sample; lane 2, positive control; lane 3, negative control. (B) ASFV p30 proteins in the infected primary porcine alveolar macrophages (PAMs) were detected by Western blotting. 293T cells were transfected with pCAGGS-HA-p30 as a positive control. Lane 1, sample; lane 2, positive control; lane 3, negative control. (C) ASFV infectious virus particles were detected using a hemadsorption assay in peripheral blood mononuclear cells. (D) ASFV particles were observed by electron microscopy in primary PAMs. (E) ASFV p30 proteins in the infected primary PAMs were detected by immunofluorescence assay. DAPI, 4′,6-diamidino-2-phenylindole. (F) The growth curve of ASFV was determined by qPCR targeting the viral B646L gene.

The OIE-recommended PCR method (13) was used to detect ASFV in the spleen samples from wild boars. As shown in Fig. 1A, a 257-bp product was consistent with the positive-control sample, illustrating that spleen samples contained ASFV. The supernatants of spleen homogenates were inoculated in primary porcine alveolar macrophages (PAMs) for 5 days, and cell supernatants were collected as the first generation of the virus; furthermore, the expression of ASFV p30 proteins was detected in cell lysates by Western blotting. The molecular weights of specific bands were consistent with the positive-control sample transfection with pCAGGS-HA-p30 plasmids in 293T cells (Fig. 1B). The first-passage virus was inoculated again in PAMs, and the characteristic hemadsorption (HAD) of ASFV was observed (Fig. 1C). Similarly, the expression of ASFV p30 proteins was also detected by an indirect immunofluorescence assay in PAMs (Fig. 1E). Additionally, the characteristic morphology of ASFV particles was observed by electron microscopy in the cytoplasm of primary PAMs (Fig. 1D). Therefore, these results demonstrated that ASFV was successfully isolated from the spleen samples of wild boars. To estimate the viral growth dynamic characteristics, primary PAMs were inoculated with ASFV at a multiplicity of infection (MOI) of 0.1, and the cell supernatants were collected at indicated time points after infection to determine the number of viral genome copies by quantitative PCR (qPCR). The viral genome copy numbers reached 8.7 × 10^6^/mL at 5 days postinfection (dpi) (Fig. 1F).

**FIG 2:**
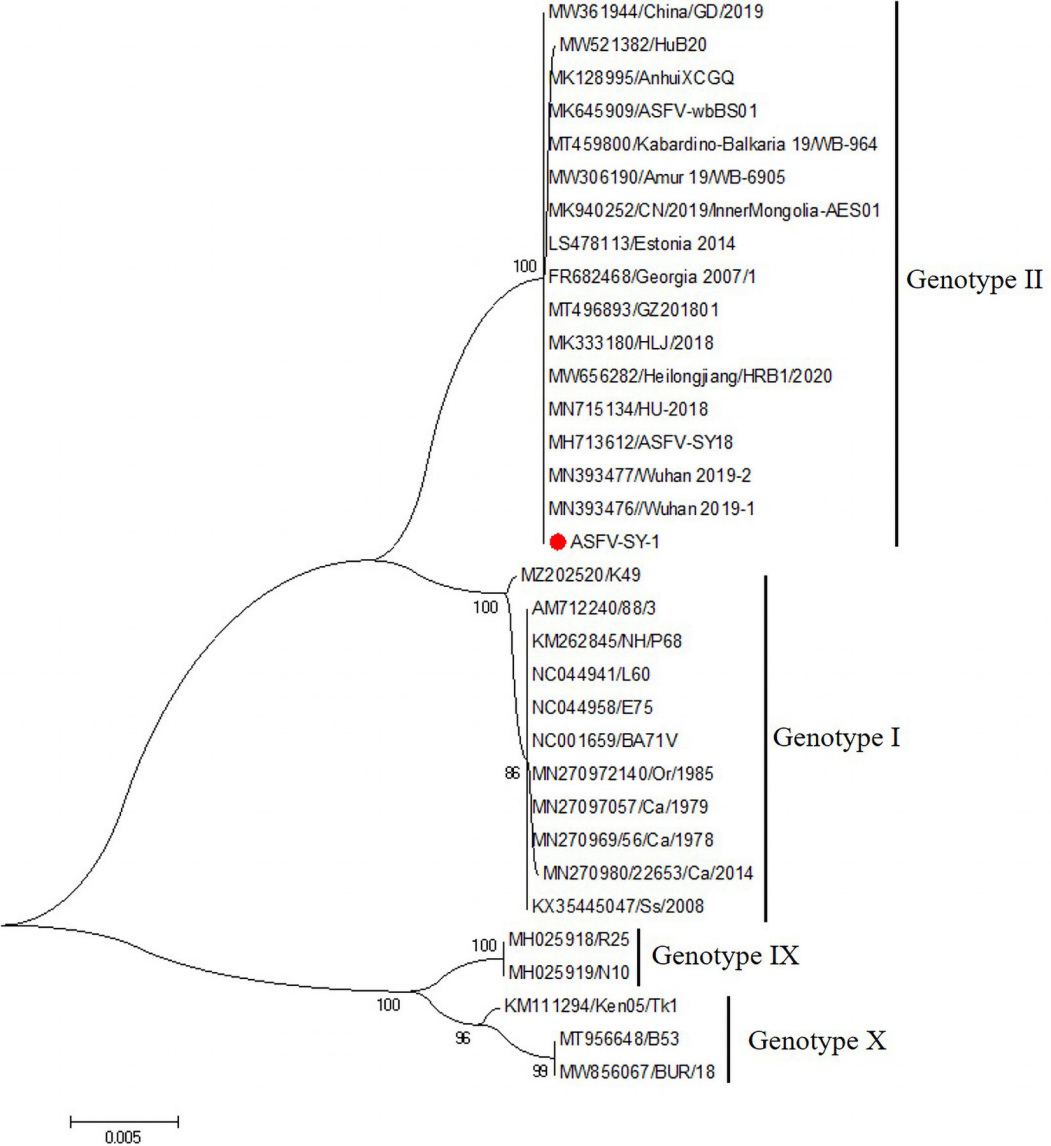
The genotype of ASFV SY-1 was analyzed by constructing phylogenetic trees using MEGA X software. The red circle indicates the ASFV isolate in this study.

To determine the genotype of ASFV SY-1, we analyzed the gene sequence of *B646L*, which encodes the p72 protein. ASFV SY-1 belonged to genotype II (Fig. 2). The whole-genome sequencing analysis found that the ASFV SY-1 genome was similar to the Pig/HLJ/18 strain, and there were only seven base site mutations in the left repeat region, MGF110-13L, MGF505-9R, F778R, C717R, and G1340L.

### Bama minipigs infected with various doses of ASFV SY-1.

**FIG 3:**
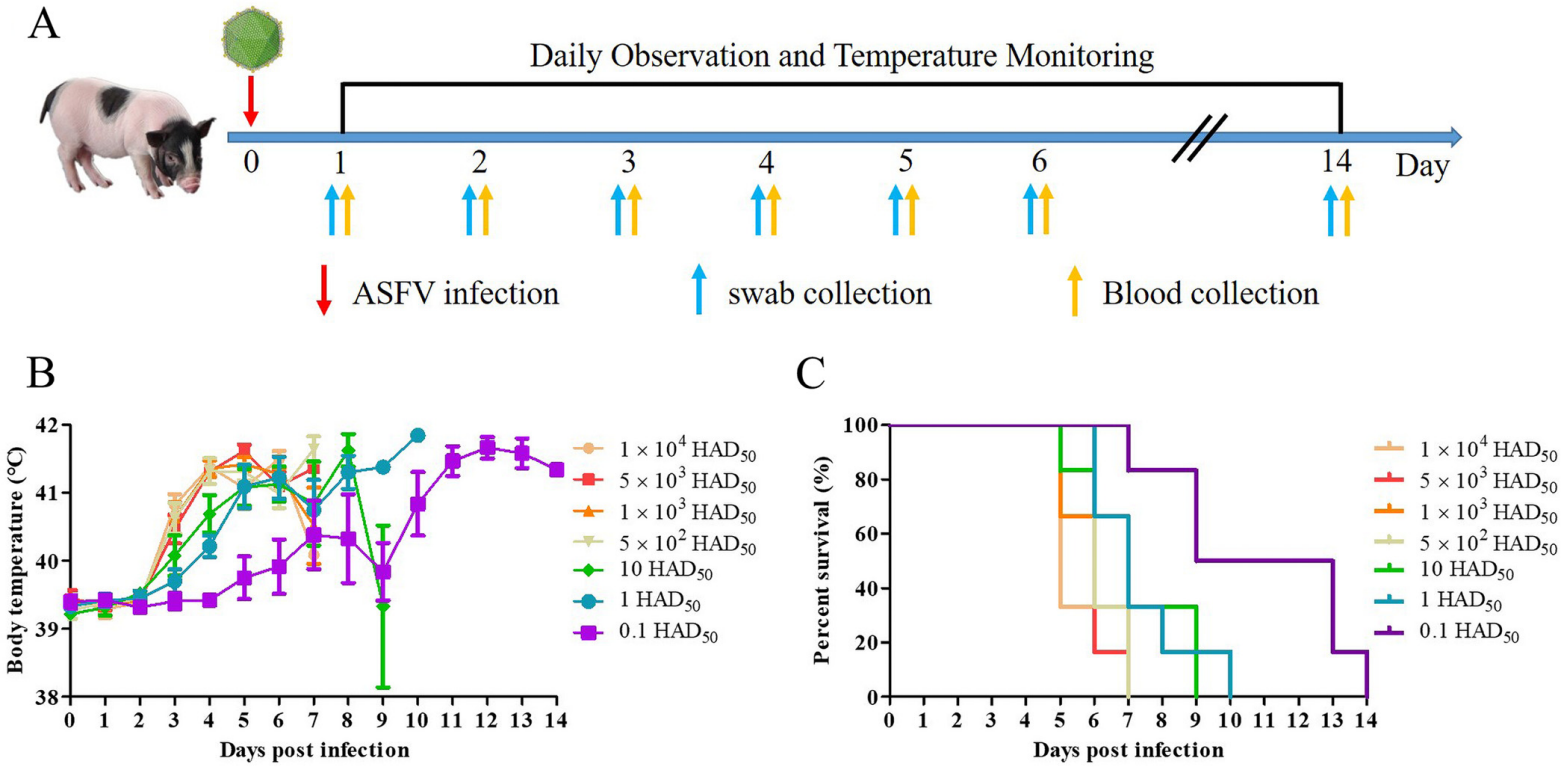
Schematic overview of the established infection model. Bama minipigs were infected with African swine fever virus (ASFV), leading to changes in body temperature and survival rate. (A) Bama minipigs were infected with ASFV SY-1 doses ranging from 0.1 HAD_50_ to 10^4^ HAD_50_. Swabs and blood were collected, and body temperature was monitored every day after infection. (B) Body temperature changes of Bama minipigs infected with ASFV at different doses from 0.1 HAD_50_ to 10^4^ HAD_50_. (C) Differences in survival rate of Bama minipigs infected ASFV at various doses ranging from 0.1 HAD_50_ to 10^4^ HAD_50_.

The ASFV Pig/HLJ/18 strain is an extremely virulent epidemic strain previously reported in China (7), and the genome of SY-1 is similar to Pig/HLJ/18. Therefore, to establish a model of Bama minipigs for ASFV infection, SY-1 was chosen as the challenge strain. The whole experimental procedure was performed as shown in Fig. 3A. Swabs and blood were collected, and body temperature was monitored every day after infection. Seven experimental groups, with six pigs in each group, were inoculated with 10^4^ 50% HAD doses (HAD_50_), 5 × 10^3^ HAD_50_, 10^3^ HAD_50_, 5 × 10^2^ HAD_50_, 10 HAD_50_, 1 HAD_50_, and 0.1 HAD_50_. The body temperatures of pigs from six experimental groups besides the 0.1 HAD_50_ group exceeded 40°C at 3 dpi until death, while that of 0.1 HAD_50_ exceeded 40°C at 5 dpi (Fig. 3B). The pigs of the challenge groups infected with 5 × 10^2^ to 5 × 10^4^ HAD_50_ died 5 to 7 dpi, and those infected with 0.1 to 10 HAD_50_ died 5 to 14 dpi (Fig. 3C and Table S2). These results showed that a 50% lethality dose (LD_50_) in Bama minipigs infected with virulent ASFV was lower than 0.1 HAD_50_. The genome of the viral progeny from infected Bama minipigs was highly consistent with the original viral genome.

### Shedding and replication of ASFV SY-1 in Bama minipigs.

**FIG 4:**
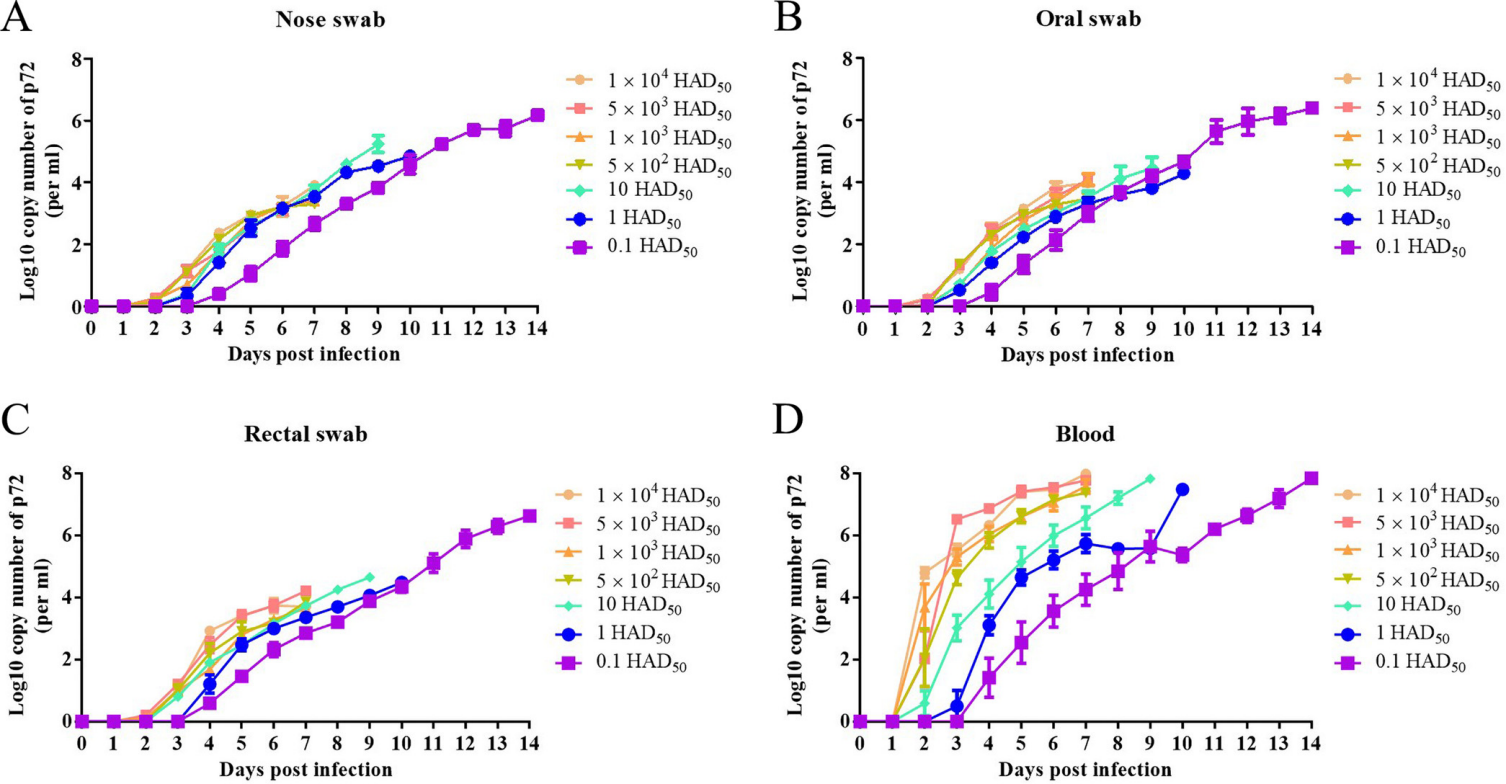
Viral genomic copies were detected from the swabs and blood of Bama minipigs infected with African swine fever virus (ASFV) SY-1 at different doses. Viral genomic copies were detected from nose swabs (A), oral swabs (B), and rectal swabs (C) of Bama minipigs infected with ASFV SY-1 at doses of 0.1 HAD_50_ to 10^4^ HAD_50_ every day after infection. (D) Viral genomic copies in the blood of Bama minipigs infected with ASFV SY-1 were detected in different groups every day after infection.

To assess the shedding and replication of the virus with Bama minipigs infected with the ASFV SY-1 strain, nose, oral, rectal swabs, and blood samples were collected for further detection by qPCR. The viral genome copy numbers of nose swabs, oral swabs, and rectal swabs were detected earliest at 2 to 4 dpi in all groups (Fig. 4A to C), while the viral genome copies from blood were detected as early as 2 to 4 dpi in seven experimental groups (Fig. 4D). Meanwhile, a viral genome copy of organs was detected after pig death found that the quantity of the virus was the highest in spleen in all groups up to 1.4 × 10^9^ copies/g (see Table S2 in the supplemental material). The shedding of the virus from pigs infected with a dose of 0.1 HAD_50_ was 1 to 2 days later than the other six groups in all swab samples. Moreover, viral replication of blood from pigs infected with doses of 10^4^ HAD_50_, 5 × 10^3^ HAD_50_, 10^3^ HAD_50_, 5 × 10^2^ HAD_50_, 10 HAD_50_, and 1 HAD_50_ was 2 days earlier than those infected with 0.1 HAD_50_.

### Cytokines of Bama minipigs infected with ASFV SY-1.

**FIG 5:**
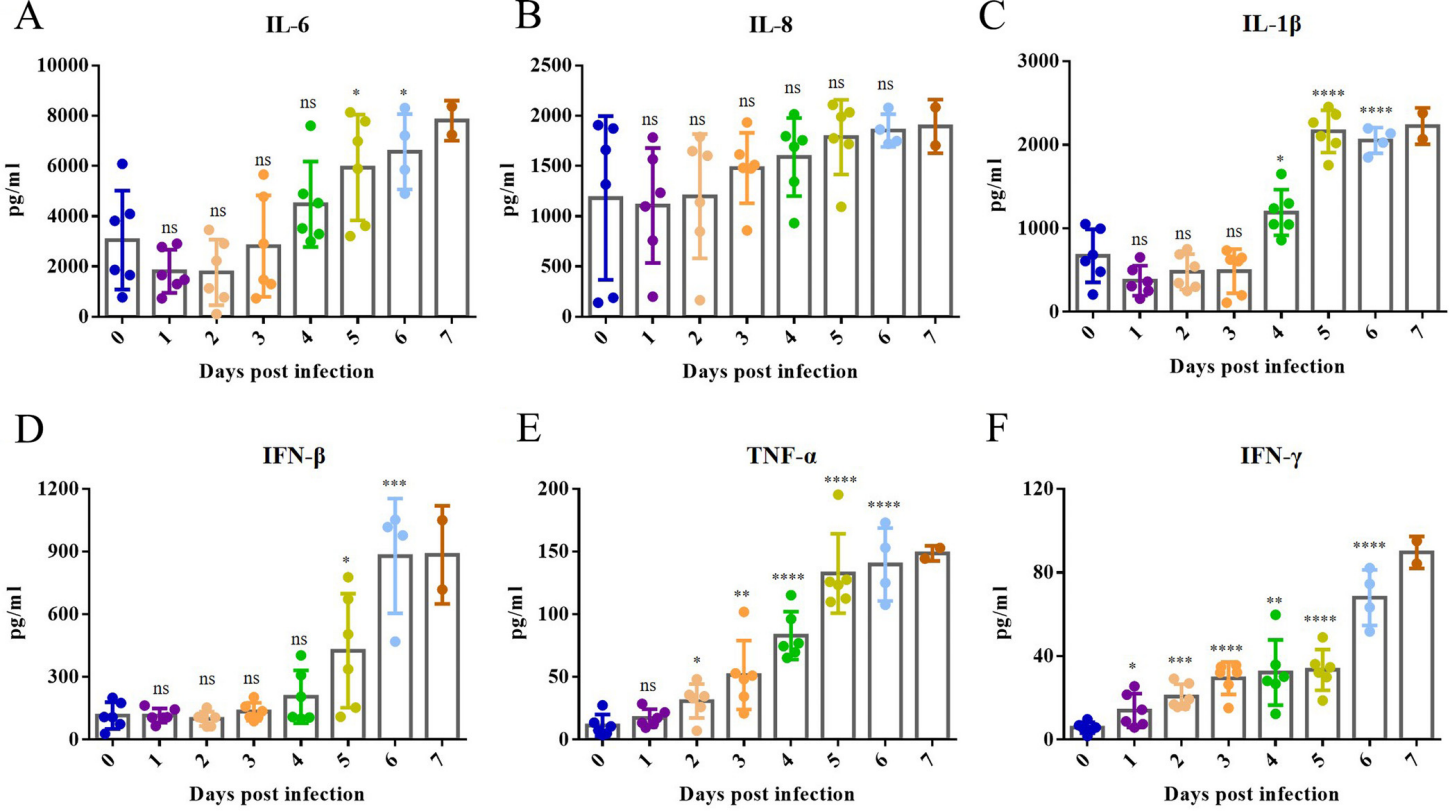
Cytokines were detected from the serum samples of Bama minipigs infected with African swine fever virus (ASFV) SY-1. Cytokines, including IL-6 (A), IL-8 (B), IL-1β (C), IFN-β (D), TNF-α (E), and IFN-γ (F), were detected from the serum samples of Bama minipigs infected with ASFV SY-1 at doses of 10^3^ HAD_50_ every day after infection.

To evaluate the cytokine expression with Bama minipigs infected with the ASFV SY-1 strain, serum samples of Bama minipigs infected with 10^3^ HAD_50_ were collected every day after infection for further detection by enzyme-linked immunosorbent assay (ELISA). The expression of interleukin 6 (IL-6) was upregulated 2.15-fold at 6 dpi compared to 0 days (Fig. 5A). The expression of IL-8 was no significant change (Fig. 5B). The expression of IL-1β, interferon beta (IFN-β), tumor necrosis factor alpha (TNF-α), and IFN-γ was upregulated by 3.05-fold, 7.58-fold, 12.33-fold, and 11.76-fold, respectively, at 6 dpi compared to 0 days (Fig. 5C to F). These results proved that cytokines showed a persistent rise after ASFV infection until death.

### Organ lesions of Bama minipigs infected with ASFV SY-1.

**FIG 6:**
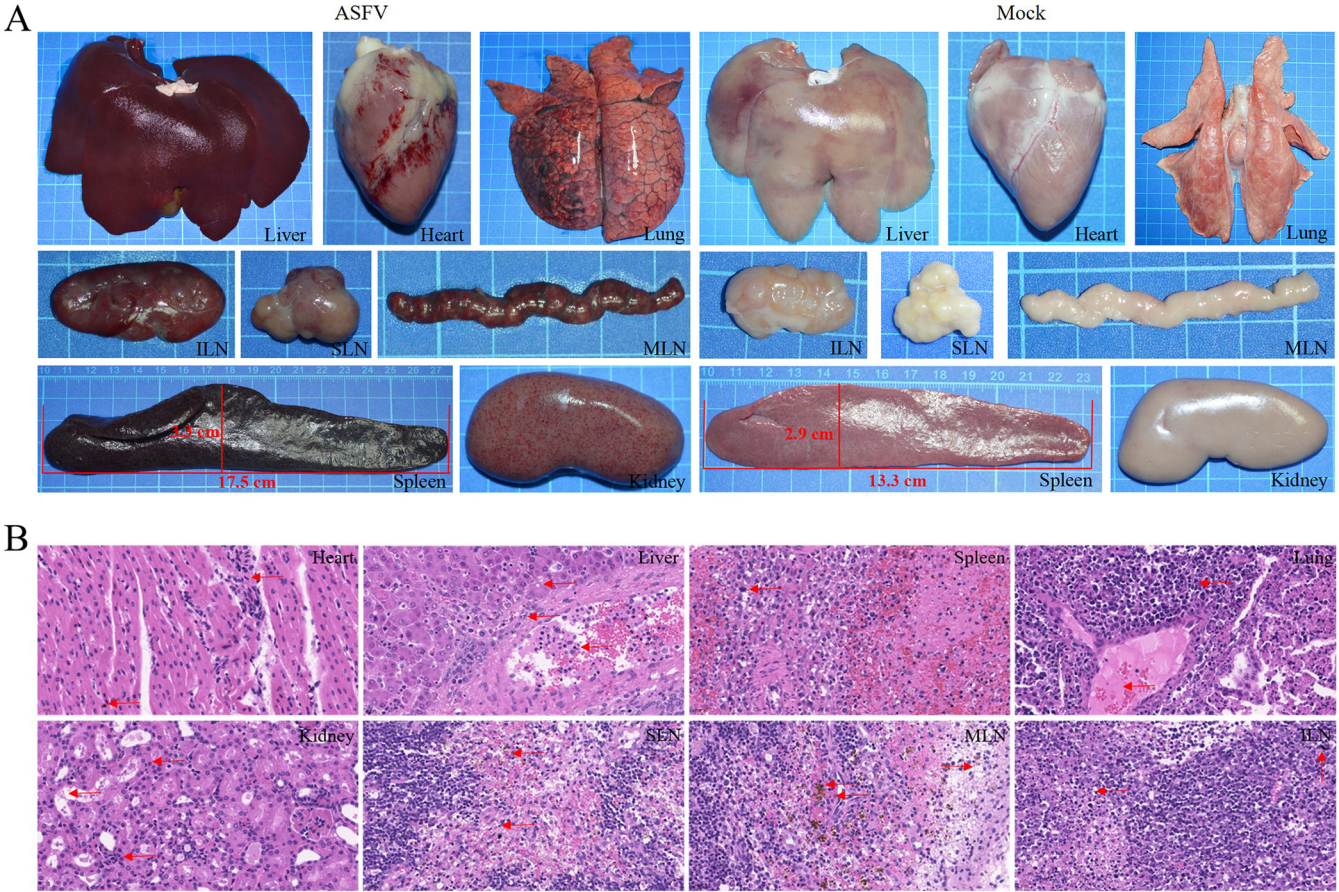
Gross lesions of different tissues of Bama minipigs that died as a result of infection with African swine fever virus (ASFV) SY-1. (A) Tissue samples, including those of the heart, liver, spleen, lung, kidney, SLN, ILN, and MLN, from Bama minipigs infected with ASFV SY-1 at 10^3^ HAD_50_ compared to the mock group. (B) Hematoxylin and eosin staining assay was used to analyze subtle pathological changes of the different tissue samples from Bama minipigs infected with ASFV SY-1 at 10^3^ HAD_50_. Red arrows indicate lesions of different tissues. SLN, submaxillary lymph node; ILN, inguinal lymph node; MLN, mesenteric lymph node.

Pigs infected with ASFV were found to have multiple organ lesions after death (7). Upon observation, organ lesions were found in the liver, heart, lung, spleen, kidney, mesenteric lymph nodes (MLNs), submaxillary lymph nodes (SLNs), and inguinal lymph nodes (ILNs) collected from Bama minipigs inoculated with 10^3^ HAD_50_. When comparing the pigs of ASFV infection groups to the mock group, we found hemorrhage in the heart, mesenteric lymph nodes, submaxillary lymph nodes, and inguinal lymph nodes, while the kidneys showed needle-shaped bleeding points after ASFV infection (Fig. 6A). The spleens of Bama minipigs presented with splenomegaly and hemorrhage, which are classic characteristic of ASFV infection, and the average spleen length and width increased by 4.2 cm and 0.9 cm, respectively, compared to the mock group (Fig. 6A).

To further analyze the pathological changes in the organs of ASFV-infected Bama minipigs, a hematoxylin and eosin staining assay was performed. As shown in Fig. 6B, small amounts of inflammatory cell infiltration andccc hemosiderin deposition were observed in the heart. Tissue cavitation, a small quantity of inflammatory cells, and nuclear fragmentation were detected in the liver. Unclear boundaries between the red and white pulp and the heteromorphic nuclear cells in the parenchyma could be discerned in the spleen. In the lung parenchyma, partial inflammatory cell infiltration and partial protein deposition were seen. Acute tubular necrosis was caused by ischemia, the tubular epithelium was broken due to necrosis, and a small number of inflammatory cells had infiltrated the renal tubulointerstitium. A small quantity of inflammatory cell infiltration and hemosiderin deposition was visible in submaxillary lymph nodes and mesenteric lymph nodes. Localized areas in mitotic phases, dark inflammatory cells, and individual sites of melanin deposition were seen in inguinal lymph nodes. These results demonstrated that Bama minipigs infected with ASFV also presented with severe organ lesions.

### Detection of virions infected with ASFV SY-1.

**FIG 7:**
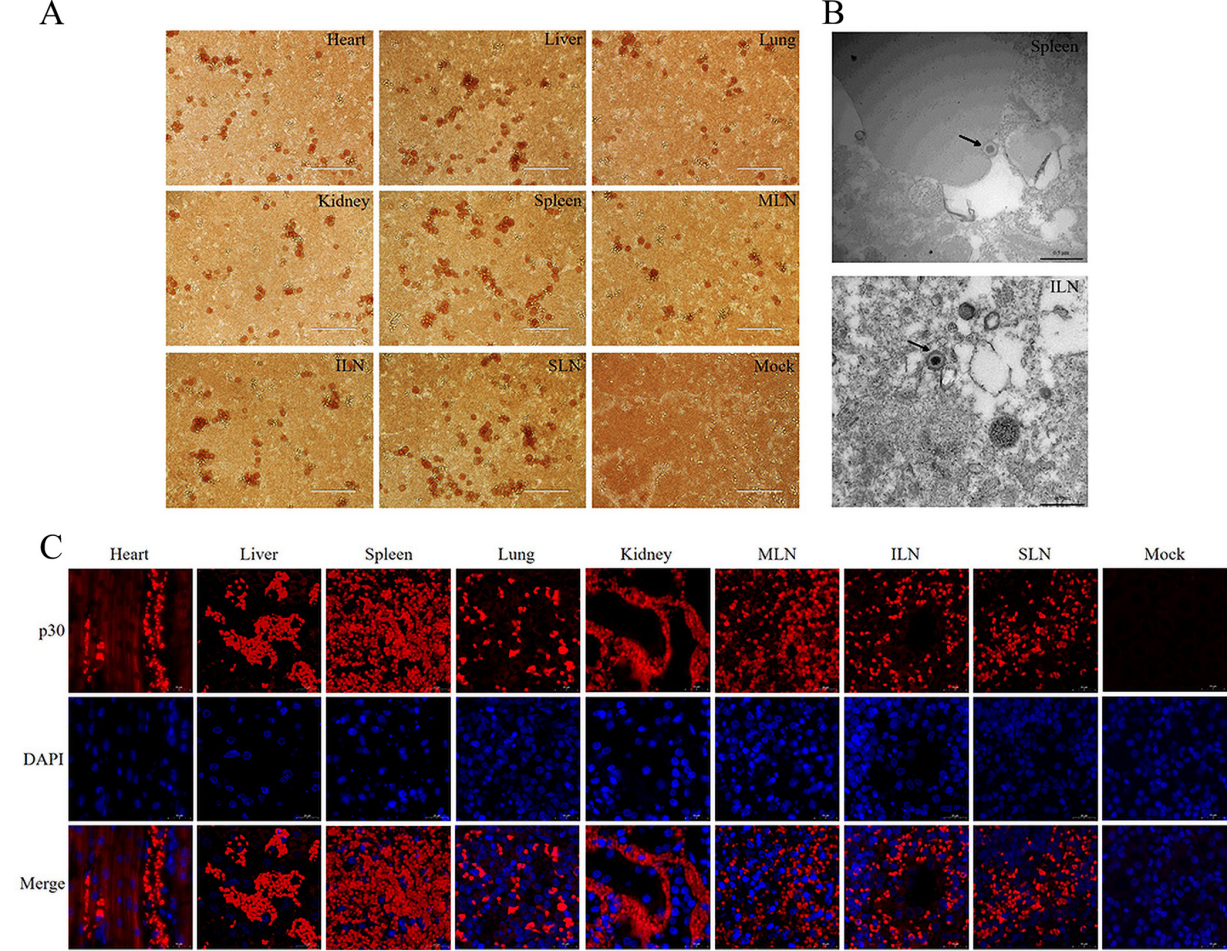
African swine fever virus (ASFV) infectious particles of different tissues were determined from Bama minipigs that died as a result of ASFV SY-1 infection. (A) Infectious virus particles of the heart, liver, spleen, lung, kidney, SLN, ILN, and MLN from Bama minipigs infected with ASFV SY-1 at 10^3^ HAD_50_ were detected by HAD assay. (B) ASFV particles of the spleen and ILN from Bama minipigs infected with ASFV SY-1 were observed by electron microscopy. (C) The antibodies of ASFV p30 were used to detect virions by immunohistochemistry in diverse tissues from Bama minipigs infected with ASFV SY-1. SLN, submaxillary lymph node; ILN, inguinal lymph node; MLN, mesenteric lymph node.

Subsequently, HAD, electron microscopy, and immunohistochemical (IHC) assay were performed to detect the ASFV virions in the organs of Bama minipigs infected with 10^3^ HAD_50_. Homogenates of the liver, heart, lung, spleen, kidney, mesenteric lymph nodes, submaxillary lymph nodes, and inguinal lymph node-inoculated cells, which exhibited ASFV-unique HAD characteristics (Fig. 7A). ASFV virions were obviously observed in the spleen and inguinal lymph nodes by electron microscopy, which presented a 20-anhedral symmetric structure (Fig. 7B). Furthermore, the antibodies of ASFV p30 were used to detect virions in various organs by IHC assay, and the highest quantity of virions was in the spleen (Fig. 7C). Hence, these results revealed that we successfully established a model of ASFV infection in Bama minipigs, which could be used to evaluate the efficacy of vaccines and antiviral drugs *in vivo* in the future.

### Differentially expressed genes in the spleen and inguinal lymph node during ASFV SY-1 infection.

**FIG 8:**
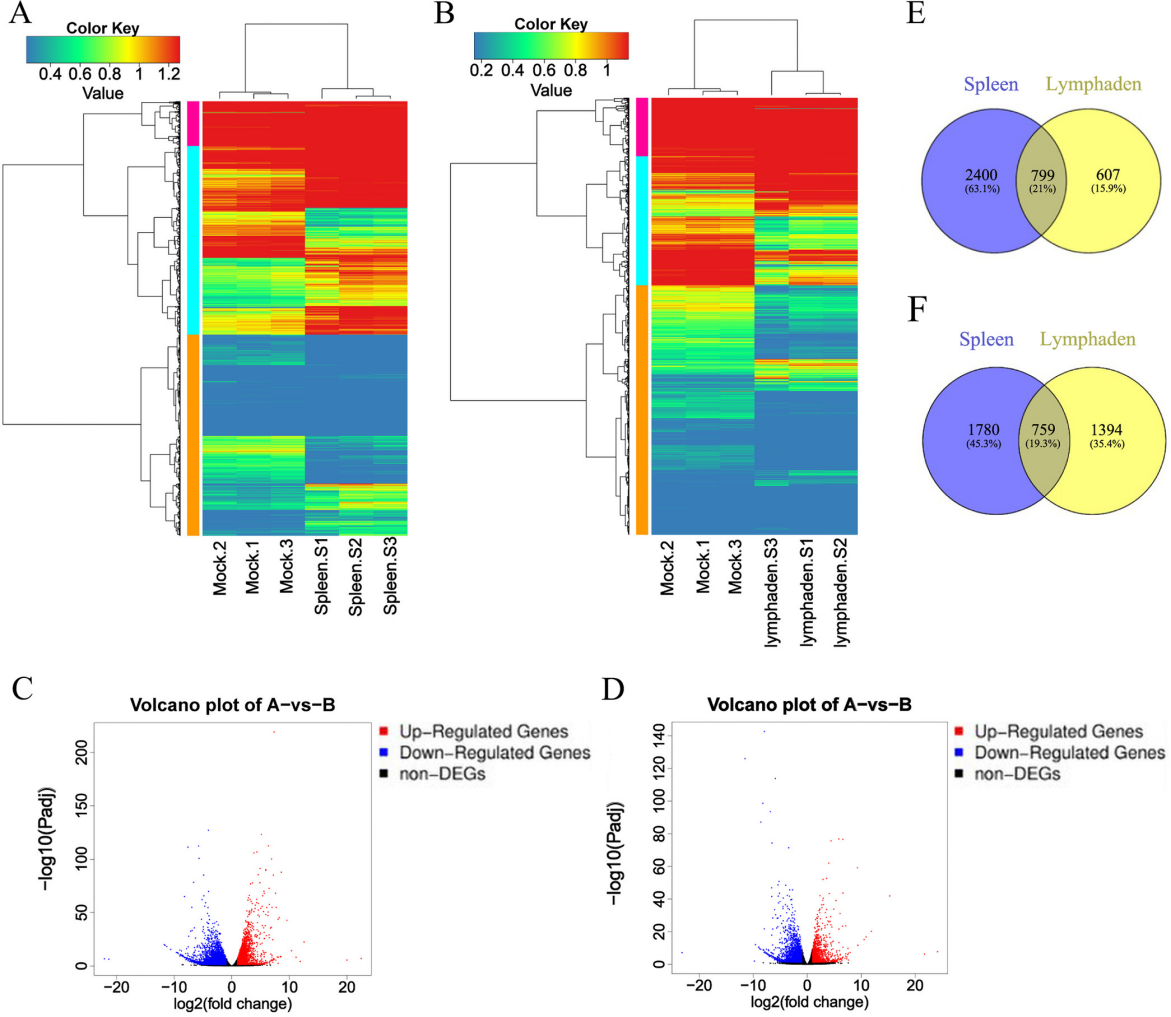
Differentially expressed genes (DEGs) were identified by transcriptomic analyses between the African swine fever virus (ASFV)-infected and noninfected spleen and inguinal lymph node samples. (A and C) Heatmap (A) and volcano plot (C) of spleen samples from Bama minipigs infected ASFV SY-1 at 10^3^ HAD_50_ compared to the mock group. (B and D) Heatmap (B) and volcano plot (D) of inguinal lymph node samples from Bama minipigs infected ASFV SY-1 at 10^3^ HAD_50_ compared to the mock group. A Venn diagram was used to analyze the common upregulated (E) and downregulated (F) DEGs in the spleen and inguinal lymph node samples.

To further analyze the host responses of Bama minipigs to ASFV infection, we chose the spleen and inguinal lymph nodes from Bama minipigs infected with ASFV at 10^3^ HAD_50_ and the mock group for further transcriptomic analysis. The transcriptomic data were filtered by the thresholds of *P* of value <0.05 and |log_2_(fold change)| of >1.0. Under these criteria, 5,738 differentially expressed genes (DEGs; 3,199 upregulated and 2,539 downregulated) were identified in the spleen samples of the ASFV-infected group compared to the mock group (Fig. 8A and C). A list of the DEGs is provided in Table S3. The upregulation of STAT2 [|log_2_ (fold change)| = 11.87] was the highest among all upregulated DEGs in spleen samples, and the downregulation of LYPD3 [|log_2_ (fold change)|, −22.09] was the highest among all downregulated DEGs in spleen samples of the ASFV-infected group compared to the mock group. Subsequently, 3,559 DEGs (1,406 upregulated and 2,153 downregulated) were identified in the inguinal lymph node samples of the ASFV-infected group compared to the mock group (Fig. 8B and D). A list of the DEGs is provided in Table S4. The upregulation of LYZ [|log_2_ (fold change)|, 24.13] was the highest among all upregulated DEGs in inguinal lymph node samples, and the downregulation of APOR [|log_2_ (fold change)|, −11.52] was the highest among all downregulated DEGs in inguinal lymph node samples of the ASFV-infected group compared to the mock group. All upregulated and downregulated DEGs from the spleen and inguinal lymph node samples were analyzed for common DEGs by Venn diagram. There were 799 common upregulated genes, accounting for 21% of all upregulated DEGs (Fig. 8E); meanwhile, there were 759 common downregulated genes, which account for 19.3% of all downregulated DEGs (Fig. 8F) in the spleen and inguinal lymph nodes. The common upregulated and downregulated DEGs are provided in Table S5 and Table S6, respectively.

To further validate the DEGs identified in the transcriptome analysis, we performed real-time quantitative PCR (RT-qPCR) for 12 common DEGs, including *STAT2*, *CCRL2*, *RSAD2*, *TMED3*, *CXCL9*, *USP18*, *PHGDH*, *SPAG5*, *CAMK1G*, *FADS3*, *TRDMT1*, and *AMY2* (Fig. S1). We found that the expression levels of *STAT2*, *CCRL2*, *RSAD2*, *TMED3*, *CXCL9*, *USP18*, *PHGDH*, and *SPAG5* were higher, while those of *CAMK1G*, *FADS3*, *TRDMT1*, and *AMY2* were lower in the ASFV-infected than in the mock group, consistent with the transcriptomic data.

### GO and KEGG enrichment analyses for differentially expressed genes.

**FIG 9:**
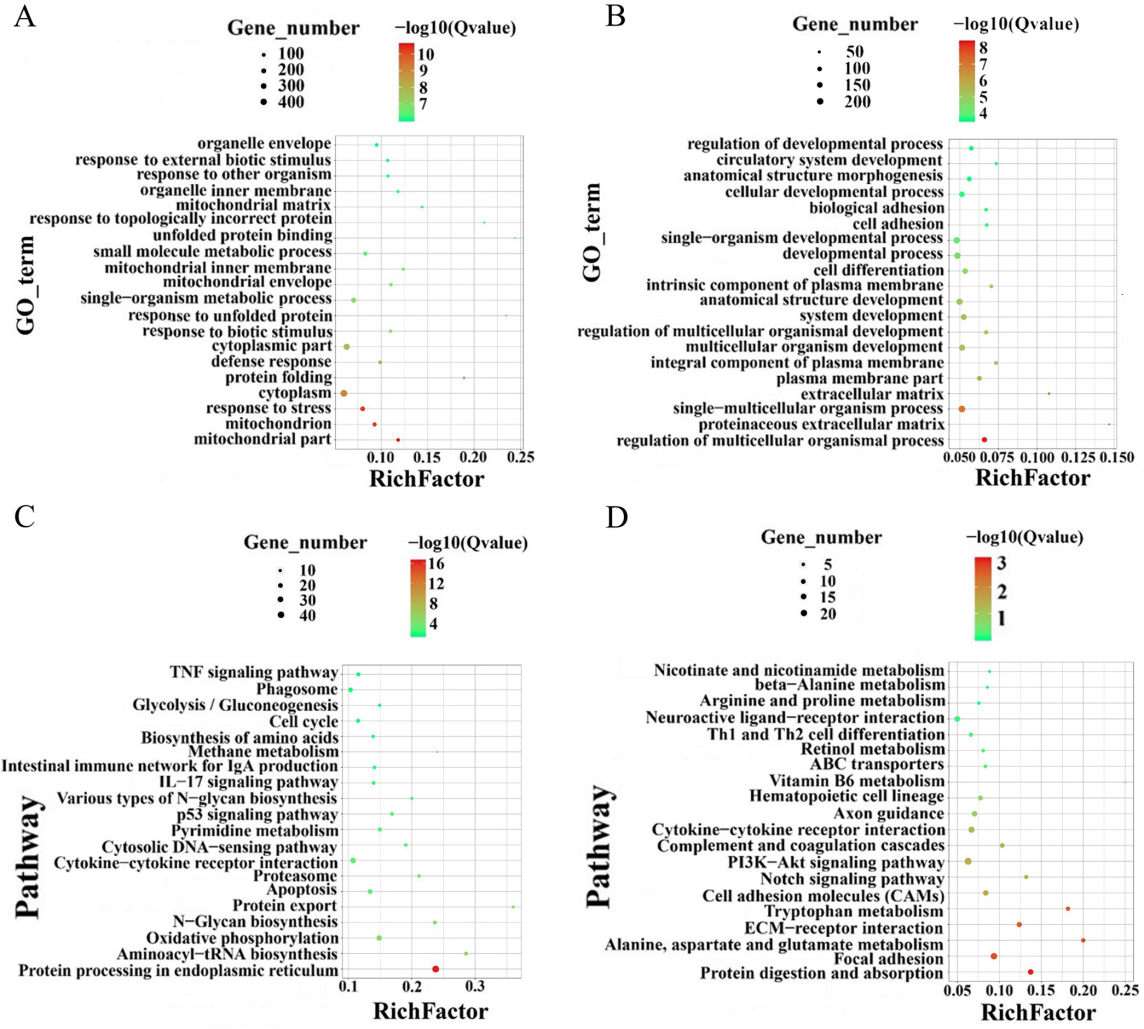
Gene ontology (GO) enrichment and Kyoto Encyclopedia of Genes and Genomes (KEGG) pathway analysis of the common differentially expressed genes (DEGs) identified by transcriptomic analysis between the African swine fever virus (ASFV)-infected and noninfected spleen and inguinal lymph node samples. (A and B) GO enrichment analysis of common upregulated (A) and downregulated (B) DEGs in spleen and inguinal lymph node samples. (C and D) KEGG pathway analysis of common upregulated (and downregulated DEGs in spleen and inguinal lymph node samples.

Gene ontology (GO) and Kyoto Encyclopedia of Genes and Genomes (KEGG) enrichment analyses were used to cluster the common upregulated and downregulated DEGs. The common upregulated and downregulated DEGs concentrated on cytoplasm and metabolic process by GO enrichment analyses (Fig. 9A and B). The pathways of common upregulated and downregulated DEGs focused primarily on signal transduction and the immune system (Fig. 9C and D). Among common upregulated DEGs, most genes (up to 44) were enriched in protein processing in the endoplasmic reticulum signaling pathway, followed by 29 genes enriched in the cytokine-cytokine receptor interaction signaling pathway. Among common downregulated DEGs, the most genes (up to 24) were enriched in the PI3K-Akt signaling pathway, followed by 20 genes enriched in the focal adhesion signaling pathway. These results demonstrated that infection of Bama minipigs with ASFV leads to change in multiple host genes, which participate in metabolic and immune pathways.

## DISCUSSION

In this study, ASFV was detected in homogenates of wild boar spleens, and its presence was further confirmed by HAD assays, electron microscopy, Western blotting, and indirect immunofluorescence assays in PBMCs and PAMs. We successfully isolated the ASFV SY-1 strain. The ASFV SY-1 isolate belonged to genotype II according to sequence analysis of the ASFV B646L gene encoding the p72 protein, and the whole gene sequences were generally consistent with Pig/HLJ/18, which is an epidemic strain in China with only seven base site mutations. Bama minipigs must be kept in a negative-pressure isolator of an ABSL-3 laboratory to assess the pathogenicity of ASFV SY-1. We found that the dose of 0.1 HAD_50_ led to all pigs dying at 14 dpi, which showed high virulence of the isolate. Further transcriptomic analysis revealed that signal transduction, metabolism, and the immune-related pathway were remarkably enriched in spleen and inguinal lymph nodes when infected groups were compared with mock groups.

In China, ASFVs of primary genotype I and genotype II were isolated successfully from domestic pigs (7, 14). However, there are few reports about ASFVs from wild boars. Li et al. previously reported that ASFV from wild boars in China and the phylogenetic analysis showed that it belonged to genotype II and that it contained no additional tandem repeat sequences between the I73R and the I329L genes (15). Here, we also isolated ASFV belonging to genotype II from wild boar. Although two strains were isolated from wild boars, the whole viral genome sequences were inconsistent. The I73R and I329L genes of our isolated strain had complete, rather than deleted, tandem repeat sequences as previously reported, but there were seven base site mutations in the left repeat region and MGF110-13L, MGF505-9R, F778R, C717R, and G1340L genes compared to Pig/HLJ/18, which has been the prevalent strain in China. Therefore, it is vital to isolate ASFV strains from domestic pigs and wild boars to better understand the evolution of the virus and control its transmission.

A previous study found that specific-pathogen-free (SPF) Large White and Landrace-crossed pigs were infected with various doses of ASFV Pig/HLJ/18, ranging from 10^3.5^ HAD_50_ to 10^6.5^ HAD_50_ and started to show early disease signs at 3 to 5 dpi, and all of the animals died at 6 to 9 dpi (7). All SPF pigs infected with Pig/HLJ/18 at the highest dose of 10^6.5^ HAD_50_ died at 7 dpi, and all Bama minipigs infected with ASFV SY-1 at the highest dose of 10^4^ HAD_50_ died at 5 dpi (Fig. 3C; see Table S2 in the supplemental material), which illustrates that Bama minipigs were susceptible to ASFV infection.

The tissues and organs of domestic pigs and wild boars infected with ASFV show a variety of characteristic pathological changes, including splenomegaly, hemorrhage, and necrosis of lymphocytes within splenic nodule; petechial hemorrhages on the kidney with focal lymphocytic infiltration in the cortex and necrotic cells in the tubular epithelium and glomeruli; and submaxillary, inguinal, and mesenteric lymph nodes showing marked diffuse hemorrhages (7, 16). Here, we found that Bama minipigs infected with ASFV exhibited similar characteristic organ lesions to domestic pigs and wild boars infected with ASFV (Fig. 6). Hence, Bama minipigs can be used as a model for ASFV infection in negative-pressure isolators of ABSL-3 laboratories.

Spleen and inguinal lymph node samples of ASFV-infected Bama minipigs were subjected to transcriptomic analysis, revealing that immune-related, metabolic, and inflammatory response pathways were significantly upregulated (Fig. 9). As previously reported, tissue samples of domestic pigs infected with ASFV were also subjected to transcriptomic and proteomic analyses, revealing enrichment of innate immune response, metabolic regulatory, and inflammatory response pathways (17). A genome-wide transcriptomic analysis of ASFV-infected PAMs revealed that host immune response and metabolic processes were upregulated (18). A proteomic analysis of ASFV-infected PAMs also showed that immune system response, complement and coagulation cascade, and metabolic processes are crucial pathways during infection (19). Our results showed that the well-known interferon signaling pathway-related DEGs, including cGAS, TBK1, STAT1, STAT2, interferon-stimulated gene 20 (ISG20), ISG15, and Mx1, were identified, which were found in previous studies after ASFV infection (20). The A137R, pI215L, and M1249L proteins of ASFV inhibit interferon production through the interferon function of TBK1 (21–23). ISG20 exhibits strong RNase properties, as it belongs to the large family of DEDD exonucleases, which inhibit various types of viral replication, including hepatitis B virus and human immunodeficiency virus type 1 (24–26). Whether ISG20 affects ASFV infection deserves further study. The expression of IL-6 and IL-1β was upregulated in our result, which is consistent with previous studies (27), and there is a typical cytokine storm after ASFV infection (28). Therefore, infection with ASFV caused changes in the host genes of Bama minipigs, and these DEGs and enriched pathways were consistent with previous *in vivo* or *in vitro* studies. These DEGs may be potential antiviral targets against ASFV infection, and their detailed mechanism needs to be further explored.

In conclusion, we successfully isolated ASFV SY-1 from wild boars and established the first model of ASFV infection in Bama minipigs in the negative-pressure isolator of a small ABSL-3 laboratory. Because Bama minipigs are miniature swine that grow slowly, they are suitable for long-term rearing and are very sensitive to ASFV infection. Therefore, Bama minipigs are a suitable animal model for small ABSL-3 laboratories, and their application should accelerate the research into anti-ASFV drugs and vaccines and further improve understanding of their pathogenesis.

## MATERIALS AND METHODS

### Cells and virus isolation.

Primary porcine alveolar macrophages (PAMs) were obtained from 1-month-old pigs (both ASFV antigen and antibody are negative) by bronchoalveolar lavage as previously described (29). Peripheral blood mononuclear (PBMC) cells from EDTA-treated swine blood were collected by using a pig PBMC isolation kit (TBD Sciences, Tianjin, China). Human embryonic kidney 293T (HEK-293T) cells were conserved in our laboratory. PAMs and PBMCs were grown in RPMI 1640 culture medium (Gibco, Waltham, MA, USA), and 293T cells were grown in Dulbecco’s modified Eagle’s medium (Gibco). These were supplemented with 10% fetal bovine serum (FBS) (Gibco, USA), 100 μg/mL streptomycin, and 100 IU/mL penicillin at 37°C in a 5% CO_2_ atmosphere.

Spleen samples were obtained from wild boars infected with ASFV in Shennongjia Forest, Hubei Province, China, which was reported by the Ministry of Agriculture and Rural Affairs of China. The homogenized wild boar spleen samples were tested by OIE-recommended standard PCR and hemadsorption (HAD) assay. The positive samples were used to aseptically inoculate primary PAMs. At 5 to 7 days postinfection (dpi), the cell supernatants were collected for qPCR detection, which were used to again inoculate PAMs for ASFV proliferation. Each generation of ASFV stock was tested to confirm the absence of bacteria or other viruses such as porcine circoviruses (PCVs), classical swine fever virus (CSFV), porcine respiratory and reproductive syndrome virus (PRRSV), and pseudorabies virus (PRV). The isolated ASFV strain was named SY-1. The ASFV was propagated and stored at −80°C until use.

### Hemadsorption assay.

The HAD assay was performed according to a previous study, with minor adjustments (30). Briefly, PBMCs were cultured in 96-well plates, and the viruses were added to the 96-well plates and titrated in eight replicates using 10-fold serial dilutions. Cells were observed as having characteristic rosette formation for viral titer detection at 7 dpi, and 50% HAD doses (HAD_50_) were calculated using the Reed-Muench method (31).

### Immunofluorescence assay and Western blot analysis.

PAMs were seeded in 24-well plates and infected with ASFV at a multiplicity of infection (MOI) of 0.1 for 36 h. Next, cells were fixed with 4% paraformaldehyde and permeabilized with 0.1% Triton X-100. After cells were blocked with 1% bovine serum albumin (BSA) for 1 h at 37°C and then incubated with an ASFV p30 monoclonal antibody preserved in our laboratory at 37°C for 2 h. After washing thrice with phosphate-buffered saline (PBS) for 5 min each time, the cells were incubated with CoraLite488-conjugated goat anti-mouse IgG (H+L) (Proteintech, Wuhan, China) at 37°C for 1 h. Samples were visualized with the EVOS FL Auto system (Thermo Fisher Scientific, Waltham, MA, USA).

For Western blot analysis, briefly, the proteins of cells were separated by SDS-PAGE and later transferred to a nitrocellulose (NC) filter membrane (GE Healthcare, USA) in transfer buffer (100 mM Tris, 190 mM glycine, and 10% methanol). The membrane was blocked by 1% BSA for 1 h at 37°C. After the membrane was incubated with an ASFV p30 monoclonal antibody preserved in our laboratory at 37°C for 2 h followed by washing three times with Tris-buffered saline with Tween 20 (TBST). Finally, the membrane was incubated with horseradish peroxidase (HRP)-conjugated AffiniPure goat anti-mouse IgG (H+L) (Proteintech) secondary antibody at 37°C for 1 h and washed three times with TBST. The specific bands of the membrane were visualized using Western blotting ECL reagent (Advansta, USA).

### Enzyme-linked immunosorbent assay.

Serum samples from pigs were collected and assayed for porcine IL-6, IL-1β, IL-8, TNF-α, IFN-β, and IFN-r using porcine IL-6, IL-1β, IL-8, TNF-α, IFN-β, and IFN-γ ELISA kits (Solarbio), respectively. The measured value was compared with the standard according to the manufacturer’s instructions.

### Electron microscopy.

To observe the virions, primary PAMs were infected with ASFV (MOI = 0.1) for 72 h, and tissue samples consisting of spleen and inguinal lymph nodes were infected with ASFV and collected for electron microscopy as described previously (7). In brief, samples were fixed in 2.5% glutaraldehyde (pH = 7.2) for 24 h at 4°C followed by 1% OsO_4_ (pH = 7.4) at 4°C for 2 h, dehydrated at 4°C in stepwise acetone, and embedded in 812 Epon resin. Thin sections of 80 nm were stained with 1% uranyl acetate (pH = 6.5) and 1% lead citrate (pH = 7.2). The samples were observed using an H-7650 (Hitachi, Tokyo, Japan) at 100 kV.

### Real-time quantitative PCR.

To validate the transcriptome data of spleen and inguinal lymph nodes after ASFV infection, real-time quantitative PCR (RT-qPCR) was performed, and primers are listed in Table S1 in the supplemental material. In brief, the total mRNA of homogenized tissues was extracted using an RNA extraction kit (Magen, Guangzhou, China) and followed by reverse transcription by means of HiScript reverse transcriptase (Vazyme, Nanjing, China). qPCRs were conducted on a QuantStudio 6 Flex system (Life Technologies, Carlsbad, CA, USA) as follows: 95°C for 10 min, followed by 40 cycles of 95°C for 15 s, 60°C for 15 s, and 72°C for 15 s.

To calculate the copy number of ASFV genomic DNA from swabs, cell supernatants, and tissue homogenates, qPCR was performed as described previously (32). In brief, ASFV genomic DNA samples were extracted using EasyPure viral DNA/RNA kit (TransGen Biotech, Beijing, China) and qPCR was performed on a QuantStudio 6 system (Applied Biosystems, Waltham, MA, USA).

### Viral genome sequencing and genetic analysis.

The ASFV genome was sequenced first by next-generation DNA sequencing and alignment with the ASFV Pig/HLJ/18 genome, but there were gap regions in the sequencing results. Therefore, the gap regions were amplified by PCR and sequenced using segment-specific primers. Finally, the ASFV complete genome was spliced.

The genotype of the virus, based on the B646L gene encoding the p72 protein, was analyzed using MEGA 7.0 software.

### Animal experiments and histological analysis.

All experiments on live ASFV manipulations and animal infection were carried out in the ABSL-3 laboratory of Huazhong Agricultural University. All experiments were approved by the Ministry of Agriculture and Rural Affairs in China. All animal-related study processes were performed according to the Care and Use of Laboratory Animals of the Research Ethics Committee.

Bama minipigs were used in the animal experiment. Before the ASFV challenge, the pigs were tested to ensure that they did not have PCV, PRRSV, PRV, or CSFV infections. Seven-week-old pigs were randomly divided into eight groups with six pigs in each group, including seven experimental groups and one negative-control group, and placed in a negative-pressure isolator of the ABSL-3 lab of Huazhong Agricultural University. The pigs of the experimental groups were intramuscularly inoculated with ASFV at doses of 10^4^ HAD_50_, 5 × 10^3^ HAD_50_, 10^3^ HAD_50_, 5 × 10^2^ HAD_50_, 10 HAD_50_, 1 HAD_50_, and 0.1 HAD_50_, respectively. Each pig was observed daily for disease signs, including anorexia, depression, fever, purple skin discoloration, staggering gait, diarrhea, cough, and body temperature changes, which were recorded throughout the experiment. Blood, nasal, oral, and rectal swabs were collected at the indicated day postinfection for viral detection. The pigs were instantly dissected upon death. Tissue samples were observed for pathological changes, and samples were retained from each necropsied pig for further analysis. The viral titers of samples were determined by qPCR assay. There was only one isolated ASFV strain of progeny from the spleen of ASFV-infected Bama minipigs that was sequenced. The ASFV progeny strain was named SY-2.

The collected tissue samples from ASFV-infected Bama minipigs were fixed in a 4% buffered formalin solution, embedded in paraffin, and sectioned at 4 μm. The histopathological examination (HE) staining was performed according to a conventional procedure in different tissues.

Immunohistochemistry (IHC) was conducted according to a previous study (33). In brief, tissue samples from ASFV-infected Bama minipigs were fixed in a 4% buffered formalin solution, embedded in paraffin, and sectioned at 4 μm. After hydration, sections of tissue were incubated in trypsin solution (0.1%) in the presence of calcium chloride dihydrate (3 M) for 20 min at 37°C. Slides were incubated with mouse monoclonal antibodies against ASFV p30 protein and preserved in our laboratory at 4°C overnight. The sections were incubated with CoraLite594-conjugated goat anti-mouse IgG (H+L) (Proteintech).

### RNA sequencing.

For transcriptomic analysis, RNA was extracted from the spleens and inguinal lymph nodes of ASFV-infected and mock-infected Bama minipigs. The quality and integrity of extracted total RNA were analyzed using 1% (wt/vol) agarose gel and a Nanodrop 2000C system (Thermo Fisher Scientific, Waltham, MA, USA). All RNA samples were delivered to Frasergen (Wuhan, China) for further transcriptome sequencing. Generated RNA data were clustered for bioinformatic analysis by gene ontology (GO) and Kyoto Encyclopedia of Genes and Genomes (KEGG) pathway enrichment analyses.

### Statistical analysis.

Data were analyzed for statistical significance by Student's *t* test. Significant differences were considered as follows: *, *P* < 0.05; **, *P* < 0.01; ***, *P* < 0.001; and ****, *P* < 0.0001.

### Data availability.

The transcriptome data files were uploaded into the Gene Expression Omnibus (GEO) database of the National Center for Biotechnology Information (NCBI) with accession no. GSE215197. The genome of ASFV SY-1 and SY-2 has been uploaded to the NCBI database (GenBank accession nos. OM161110 and OP612151).
