# Supplementary material for: Infection Characteristics and Transcriptomics of African Swine Fever Virus in Bama Minipigs
Source: Microbiol Spectr. 2022 Nov 29;10(6):e03834-22. doi: 10.1128/spectrum.03834-22 (PMC9769931; doi:10.1128/spectrum.03834-22)
Supplement: Supplemental file 1 — Fig. S1 and Tables S1 to S6. Download spectrum.03834-22-s0001.pdf, PDF file, 5.9 MB [file spectrum.03834-22-s0001.pdf]

Supplementary Materials

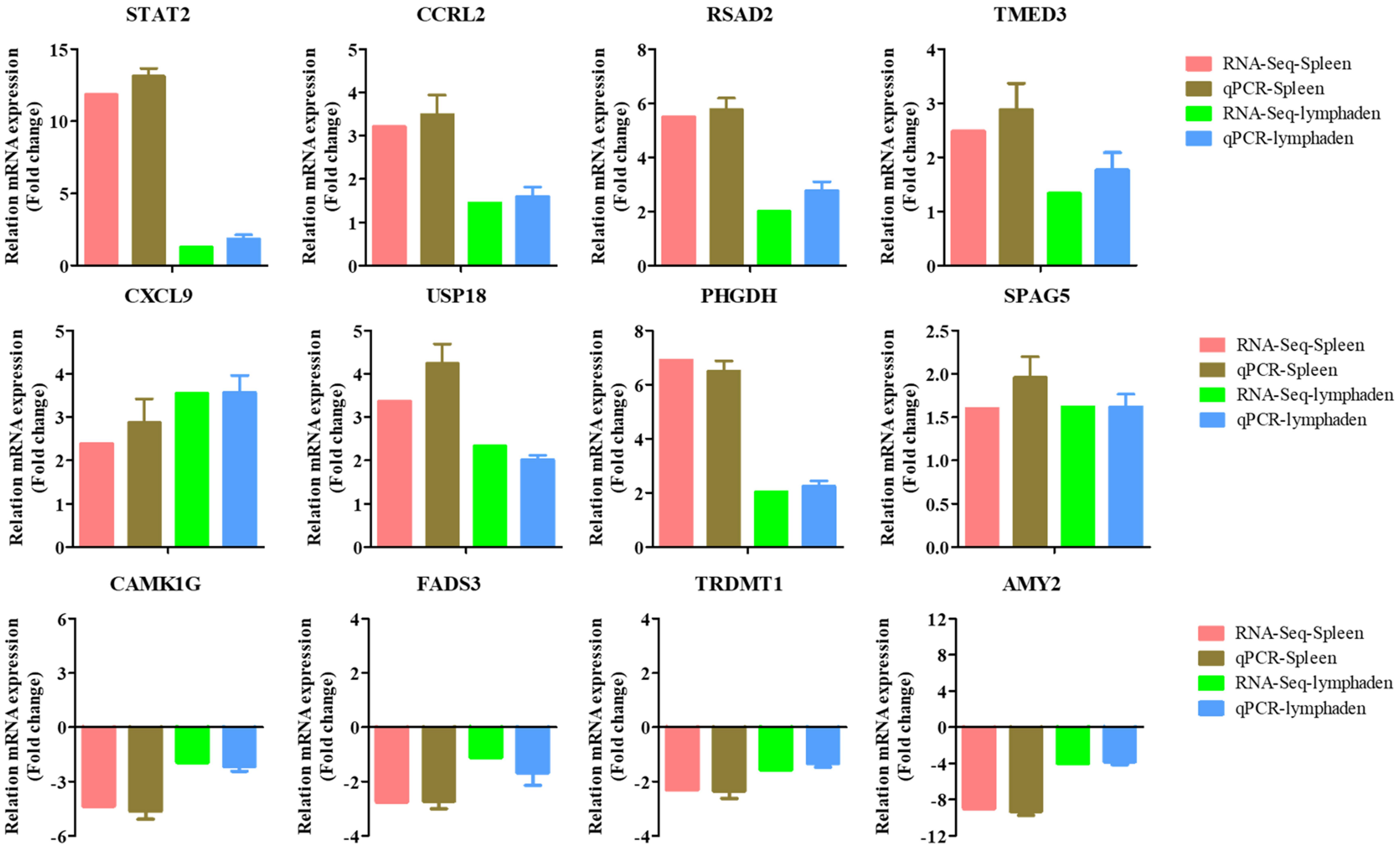

Supplementary Figure 1. Verification of the RNA-sequencing results by real-time quantitative PCR (RT-qPCR). The GAPDH gene was used for normalization.

Supplementary Table 1. List of primers of real-time quantitative PCR (RT-qPCR) used in this study

Target gene	Primer sequence (5′-3′)
STAT2-F	GGATTGAGTAGCGTGGTT
STAT2-R	AGTCTTGAGGATGAGGGG
CCRL2-F	GGCGAGCCCCTGTGTAAA
CCRL2-R	CATCCTGGTGGCCGAGAA
RSAD2-F	CGGGCAGGGGGTGATAGG
RSAD2-R	TGGCGGGTGAAGTGGTAA
TMED3-F	TGGACTGCTATGTGGAAG
TMED3-R	ATGAGAGAAGGTGGAAAA
CXCL9-F	AGTGTTGCCTTGCTTTTG
CXCL9-R	CTTTGGCTGGTGTTGATG
USP18-F	TGAGGAGCAGAGGAGAAA
USP18-R	CAGACGGTGAGGTAGAGC
PHGDH-F	CAAATGGGAGCGGAAAAA
PHGDH-R	GACCTCTGGAGCAATGAT
SPAG5-F	CTCAGCATCTTCCGCACA
SPAG5-R	CAAAACAGCAGGACACCA
CAMK1G-F	CCATTGGCGTCATCACTT
CAMK1G-R	CTCCTTCGGGTCCTTCTC
FADS3-F	AGCAGTTCGTGATGGGGC
FADS3-R	GCTGGTGGTTGTAGGGTA
TRDMT1-F	ATTCAAGGCTACGGTATT
TRDMT1-R	TGTTTTTGTGGATGTTCG
AMY2-F	TTTCCAGCAGTCCCATAC
AMY2-R	CGCACATAATCTTTTTCC
GAPDH-F	ACATGGCCTCCAAGGAGTAAGA
GAPDH-R	GATCGAGTTGGGGCTGTGACT

Supplementary Table 2. Replication and virulence of African swine fever virus SY-1 in Bama mini-pigs

Virus dose (HAD_{50})	The earliest time viral genomic DNA was detected in swabs by qPCR day post infection (dpi)			Death time (dpi)	Highest titre of virus in organs of dead pigs (Copy number of the viral p72 gene per gram of tissue)					
	Oral swab	Rectal swab	Nose swab		Liver	Spleen	Lung	Kidney	Groin lymph node	Submaxillary lymph node
1×10^4	2	2	2	5-7	1.3×10^8	4.8×10^8	1.6×10^7	1.5×10^7	5.8×10^7	7.3×10^7
5×10^3	2	2	2	5-7	1.8×10^8	7.4×10^8	7.2×10^6	2.4×10^7	2.2×10^7	4.1×10^6
1×10^3	2	2	2	5-7	1.5×10^8	4.3×10^8	1.8×10^7	4.3×10^6	3.9×10^7	1.7×10^7
5×10^2	2	3	2	5-7	1.2×10^8	3.2×10^8	7.5×10^6	2.7×10^7	2.3×10^7	4.3×10^7
10	3	3	3	5-9	1.6×10^8	2.5×10^8	3.2×10^7	1.1×10^7	4.5×10^7	5.3×10^7
1	3	4	3	6-10	2.7×10^8	8.3×10^8	6.2×10^6	7.4×10^7	3.1×10^6	7.9×10^7
0.1	4	4	4	7-14	5.3×10^8	1.4×10^9	5.8×10^7	8.6×10^7	4.9×10^7	8.4×10^7

Supplementary Table 3. The differentially expressed genes between the infected and mock group from spleen samples

GeneID	logFC	Pvalue
ENSSSCG00000000396	11.86978846	7.27E-06
ENSSSCG00000021092	11.13200406	5.58E-10
ENSSSCG00000039252	10.35267174	1.83E-16
ENSSSCG00000014985	9.562029176	4.56E-46
ENSSSCG00000010948	8.580168002	8.46E-92
ENSSSCG00000029675	8.506445752	2.03E-10
ENSSSCG00000023305	8.228962933	1.56E-48
ENSSSCG00000036825	8.071331848	6.57E-10
ENSSSCG00000033183	8.025273594	4.91E-47
ENSSSCG00000009239	8.022332102	0.000819352
ENSSSCG00000029419	7.882375115	2.98E-10
ENSSSCG00000009921	7.70007188	2.53E-58
ENSSSCG00000025717	7.638003127	4.49E-07
ENSSSCG00000038521	7.589582768	6.56E-72
ENSSSCG00000030879	7.343758097	4.49E-08
ENSSSCG00000005287	7.342952587	2.90E-224
ENSSSCG00000039672	7.212442811	6.07E-89
ENSSSCG00000006238	7.178361864	4.22E-08
ENSSSCG00000047793	7.108651757	7.90E-05
ENSSSCG00000013102	6.95984092	2.67E-58
ENSSSCG00000010273	6.955767516	4.58E-10
ENSSSCG00000034961	6.953836609	5.76E-07
ENSSSCG00000006717	6.906011777	2.10E-104
ENSSSCG00000026339	6.884062881	1.71E-07
ENSSSCG00000047223	6.873257734	1.15E-06
ENSSSCG00000033808	6.857979302	1.24E-06
ENSSSCG00000036669	6.839547555	2.61E-39
ENSSSCG00000012791	6.707539314	1.79E-38
ENSSSCG00000034371	6.623956709	0.007732777
ENSSSCG00000015069	6.600144484	1.70E-05
ENSSSCG00000023684	6.504100089	3.26E-29
ENSSSCG00000009216	6.464373134	1.76E-76
ENSSSCG00000003483	6.460906511	6.39E-32
ENSSSCG00000040651	6.341349733	5.39E-117
ENSSSCG00000037913	6.264271781	1.67E-50
ENSSSCG00000034197	6.07029091	0.00148432
ENSSSCG00000009469	6.052658161	3.20E-58
ENSSSCG00000022258	5.991044994	2.51E-31
ENSSSCG00000026333	5.910398516	7.10E-15
ENSSSCG00000002847	5.901384278	2.00E-51
ENSSSCG00000000194	5.900922237	3.08E-94
ENSSSCG00000039146	5.86884868	1.99E-93
ENSSSCG00000033640	5.857113942	9.65E-07
ENSSSCG00000013337	5.829242434	4.03E-05
ENSSSCG00000004509	5.814435238	7.30E-41
ENSSSCG00000046104	5.668085955	0.011770501
ENSSSCG00000034215	5.646622559	1.17E-28
ENSSSCG00000028871	5.638595667	2.83E-14
ENSSSCG00000028331	5.637014106	2.45E-28
ENSSSCG00000035928	5.632423297	2.47E-10
ENSSSCG00000026043	5.627548143	8.34E-72
ENSSSCG00000030388	5.616253803	2.17E-34
ENSSSCG00000003251	5.611677623	1.19E-12
ENSSSCG00000009182	5.601351852	1.65E-10
ENSSSCG00000002010	5.585604818	0.000864838
ENSSSCG00000046670	5.570570554	0.000306778
ENSSSCG00000015477	5.520297514	5.27E-15

ENSSSCG00000008648	5.510117122	2.54E-54
ENSSSCG00000001576	5.503888531	0.001084591
ENSSSCG00000031143	5.469672604	0.013774861
ENSSSCG00000024347	5.41206967	0.003510606
ENSSSCG00000023041	5.41069986	1.62E-16
ENSSSCG00000046783	5.410291887	9.85E-06
ENSSSCG00000032691	5.402667501	7.78E-06
ENSSSCG00000040986	5.351180435	2.29E-69
ENSSSCG00000047085	5.314452535	0.002033683
ENSSSCG00000022758	5.292919509	5.88E-41
ENSSSCG00000016902	5.271520258	3.47E-59
ENSSSCG00000038326	5.265086988	0.003478806
ENSSSCG00000033251	5.259394784	6.50E-24
ENSSSCG00000046386	5.202164396	1.07E-12
ENSSSCG00000006043	5.20073885	0.004802789
ENSSSCG00000040009	5.194578306	3.40E-08
ENSSSCG00000002792	5.169999508	0.005671346
ENSSSCG00000008088	5.148175326	5.99E-05
ENSSSCG00000040513	5.135793267	6.80E-128
ENSSSCG00000042806	5.12715707	0.005213731
ENSSSCG00000013575	5.125947424	1.17E-13
ENSSSCG00000035443	5.077749695	6.29E-07
ENSSSCG00000005452	5.073415625	1.68E-10
ENSSSCG00000042112	5.031432986	0.010441457
ENSSSCG00000027621	5.020865218	2.33E-57
ENSSSCG00000050493	5.007085418	8.89E-12
ENSSSCG00000043809	4.979472109	8.02E-34
ENSSSCG00000041327	4.977610068	0.003921315
ENSSSCG00000024911	4.952691579	5.97E-23
ENSSSCG00000050635	4.939287849	0.011788157
ENSSSCG00000007716	4.935390129	0.001287892
ENSSSCG00000021610	4.934821864	0.002919451
ENSSSCG00000015340	4.920508857	2.10E-95
ENSSSCG00000012871	4.894351218	0.009969689
ENSSSCG00000000171	4.867741525	2.05E-32
ENSSSCG00000003968	4.864818835	6.09E-06
ENSSSCG00000031037	4.862978039	6.75E-82
ENSSSCG00000034920	4.845070536	0.017249008
ENSSSCG00000004001	4.818901247	0.003918365
ENSSSCG00000039758	4.785807003	9.91E-29
ENSSSCG00000001978	4.781488775	9.28E-50
ENSSSCG00000028671	4.762378695	5.92E-06
ENSSSCG00000009645	4.744090118	0.001367736
ENSSSCG00000038418	4.734704747	0.004643289
ENSSSCG00000042654	4.666432012	0.00294783
ENSSSCG00000010023	4.663588731	0.017562094
ENSSSCG00000034023	4.631844528	1.18E-35
ENSSSCG00000008284	4.624115826	0.003452414
ENSSSCG00000039102	4.617031687	4.44E-62
ENSSSCG00000024867	4.582744815	1.03E-55
ENSSSCG00000014168	4.563002843	1.93E-89
ENSSSCG00000023796	4.55942323	0.00436047
ENSSSCG00000039862	4.541531114	5.03E-51
ENSSSCG00000007596	4.480399474	3.60E-24
ENSSSCG00000035457	4.478357525	9.42E-13
ENSSSCG00000035297	4.457951987	2.40E-31
ENSSSCG00000037524	4.455260133	4.00E-22
ENSSSCG00000034792	4.454676423	0.006465626

ENSSSCG00000000866	4.441767188	7.70E-08
ENSSSCG00000012161	4.430608448	1.02E-43
ENSSSCG00000039553	4.425219953	2.79E-15
ENSSSCG00000044175	4.394912889	0.007099612
ENSSSCG00000032578	4.379264547	7.05E-37
ENSSSCG00000002483	4.363401153	1.40E-06
ENSSSCG00000002009	4.354502043	4.29E-111
ENSSSCG00000042296	4.347938243	0.01392948
ENSSSCG00000051443	4.344102874	0.012474602
ENSSSCG00000011636	4.336266323	0.013750518
ENSSSCG00000036746	4.330189373	1.10E-30
ENSSSCG00000024311	4.327145572	6.81E-66
ENSSSCG00000044986	4.314392493	0.000140026
ENSSSCG00000033114	4.308457025	7.69E-63
ENSSSCG00000016903	4.293833426	6.78E-30
ENSSSCG00000039573	4.289576088	1.21E-20
ENSSSCG00000040575	4.276970655	1.67E-24
ENSSSCG00000043636	4.25901208	0.011612482
ENSSSCG00000007358	4.244104891	0.001901155
ENSSSCG00000006588	4.238184289	5.02E-06
ENSSSCG00000007371	4.226924669	0.011480864
ENSSSCG00000042841	4.225322531	1.06E-06
ENSSSCG00000035379	4.18327041	3.18E-32
ENSSSCG00000023691	4.174918159	0.017927019
ENSSSCG00000008560	4.158165529	0.003066383
ENSSSCG00000050056	4.136120372	0.01679781
ENSSSCG00000041044	4.136109089	0.016178086
ENSSSCG00000049508	4.120863382	0.016263509
ENSSSCG00000029879	4.100019944	6.63E-17
ENSSSCG00000049441	4.087095643	1.70E-07
ENSSSCG00000039158	4.086322692	1.94E-10
ENSSSCG00000015215	4.085447308	2.27E-06
ENSSSCG00000010271	4.078366128	1.58E-60
ENSSSCG00000045124	4.074736994	0.018000225
ENSSSCG00000011596	4.068881653	0.005100668
ENSSSCG00000036157	4.054814259	0.000448122
ENSSSCG00000032857	4.040964894	2.76E-06
ENSSSCG00000007748	4.034605922	2.66E-57
ENSSSCG00000017700	4.031601728	2.68E-30
ENSSSCG00000034570	4.014030124	1.55E-31
ENSSSCG00000000638	3.991612002	1.26E-12
ENSSSCG00000049151	3.926586904	0.00696193
ENSSSCG00000003582	3.912254019	3.47E-08
ENSSSCG00000003259	3.905250512	1.29E-23
ENSSSCG00000051633	3.898302164	7.68E-19
ENSSSCG00000008228	3.880086787	3.46E-24
ENSSSCG00000021259	3.865233896	9.21E-05
ENSSSCG00000031374	3.857715686	7.88E-16
ENSSSCG00000004826	3.837299818	2.27E-54
ENSSSCG00000001786	3.830933354	0.000232827
ENSSSCG00000012077	3.825254025	3.86E-110
ENSSSCG00000003306	3.818798706	0.014461384
ENSSSCG00000033675	3.805466515	7.51E-08
ENSSSCG00000034723	3.803385385	5.91E-37
ENSSSCG00000038719	3.789723963	8.03E-40
ENSSSCG00000043070	3.787716507	9.73E-14
ENSSSCG00000024568	3.776434114	6.02E-19
ENSSSCG00000005948	3.774304546	4.35E-10

ENSSSCG00000000728	3.754796031	4.34E-12
ENSSSCG00000006590	3.747096676	7.68E-05
ENSSSCG00000041810	3.746541868	4.90E-09
ENSSSCG00000039966	3.738182627	1.40E-06
ENSSSCG00000022649	3.733227381	1.52E-12
ENSSSCG00000004597	3.710886268	2.69E-07
ENSSSCG00000017392	3.702276377	1.31E-17
ENSSSCG00000051050	3.698488153	5.35E-05
ENSSSCG00000031979	3.677967239	0.001386018
ENSSSCG00000010452	3.646335357	4.14E-43
ENSSSCG00000006966	3.6445685	0.002580404
ENSSSCG00000003491	3.628227429	6.17E-23
ENSSSCG00000026414	3.62455039	2.12E-16
ENSSSCG00000008823	3.623673258	4.14E-10
ENSSSCG00000006808	3.618568675	7.03E-13
ENSSSCG00000050389	3.617318235	0.006534945
ENSSSCG00000013310	3.612753756	0.010059044
ENSSSCG00000023298	3.612519816	8.07E-25
ENSSSCG00000009532	3.60503294	1.73E-38
ENSSSCG00000041921	3.593452781	0.001041105
ENSSSCG00000030996	3.585541388	1.51E-81
ENSSSCG00000032477	3.583875143	1.17E-18
ENSSSCG00000032474	3.580050158	1.28E-26
ENSSSCG00000032436	3.566487814	5.30E-24
ENSSSCG00000050951	3.556223153	0.007158702
ENSSSCG00000011965	3.553106309	9.14E-05
ENSSSCG00000009945	3.549102379	4.85E-62
ENSSSCG00000001909	3.548810825	6.06E-09
ENSSSCG00000036383	3.543000383	6.94E-41
ENSSSCG00000017723	3.535658659	3.70E-23
ENSSSCG00000011195	3.508828132	6.64E-15
ENSSSCG00000010536	3.477949416	1.59E-10
ENSSSCG00000038912	3.477732912	1.44E-40
ENSSSCG00000010507	3.474489016	9.66E-05
ENSSSCG00000006830	3.465494295	1.24E-28
ENSSSCG00000037431	3.461409816	3.44E-26
ENSSSCG00000022726	3.458368482	2.33E-13
ENSSSCG00000040648	3.456631316	3.19E-15
ENSSSCG00000013579	3.455436079	1.77E-21
ENSSSCG00000032343	3.453570272	2.09E-56
ENSSSCG00000004369	3.443436126	1.32E-55
ENSSSCG00000040025	3.430016292	0.007370486
ENSSSCG00000016449	3.425009149	7.22E-36
ENSSSCG00000016175	3.402806908	2.85E-30
ENSSSCG00000001137	3.395166427	2.79E-11
ENSSSCG00000037709	3.394406047	6.70E-07
ENSSSCG00000011447	3.379354538	2.09E-23
ENSSSCG00000036130	3.378091929	0.001342417
ENSSSCG00000000774	3.375694046	6.67E-54
ENSSSCG00000015742	3.370730593	5.18E-09
ENSSSCG00000013513	3.370472607	7.83E-19
ENSSSCG00000037572	3.370192327	1.04E-31
ENSSSCG00000032525	3.36295974	5.36E-06
ENSSSCG00000028858	3.362770991	0.006639752
ENSSSCG00000004912	3.36080712	1.29E-23
ENSSSCG00000007508	3.359339618	3.06E-44
ENSSSCG00000006164	3.352949979	0.001777216
ENSSSCG00000004908	3.346354525	3.89E-64

ENSSSCG00000023178	3.333628265	1.03E-27
ENSSSCG00000046117	3.323129496	0.001405834
ENSSSCG00000007007	3.320224351	1.05E-26
ENSSSCG00000029756	3.314872767	0.001028541
ENSSSCG00000024300	3.311735807	2.27E-16
ENSSSCG00000005995	3.306848359	4.79E-19
ENSSSCG00000001589	3.298122979	2.13E-10
ENSSSCG00000025795	3.2805211	7.00E-19
ENSSSCG00000050665	3.276547631	0.010673753
ENSSSCG00000017258	3.275294134	1.32E-12
ENSSSCG00000004728	3.265600002	7.46E-37
ENSSSCG00000035863	3.260863158	4.27E-39
ENSSSCG00000010451	3.260417604	2.77E-30
ENSSSCG00000044155	3.256389493	5.61E-11
ENSSSCG00000032652	3.254270889	8.87E-12
ENSSSCG00000040815	3.253394801	1.12E-80
ENSSSCG00000044078	3.246512634	3.50E-17
ENSSSCG00000011870	3.244234975	6.18E-42
ENSSSCG00000043043	3.243398369	6.13E-12
ENSSSCG00000032060	3.242181494	2.54E-13
ENSSSCG00000049851	3.240520151	0.008154404
ENSSSCG00000007130	3.234469504	0.002879467
ENSSSCG00000023557	3.219812378	4.05E-27
ENSSSCG00000004889	3.199074057	1.02E-22
ENSSSCG00000022404	3.19901546	4.37E-27
ENSSSCG00000012967	3.191117461	9.17E-05
ENSSSCG00000040162	3.187101378	6.03E-24
ENSSSCG00000031741	3.177057013	7.69E-66
ENSSSCG00000027709	3.166196045	0.004229355
ENSSSCG00000012171	3.165127228	6.79E-29
ENSSSCG00000000982	3.16186413	2.64E-09
ENSSSCG00000032053	3.158135137	3.95E-25
ENSSSCG00000005601	3.152529728	1.29E-31
ENSSSCG00000048083	3.150017463	0.015399887
ENSSSCG00000028203	3.149567631	4.06E-05
ENSSSCG00000021241	3.14085578	1.49E-20
ENSSSCG00000030548	3.140807536	1.04E-52
ENSSSCG00000016092	3.137399144	6.85E-32
ENSSSCG00000016504	3.135554947	2.25E-19
ENSSSCG00000010084	3.133927345	2.86E-20
ENSSSCG00000006831	3.13370818	3.08E-42
ENSSSCG00000015496	3.1184554	1.35E-27
ENSSSCG00000015590	3.112181805	1.49E-24
ENSSSCG00000020906	3.111535389	2.12E-32
ENSSSCG00000039214	3.110427585	2.24E-10
ENSSSCG00000030575	3.10471028	3.83E-07
ENSSSCG00000040710	3.102386479	6.31E-35
ENSSSCG00000029239	3.100099554	1.05E-15
ENSSSCG00000047929	3.098503153	4.58E-20
ENSSSCG00000006294	3.094754438	2.78E-11
ENSSSCG00000015453	3.089634195	7.51E-55
ENSSSCG00000040779	3.086306034	4.32E-19
ENSSSCG00000002452	3.085015474	2.58E-23
ENSSSCG00000039508	3.076516758	0.011875643
ENSSSCG00000037950	3.073421784	2.76E-12
ENSSSCG00000008976	3.071557843	7.25E-12
ENSSSCG00000034898	3.060892854	1.01E-21
ENSSSCG00000024437	3.055999273	9.03E-24

ENSSSCG00000011621	3.053965766	6.81E-70
ENSSSCG00000012076	3.051948032	1.66E-51
ENSSSCG00000038429	3.047911741	4.23E-08
ENSSSCG00000002414	3.046217614	2.78E-29
ENSSSCG00000030408	3.041308258	7.03E-34
ENSSSCG00000011560	3.037917295	1.46E-26
ENSSSCG00000038305	3.031431855	1.84E-08
ENSSSCG00000006729	3.008612701	5.16E-21
ENSSSCG00000002849	3.002913065	2.05E-29
ENSSSCG00000001835	3.001234494	5.13E-12
ENSSSCG00000006835	2.999330731	6.28E-54
ENSSSCG00000021206	2.995321438	7.30E-22
ENSSSCG00000033222	2.988138455	7.61E-08
ENSSSCG00000033703	2.986773735	7.10E-19
ENSSSCG00000034260	2.98675169	3.05E-08
ENSSSCG00000044354	2.983965056	0.001246042
ENSSSCG00000010261	2.979508366	7.72E-26
ENSSSCG00000033457	2.975829203	3.55E-07
ENSSSCG00000020666	2.971675066	8.99E-07
ENSSSCG00000004870	2.970363985	1.68E-27
ENSSSCG00000029326	2.967413345	8.64E-33
ENSSSCG00000038489	2.966197163	6.87E-40
ENSSSCG00000037358	2.964578152	1.43E-22
ENSSSCG00000000849	2.962254682	7.39E-42
ENSSSCG00000017473	2.96200978	8.81E-44
ENSSSCG00000033507	2.953705961	0.001564174
ENSSSCG00000004666	2.949360188	8.83E-26
ENSSSCG00000034131	2.948961271	9.03E-27
ENSSSCG00000034555	2.948880151	1.58E-16
ENSSSCG00000008601	2.946416188	1.04E-17
ENSSSCG00000017176	2.945645752	0.005561359
ENSSSCG00000003113	2.937331247	6.02E-10
ENSSSCG00000028696	2.934840998	2.22E-34
ENSSSCG00000039854	2.934839827	3.70E-35
ENSSSCG00000040990	2.92159056	2.42E-07
ENSSSCG00000029438	2.920727553	2.48E-51
ENSSSCG00000036438	2.908329279	9.88E-21
ENSSSCG00000006561	2.907391541	1.41E-42
ENSSSCG00000024344	2.904751797	3.50E-23
ENSSSCG00000008647	2.90345215	1.98E-67
ENSSSCG00000002990	2.901302762	5.75E-06
ENSSSCG00000035176	2.900382045	6.94E-12
ENSSSCG00000003046	2.899633394	1.18E-15
ENSSSCG00000006360	2.895365517	1.06E-43
ENSSSCG00000032996	2.889325484	2.10E-44
ENSSSCG00000008769	2.888609768	9.63E-07
ENSSSCG00000033750	2.88837087	0.005834199
ENSSSCG00000009100	2.886935971	2.72E-09
ENSSSCG00000013632	2.886104001	1.47E-13
ENSSSCG00000009145	2.885227115	5.80E-20
ENSSSCG00000017235	2.880322387	1.77E-07
ENSSSCG00000022786	2.875367622	4.30E-36
ENSSSCG00000008978	2.87507667	1.36E-07
ENSSSCG00000016653	2.872574561	3.35E-34
ENSSSCG00000032367	2.86985733	1.18E-15
ENSSSCG00000026067	2.862912947	1.04E-05
ENSSSCG00000009964	2.858429742	2.28E-08
ENSSSCG00000017103	2.855437284	1.97E-18

ENSSSCG00000027629	2.854883008	1.09E-19
ENSSSCG00000011322	2.85474079	4.17E-18
ENSSSCG00000010454	2.853732474	1.63E-29
ENSSSCG00000017614	2.852161833	1.12E-30
ENSSSCG00000010312	2.84776244	1.43E-32
ENSSSCG00000036132	2.836904816	6.76E-09
ENSSSCG00000026724	2.834678956	7.16E-27
ENSSSCG00000006550	2.832370037	2.44E-08
ENSSSCG00000000265	2.829289334	4.28E-20
ENSSSCG00000032914	2.828486121	4.08E-15
ENSSSCG00000032644	2.821659335	2.88E-17
ENSSSCG00000044489	2.820682252	1.06E-08
ENSSSCG00000002855	2.820252835	3.20E-44
ENSSSCG00000034802	2.818595494	4.95E-12
ENSSSCG00000026082	2.815600817	2.92E-42
ENSSSCG00000017416	2.815595928	2.14E-28
ENSSSCG00000022402	2.813839444	6.24E-30
ENSSSCG00000024841	2.812772147	1.22E-27
ENSSSCG00000010912	2.804380544	1.01E-05
ENSSSCG00000005688	2.804098434	6.54E-11
ENSSSCG00000037491	2.803974899	2.38E-21
ENSSSCG00000028019	2.803820432	3.68E-65
ENSSSCG00000015016	2.801022807	5.78E-18
ENSSSCG00000007933	2.800376503	0.002340003
ENSSSCG00000008207	2.798668464	9.22E-40
ENSSSCG00000013880	2.79805321	1.11E-06
ENSSSCG00000009720	2.787752937	2.80E-27
ENSSSCG00000040317	2.78620415	4.20E-37
ENSSSCG00000001566	2.785526554	0.003985395
ENSSSCG00000034506	2.784939538	1.28E-10
ENSSSCG00000026852	2.783124673	3.08E-21
ENSSSCG00000004164	2.772242613	2.61E-31
ENSSSCG00000008875	2.770938329	0.000164804
ENSSSCG00000039713	2.769175798	4.65E-12
ENSSSCG00000016677	2.767511341	1.18E-49
ENSSSCG00000002852	2.763460852	3.96E-19
ENSSSCG00000004125	2.75923043	8.31E-41
ENSSSCG00000001892	2.758140775	1.02E-33
ENSSSCG00000010461	2.757615875	0.000363048
ENSSSCG00000010992	2.747136023	0.000700218
ENSSSCG00000014565	2.746061778	1.68E-15
ENSSSCG00000034213	2.745075823	0.009150394
ENSSSCG00000002033	2.7397718	0.001931214
ENSSSCG00000012006	2.738195505	3.04E-21
ENSSSCG00000013787	2.729833759	2.81E-05
ENSSSCG00000011797	2.725357152	5.13E-30
ENSSSCG00000022301	2.721052625	3.45E-17
ENSSSCG00000039148	2.716546766	3.55E-30
ENSSSCG00000024233	2.715036228	3.84E-16
ENSSSCG00000050689	2.713882633	0.001983072
ENSSSCG00000037775	2.713145372	1.31E-17
ENSSSCG00000017772	2.712851763	1.92E-30
ENSSSCG00000001636	2.709966716	0.014834112
ENSSSCG00000047515	2.709874013	1.02E-12
ENSSSCG00000008937	2.706343234	3.11E-19
ENSSSCG00000004419	2.702205803	0.000494656
ENSSSCG00000004554	2.702144446	6.94E-16
ENSSSCG00000045298	2.701818182	0.004043898

ENSSSCG00000000289	2.700573923	6.29E-13
ENSSSCG00000016817	2.699695674	1.97E-44
ENSSSCG00000017061	2.695519389	0.005850865
ENSSSCG00000017098	2.688553234	0.000222789
ENSSSCG00000000981	2.687125429	2.13E-16
ENSSSCG00000013772	2.682010985	7.57E-22
ENSSSCG00000042208	2.678011218	2.10E-21
ENSSSCG00000037771	2.677833601	1.25E-06
ENSSSCG00000027455	2.676683965	7.85E-27
ENSSSCG00000006720	2.674553606	1.39E-05
ENSSSCG00000032397	2.673597768	7.63E-34
ENSSSCG00000011714	2.672989794	3.92E-15
ENSSSCG00000040663	2.671131341	1.43E-37
ENSSSCG00000044762	2.669883268	0.01148125
ENSSSCG00000007160	2.668050925	1.38E-16
ENSSSCG00000034738	2.665962974	1.28E-10
ENSSSCG00000015492	2.665361006	0.000282446
ENSSSCG00000042147	2.665313065	0.011348045
ENSSSCG00000003201	2.665174851	4.67E-30
ENSSSCG00000008938	2.664409969	0.000224321
ENSSSCG00000029005	2.662972406	4.96E-34
ENSSSCG00000011627	2.657883801	0.005366577
ENSSSCG00000035746	2.654760762	3.20E-18
ENSSSCG00000000858	2.652212705	5.08E-06
ENSSSCG00000048661	2.649807003	0.00049348
ENSSSCG00000008261	2.645683442	7.80E-26
ENSSSCG00000034129	2.642771839	6.82E-10
ENSSSCG00000000405	2.636039787	4.59E-23
ENSSSCG00000000688	2.633570456	8.54E-16
ENSSSCG00000022483	2.629876645	4.25E-05
ENSSSCG00000008703	2.629610463	4.21E-08
ENSSSCG00000040486	2.628966761	1.61E-14
ENSSSCG00000012066	2.628671143	0.001409319
ENSSSCG00000037347	2.627672235	2.82E-11
ENSSSCG00000011680	2.627455788	3.18E-06
ENSSSCG00000050451	2.626962953	1.39E-11
ENSSSCG00000010471	2.62652169	5.75E-26
ENSSSCG00000023172	2.625464868	0.000161358
ENSSSCG00000015839	2.624983136	4.28E-11
ENSSSCG00000028377	2.624336985	0.000438413
ENSSSCG00000000699	2.623548547	6.24E-10
ENSSSCG00000037074	2.621284534	1.60E-07
ENSSSCG00000025423	2.613175477	2.62E-25
ENSSSCG00000025565	2.612983776	5.98E-26
ENSSSCG00000036286	2.611424179	6.67E-55
ENSSSCG00000000950	2.610677294	5.50E-24
ENSSSCG00000022292	2.60910126	1.08E-10
ENSSSCG00000012346	2.606760219	4.92E-05
ENSSSCG00000011798	2.606410308	7.94E-28
ENSSSCG00000024119	2.603564352	4.26E-29
ENSSSCG00000032241	2.6027614	2.66E-15
ENSSSCG00000027249	2.602755018	0.008454537
ENSSSCG00000038360	2.599761419	0.000753535
ENSSSCG00000017022	2.597645027	1.26E-27
ENSSSCG00000003229	2.596829531	1.94E-10
ENSSSCG00000031860	2.596538268	1.42E-09
ENSSSCG00000026455	2.593966412	7.64E-09
ENSSSCG00000015241	2.592313957	0.001595719

ENSSSCG00000036568	2.59101206	6.99E-14
ENSSSCG00000032135	2.587997807	5.40E-17
ENSSSCG00000025965	2.587903827	3.94E-21
ENSSSCG00000015786	2.587174022	7.09E-26
ENSSSCG00000035564	2.586900455	8.08E-33
ENSSSCG00000040134	2.586756791	8.15E-12
ENSSSCG00000040709	2.586201754	4.05E-29
ENSSSCG00000015566	2.585766303	5.03E-19
ENSSSCG00000003763	2.585490002	3.14E-33
ENSSSCG00000015106	2.58429201	1.23E-34
ENSSSCG00000048903	2.583275954	0.000315336
ENSSSCG00000047799	2.582414263	0.00325268
ENSSSCG00000003044	2.578291738	0.000957836
ENSSSCG00000044512	2.57708759	3.13E-08
ENSSSCG00000013296	2.575185766	1.41E-11
ENSSSCG00000016590	2.572936319	4.97E-33
ENSSSCG00000046958	2.567308114	2.80E-12
ENSSSCG00000011855	2.565547302	0.001224432
ENSSSCG00000039216	2.563284683	8.39E-16
ENSSSCG00000001862	2.563271399	1.50E-30
ENSSSCG00000013842	2.557037593	2.05E-06
ENSSSCG00000038307	2.556734143	6.88E-14
ENSSSCG00000029592	2.556716572	1.67E-06
ENSSSCG00000039419	2.555006636	1.25E-20
ENSSSCG00000031788	2.552015835	4.95E-19
ENSSSCG00000003069	2.551426265	0.00383069
ENSSSCG00000001011	2.551008087	2.32E-26
ENSSSCG00000000685	2.548124662	1.98E-15
ENSSSCG00000017637	2.544738418	3.98E-05
ENSSSCG00000009360	2.536695464	5.88E-17
ENSSSCG00000013911	2.534710133	9.09E-08
ENSSSCG00000035634	2.531179375	1.49E-25
ENSSSCG00000009799	2.529168789	1.30E-23
ENSSSCG00000035532	2.518711434	1.23E-16
ENSSSCG00000013107	2.513143768	1.40E-38
ENSSSCG00000012307	2.511840143	5.79E-30
ENSSSCG00000035344	2.51069825	2.36E-11
ENSSSCG00000005212	2.509154696	2.38E-09
ENSSSCG00000017420	2.503387191	1.17E-27
ENSSSCG00000039107	2.495636051	2.03E-19
ENSSSCG00000013730	2.495138953	3.36E-05
ENSSSCG00000035914	2.495071817	8.20E-13
ENSSSCG00000017705	2.492478387	1.20E-12
ENSSSCG00000000158	2.490572432	4.65E-34
ENSSSCG00000006865	2.488140746	2.95E-25
ENSSSCG00000001773	2.487903788	1.03E-10
ENSSSCG00000012277	2.485089584	4.95E-13
ENSSSCG00000009035	2.48076451	1.71E-16
ENSSSCG00000005628	2.476158724	2.74E-21
ENSSSCG00000009334	2.473957694	2.11E-15
ENSSSCG00000003272	2.471769838	6.44E-07
ENSSSCG00000002723	2.471222187	2.58E-44
ENSSSCG00000004503	2.468034374	0.000345056
ENSSSCG00000040277	2.467131698	5.23E-33
ENSSSCG00000020970	2.467068008	6.03E-29
ENSSSCG00000013909	2.465211855	2.79E-12
ENSSSCG00000005344	2.464606064	2.31E-25
ENSSSCG00000008316	2.464404781	1.95E-14

ENSSSCG00000036723	2.463762046	2.53E-21
ENSSSCG00000016744	2.463742916	9.64E-24
ENSSSCG00000025981	2.463165689	1.01E-18
ENSSSCG00000036924	2.461966667	2.53E-13
ENSSSCG00000012377	2.457840649	4.66E-22
ENSSSCG00000009373	2.457703346	1.45E-07
ENSSSCG00000025010	2.455864201	6.56E-21
ENSSSCG00000027121	2.454192607	1.34E-35
ENSSSCG00000015337	2.450689993	7.19E-07
ENSSSCG00000003697	2.449577365	4.59E-16
ENSSSCG00000014136	2.449027749	2.04E-21
ENSSSCG00000051622	2.44890517	0.012443178
ENSSSCG00000034913	2.44505496	5.58E-18
ENSSSCG00000001565	2.444661673	4.73E-13
ENSSSCG00000035565	2.444416597	1.43E-32
ENSSSCG00000009526	2.44272011	3.16E-07
ENSSSCG00000010332	2.442128893	6.92E-15
ENSSSCG00000029414	2.441212475	3.52E-13
ENSSSCG00000032234	2.441095413	5.48E-23
ENSSSCG00000023379	2.437187397	1.56E-20
ENSSSCG00000009330	2.436978885	1.31E-08
ENSSSCG00000008352	2.435180904	2.96E-25
ENSSSCG00000039635	2.433811579	1.54E-15
ENSSSCG00000007717	2.433040479	2.35E-08
ENSSSCG00000039582	2.432705794	3.67E-13
ENSSSCG00000005666	2.432674882	3.94E-11
ENSSSCG00000039300	2.432521961	6.10E-10
ENSSSCG00000001560	2.432065658	0.001761161
ENSSSCG00000010315	2.431692022	1.01E-28
ENSSSCG00000009671	2.429924887	7.68E-15
ENSSSCG00000000847	2.427303861	2.96E-16
ENSSSCG00000003006	2.426618767	2.41E-08
ENSSSCG00000013082	2.425948526	1.49E-13
ENSSSCG00000022679	2.42359108	4.62E-20
ENSSSCG00000001656	2.423297788	3.16E-10
ENSSSCG00000003105	2.42270943	9.48E-46
ENSSSCG00000008633	2.42079605	2.42E-25
ENSSSCG00000028711	2.419014708	6.29E-12
ENSSSCG00000010691	2.418884981	5.82E-06
ENSSSCG00000005056	2.418829788	7.33E-22
ENSSSCG00000004101	2.417798632	1.06E-13
ENSSSCG00000020885	2.415752989	1.87E-28
ENSSSCG00000039538	2.415177881	2.58E-15
ENSSSCG00000014898	2.414661255	6.53E-15
ENSSSCG00000023604	2.414597941	9.36E-26
ENSSSCG00000029538	2.411365488	5.60E-35
ENSSSCG00000022045	2.411336945	0.00592679
ENSSSCG00000010470	2.409601848	7.81E-19
ENSSSCG00000003861	2.408527475	4.34E-30
ENSSSCG00000012440	2.408433038	3.11E-25
ENSSSCG00000013318	2.408151085	5.33E-30
ENSSSCG00000016183	2.406811144	0.007998439
ENSSSCG00000009938	2.401528645	5.03E-20
ENSSSCG00000022351	2.401261867	6.17E-09
ENSSSCG00000009132	2.398730672	1.02E-09
ENSSSCG00000011207	2.398541428	1.68E-18
ENSSSCG00000010214	2.398144657	1.44E-12
ENSSSCG00000007745	2.395182791	1.88E-11

ENSSSCG00000013401	2.393768133	1.48E-13
ENSSSCG00000000783	2.39161975	0.008704411
ENSSSCG00000032777	2.391467026	4.91E-21
ENSSSCG00000000719	2.391162968	6.19E-13
ENSSSCG00000032261	2.389692408	8.62E-19
ENSSSCG00000005661	2.389554252	9.63E-07
ENSSSCG00000009772	2.389241675	2.33E-23
ENSSSCG00000007436	2.388223391	2.08E-06
ENSSSCG00000009112	2.384641569	3.07E-14
ENSSSCG00000036436	2.383569971	3.29E-05
ENSSSCG00000031855	2.383334676	7.04E-12
ENSSSCG00000036224	2.382797266	2.36E-14
ENSSSCG00000028879	2.382667722	3.11E-37
ENSSSCG00000028282	2.379952819	1.34E-32
ENSSSCG00000022787	2.378211373	1.42E-11
ENSSSCG00000030300	2.377463887	9.46E-10
ENSSSCG00000005600	2.376956712	2.75E-08
ENSSSCG00000045883	2.376863863	2.46E-05
ENSSSCG00000032063	2.376611605	0.001051418
ENSSSCG00000013114	2.376416047	3.11E-18
ENSSSCG00000016186	2.375573442	2.68E-19
ENSSSCG00000014048	2.375189333	1.17E-23
ENSSSCG00000003231	2.37321473	2.36E-15
ENSSSCG00000024145	2.373167039	6.07E-26
ENSSSCG00000037642	2.37199135	7.94E-16
ENSSSCG00000006560	2.369789418	7.78E-09
ENSSSCG00000035684	2.368502525	5.40E-25
ENSSSCG00000014304	2.367709639	1.26E-31
ENSSSCG00000021815	2.364778621	1.11E-11
ENSSSCG00000021145	2.363533483	1.32E-13
ENSSSCG00000013747	2.362962299	6.15E-32
ENSSSCG00000041543	2.362273852	0.008993984
ENSSSCG00000051107	2.361289537	0.005987137
ENSSSCG00000015299	2.360678591	2.49E-06
ENSSSCG00000017810	2.357654342	0.008174208
ENSSSCG00000011000	2.357114026	7.13E-19
ENSSSCG00000008089	2.356106092	2.84E-26
ENSSSCG00000010644	2.355907818	2.52E-17
ENSSSCG00000033443	2.355426721	4.88E-15
ENSSSCG00000002793	2.352454272	0.000122064
ENSSSCG00000034119	2.351206259	1.02E-05
ENSSSCG00000008841	2.35066171	2.38E-17
ENSSSCG00000050530	2.347979935	0.000179369
ENSSSCG00000006298	2.347148465	5.91E-13
ENSSSCG00000038895	2.345855632	1.01E-12
ENSSSCG00000016272	2.343926578	3.03E-28
ENSSSCG00000008077	2.343743232	3.46E-12
ENSSSCG00000033770	2.342352798	0.000770035
ENSSSCG00000047090	2.334722501	4.10E-19
ENSSSCG00000022343	2.331909808	1.08E-25
ENSSSCG00000005440	2.330451506	1.01E-18
ENSSSCG00000028433	2.329585942	0.000101656
ENSSSCG00000007351	2.32946891	9.93E-12
ENSSSCG00000027130	2.327251066	1.35E-07
ENSSSCG00000000579	2.325846323	9.83E-14
ENSSSCG00000040012	2.325529949	1.18E-11
ENSSSCG00000039267	2.325511595	2.79E-11
ENSSSCG00000034758	2.322198702	2.22E-10

ENSSSCG00000008125	2.32133338	2.51E-32
ENSSSCG00000036257	2.320840926	2.08E-20
ENSSSCG00000039474	2.320664842	3.52E-06
ENSSSCG00000004092	2.320355874	7.36E-16
ENSSSCG00000006400	2.319817706	0.000821147
ENSSSCG00000000184	2.318016481	7.35E-09
ENSSSCG00000012623	2.317178365	1.24E-16
ENSSSCG00000033149	2.312092649	1.46E-18
ENSSSCG00000035434	2.312045467	4.15E-08
ENSSSCG00000010548	2.31189148	0.00020695
ENSSSCG00000010404	2.31178924	7.65E-10
ENSSSCG00000012007	2.310732201	8.70E-16
ENSSSCG00000049674	2.310612755	0.009830525
ENSSSCG00000000839	2.307587962	7.57E-17
ENSSSCG00000032632	2.304998786	0.001205215
ENSSSCG00000005710	2.304291807	2.53E-06
ENSSSCG00000033982	2.303215107	1.91E-08
ENSSSCG00000021597	2.30300753	5.59E-07
ENSSSCG00000037637	2.301956908	8.88E-14
ENSSSCG00000036178	2.299521435	1.22E-13
ENSSSCG00000008317	2.298687978	6.36E-16
ENSSSCG00000024990	2.298201473	3.86E-22
ENSSSCG00000017255	2.297398216	7.12E-35
ENSSSCG00000016581	2.295564868	1.31E-29
ENSSSCG00000001955	2.294865165	1.14E-22
ENSSSCG00000007845	2.293519223	1.08E-19
ENSSSCG00000044930	2.29316721	2.80E-17
ENSSSCG00000011158	2.292454133	5.51E-28
ENSSSCG00000034229	2.291510847	2.95E-12
ENSSSCG00000036883	2.291076889	4.69E-11
ENSSSCG00000014369	2.291032419	5.44E-09
ENSSSCG00000033493	2.290196164	0.001111972
ENSSSCG00000008240	2.289390583	1.32E-06
ENSSSCG00000029509	2.287724253	1.43E-16
ENSSSCG00000016041	2.287162908	8.55E-14
ENSSSCG00000006191	2.285791327	0.007296664
ENSSSCG00000022797	2.285463083	3.95E-19
ENSSSCG00000008068	2.28461392	6.89E-26
ENSSSCG00000038423	2.281853473	3.38E-16
ENSSSCG00000040401	2.28176102	0.000233829
ENSSSCG00000040845	2.281758854	2.14E-08
ENSSSCG00000030567	2.280380992	1.48E-12
ENSSSCG00000026038	2.279831777	1.33E-12
ENSSSCG00000022295	2.279317527	2.07E-06
ENSSSCG00000007808	2.279078515	2.11E-11
ENSSSCG00000024313	2.27654584	5.97E-09
ENSSSCG00000005650	2.276542836	7.57E-22
ENSSSCG00000016585	2.275830065	7.73E-28
ENSSSCG00000009746	2.275180387	3.99E-22
ENSSSCG00000013094	2.273476766	2.13E-29
ENSSSCG00000021587	2.272600373	2.16E-23
ENSSSCG00000031117	2.271394101	9.18E-11
ENSSSCG00000007602	2.27103352	1.08E-08
ENSSSCG00000012327	2.270629038	4.28E-07
ENSSSCG00000034137	2.269485691	7.55E-19
ENSSSCG00000001561	2.266976058	1.16E-37
ENSSSCG00000048806	2.266259812	0.00051433
ENSSSCG00000038607	2.266118955	0.002724661

ENSSSCG00000007423	2.265032218	1.97E-17
ENSSSCG00000013746	2.255947062	8.99E-30
ENSSSCG00000016441	2.254099115	2.91E-32
ENSSSCG00000018023	2.244813324	2.20E-06
ENSSSCG00000029030	2.244642423	8.83E-16
ENSSSCG00000045225	2.244537626	7.44E-11
ENSSSCG00000041738	2.243774086	1.73E-19
ENSSSCG00000023482	2.243201174	4.79E-21
ENSSSCG00000003170	2.241405314	0.000113821
ENSSSCG00000035960	2.239537796	3.37E-08
ENSSSCG00000006084	2.237336906	7.04E-27
ENSSSCG00000005629	2.237185793	6.42E-10
ENSSSCG00000022643	2.23717611	0.000973292
ENSSSCG00000017668	2.236311189	5.54E-16
ENSSSCG00000009164	2.235862104	6.65E-10
ENSSSCG00000035051	2.235256653	5.31E-16
ENSSSCG00000001950	2.234059047	1.22E-12
ENSSSCG00000023706	2.233889096	0.014254662
ENSSSCG00000011620	2.231306515	2.97E-29
ENSSSCG00000011313	2.231185513	9.08E-25
ENSSSCG00000003788	2.230669786	0.000272424
ENSSSCG00000022925	2.22830204	2.97E-21
ENSSSCG00000017169	2.225018587	0.000105616
ENSSSCG00000013313	2.223070114	0.00119846
ENSSSCG00000039896	2.221743275	7.09E-10
ENSSSCG00000002709	2.221027638	2.69E-13
ENSSSCG00000009959	2.220707045	2.33E-18
ENSSSCG00000033844	2.21991246	1.05E-27
ENSSSCG00000033146	2.219443815	2.21E-20
ENSSSCG00000009963	2.219338173	2.84E-19
ENSSSCG00000003505	2.216986863	5.60E-24
ENSSSCG00000018051	2.216462804	8.92E-15
ENSSSCG00000040452	2.215370458	1.68E-13
ENSSSCG00000009670	2.21476502	5.21E-12
ENSSSCG00000037077	2.212144997	1.06E-23
ENSSSCG00000004806	2.205605792	1.58E-20
ENSSSCG00000014329	2.202678196	5.59E-15
ENSSSCG00000001573	2.201661412	3.50E-25
ENSSSCG00000013258	2.200843871	4.79E-07
ENSSSCG00000003852	2.200458809	0.002150589
ENSSSCG00000015973	2.19977863	7.42E-11
ENSSSCG00000037153	2.199278543	9.43E-05
ENSSSCG00000012491	2.197605016	2.25E-19
ENSSSCG00000033453	2.196986998	5.52E-09
ENSSSCG00000000290	2.195948963	6.15E-17
ENSSSCG00000017493	2.194665845	2.98E-10
ENSSSCG00000033015	2.194487412	9.66E-12
ENSSSCG00000011576	2.194005881	0.000464331
ENSSSCG00000017552	2.193526857	0.000402638
ENSSSCG00000011398	2.192424434	6.29E-12
ENSSSCG00000008796	2.192218548	2.65E-12
ENSSSCG00000046269	2.191416482	0.00327189
ENSSSCG00000024428	2.191275917	1.86E-05
ENSSSCG00000021712	2.185848202	2.64E-28
ENSSSCG00000031990	2.185810349	2.16E-15
ENSSSCG00000035875	2.185365001	2.47E-27
ENSSSCG00000014264	2.184613085	3.83E-09
ENSSSCG00000017754	2.183524845	2.40E-23

ENSSSCG00000016990	2.183408726	2.72E-10
ENSSSCG00000007131	2.18248734	2.73E-10
ENSSSCG00000009889	2.181396378	2.37E-13
ENSSSCG00000004244	2.180136041	5.60E-20
ENSSSCG00000008289	2.1779323	2.14E-19
ENSSSCG00000017396	2.177369159	1.07E-13
ENSSSCG00000023539	2.176780371	8.33E-14
ENSSSCG00000010995	2.175933585	3.67E-22
ENSSSCG00000029668	2.175265338	3.69E-25
ENSSSCG00000004624	2.173567556	1.87E-15
ENSSSCG00000017316	2.171931786	1.26E-24
ENSSSCG00000015214	2.169394139	3.34E-33
ENSSSCG00000017089	2.168208287	1.78E-24
ENSSSCG00000012730	2.167805131	2.02E-09
ENSSSCG00000035170	2.165557568	1.01E-06
ENSSSCG00000015769	2.165211574	5.81E-09
ENSSSCG00000012852	2.158622831	0.007204421
ENSSSCG00000008562	2.156269616	4.86E-17
ENSSSCG00000010435	2.15577125	4.36E-16
ENSSSCG00000012658	2.155242066	0.002215825
ENSSSCG00000017471	2.154168429	7.52E-26
ENSSSCG00000021118	2.153223448	5.80E-20
ENSSSCG00000005040	2.151267766	7.39E-13
ENSSSCG00000033246	2.150807392	6.10E-08
ENSSSCG00000016877	2.148646035	1.09E-25
ENSSSCG00000038500	2.147385767	4.35E-19
ENSSSCG00000005134	2.147051512	3.18E-07
ENSSSCG00000035195	2.146111749	6.52E-11
ENSSSCG00000011065	2.145862377	4.87E-18
ENSSSCG00000045890	2.145701439	2.68E-11
ENSSSCG00000014338	2.145119868	4.45E-24
ENSSSCG00000022940	2.14479264	1.01E-13
ENSSSCG00000013366	2.144742935	1.17E-18
ENSSSCG00000027806	2.144288187	5.07E-06
ENSSSCG00000011612	2.144230474	4.41E-27
ENSSSCG00000038351	2.144173081	4.39E-08
ENSSSCG00000042196	2.142868974	6.09E-06
ENSSSCG00000005403	2.139564111	4.32E-16
ENSSSCG00000016658	2.13896392	4.26E-14
ENSSSCG00000007493	2.137742051	9.11E-30
ENSSSCG00000022312	2.137131212	1.54E-14
ENSSSCG00000047395	2.135938361	2.29E-17
ENSSSCG00000003776	2.134621963	5.47E-15
ENSSSCG00000011410	2.134524487	1.88E-08
ENSSSCG00000017137	2.133052058	7.51E-08
ENSSSCG00000004179	2.132888346	0.001026154
ENSSSCG00000016220	2.13010456	1.17E-13
ENSSSCG00000012247	2.129528869	9.12E-18
ENSSSCG00000040397	2.129372626	4.77E-05
ENSSSCG00000038964	2.127353357	3.61E-08
ENSSSCG00000003756	2.12723631	0.000204728
ENSSSCG00000003779	2.125583345	1.91E-08
ENSSSCG00000010517	2.125302585	1.16E-17
ENSSSCG00000017261	2.124599626	5.31E-08
ENSSSCG00000032527	2.123750747	6.70E-09
ENSSSCG00000044518	2.12371926	5.65E-06
ENSSSCG00000029652	2.123335404	1.47E-19
ENSSSCG00000004542	2.121189609	4.26E-20

ENSSSCG00000027689	2.120308999	1.18E-11
ENSSSCG00000040931	2.119875656	7.28E-23
ENSSSCG00000034207	2.117990958	1.80E-12
ENSSSCG00000036613	2.116839244	1.34E-14
ENSSSCG00000004160	2.113161004	2.20E-06
ENSSSCG00000032963	2.113054199	0.00261653
ENSSSCG00000007861	2.111721927	1.64E-10
ENSSSCG00000006378	2.111160898	7.00E-19
ENSSSCG00000003134	2.109325858	5.17E-13
ENSSSCG00000024310	2.108397782	1.36E-16
ENSSSCG00000047247	2.106370616	2.91E-11
ENSSSCG00000010568	2.105535081	6.09E-12
ENSSSCG00000028132	2.10474236	1.42E-16
ENSSSCG00000031837	2.104713295	6.51E-10
ENSSSCG00000045512	2.104634313	1.28E-10
ENSSSCG00000010865	2.103858273	1.11E-15
ENSSSCG00000010345	2.103825821	1.67E-23
ENSSSCG00000047379	2.103327533	6.03E-17
ENSSSCG00000033451	2.103235915	1.16E-05
ENSSSCG00000015360	2.102853612	1.42E-23
ENSSSCG00000034698	2.100437867	6.04E-10
ENSSSCG00000007737	2.100034886	2.65E-21
ENSSSCG00000032607	2.096874658	2.83E-05
ENSSSCG00000005164	2.096857766	8.41E-08
ENSSSCG00000014249	2.096268899	1.67E-08
ENSSSCG00000003419	2.095293939	1.17E-11
ENSSSCG00000027119	2.095101392	0.00018249
ENSSSCG00000040379	2.094078443	3.46E-16
ENSSSCG00000003132	2.092672357	4.53E-12
ENSSSCG00000026446	2.091812196	2.85E-17
ENSSSCG00000014600	2.091101437	1.46E-14
ENSSSCG00000002516	2.089997534	2.31E-36
ENSSSCG00000038149	2.08866302	3.10E-09
ENSSSCG00000038112	2.08738326	7.38E-07
ENSSSCG00000006667	2.087227666	9.91E-09
ENSSSCG00000000197	2.086931662	0.006052185
ENSSSCG00000033780	2.08658736	0.004447148
ENSSSCG00000000002	2.085757328	7.66E-07
ENSSSCG00000000864	2.085666779	1.33E-10
ENSSSCG00000015483	2.085498501	1.21E-16
ENSSSCG00000029945	2.084823806	8.49E-05
ENSSSCG00000012927	2.084533183	7.69E-10
ENSSSCG00000029231	2.083057941	4.24E-11
ENSSSCG00000046439	2.082486885	1.33E-08
ENSSSCG00000004344	2.081062305	6.89E-18
ENSSSCG00000015550	2.080355298	9.66E-12
ENSSSCG00000029425	2.079859045	4.13E-11
ENSSSCG00000016519	2.076919738	3.57E-05
ENSSSCG00000016616	2.075328784	2.36E-18
ENSSSCG00000012953	2.074444534	1.74E-09
ENSSSCG00000000394	2.071784128	1.11E-14
ENSSSCG00000034696	2.071746255	0.0007141
ENSSSCG00000024411	2.071541872	1.37E-12
ENSSSCG00000021834	2.071196275	7.93E-16
ENSSSCG00000030108	2.071052117	1.09E-15
ENSSSCG00000004466	2.070279929	1.85E-19
ENSSSCG00000000690	2.069966162	1.92E-13
ENSSSCG00000004766	2.069582497	8.76E-13

ENSSSCG00000014248	2.069167358	1.66E-18
ENSSSCG00000007743	2.067821457	6.61E-11
ENSSSCG00000028964	2.066694061	7.73E-24
ENSSSCG00000035677	2.065846777	0.003719331
ENSSSCG00000003109	2.061279246	1.75E-06
ENSSSCG00000028501	2.061029204	1.90E-08
ENSSSCG00000026602	2.05910705	2.99E-16
ENSSSCG00000012402	2.05863251	1.94E-10
ENSSSCG00000030653	2.057439522	8.03E-34
ENSSSCG00000033272	2.05599936	1.11E-25
ENSSSCG00000038401	2.055729372	2.25E-11
ENSSSCG00000028977	2.055695988	0.01239874
ENSSSCG00000041146	2.055372579	0.001324229
ENSSSCG00000041665	2.055192848	5.06E-07
ENSSSCG00000033381	2.05429996	1.75E-19
ENSSSCG00000000656	2.053645267	2.56E-09
ENSSSCG00000017544	2.053552306	1.53E-06
ENSSSCG00000003551	2.053282975	9.64E-14
ENSSSCG00000006740	2.052090653	3.89E-34
ENSSSCG00000029264	2.051099105	2.59E-07
ENSSSCG00000028889	2.04927778	1.90E-17
ENSSSCG00000004646	2.046261098	5.08E-13
ENSSSCG00000035893	2.045477285	0.000107008
ENSSSCG00000031635	2.045386655	0.000114452
ENSSSCG00000011864	2.045363568	8.13E-28
ENSSSCG00000022237	2.044653928	2.15E-11
ENSSSCG00000012055	2.044080627	3.50E-18
ENSSSCG00000009794	2.043010212	7.40E-05
ENSSSCG00000024189	2.042370076	9.63E-11
ENSSSCG00000027294	2.041365995	2.94E-11
ENSSSCG00000016233	2.0411132	1.38E-15
ENSSSCG00000005619	2.038171409	4.04E-10
ENSSSCG00000033913	2.037898126	7.25E-12
ENSSSCG00000008742	2.037631142	2.27E-19
ENSSSCG00000015600	2.037413036	3.79E-05
ENSSSCG00000047625	2.034653775	0.005179726
ENSSSCG00000033816	2.032201383	3.04E-14
ENSSSCG00000046466	2.029466526	5.90E-11
ENSSSCG00000023531	2.028851296	1.32E-06
ENSSSCG00000004456	2.028447514	1.28E-15
ENSSSCG00000051135	2.026470806	0.007365706
ENSSSCG00000007133	2.025433357	3.58E-30
ENSSSCG00000027855	2.022382686	4.08E-12
ENSSSCG00000010495	2.022277348	3.33E-20
ENSSSCG00000023807	2.021073348	6.39E-14
ENSSSCG00000034012	2.019448189	4.48E-17
ENSSSCG00000023630	2.018644095	9.45E-05
ENSSSCG00000007173	2.018166225	8.46E-16
ENSSSCG00000005994	2.017973933	2.07E-12
ENSSSCG00000010186	2.017829087	7.15E-09
ENSSSCG00000039021	2.01754083	2.95E-11
ENSSSCG00000015735	2.017246726	3.69E-05
ENSSSCG00000023333	2.014921338	7.71E-11
ENSSSCG00000006487	2.013539504	5.68E-22
ENSSSCG00000009178	2.012678242	7.61E-10
ENSSSCG00000000667	2.012186569	1.72E-06
ENSSSCG00000029371	2.011673818	8.07E-09
ENSSSCG00000030888	2.010879443	6.36E-09

ENSSSCG00000017342	2.010535438	1.20E-13
ENSSSCG00000004822	2.009533835	1.45E-14
ENSSSCG00000000648	2.008734081	0.001062759
ENSSSCG00000009378	2.008689521	3.36E-13
ENSSSCG00000007795	2.007282602	4.21E-23
ENSSSCG00000013232	2.006206829	4.10E-10
ENSSSCG00000013411	2.006139311	4.60E-16
ENSSSCG00000010292	2.005127269	5.50E-16
ENSSSCG00000021161	2.002366575	3.29E-11
ENSSSCG00000039922	1.999998332	0.006290362
ENSSSCG00000007097	1.999712234	8.19E-23
ENSSSCG00000026617	1.999626807	2.15E-17
ENSSSCG00000036584	1.998935557	8.81E-15
ENSSSCG00000027257	1.998897836	1.93E-08
ENSSSCG00000039556	1.997844886	5.15E-06
ENSSSCG00000013517	1.997505546	7.91E-05
ENSSSCG00000018057	1.996916719	0.000123018
ENSSSCG00000015794	1.996901765	5.38E-09
ENSSSCG00000000488	1.996497609	8.32E-23
ENSSSCG00000032710	1.994783967	2.20E-19
ENSSSCG00000005105	1.992634084	3.15E-10
ENSSSCG00000006059	1.991186751	2.36E-13
ENSSSCG00000012845	1.990406955	1.13E-06
ENSSSCG00000040743	1.99016989	8.80E-19
ENSSSCG00000034487	1.989785266	8.85E-07
ENSSSCG00000001836	1.988362994	7.07E-10
ENSSSCG00000009077	1.987967429	1.28E-10
ENSSSCG00000002868	1.987914084	9.45E-10
ENSSSCG00000022864	1.987320619	9.82E-10
ENSSSCG00000006620	1.987318897	9.80E-06
ENSSSCG00000007763	1.986151414	8.22E-16
ENSSSCG00000031617	1.983995596	6.80E-09
ENSSSCG00000033295	1.982715688	2.72E-20
ENSSSCG00000033310	1.981485076	4.94E-08
ENSSSCG00000000975	1.980099545	5.06E-11
ENSSSCG00000015379	1.980038747	0.005608669
ENSSSCG00000020856	1.979910957	4.84E-24
ENSSSCG00000033608	1.978771076	3.02E-12
ENSSSCG00000008094	1.978542422	5.08E-06
ENSSSCG00000009646	1.97837177	0.000967074
ENSSSCG00000001973	1.977753913	5.38E-19
ENSSSCG00000009868	1.975789662	1.98E-18
ENSSSCG00000011676	1.974783655	5.91E-13
ENSSSCG00000003513	1.973057115	3.78E-17
ENSSSCG00000009854	1.971221709	1.67E-15
ENSSSCG00000002754	1.970805479	1.30E-11
ENSSSCG00000007525	1.970361102	4.73E-09
ENSSSCG00000037465	1.969503019	4.02E-13
ENSSSCG00000001867	1.969305317	1.75E-12
ENSSSCG00000040281	1.968964959	2.42E-18
ENSSSCG00000016243	1.968505871	5.76E-22
ENSSSCG00000022151	1.967568336	0.000112723
ENSSSCG00000036612	1.967422317	6.92E-06
ENSSSCG00000001422	1.966752016	7.32E-12
ENSSSCG00000035561	1.966373557	2.14E-06
ENSSSCG00000009886	1.965654595	2.29E-23
ENSSSCG00000006359	1.964789759	0.000599418
ENSSSCG00000015924	1.964632338	2.48E-11

ENSSSCG00000007228	1.964565214	2.20E-11
ENSSSCG00000001816	1.963343224	1.79E-14
ENSSSCG00000007366	1.962644503	1.63E-21
ENSSSCG00000037598	1.961061622	2.03E-12
ENSSSCG00000000368	1.960282716	1.53E-14
ENSSSCG00000000859	1.960090007	6.05E-11
ENSSSCG00000016659	1.96005453	8.89E-09
ENSSSCG00000008820	1.959905724	1.41E-17
ENSSSCG00000031773	1.958619528	5.03E-18
ENSSSCG00000022659	1.958580042	6.89E-22
ENSSSCG00000011630	1.958281366	1.01E-09
ENSSSCG00000030125	1.956982458	3.59E-18
ENSSSCG00000013332	1.955444664	9.50E-16
ENSSSCG00000001531	1.955060091	9.07E-10
ENSSSCG00000033854	1.954982635	4.44E-07
ENSSSCG00000014670	1.95335597	3.06E-24
ENSSSCG00000022226	1.951307156	5.03E-12
ENSSSCG00000006751	1.950683694	3.57E-06
ENSSSCG00000008496	1.950098688	8.66E-23
ENSSSCG00000000738	1.949391579	3.02E-18
ENSSSCG00000011171	1.949292878	5.09E-13
ENSSSCG00000029938	1.948837233	0.002072577
ENSSSCG00000028288	1.948803047	4.31E-19
ENSSSCG00000038914	1.946578662	2.19E-07
ENSSSCG00000024059	1.94442575	9.62E-22
ENSSSCG00000001022	1.943833368	1.75E-15
ENSSSCG00000017517	1.943620412	2.74E-26
ENSSSCG00000002822	1.943520345	5.25E-14
ENSSSCG00000004245	1.942396019	0.002534992
ENSSSCG00000014924	1.939612571	2.99E-17
ENSSSCG00000006078	1.939314206	4.38E-10
ENSSSCG00000008303	1.937735357	7.43E-16
ENSSSCG00000008557	1.937274521	3.40E-10
ENSSSCG00000028821	1.936182472	5.82E-21
ENSSSCG00000002433	1.933609998	1.17E-10
ENSSSCG00000024018	1.933450769	5.36E-08
ENSSSCG00000008300	1.933401061	6.71E-15
ENSSSCG00000011356	1.931884648	1.95E-12
ENSSSCG00000006358	1.931704545	4.41E-22
ENSSSCG00000006386	1.931276306	7.69E-09
ENSSSCG00000017525	1.929597783	2.51E-16
ENSSSCG00000008540	1.928838401	3.58E-19
ENSSSCG00000011179	1.926226706	3.00E-16
ENSSSCG00000011425	1.92186064	0.008748621
ENSSSCG00000010606	1.92145228	0.000696706
ENSSSCG00000033860	1.919827415	2.03E-05
ENSSSCG00000013178	1.918407734	2.51E-09
ENSSSCG00000008304	1.917947221	3.70E-07
ENSSSCG00000013936	1.917754856	3.76E-07
ENSSSCG00000010540	1.917077974	1.21E-12
ENSSSCG00000017236	1.916572047	8.66E-07
ENSSSCG00000005267	1.914682424	3.92E-12
ENSSSCG00000038079	1.914598676	1.87E-06
ENSSSCG00000022322	1.913951224	4.34E-28
ENSSSCG00000012375	1.91149105	1.55E-11
ENSSSCG00000014337	1.909729083	1.12E-20
ENSSSCG00000004077	1.909562382	1.39E-12
ENSSSCG00000046387	1.909383507	2.42E-23

ENSSSCG00000032782	1.909213462	2.37E-11
ENSSSCG00000016218	1.907878799	2.05E-06
ENSSSCG00000024614	1.907508848	2.41E-11
ENSSSCG00000024676	1.907211157	6.60E-05
ENSSSCG00000008202	1.905515734	1.34E-12
ENSSSCG00000016834	1.905132934	0.000157374
ENSSSCG00000026752	1.904216378	5.26E-10
ENSSSCG00000017861	1.903122529	1.67E-07
ENSSSCG00000009704	1.901510105	1.24E-15
ENSSSCG00000000034	1.901101887	9.66E-20
ENSSSCG00000015604	1.900691249	7.33E-13
ENSSSCG00000035211	1.900660311	0.000169387
ENSSSCG00000007355	1.900364649	9.85E-11
ENSSSCG00000020915	1.900202255	7.74E-18
ENSSSCG00000038779	1.899000631	5.76E-18
ENSSSCG00000037343	1.898688103	3.98E-09
ENSSSCG00000022742	1.898395925	5.81E-11
ENSSSCG00000036658	1.897616803	3.89E-10
ENSSSCG00000001232	1.896647184	8.20E-07
ENSSSCG00000002767	1.895176387	0.000331375
ENSSSCG00000010260	1.895157904	2.89E-17
ENSSSCG00000013100	1.893505077	5.51E-07
ENSSSCG00000039782	1.893138756	1.07E-11
ENSSSCG00000033057	1.892509077	3.25E-08
ENSSSCG00000040793	1.891722152	7.52E-08
ENSSSCG00000017052	1.890549419	2.77E-16
ENSSSCG00000025245	1.890294242	2.89E-07
ENSSSCG00000001418	1.890136232	2.16E-17
ENSSSCG00000013891	1.889053282	1.10E-08
ENSSSCG00000034120	1.888571243	5.09E-07
ENSSSCG00000040119	1.887393859	0.000133306
ENSSSCG00000003555	1.887158824	9.59E-07
ENSSSCG00000016189	1.885783399	1.11E-08
ENSSSCG00000037929	1.884722322	0.000538019
ENSSSCG00000033673	1.884677841	7.43E-12
ENSSSCG00000030318	1.883582084	8.29E-13
ENSSSCG00000014835	1.88327161	4.66E-10
ENSSSCG00000037314	1.882624482	2.46E-15
ENSSSCG00000011806	1.882456083	4.03E-12
ENSSSCG00000010477	1.881435731	1.31E-17
ENSSSCG00000011562	1.881296651	4.60E-13
ENSSSCG00000036832	1.881247475	1.08E-19
ENSSSCG00000003949	1.881003072	4.15E-10
ENSSSCG00000031938	1.880659474	2.08E-06
ENSSSCG00000014959	1.880610031	1.83E-17
ENSSSCG00000037425	1.879990128	2.17E-06
ENSSSCG00000030053	1.87773086	1.29E-12
ENSSSCG00000004403	1.876953446	3.65E-05
ENSSSCG00000007235	1.876303809	7.14E-25
ENSSSCG00000022149	1.876211506	1.46E-19
ENSSSCG00000026686	1.873630692	3.38E-11
ENSSSCG00000015892	1.873000715	5.24E-14
ENSSSCG00000024316	1.872051115	3.00E-08
ENSSSCG00000034353	1.868480386	1.94E-05
ENSSSCG00000035611	1.867510036	2.28E-09
ENSSSCG00000021739	1.867412703	1.67E-17
ENSSSCG00000015872	1.866970178	8.02E-13
ENSSSCG00000014997	1.865942963	1.88E-16

ENSSSCG00000040608	1.865942147	1.00E-09
ENSSSCG00000013940	1.863459754	4.10E-10
ENSSSCG00000027902	1.863267896	2.44E-10
ENSSSCG00000034045	1.862731511	8.95E-11
ENSSSCG00000038062	1.862401044	4.06E-10
ENSSSCG00000011624	1.862013751	2.62E-26
ENSSSCG00000025486	1.861670223	6.73E-24
ENSSSCG00000032623	1.861650792	0.005639112
ENSSSCG00000028227	1.860152779	3.78E-16
ENSSSCG00000015009	1.859792351	1.11E-07
ENSSSCG00000001769	1.859004486	1.94E-21
ENSSSCG00000032806	1.858685394	1.18E-11
ENSSSCG00000016057	1.85727676	1.04E-21
ENSSSCG00000039191	1.856752352	5.19E-14
ENSSSCG00000017262	1.855897426	1.27E-09
ENSSSCG00000008309	1.855741981	3.02E-12
ENSSSCG00000032908	1.855708628	2.10E-13
ENSSSCG00000014054	1.855384646	0.00030375
ENSSSCG00000003160	1.855273038	0.002057244
ENSSSCG00000024166	1.854195486	4.73E-11
ENSSSCG00000030115	1.85356143	9.71E-12
ENSSSCG00000009881	1.853444178	4.76E-27
ENSSSCG00000014036	1.852346922	1.26E-16
ENSSSCG00000038562	1.850999919	1.13E-08
ENSSSCG00000012258	1.849256887	3.39E-10
ENSSSCG00000012853	1.849184036	3.26E-07
ENSSSCG00000000228	1.845998203	1.17E-13
ENSSSCG00000002535	1.845390274	9.01E-17
ENSSSCG00000011561	1.84534443	6.40E-08
ENSSSCG00000036256	1.84525794	7.49E-08
ENSSSCG00000023886	1.84507138	1.09E-06
ENSSSCG00000017731	1.844891754	9.83E-22
ENSSSCG00000006193	1.843295028	6.44E-10
ENSSSCG00000012348	1.843146397	6.69E-12
ENSSSCG00000010276	1.841890793	0.008428591
ENSSSCG00000005949	1.841702792	1.39E-20
ENSSSCG00000004392	1.840691801	5.42E-14
ENSSSCG00000017047	1.840478181	2.22E-13
ENSSSCG00000034572	1.840063726	2.68E-08
ENSSSCG00000050193	1.838804598	0.000181288
ENSSSCG00000003432	1.837820867	9.93E-10
ENSSSCG00000009393	1.837064338	0.000618579
ENSSSCG00000051304	1.836564788	0.007126562
ENSSSCG00000039619	1.83611231	2.06E-08
ENSSSCG00000008414	1.835527643	2.11E-11
ENSSSCG00000006734	1.83527579	9.79E-07
ENSSSCG00000040535	1.834997391	3.84E-07
ENSSSCG00000039695	1.834898932	0.001164119
ENSSSCG00000005486	1.833755123	0.000530876
ENSSSCG00000021828	1.833246747	2.62E-16
ENSSSCG00000048507	1.832577989	0.01484611
ENSSSCG00000039494	1.832494853	4.59E-07
ENSSSCG00000025870	1.832189098	1.45E-07
ENSSSCG00000007671	1.830574522	2.56E-05
ENSSSCG00000029183	1.830007701	3.71E-10
ENSSSCG00000009914	1.829773189	2.32E-23
ENSSSCG00000000161	1.829528358	6.85E-12
ENSSSCG00000017260	1.827628426	3.30E-19

ENSSSCG00000016085	1.826636721	0.000494771
ENSSSCG00000016275	1.82613945	3.48E-20
ENSSSCG00000017971	1.825869851	1.36E-07
ENSSSCG00000013599	1.82516473	3.78E-08
ENSSSCG00000039327	1.824928367	4.84E-08
ENSSSCG00000028748	1.824924593	1.40E-16
ENSSSCG00000008553	1.824821195	4.24E-17
ENSSSCG00000009466	1.824763512	3.81E-08
ENSSSCG00000000186	1.822744084	0.007808537
ENSSSCG00000011465	1.822623347	5.44E-09
ENSSSCG00000011916	1.821271985	1.00E-11
ENSSSCG00000026812	1.820997712	1.87E-08
ENSSSCG00000013497	1.820743275	1.34E-09
ENSSSCG00000001723	1.820675921	1.91E-10
ENSSSCG00000009759	1.820634684	1.94E-20
ENSSSCG00000017364	1.819068351	7.28E-12
ENSSSCG00000016122	1.81902576	7.80E-12
ENSSSCG00000000860	1.81741127	8.14E-09
ENSSSCG00000015581	1.817409344	3.73E-07
ENSSSCG00000012042	1.817194947	2.06E-07
ENSSSCG00000023873	1.814865636	5.16E-15
ENSSSCG00000016672	1.814337519	6.27E-15
ENSSSCG00000036328	1.813594253	1.24E-08
ENSSSCG00000003876	1.811136465	8.32E-11
ENSSSCG00000001516	1.810702685	3.31E-12
ENSSSCG00000044686	1.810507453	0.000681353
ENSSSCG00000040154	1.810253512	4.71E-08
ENSSSCG00000000683	1.810210644	4.85E-09
ENSSSCG00000001475	1.810066818	2.29E-17
ENSSSCG00000025027	1.809405592	2.78E-13
ENSSSCG00000011197	1.809049009	2.57E-12
ENSSSCG00000024791	1.808935715	2.73E-17
ENSSSCG00000015414	1.808620717	2.29E-09
ENSSSCG00000007709	1.807571163	1.50E-19
ENSSSCG00000008602	1.805444565	1.82E-16
ENSSSCG00000027085	1.804567659	5.91E-11
ENSSSCG00000043447	1.804379261	0.009102425
ENSSSCG00000030642	1.80394916	5.64E-13
ENSSSCG00000012857	1.803174672	2.50E-22
ENSSSCG00000001638	1.802299788	4.75E-12
ENSSSCG00000033116	1.802233461	2.03E-07
ENSSSCG00000002814	1.800974155	0.000172801
ENSSSCG00000027334	1.800476569	8.61E-20
ENSSSCG00000015362	1.799787947	3.02E-14
ENSSSCG00000037372	1.799744666	4.47E-05
ENSSSCG00000011048	1.799696612	4.24E-11
ENSSSCG00000021971	1.798880336	0.000351557
ENSSSCG00000017717	1.795980994	2.85E-07
ENSSSCG00000002050	1.794919233	3.82E-08
ENSSSCG00000034190	1.793910697	2.04E-09
ENSSSCG00000032221	1.792748744	2.66E-05
ENSSSCG00000032633	1.792616788	1.61E-06
ENSSSCG00000001848	1.792293411	0.001019798
ENSSSCG00000047578	1.791872007	0.000738092
ENSSSCG00000015897	1.790849734	3.64E-18
ENSSSCG00000016629	1.790747548	6.51E-15
ENSSSCG00000011804	1.790734921	1.08E-13
ENSSSCG00000013460	1.790318046	1.49E-07

ENSSSCG00000038422	1.790089094	6.45E-13
ENSSSCG00000032115	1.789298913	4.57E-06
ENSSSCG00000000843	1.789215168	2.51E-18
ENSSSCG00000003914	1.789030384	1.81E-09
ENSSSCG00000013226	1.787428453	9.28E-13
ENSSSCG00000001847	1.786835492	0.004501351
ENSSSCG00000008359	1.786429401	1.01E-15
ENSSSCG00000009593	1.786240679	5.87E-07
ENSSSCG00000042512	1.785150238	3.21E-07
ENSSSCG00000003885	1.78347834	2.48E-10
ENSSSCG00000004058	1.782681978	4.15E-14
ENSSSCG00000011570	1.782560427	2.94E-14
ENSSSCG00000029163	1.782364932	1.73E-14
ENSSSCG00000005508	1.78223307	0.002203006
ENSSSCG00000008980	1.781951009	2.24E-11
ENSSSCG00000004105	1.781062811	3.53E-12
ENSSSCG00000015690	1.780888068	4.04E-18
ENSSSCG00000034217	1.780732777	4.58E-10
ENSSSCG00000005095	1.780525103	5.73E-08
ENSSSCG00000000800	1.780496907	1.95E-07
ENSSSCG00000038348	1.78036465	7.90E-09
ENSSSCG00000034942	1.780357007	4.43E-07
ENSSSCG00000030582	1.779164256	5.64E-06
ENSSSCG00000015808	1.778457801	4.40E-08
ENSSSCG00000012315	1.778060593	4.84E-06
ENSSSCG00000026626	1.777719197	1.21E-07
ENSSSCG00000014195	1.777513728	4.94E-12
ENSSSCG00000038888	1.777287169	1.71E-05
ENSSSCG00000008898	1.777227719	3.06E-09
ENSSSCG00000005000	1.775042561	1.64E-09
ENSSSCG00000007529	1.7749408	0.005027944
ENSSSCG00000005908	1.774269227	1.45E-07
ENSSSCG00000025020	1.774014109	0.00638097
ENSSSCG00000013361	1.773298934	8.46E-17
ENSSSCG00000037185	1.771284535	8.61E-17
ENSSSCG00000011133	1.770829398	1.69E-10
ENSSSCG00000024267	1.769147773	9.06E-07
ENSSSCG00000040236	1.768770647	1.80E-09
ENSSSCG00000038938	1.768711693	2.10E-06
ENSSSCG00000008239	1.768216352	2.10E-06
ENSSSCG00000017273	1.767642976	2.74E-15
ENSSSCG00000048488	1.767400665	0.001924523
ENSSSCG00000035253	1.767240021	8.40E-10
ENSSSCG00000004588	1.76707585	9.61E-10
ENSSSCG00000016077	1.76705826	2.23E-13
ENSSSCG00000024290	1.765288772	0.004246674
ENSSSCG00000022780	1.763683028	0.010955735
ENSSSCG00000012448	1.759081153	9.45E-08
ENSSSCG00000031669	1.758519206	5.95E-12
ENSSSCG00000004180	1.757184397	0.007998769
ENSSSCG00000037606	1.756672776	1.47E-06
ENSSSCG00000036751	1.754970629	1.79E-09
ENSSSCG00000021557	1.753958826	8.86E-07
ENSSSCG00000000684	1.753908587	8.20E-15
ENSSSCG00000004935	1.751931063	1.18E-11
ENSSSCG00000021620	1.751320354	6.90E-19
ENSSSCG00000009542	1.749745705	2.43E-11
ENSSSCG00000013046	1.748233281	5.78E-07

ENSSSCG00000008019	1.747936627	2.74E-17
ENSSSCG00000006325	1.747799921	1.52E-05
ENSSSCG00000031485	1.747113534	0.000154639
ENSSSCG00000011752	1.746838029	2.02E-12
ENSSSCG00000003931	1.746145998	5.08E-17
ENSSSCG00000033025	1.74589267	9.11E-11
ENSSSCG00000031730	1.743493494	2.56E-07
ENSSSCG00000030165	1.740052379	1.28E-10
ENSSSCG00000007723	1.739897468	8.79E-07
ENSSSCG00000003177	1.739034451	6.93E-06
ENSSSCG00000037132	1.73900804	0.00649061
ENSSSCG00000038594	1.737675938	2.28E-10
ENSSSCG00000017690	1.73686226	1.36E-08
ENSSSCG00000006743	1.736609193	9.80E-05
ENSSSCG00000039662	1.736193866	3.85E-06
ENSSSCG00000017865	1.735929786	1.22E-10
ENSSSCG00000004317	1.734848702	1.44E-18
ENSSSCG00000043102	1.734297874	0.003245379
ENSSSCG00000004929	1.733840853	9.56E-13
ENSSSCG00000006273	1.73371634	1.08E-21
ENSSSCG00000000884	1.733417109	1.37E-06
ENSSSCG00000022222	1.732915739	1.71E-10
ENSSSCG00000003433	1.730921846	9.98E-21
ENSSSCG00000015952	1.730839819	4.01E-10
ENSSSCG00000022823	1.729859193	5.66E-16
ENSSSCG00000010874	1.729528774	2.56E-14
ENSSSCG00000012913	1.729515235	1.14E-07
ENSSSCG00000002959	1.729333242	3.01E-06
ENSSSCG00000009653	1.728474625	3.78E-09
ENSSSCG00000014088	1.727446791	2.77E-10
ENSSSCG00000006395	1.726497937	3.33E-07
ENSSSCG00000040811	1.725616397	1.26E-05
ENSSSCG00000033700	1.725076093	5.98E-09
ENSSSCG00000016841	1.72499373	0.002658748
ENSSSCG00000026931	1.724766044	6.17E-06
ENSSSCG00000012997	1.724575874	7.23E-11
ENSSSCG00000014020	1.724438426	4.57E-12
ENSSSCG00000007522	1.72395041	1.07E-09
ENSSSCG00000038717	1.723017999	4.13E-06
ENSSSCG00000004969	1.721106377	6.74E-16
ENSSSCG00000010774	1.721010022	1.96E-05
ENSSSCG00000031442	1.719927354	1.14E-11
ENSSSCG00000018033	1.718105938	3.67E-08
ENSSSCG00000033297	1.717053455	5.92E-13
ENSSSCG00000011444	1.71695695	3.06E-08
ENSSSCG00000009770	1.71692904	2.97E-14
ENSSSCG00000040634	1.716453836	1.31E-13
ENSSSCG00000007003	1.715508381	0.012831603
ENSSSCG00000015771	1.71424037	6.00E-10
ENSSSCG00000005615	1.714196849	1.06E-05
ENSSSCG00000015282	1.713754321	1.36E-09
ENSSSCG00000001436	1.713183534	1.26E-07
ENSSSCG00000005895	1.71169681	5.64E-06
ENSSSCG00000003694	1.710608667	1.55E-11
ENSSSCG00000035991	1.708943716	9.56E-08
ENSSSCG00000039071	1.7084885	9.77E-07
ENSSSCG00000033444	1.707319853	2.46E-06
ENSSSCG00000000623	1.70677595	8.55E-09

ENSSSCG00000015784	1.705148953	1.96E-11
ENSSSCG00000009820	1.704413588	7.17E-10
ENSSSCG00000040400	1.704196367	1.42E-11
ENSSSCG00000006733	1.704158272	4.28E-05
ENSSSCG00000000764	1.704039844	0.00727913
ENSSSCG00000000838	1.702468295	2.02E-13
ENSSSCG00000011391	1.702028891	1.02E-06
ENSSSCG00000006885	1.700480111	9.27E-10
ENSSSCG00000033374	1.700336671	3.75E-06
ENSSSCG00000015844	1.699940849	7.70E-10
ENSSSCG00000015945	1.699769072	4.57E-09
ENSSSCG00000009348	1.699720676	6.81E-11
ENSSSCG00000028802	1.699352182	1.92E-06
ENSSSCG00000015567	1.698876944	0.001557166
ENSSSCG00000029757	1.698351532	0.000255289
ENSSSCG00000007623	1.697568271	5.18E-06
ENSSSCG00000033731	1.696952295	2.81E-13
ENSSSCG00000031432	1.695646274	1.87E-11
ENSSSCG00000001362	1.695641254	1.44E-06
ENSSSCG00000011662	1.693590031	1.28E-14
ENSSSCG00000033944	1.693324845	2.93E-14
ENSSSCG00000038554	1.693255739	1.12E-14
ENSSSCG00000006853	1.69306936	2.78E-07
ENSSSCG00000037767	1.692982211	3.59E-08
ENSSSCG00000016737	1.692810164	5.20E-07
ENSSSCG00000011639	1.69250702	8.69E-15
ENSSSCG00000033385	1.691808365	0.004234176
ENSSSCG00000048696	1.691771473	0.00018941
ENSSSCG00000012000	1.691485965	5.28E-05
ENSSSCG00000038776	1.690358151	5.44E-08
ENSSSCG00000021380	1.69000257	2.67E-07
ENSSSCG00000023562	1.689541389	1.15E-05
ENSSSCG00000030420	1.688699293	1.04E-05
ENSSSCG00000002431	1.688608884	1.99E-12
ENSSSCG00000021319	1.687845945	1.07E-10
ENSSSCG00000036731	1.687516653	4.68E-06
ENSSSCG00000008368	1.687017349	1.60E-12
ENSSSCG00000013008	1.686328105	8.88E-18
ENSSSCG00000035417	1.685375901	0.016765148
ENSSSCG00000049430	1.685293755	3.61E-09
ENSSSCG00000017955	1.684914015	8.21E-06
ENSSSCG00000001817	1.684555143	6.86E-07
ENSSSCG00000011578	1.682827944	4.29E-09
ENSSSCG00000006850	1.681920669	0.000228573
ENSSSCG00000006488	1.68173804	8.01E-09
ENSSSCG00000010146	1.681304925	2.18E-14
ENSSSCG00000013853	1.680931206	4.18E-19
ENSSSCG00000023378	1.680704069	0.007435751
ENSSSCG00000026326	1.680363524	1.58E-17
ENSSSCG00000007475	1.679963693	2.78E-12
ENSSSCG00000014129	1.679664349	3.58E-09
ENSSSCG00000025440	1.67701926	4.64E-19
ENSSSCG00000002294	1.6765445	0.000628809
ENSSSCG00000017492	1.6765128	7.11E-11
ENSSSCG00000002354	1.676199074	4.36E-07
ENSSSCG00000027538	1.675473079	4.88E-06
ENSSSCG00000006641	1.672905935	1.03E-15
ENSSSCG00000016513	1.672866522	0.012907896

ENSSSCG00000013745	1.672529418	1.17E-17
ENSSSCG00000011535	1.671346831	2.01E-16
ENSSSCG00000001987	1.671160793	1.47E-12
ENSSSCG00000013110	1.669924085	1.41E-12
ENSSSCG00000025571	1.669688657	2.12E-06
ENSSSCG00000008227	1.668642767	4.16E-20
ENSSSCG00000022058	1.668474888	7.39E-10
ENSSSCG00000028694	1.6678404	1.30E-09
ENSSSCG00000001679	1.667231067	1.63E-18
ENSSSCG00000013392	1.66704891	2.51E-15
ENSSSCG00000033311	1.666965503	3.53E-08
ENSSSCG00000021652	1.666883796	3.12E-08
ENSSSCG00000012249	1.666681801	5.53E-13
ENSSSCG00000007019	1.666616655	8.84E-14
ENSSSCG00000023972	1.665940248	2.58E-09
ENSSSCG00000007282	1.665045713	2.63E-09
ENSSSCG00000003795	1.662876889	1.29E-08
ENSSSCG00000043494	1.662704509	0.000613286
ENSSSCG00000006552	1.661601589	1.53E-07
ENSSSCG00000034086	1.660395343	8.96E-09
ENSSSCG00000012560	1.660358098	1.35E-13
ENSSSCG00000023374	1.658950714	1.44E-05
ENSSSCG00000013391	1.657325092	3.30E-12
ENSSSCG00000049464	1.657033765	0.001018802
ENSSSCG00000015137	1.656321808	5.15E-10
ENSSSCG00000033169	1.655670857	7.18E-05
ENSSSCG00000023423	1.654575855	6.06E-07
ENSSSCG00000028978	1.653511636	6.07E-08
ENSSSCG00000003558	1.653227611	1.05E-18
ENSSSCG00000002496	1.652288585	1.45E-07
ENSSSCG00000022011	1.651604227	4.54E-08
ENSSSCG00000013303	1.651158366	9.92E-11
ENSSSCG00000013524	1.649846885	7.71E-05
ENSSSCG00000000033	1.649662852	0.000153343
ENSSSCG00000004789	1.649541903	3.00E-07
ENSSSCG00000004499	1.649324979	2.88E-11
ENSSSCG00000006048	1.648207145	2.60E-12
ENSSSCG00000006490	1.64742074	5.71E-20
ENSSSCG00000032819	1.647339662	2.85E-10
ENSSSCG00000035544	1.644794759	5.21E-15
ENSSSCG00000015067	1.6442228	3.55E-13
ENSSSCG00000004725	1.643423256	0.000510348
ENSSSCG00000002790	1.642998217	9.75E-06
ENSSSCG00000008829	1.642729933	8.27E-05
ENSSSCG00000029339	1.642585263	5.03E-05
ENSSSCG00000007710	1.641311691	0.014819151
ENSSSCG00000030643	1.640371244	4.35E-16
ENSSSCG00000031791	1.640234084	1.61E-10
ENSSSCG00000025881	1.640094333	3.19E-08
ENSSSCG00000039770	1.639997832	1.73E-17
ENSSSCG00000015860	1.63986868	5.89E-05
ENSSSCG00000011653	1.63950778	4.03E-10
ENSSSCG00000015976	1.639471514	1.07E-07
ENSSSCG00000032763	1.639223292	1.65E-05
ENSSSCG00000017549	1.638263346	4.18E-06
ENSSSCG00000032860	1.637610603	0.000391511
ENSSSCG00000046707	1.637073516	0.000708904
ENSSSCG00000000264	1.637029737	5.35E-07

ENSSSCG00000001661	1.636984743	1.00E-08
ENSSSCG00000009555	1.636465601	2.13E-10
ENSSSCG00000000721	1.636348128	1.25E-11
ENSSSCG00000022380	1.634360723	5.39E-06
ENSSSCG00000012392	1.633978446	0.013787206
ENSSSCG00000004629	1.633175681	0.00013199
ENSSSCG00000045632	1.631227911	4.12E-07
ENSSSCG00000026257	1.630294022	4.87E-09
ENSSSCG00000013467	1.63011014	6.13E-15
ENSSSCG00000023912	1.629983183	4.43E-18
ENSSSCG00000039778	1.629193284	4.64E-09
ENSSSCG00000006520	1.629064848	4.55E-08
ENSSSCG00000031105	1.628689111	5.91E-06
ENSSSCG00000037170	1.62846617	7.84E-06
ENSSSCG00000017913	1.628465649	1.15E-07
ENSSSCG00000015524	1.628230412	7.05E-11
ENSSSCG00000011563	1.627363865	1.47E-14
ENSSSCG00000025618	1.627278627	4.06E-15
ENSSSCG00000039290	1.627272812	3.34E-07
ENSSSCG00000016793	1.627090037	1.55E-10
ENSSSCG00000024206	1.62678572	6.63E-08
ENSSSCG00000007713	1.625563341	5.56E-08
ENSSSCG00000030118	1.625287774	9.00E-08
ENSSSCG00000007280	1.625114839	2.49E-14
ENSSSCG00000028840	1.624343351	1.07E-17
ENSSSCG00000024692	1.62420705	2.53E-14
ENSSSCG00000022492	1.623096616	1.55E-10
ENSSSCG00000015602	1.622897855	1.32E-13
ENSSSCG00000006392	1.622477713	4.78E-07
ENSSSCG00000017213	1.622411423	1.75E-08
ENSSSCG00000040678	1.621143925	8.62E-13
ENSSSCG00000008878	1.620127167	2.15E-09
ENSSSCG00000014579	1.620076587	6.83E-12
ENSSSCG00000016929	1.619395498	2.42E-09
ENSSSCG00000013181	1.619163293	7.54E-17
ENSSSCG00000009083	1.618689186	1.11E-10
ENSSSCG00000001910	1.618318991	9.25E-10
ENSSSCG00000026465	1.617824398	2.41E-12
ENSSSCG00000013896	1.617631074	2.07E-06
ENSSSCG00000036529	1.61721969	0.000643326
ENSSSCG00000040881	1.617049562	1.90E-06
ENSSSCG00000002697	1.616523097	2.14E-07
ENSSSCG00000012604	1.616020765	5.80E-11
ENSSSCG00000038156	1.615576557	1.46E-08
ENSSSCG00000013602	1.61554925	7.71E-16
ENSSSCG00000012950	1.614317732	0.001858167
ENSSSCG00000005047	1.614250413	1.52E-08
ENSSSCG00000031680	1.613661033	9.65E-06
ENSSSCG00000015140	1.613229716	1.49E-09
ENSSSCG00000035454	1.612472116	2.21E-13
ENSSSCG00000030426	1.61247027	9.38E-12
ENSSSCG00000001625	1.612229461	8.74E-05
ENSSSCG00000021871	1.611542999	9.09E-05
ENSSSCG00000001507	1.610920015	5.06E-08
ENSSSCG00000017835	1.610105777	4.17E-14
ENSSSCG00000015378	1.609974781	2.96E-08
ENSSSCG00000028553	1.609070315	1.41E-06
ENSSSCG00000040140	1.608501182	1.64E-10

ENSSSCG00000025218	1.607990183	1.13E-09
ENSSSCG00000017758	1.60758579	1.29E-05
ENSSSCG00000008491	1.60753783	5.16E-05
ENSSSCG00000030326	1.606669943	7.40E-16
ENSSSCG00000031808	1.606148869	2.27E-11
ENSSSCG00000016334	1.605700196	2.46E-10
ENSSSCG00000041063	1.605453894	1.03E-07
ENSSSCG00000017507	1.604784612	0.002443591
ENSSSCG00000031452	1.604239941	1.12E-11
ENSSSCG00000030546	1.60423718	8.19E-06
ENSSSCG00000007337	1.603209434	2.46E-14
ENSSSCG00000022240	1.601696421	3.36E-07
ENSSSCG00000017936	1.601301016	6.87E-09
ENSSSCG00000015331	1.601028198	1.44E-09
ENSSSCG00000005318	1.600425692	1.70E-13
ENSSSCG00000032655	1.599804356	3.71E-05
ENSSSCG00000025092	1.599648998	2.15E-07
ENSSSCG00000014252	1.599577218	6.27E-13
ENSSSCG00000010608	1.598733907	0.000194098
ENSSSCG00000010185	1.598475885	2.07E-13
ENSSSCG00000003386	1.598036023	4.81E-09
ENSSSCG00000030415	1.597152208	1.35E-13
ENSSSCG00000015709	1.597019299	5.09E-10
ENSSSCG00000037910	1.596474369	6.43E-09
ENSSSCG00000033278	1.594590047	4.94E-11
ENSSSCG00000004888	1.594562312	2.11E-07
ENSSSCG00000033690	1.593065215	7.98E-12
ENSSSCG00000009904	1.592152632	2.25E-06
ENSSSCG00000031381	1.591448708	2.13E-07
ENSSSCG00000009436	1.590480089	5.90E-08
ENSSSCG00000037305	1.589973309	2.21E-09
ENSSSCG00000011700	1.589501679	4.02E-10
ENSSSCG00000024259	1.589476316	3.88E-09
ENSSSCG00000008635	1.589156126	3.46E-13
ENSSSCG00000033051	1.589102079	1.06E-09
ENSSSCG00000015135	1.587982223	4.40E-08
ENSSSCG00000015093	1.587494902	3.43E-10
ENSSSCG00000035091	1.587491598	0.000471004
ENSSSCG00000017203	1.587328985	8.01E-05
ENSSSCG00000034984	1.587120775	2.08E-06
ENSSSCG00000000097	1.586813113	1.19E-06
ENSSSCG00000016928	1.586708484	2.12E-13
ENSSSCG00000011318	1.586663545	1.41E-07
ENSSSCG00000015598	1.586527499	2.21E-14
ENSSSCG00000004048	1.58610126	2.57E-10
ENSSSCG00000029279	1.585933386	5.21E-13
ENSSSCG00000037209	1.585403815	1.15E-14
ENSSSCG00000021464	1.585227657	6.83E-13
ENSSSCG00000030067	1.585060408	1.28E-08
ENSSSCG00000026520	1.584684267	0.009040979
ENSSSCG00000010402	1.584665564	0.000110262
ENSSSCG00000002013	1.5837988	1.97E-08
ENSSSCG00000000139	1.583369943	0.000871917
ENSSSCG00000023761	1.583335143	1.30E-13
ENSSSCG00000021988	1.583205661	0.001710632
ENSSSCG00000040139	1.583125023	2.49E-06
ENSSSCG00000010274	1.582790847	4.01E-13
ENSSSCG00000023548	1.581199185	7.70E-08

ENSSSCG00000014850	1.580891602	5.75E-08
ENSSSCG00000032591	1.58076925	0.000102933
ENSSSCG00000039947	1.580570241	0.000624116
ENSSSCG00000035134	1.580217875	5.82E-06
ENSSSCG00000012378	1.580148967	8.66E-09
ENSSSCG00000007715	1.579695769	1.18E-05
ENSSSCG00000038329	1.578611202	1.69E-12
ENSSSCG00000031835	1.578193567	3.66E-08
ENSSSCG00000013593	1.578142544	0.000251102
ENSSSCG00000005935	1.577969215	1.36E-06
ENSSSCG00000015335	1.577137045	2.51E-08
ENSSSCG00000016346	1.576870585	1.05E-10
ENSSSCG00000037213	1.576758032	4.76E-08
ENSSSCG00000017508	1.576414102	6.47E-06
ENSSSCG00000023837	1.574991025	2.38E-06
ENSSSCG00000013855	1.574845716	1.65E-07
ENSSSCG00000011229	1.574647528	5.66E-09
ENSSSCG00000007803	1.574500869	1.66E-08
ENSSSCG00000003137	1.574350296	3.71E-07
ENSSSCG00000024696	1.574107024	8.39E-09
ENSSSCG00000032985	1.573931963	8.01E-14
ENSSSCG00000041289	1.573753513	8.96E-09
ENSSSCG00000014934	1.573683966	1.32E-10
ENSSSCG00000012847	1.573605253	6.37E-06
ENSSSCG00000023174	1.573601203	5.20E-07
ENSSSCG00000006477	1.573586068	0.015107372
ENSSSCG00000036553	1.573353919	0.017496171
ENSSSCG00000008208	1.573247679	2.80E-07
ENSSSCG00000001934	1.572969283	4.42E-10
ENSSSCG00000031098	1.572644034	2.26E-12
ENSSSCG00000011424	1.572546719	6.09E-09
ENSSSCG00000005946	1.57091007	0.008421949
ENSSSCG00000030371	1.570821493	5.09E-06
ENSSSCG00000011978	1.569115632	1.06E-09
ENSSSCG00000028346	1.568680519	4.87E-07
ENSSSCG00000000217	1.568409957	1.63E-11
ENSSSCG00000000365	1.567905434	5.41E-08
ENSSSCG00000005678	1.567581286	2.19E-05
ENSSSCG00000003282	1.567127147	1.66E-08
ENSSSCG00000005911	1.566849938	2.27E-07
ENSSSCG00000036322	1.566815086	2.20E-09
ENSSSCG00000004782	1.566509454	4.06E-19
ENSSSCG00000028397	1.565435422	5.85E-11
ENSSSCG00000013576	1.565346005	6.93E-09
ENSSSCG00000016226	1.563508745	5.34E-08
ENSSSCG00000023785	1.563283418	1.61E-07
ENSSSCG00000013001	1.5631538	5.53E-05
ENSSSCG00000033421	1.563033843	2.23E-09
ENSSSCG00000014040	1.562634323	1.63E-09
ENSSSCG00000000182	1.562257235	5.95E-08
ENSSSCG00000016606	1.562082362	0.007360851
ENSSSCG00000031242	1.561659801	0.004750667
ENSSSCG00000002719	1.56143071	0.000436859
ENSSSCG00000000635	1.560181511	6.49E-09
ENSSSCG00000039546	1.559833793	1.62E-09
ENSSSCG00000035264	1.5597498	0.006212651
ENSSSCG00000038526	1.559477396	0.003253698
ENSSSCG00000010821	1.559009546	1.24E-11

ENSSSCG00000001990	1.558854585	1.26E-06
ENSSSCG00000035455	1.558593162	6.51E-11
ENSSSCG00000017192	1.558585761	1.72E-09
ENSSSCG00000009097	1.558539748	2.01E-09
ENSSSCG00000010247	1.557459026	9.07E-06
ENSSSCG00000020864	1.557208335	4.62E-06
ENSSSCG00000029753	1.555726354	9.42E-13
ENSSSCG00000012352	1.55555699	3.27E-06
ENSSSCG00000027051	1.554362416	9.37E-12
ENSSSCG00000015095	1.553953931	4.20E-09
ENSSSCG00000002081	1.553868564	0.004516773
ENSSSCG00000000082	1.553508984	9.48E-06
ENSSSCG00000039658	1.552809263	4.67E-12
ENSSSCG00000013105	1.551961901	2.48E-12
ENSSSCG00000003332	1.551343894	4.69E-09
ENSSSCG00000004480	1.551057536	7.51E-05
ENSSSCG00000028620	1.55025038	5.40E-13
ENSSSCG00000037277	1.549173362	1.13E-09
ENSSSCG00000038854	1.548871347	1.73E-12
ENSSSCG00000001379	1.548092406	1.16E-17
ENSSSCG00000013239	1.546813142	4.36E-15
ENSSSCG00000012784	1.546180828	0.000158723
ENSSSCG00000036865	1.543890748	0.000235908
ENSSSCG00000006494	1.543268723	4.40E-15
ENSSSCG00000037120	1.542752509	8.59E-06
ENSSSCG00000026784	1.542406362	1.02E-16
ENSSSCG00000017617	1.541792904	1.11E-09
ENSSSCG00000017747	1.541171365	0.000142232
ENSSSCG00000006379	1.540580138	3.21E-11
ENSSSCG00000016563	1.540488014	1.72E-13
ENSSSCG00000039372	1.540335156	5.63E-07
ENSSSCG00000013752	1.540171403	1.45E-05
ENSSSCG00000009839	1.540058273	3.61E-08
ENSSSCG00000004224	1.539771026	6.00E-06
ENSSSCG00000014804	1.539760622	6.00E-06
ENSSSCG00000039640	1.539434554	4.30E-05
ENSSSCG00000031913	1.539425984	4.44E-05
ENSSSCG00000039476	1.537930422	7.01E-05
ENSSSCG00000016520	1.536800924	7.95E-11
ENSSSCG00000010306	1.535601471	6.66E-10
ENSSSCG00000027660	1.53548323	1.25E-12
ENSSSCG00000025788	1.535172394	2.04E-10
ENSSSCG00000010029	1.533535747	2.15E-06
ENSSSCG00000002459	1.533343981	9.91E-06
ENSSSCG00000012631	1.533065131	8.36E-10
ENSSSCG00000033697	1.532940545	8.67E-07
ENSSSCG00000023280	1.532803834	0.000102318
ENSSSCG00000005959	1.532585274	1.66E-09
ENSSSCG00000033524	1.532578494	3.92E-05
ENSSSCG00000010496	1.532520046	4.73E-07
ENSSSCG00000006101	1.532406369	4.25E-05
ENSSSCG00000013003	1.532380558	0.000848759
ENSSSCG00000017087	1.531893563	1.04E-09
ENSSSCG00000032467	1.531817333	1.23E-11
ENSSSCG00000031789	1.531800569	4.04E-12
ENSSSCG00000039867	1.531432427	6.98E-11
ENSSSCG00000025685	1.531340306	1.69E-06
ENSSSCG00000015820	1.531111969	0.006037252

ENSSSCG00000005472	1.531009728	4.90E-07
ENSSSCG00000021384	1.530594033	0.000679621
ENSSSCG00000016078	1.530385665	3.29E-06
ENSSSCG00000006984	1.530275207	8.17E-12
ENSSSCG00000040060	1.529667699	8.04E-11
ENSSSCG00000035193	1.529456381	5.47E-05
ENSSSCG00000003578	1.528497644	2.83E-10
ENSSSCG00000017593	1.528214594	3.17E-08
ENSSSCG00000023441	1.527592362	0.014099335
ENSSSCG00000017877	1.527184503	5.91E-18
ENSSSCG00000015011	1.527170663	6.02E-10
ENSSSCG00000013304	1.527099915	8.67E-17
ENSSSCG00000010281	1.526673675	1.17E-12
ENSSSCG00000003652	1.526641556	8.46E-17
ENSSSCG00000031901	1.526638907	8.83E-14
ENSSSCG00000010576	1.526434484	4.63E-17
ENSSSCG00000002077	1.526378349	0.006225355
ENSSSCG00000043051	1.526029586	3.49E-06
ENSSSCG00000016215	1.525258164	1.20E-08
ENSSSCG00000001493	1.524959568	3.28E-08
ENSSSCG00000037975	1.524885322	5.53E-06
ENSSSCG00000038359	1.524211872	3.99E-08
ENSSSCG00000037168	1.524049902	0.002702485
ENSSSCG00000009592	1.523856457	7.56E-10
ENSSSCG00000017741	1.523015128	1.45E-05
ENSSSCG00000003903	1.52296349	1.45E-07
ENSSSCG00000032554	1.522358633	7.49E-15
ENSSSCG00000003089	1.522270126	2.88E-10
ENSSSCG00000011038	1.521966928	1.01E-07
ENSSSCG00000006480	1.521728837	8.64E-07
ENSSSCG00000037745	1.521074349	0.012732266
ENSSSCG00000006796	1.520919506	2.18E-08
ENSSSCG00000004986	1.51955654	9.40E-07
ENSSSCG00000014880	1.51938014	0.000399805
ENSSSCG00000005771	1.518784794	1.14E-07
ENSSSCG00000016210	1.518759392	1.26E-08
ENSSSCG00000034973	1.518727103	2.65E-12
ENSSSCG00000011297	1.51832837	2.55E-09
ENSSSCG00000026302	1.517555813	3.46E-09
ENSSSCG00000001984	1.517188086	2.18E-07
ENSSSCG00000018047	1.517091104	0.006743174
ENSSSCG00000006860	1.516571348	1.12E-12
ENSSSCG00000034386	1.516546895	1.35E-08
ENSSSCG00000034709	1.516465425	1.84E-12
ENSSSCG00000007472	1.515795618	0.000517723
ENSSSCG00000009448	1.515149079	3.26E-10
ENSSSCG00000031262	1.515065704	2.40E-06
ENSSSCG00000007331	1.51497225	6.99E-11
ENSSSCG00000003233	1.514384949	0.001909551
ENSSSCG00000024693	1.514228467	1.17E-06
ENSSSCG00000013436	1.513303904	0.000413444
ENSSSCG00000034159	1.512753702	5.91E-08
ENSSSCG00000012657	1.512188073	3.51E-14
ENSSSCG00000034816	1.511601358	3.29E-09
ENSSSCG00000008353	1.511491885	1.48E-06
ENSSSCG00000020737	1.510769853	0.000892037
ENSSSCG00000013556	1.510219511	2.47E-07
ENSSSCG00000051090	1.509599904	0.005122657

ENSSSCG00000012542	1.509589252	0.008439565
ENSSSCG00000007776	1.509587326	6.71E-05
ENSSSCG00000021761	1.509375568	1.58E-12
ENSSSCG00000038882	1.508661196	3.07E-05
ENSSSCG00000034265	1.506974514	8.08E-14
ENSSSCG00000011912	1.506387013	2.56E-11
ENSSSCG00000012056	1.505834532	1.46E-07
ENSSSCG00000039894	1.505577418	1.41E-06
ENSSSCG00000015730	1.505522497	2.15E-11
ENSSSCG00000006166	1.505139033	1.24E-05
ENSSSCG00000008335	1.505065216	0.01088746
ENSSSCG00000004178	1.504771077	3.95E-08
ENSSSCG00000029502	1.504288974	9.84E-12
ENSSSCG00000050940	1.50405111	0.005500563
ENSSSCG00000008845	1.503993466	1.24E-07
ENSSSCG00000011555	1.503991349	1.26E-06
ENSSSCG00000032340	1.503267684	7.63E-12
ENSSSCG00000004432	1.503152287	5.13E-11
ENSSSCG00000004671	1.502951085	0.000294696
ENSSSCG00000039500	1.502165405	4.08E-06
ENSSSCG00000000457	1.502104692	2.48E-14
ENSSSCG00000013906	1.499950137	1.73E-06
ENSSSCG00000000870	1.499527828	6.76E-13
ENSSSCG00000037782	1.499275986	0.000131654
ENSSSCG00000003468	1.498900377	2.39E-12
ENSSSCG00000017528	1.496722888	7.32E-08
ENSSSCG00000036810	1.496175341	1.67E-06
ENSSSCG00000000046	1.496079127	0.004849964
ENSSSCG00000034655	1.496032514	0.000179143
ENSSSCG00000005664	1.495601022	3.29E-11
ENSSSCG00000031866	1.49509637	2.88E-09
ENSSSCG00000039416	1.494787672	1.98E-12
ENSSSCG00000002819	1.494677477	4.56E-12
ENSSSCG00000041906	1.49451827	4.49E-07
ENSSSCG00000034518	1.494480458	1.15E-06
ENSSSCG00000003844	1.494435075	4.21E-08
ENSSSCG00000035696	1.493878258	8.26E-05
ENSSSCG00000000486	1.493812643	2.01E-13
ENSSSCG00000033577	1.493785392	2.13E-05
ENSSSCG00000033918	1.493423116	7.96E-12
ENSSSCG00000025329	1.49335121	1.25E-12
ENSSSCG00000032242	1.493226568	1.71E-15
ENSSSCG00000012029	1.493113329	1.93E-06
ENSSSCG00000015850	1.493082704	1.46E-11
ENSSSCG00000009692	1.492383861	3.20E-06
ENSSSCG00000016313	1.49224108	4.29E-12
ENSSSCG00000003820	1.490876129	3.44E-11
ENSSSCG00000017773	1.490669707	1.95E-05
ENSSSCG00000017226	1.490625318	0.000878235
ENSSSCG00000027045	1.489966559	0.004877267
ENSSSCG00000014367	1.489716544	1.91E-07
ENSSSCG00000006324	1.489268039	9.39E-09
ENSSSCG00000039318	1.489051858	3.68E-08
ENSSSCG00000004610	1.487816653	2.99E-09
ENSSSCG00000006738	1.486308761	5.46E-06
ENSSSCG00000026018	1.485324268	6.67E-07
ENSSSCG00000040570	1.485247151	5.72E-07
ENSSSCG00000021944	1.484654511	2.20E-08

ENSSSCG00000048264	1.484180707	1.53E-06
ENSSSCG00000028755	1.482868447	2.86E-05
ENSSSCG00000003554	1.481974938	1.51E-11
ENSSSCG00000008729	1.480950924	4.15E-11
ENSSSCG00000011601	1.480812777	0.000156189
ENSSSCG00000037319	1.48058205	8.60E-06
ENSSSCG00000010690	1.48053529	1.75E-11
ENSSSCG00000012743	1.480080157	4.82E-06
ENSSSCG00000032469	1.479793106	1.43E-16
ENSSSCG00000003645	1.47955743	2.55E-07
ENSSSCG00000001065	1.479424542	5.46E-08
ENSSSCG00000027525	1.47903064	0.006596982
ENSSSCG00000029456	1.478994594	9.91E-17
ENSSSCG00000034521	1.478986103	1.23E-11
ENSSSCG00000023263	1.478800476	9.82E-07
ENSSSCG00000006346	1.477896038	1.49E-14
ENSSSCG00000005610	1.477131617	4.44E-05
ENSSSCG00000022230	1.477072888	5.24E-09
ENSSSCG00000028518	1.476988193	1.33E-05
ENSSSCG00000029608	1.476412105	1.55E-08
ENSSSCG00000008377	1.476379916	3.59E-08
ENSSSCG00000035048	1.476235272	2.13E-07
ENSSSCG00000034261	1.476047874	1.05E-07
ENSSSCG00000049610	1.475415653	9.29E-11
ENSSSCG00000033006	1.47514202	6.51E-05
ENSSSCG00000006870	1.474865447	2.89E-06
ENSSSCG00000005096	1.474287039	1.06E-08
ENSSSCG00000017032	1.474107542	0.000228603
ENSSSCG00000024149	1.473032939	5.58E-11
ENSSSCG00000008332	1.472693544	1.10E-08
ENSSSCG00000007559	1.472337204	4.99E-05
ENSSSCG00000013751	1.472043265	6.38E-05
ENSSSCG00000022073	1.471886064	2.06E-06
ENSSSCG00000034562	1.471016835	3.31E-05
ENSSSCG00000031205	1.469733008	4.44E-05
ENSSSCG00000010679	1.469566967	1.06E-07
ENSSSCG00000033113	1.469088551	2.47E-11
ENSSSCG00000023296	1.469085427	6.22E-09
ENSSSCG00000036064	1.468984147	1.62E-05
ENSSSCG00000034415	1.468591533	1.05E-05
ENSSSCG00000017230	1.468058259	2.51E-12
ENSSSCG00000035676	1.467941554	2.67E-10
ENSSSCG00000037605	1.467750319	3.35E-05
ENSSSCG00000026153	1.467013803	2.27E-07
ENSSSCG00000027127	1.466964026	4.63E-07
ENSSSCG00000040905	1.466958641	2.36E-05
ENSSSCG00000000487	1.46569536	4.07E-07
ENSSSCG00000017642	1.464798381	6.60E-08
ENSSSCG00000032831	1.464518006	1.05E-11
ENSSSCG00000006029	1.463315065	3.36E-10
ENSSSCG00000037468	1.46270702	1.04E-09
ENSSSCG00000021891	1.462651597	1.67E-09
ENSSSCG00000038804	1.462567054	0.002082257
ENSSSCG00000024180	1.462223354	6.43E-10
ENSSSCG00000038443	1.462188751	1.47E-09
ENSSSCG00000014326	1.461707229	6.36E-14
ENSSSCG00000038399	1.461496439	2.10E-05
ENSSSCG00000005373	1.461320741	6.92E-10

ENSSSCG00000027002	1.460843248	3.11E-05
ENSSSCG00000008127	1.46073444	6.30E-10
ENSSSCG00000006501	1.460700687	8.23E-06
ENSSSCG00000026977	1.459607095	2.37E-08
ENSSSCG00000010357	1.459604566	4.59E-08
ENSSSCG00000033368	1.457524169	3.35E-06
ENSSSCG00000032692	1.457179883	0.006113799
ENSSSCG00000003119	1.456898964	0.010381341
ENSSSCG00000012602	1.456893947	6.18E-09
ENSSSCG00000008710	1.456822707	3.30E-11
ENSSSCG00000024860	1.45642515	1.38E-07
ENSSSCG00000039670	1.456257681	6.65E-08
ENSSSCG00000001346	1.455213796	4.53E-15
ENSSSCG00000013721	1.454904708	4.40E-05
ENSSSCG00000011874	1.454118308	3.59E-11
ENSSSCG00000022089	1.453454642	0.009898706
ENSSSCG00000014869	1.453129928	3.44E-06
ENSSSCG00000000640	1.453034803	1.17E-05
ENSSSCG00000015110	1.45279881	1.41E-13
ENSSSCG00000010341	1.452623061	4.40E-14
ENSSSCG00000033450	1.452435273	3.29E-09
ENSSSCG00000014829	1.452363146	1.55E-05
ENSSSCG00000010158	1.45192924	1.69E-06
ENSSSCG00000007718	1.451507123	0.001339592
ENSSSCG00000024081	1.451285825	1.85E-08
ENSSSCG00000001695	1.450032151	4.07E-08
ENSSSCG00000003943	1.449330713	3.01E-06
ENSSSCG00000023716	1.449224259	0.003603967
ENSSSCG00000034164	1.448795788	0.000397787
ENSSSCG00000017114	1.448086715	8.54E-05
ENSSSCG00000007554	1.447922488	7.81E-10
ENSSSCG00000037595	1.447673397	2.00E-05
ENSSSCG00000002952	1.447012105	0.000281434
ENSSSCG00000008311	1.445630246	0.001673087
ENSSSCG00000006636	1.445606279	0.000875939
ENSSSCG00000001556	1.44424362	1.04E-11
ENSSSCG00000015504	1.443189331	3.65E-08
ENSSSCG00000007323	1.442370109	8.54E-09
ENSSSCG00000002651	1.441641898	3.41E-06
ENSSSCG00000015056	1.441613635	1.84E-06
ENSSSCG00000004170	1.441558087	8.91E-05
ENSSSCG00000033585	1.441237993	0.003164352
ENSSSCG00000000136	1.440154326	7.12E-08
ENSSSCG00000028875	1.439411554	2.45E-09
ENSSSCG00000035596	1.437779101	0.000390112
ENSSSCG00000006661	1.437303577	3.56E-09
ENSSSCG00000017543	1.437089306	1.99E-14
ENSSSCG00000031800	1.435996877	0.000283245
ENSSSCG00000015776	1.435591257	1.42E-07
ENSSSCG00000009674	1.435322964	9.95E-07
ENSSSCG00000022430	1.434706992	6.50E-08
ENSSSCG00000017952	1.43375296	0.000211459
ENSSSCG00000002406	1.433544512	2.99E-10
ENSSSCG00000032404	1.433205268	3.53E-05
ENSSSCG00000016747	1.433119555	3.44E-08
ENSSSCG00000015943	1.432654423	9.26E-13
ENSSSCG00000022159	1.432528076	3.28E-11
ENSSSCG00000022903	1.431965699	0.014028184

ENSSSCG00000022994	1.431819228	2.69E-06
ENSSSCG00000005453	1.431642899	0.000557975
ENSSSCG00000032180	1.431616587	2.69E-12
ENSSSCG00000035006	1.43094579	1.09E-05
ENSSSCG00000031109	1.430426267	1.66E-06
ENSSSCG00000005951	1.430082674	4.96E-09
ENSSSCG00000012633	1.429751395	2.84E-09
ENSSSCG00000013125	1.429587836	5.68E-11
ENSSSCG00000015549	1.429499807	1.64E-09
ENSSSCG00000000058	1.429162535	2.31E-10
ENSSSCG00000035648	1.428689465	5.58E-06
ENSSSCG00000031388	1.426611624	1.39E-09
ENSSSCG00000012294	1.42631528	2.76E-07
ENSSSCG00000027307	1.426197024	0.00014213
ENSSSCG00000042444	1.426149494	0.011392637
ENSSSCG00000037131	1.425984136	1.83E-06
ENSSSCG00000036709	1.425848103	3.29E-05
ENSSSCG00000036920	1.425529913	2.04E-06
ENSSSCG00000027905	1.425281136	3.72E-08
ENSSSCG00000005596	1.425100371	1.16E-07
ENSSSCG00000004465	1.42383899	5.20E-06
ENSSSCG00000008452	1.423797194	2.62E-10
ENSSSCG00000006827	1.423636288	0.00069658
ENSSSCG00000022655	1.42314242	6.08E-06
ENSSSCG00000038543	1.423006845	3.24E-09
ENSSSCG00000033442	1.422538943	0.000519042
ENSSSCG00000006651	1.421876418	2.28E-13
ENSSSCG00000027840	1.421562902	4.47E-05
ENSSSCG00000007970	1.419827236	2.06E-06
ENSSSCG00000032059	1.419649803	0.000173412
ENSSSCG00000030369	1.419569889	3.67E-17
ENSSSCG00000026969	1.419343084	1.35E-08
ENSSSCG00000011420	1.419121872	0.000114063
ENSSSCG00000005617	1.419054463	2.52E-06
ENSSSCG00000006771	1.418952328	8.32E-08
ENSSSCG00000028592	1.418641479	3.25E-09
ENSSSCG00000010330	1.417657023	5.29E-05
ENSSSCG00000006522	1.416988663	8.86E-08
ENSSSCG00000004757	1.416852338	6.98E-10
ENSSSCG00000025854	1.416132046	1.00E-10
ENSSSCG00000007278	1.415501768	7.16E-13
ENSSSCG00000032768	1.414651744	3.64E-05
ENSSSCG00000003812	1.414407833	3.45E-09
ENSSSCG00000002283	1.413702645	4.96E-12
ENSSSCG00000037066	1.413348858	1.68E-12
ENSSSCG00000011061	1.41319477	3.29E-06
ENSSSCG00000034730	1.413177938	3.26E-14
ENSSSCG00000012050	1.41188534	6.17E-11
ENSSSCG00000016216	1.411498322	6.93E-10
ENSSSCG00000016127	1.410928422	1.23E-10
ENSSSCG00000041954	1.410468438	2.17E-10
ENSSSCG00000000176	1.410399751	1.04E-13
ENSSSCG00000030632	1.409829485	1.04E-12
ENSSSCG00000000519	1.409032098	2.47E-11
ENSSSCG00000013177	1.408698199	3.14E-13
ENSSSCG00000016248	1.408558983	1.70E-09
ENSSSCG00000000743	1.408318527	7.70E-12
ENSSSCG00000008788	1.408256427	2.35E-06

ENSSSCG00000007552	1.407812003	6.98E-06
ENSSSCG00000011631	1.406339454	1.06E-10
ENSSSCG00000039909	1.40614952	1.04E-11
ENSSSCG00000016985	1.4056839	1.41E-05
ENSSSCG00000027165	1.405631632	9.50E-07
ENSSSCG00000011551	1.405240134	4.49E-06
ENSSSCG00000010955	1.404698995	1.38E-13
ENSSSCG00000022405	1.404342043	2.16E-07
ENSSSCG00000015435	1.40414046	2.73E-11
ENSSSCG00000008979	1.404096487	4.17E-09
ENSSSCG00000028516	1.403728201	6.90E-08
ENSSSCG00000025589	1.403597029	0.000758473
ENSSSCG00000002749	1.403372208	2.73E-10
ENSSSCG00000015117	1.403043555	1.28E-10
ENSSSCG00000041723	1.40241267	0.005131454
ENSSSCG00000032911	1.402191247	0.010852427
ENSSSCG00000025326	1.401948339	4.89E-07
ENSSSCG00000031091	1.401095624	0.006260555
ENSSSCG00000031629	1.400654946	0.008452835
ENSSSCG00000028197	1.399939866	1.11E-06
ENSSSCG00000004393	1.397906639	1.04E-08
ENSSSCG00000006089	1.3976936	4.50E-12
ENSSSCG00000036761	1.397676402	1.09E-05
ENSSSCG00000004612	1.397581082	5.75E-07
ENSSSCG00000015843	1.397427123	1.74E-07
ENSSSCG00000021472	1.397289869	5.81E-06
ENSSSCG00000005680	1.396984111	0.002429798
ENSSSCG00000025900	1.396501971	2.43E-11
ENSSSCG00000001907	1.396226846	2.14E-05
ENSSSCG00000050627	1.395292451	7.22E-10
ENSSSCG00000026294	1.394843778	3.39E-09
ENSSSCG00000031236	1.394586089	2.45E-08
ENSSSCG00000033833	1.394433464	0.010077017
ENSSSCG00000000130	1.393885206	3.26E-15
ENSSSCG00000001960	1.393421499	6.56E-09
ENSSSCG00000032498	1.393364002	7.83E-10
ENSSSCG00000037501	1.393335986	0.000562195
ENSSSCG00000012896	1.39294876	6.62E-06
ENSSSCG00000025751	1.392402103	2.71E-08
ENSSSCG00000016170	1.392103155	7.60E-08
ENSSSCG00000032315	1.391717882	3.48E-07
ENSSSCG00000001757	1.391273067	1.53E-07
ENSSSCG00000003608	1.391099326	2.44E-11
ENSSSCG00000010706	1.391001966	3.60E-07
ENSSSCG00000014411	1.389401785	1.03E-15
ENSSSCG00000009502	1.38933023	0.002662042
ENSSSCG00000005355	1.389255946	8.95E-05
ENSSSCG00000009176	1.388937453	2.39E-08
ENSSSCG00000015652	1.38881663	0.00045098
ENSSSCG00000025488	1.388748549	2.12E-13
ENSSSCG00000010802	1.388449597	3.58E-07
ENSSSCG00000005608	1.387601222	9.36E-12
ENSSSCG00000032166	1.387273591	0.000459556
ENSSSCG00000024109	1.38643514	4.48E-10
ENSSSCG00000000045	1.386099522	7.77E-06
ENSSSCG00000008035	1.386068282	0.000111469
ENSSSCG00000005217	1.385163384	2.94E-06
ENSSSCG00000001701	1.384895758	5.23E-10

ENSSSCG00000013400	1.384590273	0.000536381
ENSSSCG00000027340	1.384068944	6.31E-08
ENSSSCG00000010368	1.383918272	1.18E-08
ENSSSCG00000021634	1.383641703	3.06E-12
ENSSSCG00000017601	1.383438258	1.10E-05
ENSSSCG00000022099	1.383432937	5.10E-05
ENSSSCG00000026217	1.383032113	0.000299005
ENSSSCG00000032390	1.382903882	0.000213522
ENSSSCG00000032557	1.382510184	2.74E-08
ENSSSCG00000010320	1.382097322	7.04E-08
ENSSSCG00000006175	1.381707223	0.000114087
ENSSSCG00000002259	1.381415649	0.007301974
ENSSSCG00000009211	1.381321661	4.31E-08
ENSSSCG00000034510	1.381240615	2.42E-07
ENSSSCG00000001888	1.38090737	0.001133041
ENSSSCG00000014296	1.380880685	2.79E-07
ENSSSCG00000040984	1.380132793	5.61E-07
ENSSSCG00000015428	1.378334152	3.65E-10
ENSSSCG00000009492	1.37772718	1.25E-07
ENSSSCG00000027528	1.37747762	0.000626988
ENSSSCG00000029991	1.377263349	5.15E-07
ENSSSCG00000040355	1.376976004	1.24E-06
ENSSSCG00000017950	1.376953704	4.14E-06
ENSSSCG00000003552	1.376192581	0.009821004
ENSSSCG00000023318	1.375590014	1.86E-14
ENSSSCG00000022031	1.375338361	6.74E-15
ENSSSCG00000036129	1.374853473	1.29E-10
ENSSSCG00000013510	1.374452118	3.33E-06
ENSSSCG00000003144	1.374381665	2.20E-08
ENSSSCG00000016865	1.374367207	5.75E-07
ENSSSCG00000004626	1.374179817	1.27E-05
ENSSSCG00000034293	1.374155078	1.05E-08
ENSSSCG00000024809	1.373948899	8.36E-05
ENSSSCG00000001233	1.373130612	2.05E-10
ENSSSCG00000023595	1.372994841	1.18E-06
ENSSSCG00000029790	1.372063419	9.17E-06
ENSSSCG00000005659	1.371569031	1.46E-08
ENSSSCG00000040875	1.371323804	9.81E-09
ENSSSCG00000026940	1.371158075	2.04E-13
ENSSSCG00000026552	1.371112866	2.38E-06
ENSSSCG00000028920	1.371019055	1.46E-08
ENSSSCG00000003691	1.370889586	2.19E-06
ENSSSCG00000004670	1.370492079	0.005330797
ENSSSCG00000029059	1.370462465	4.22E-17
ENSSSCG00000002316	1.369880631	0.003993393
ENSSSCG00000000754	1.369530162	2.54E-12
ENSSSCG00000017202	1.368817782	1.05E-07
ENSSSCG00000005625	1.368011081	1.42E-12
ENSSSCG00000017104	1.367035206	1.20E-14
ENSSSCG00000028010	1.367032849	2.37E-07
ENSSSCG00000013474	1.366834256	1.72E-10
ENSSSCG00000033735	1.366680975	8.26E-08
ENSSSCG00000000190	1.366574209	1.70E-07
ENSSSCG00000011882	1.366023214	1.92E-06
ENSSSCG00000040745	1.365221523	0.001839319
ENSSSCG00000001993	1.365173532	1.55E-06
ENSSSCG00000036941	1.365163185	0.002849937
ENSSSCG00000003382	1.364677943	8.27E-06

ENSSSCG00000036246	1.364491023	3.10E-10
ENSSSCG00000028297	1.364281485	5.34E-10
ENSSSCG00000032549	1.364015439	8.99E-10
ENSSSCG00000006764	1.363770369	3.85E-05
ENSSSCG00000012893	1.363516716	1.70E-06
ENSSSCG00000022806	1.363423692	3.46E-07
ENSSSCG00000037000	1.363189181	4.28E-09
ENSSSCG00000022993	1.363132279	0.001518131
ENSSSCG00000001696	1.362249034	0.000852583
ENSSSCG00000011746	1.36201515	7.05E-10
ENSSSCG00000010635	1.361108392	1.20E-09
ENSSSCG00000042062	1.361031758	0.00067685
ENSSSCG00000028186	1.360987798	3.31E-12
ENSSSCG00000010896	1.360860539	1.55E-06
ENSSSCG00000006928	1.360585854	2.85E-09
ENSSSCG00000036030	1.360415319	0.002941946
ENSSSCG00000051287	1.359863999	0.008862529
ENSSSCG00000030443	1.359519893	5.52E-08
ENSSSCG00000037420	1.359031496	0.009294475
ENSSSCG00000051359	1.358908017	0.003271546
ENSSSCG00000000804	1.358347951	1.04E-06
ENSSSCG00000039731	1.357326523	3.35E-05
ENSSSCG00000009434	1.356592286	1.31E-05
ENSSSCG00000011772	1.355384498	8.26E-08
ENSSSCG00000039659	1.353943531	0.000222447
ENSSSCG00000021866	1.353256983	5.39E-08
ENSSSCG00000032067	1.352521232	6.35E-09
ENSSSCG00000013367	1.352457927	1.06E-09
ENSSSCG00000003888	1.351945814	1.44E-06
ENSSSCG00000030425	1.351788909	4.00E-08
ENSSSCG00000004576	1.351595594	1.53E-07
ENSSSCG00000030351	1.351185376	4.97E-08
ENSSSCG00000034795	1.350771438	2.78E-06
ENSSSCG00000031595	1.350637205	0.002610844
ENSSSCG00000011389	1.349514401	2.63E-11
ENSSSCG00000016643	1.349079249	2.55E-08
ENSSSCG00000000639	1.348563786	0.015081494
ENSSSCG00000031251	1.348144716	2.05E-08
ENSSSCG00000029989	1.348029845	2.45E-08
ENSSSCG00000027147	1.347709757	0.000667535
ENSSSCG00000002329	1.347293047	0.001771419
ENSSSCG00000009906	1.34719759	3.55E-11
ENSSSCG00000030255	1.346596387	2.40E-10
ENSSSCG00000006777	1.345938421	0.000464307
ENSSSCG00000039314	1.345848915	2.00E-13
ENSSSCG00000003809	1.345425158	3.98E-12
ENSSSCG00000011893	1.345076652	0.001289287
ENSSSCG00000006499	1.344988905	0.000205291
ENSSSCG00000031530	1.344840421	1.40E-05
ENSSSCG00000024596	1.344750836	1.31E-09
ENSSSCG00000038700	1.344542689	0.001521033
ENSSSCG00000023666	1.344338928	2.98E-07
ENSSSCG00000006065	1.343758956	0.00112931
ENSSSCG00000037144	1.343739242	0.000515562
ENSSSCG00000012262	1.343726502	8.90E-06
ENSSSCG00000042487	1.343483785	8.13E-06
ENSSSCG00000004368	1.342953433	4.46E-09
ENSSSCG00000000769	1.342432915	1.13E-10

ENSSSCG00000008546	1.342218799	8.37E-10
ENSSSCG00000004374	1.3420946	2.37E-07
ENSSSCG00000011859	1.341931854	1.31E-06
ENSSSCG00000010248	1.340894236	3.01E-09
ENSSSCG00000005383	1.340752646	2.33E-07
ENSSSCG00000027526	1.340100268	5.61E-05
ENSSSCG00000009557	1.340009044	3.28E-05
ENSSSCG00000049577	1.339856585	8.07E-07
ENSSSCG00000004943	1.339653379	1.72E-08
ENSSSCG00000002505	1.339471056	0.000329687
ENSSSCG00000004358	1.339432984	1.08E-05
ENSSSCG00000038138	1.339109025	8.17E-07
ENSSSCG00000010550	1.338413998	6.03E-08
ENSSSCG00000026587	1.338319444	0.002566573
ENSSSCG00000010682	1.338097657	1.41E-11
ENSSSCG00000003520	1.338059193	4.11E-09
ENSSSCG00000020808	1.337929878	1.67E-08
ENSSSCG00000018015	1.337626393	0.002241275
ENSSSCG00000040180	1.337283602	0.000503401
ENSSSCG00000038718	1.33715173	0.000230238
ENSSSCG00000013475	1.337077575	1.05E-08
ENSSSCG00000009888	1.336092512	3.78E-09
ENSSSCG00000022801	1.335887327	2.59E-11
ENSSSCG00000008966	1.335041105	1.49E-14
ENSSSCG00000013435	1.334814162	3.30E-08
ENSSSCG00000026344	1.334357102	6.19E-06
ENSSSCG00000028606	1.333810024	9.90E-10
ENSSSCG00000032083	1.333405465	1.88E-05
ENSSSCG00000008230	1.333296906	1.84E-05
ENSSSCG00000037299	1.333118807	0.000310604
ENSSSCG00000027907	1.331493339	4.90E-06
ENSSSCG00000011426	1.331459634	1.77E-09
ENSSSCG00000017496	1.331263701	0.00032504
ENSSSCG00000003348	1.331060001	2.39E-09
ENSSSCG00000042700	1.330991635	0.009628305
ENSSSCG00000008374	1.330845198	3.54E-09
ENSSSCG00000046537	1.329644365	4.89E-05
ENSSSCG00000015424	1.329452903	2.60E-08
ENSSSCG00000016646	1.329310527	1.72E-09
ENSSSCG00000005072	1.327883502	6.45E-06
ENSSSCG00000000755	1.327379547	0.000242754
ENSSSCG00000017155	1.327295572	2.08E-07
ENSSSCG00000001463	1.326611636	3.96E-05
ENSSSCG00000010198	1.326399053	1.38E-08
ENSSSCG00000040080	1.326326539	0.004157856
ENSSSCG00000008747	1.326054443	5.35E-09
ENSSSCG00000016724	1.325848777	4.47E-05
ENSSSCG00000031456	1.324415321	1.83E-06
ENSSSCG00000005649	1.324367272	2.22E-06
ENSSSCG00000032999	1.324297158	3.35E-05
ENSSSCG00000020945	1.323912237	0.0003882
ENSSSCG00000000406	1.323196586	1.07E-06
ENSSSCG00000010700	1.322963464	7.62E-09
ENSSSCG00000010012	1.32267141	0.009965379
ENSSSCG00000010161	1.322473365	0.00054411
ENSSSCG00000017519	1.321919435	9.36E-05
ENSSSCG00000001510	1.321883774	3.17E-09
ENSSSCG00000035347	1.321873615	0.004517266

ENSSSCG00000036947	1.320946991	1.19E-09
ENSSSCG00000025593	1.320937447	9.92E-12
ENSSSCG00000035795	1.320715901	0.00550691
ENSSSCG00000010514	1.320438797	2.17E-11
ENSSSCG00000020964	1.320174487	1.14E-10
ENSSSCG00000048827	1.320168673	0.014811631
ENSSSCG00000010795	1.319919093	3.61E-06
ENSSSCG00000013118	1.319882575	1.33E-06
ENSSSCG00000006347	1.319175558	8.85E-07
ENSSSCG00000032909	1.318641034	9.22E-08
ENSSSCG00000028278	1.318378474	0.016530825
ENSSSCG00000006340	1.317075092	1.01E-07
ENSSSCG00000013647	1.316601073	9.27E-11
ENSSSCG00000013064	1.315375167	1.49E-05
ENSSSCG00000022401	1.315265227	0.00012926
ENSSSCG00000003816	1.315248053	1.79E-07
ENSSSCG00000023842	1.31500471	1.03E-06
ENSSSCG00000038130	1.314963799	1.10E-14
ENSSSCG00000002682	1.314889482	5.11E-10
ENSSSCG00000036695	1.3147549	6.36E-09
ENSSSCG00000035618	1.314186769	3.06E-08
ENSSSCG00000013176	1.313896545	1.91E-09
ENSSSCG00000024254	1.313837946	0.000753029
ENSSSCG00000012583	1.313829846	1.52E-08
ENSSSCG00000035442	1.313174465	1.63E-05
ENSSSCG00000034278	1.313165897	6.56E-09
ENSSSCG00000039243	1.312686318	9.45E-05
ENSSSCG00000030469	1.312476277	1.52E-09
ENSSSCG00000006761	1.312142008	2.03E-08
ENSSSCG00000005227	1.312055149	8.15E-09
ENSSSCG00000006554	1.311898487	4.57E-07
ENSSSCG00000029594	1.311634508	3.78E-13
ENSSSCG00000000386	1.311401631	3.70E-05
ENSSSCG00000003596	1.310774422	1.63E-08
ENSSSCG00000011363	1.310608711	1.03E-08
ENSSSCG00000015384	1.308283786	0.000201581
ENSSSCG00000034742	1.308220967	9.14E-08
ENSSSCG00000013735	1.307427676	3.58E-09
ENSSSCG00000015227	1.307320924	4.56E-11
ENSSSCG00000036716	1.307307332	0.002292624
ENSSSCG00000036676	1.30711639	0.000133644
ENSSSCG00000003385	1.306802584	0.000167073
ENSSSCG00000020671	1.306751416	8.82E-05
ENSSSCG00000010438	1.306420215	1.40E-09
ENSSSCG00000013043	1.306195673	0.001650511
ENSSSCG00000003106	1.305396935	0.001349347
ENSSSCG00000016609	1.304210355	0.010643997
ENSSSCG00000004382	1.30407037	9.18E-08
ENSSSCG00000006628	1.303365387	8.10E-06
ENSSSCG00000011458	1.302889283	1.21E-11
ENSSSCG00000024279	1.302617105	0.001096003
ENSSSCG00000008339	1.302572125	1.33E-08
ENSSSCG00000015345	1.302406322	0.000100148
ENSSSCG00000034216	1.302097325	3.43E-06
ENSSSCG00000013731	1.30190914	4.41E-05
ENSSSCG00000014540	1.301598394	0.000261811
ENSSSCG00000010575	1.301015759	1.10E-09
ENSSSCG00000031664	1.300878815	5.57E-06

ENSSSCG00000038826	1.300789988	0.000311106
ENSSSCG00000003867	1.300651489	6.00E-08
ENSSSCG00000038535	1.300547077	1.24E-06
ENSSSCG00000013352	1.300354196	2.29E-08
ENSSSCG00000000611	1.30029075	1.50E-09
ENSSSCG00000009047	1.30011332	8.64E-07
ENSSSCG00000003849	1.299703618	0.000123263
ENSSSCG00000015793	1.298400203	3.14E-10
ENSSSCG00000004237	1.297878807	3.85E-06
ENSSSCG00000006083	1.297729818	1.80E-09
ENSSSCG00000001770	1.29700884	4.27E-05
ENSSSCG00000011214	1.29627405	8.41E-09
ENSSSCG00000006235	1.29580245	2.88E-07
ENSSSCG00000009084	1.29518281	2.13E-08
ENSSSCG00000013223	1.295018152	6.01E-08
ENSSSCG00000035101	1.294049054	0.000538174
ENSSSCG00000035392	1.293454063	0.006937913
ENSSSCG00000045044	1.292894218	0.009982319
ENSSSCG00000014792	1.292798514	5.04E-08
ENSSSCG00000021408	1.292564127	5.07E-09
ENSSSCG00000036096	1.292270698	0.000596488
ENSSSCG00000028465	1.29206333	3.59E-10
ENSSSCG00000013661	1.291591409	7.93E-05
ENSSSCG00000028509	1.291486775	1.54E-06
ENSSSCG00000017744	1.290927615	4.52E-07
ENSSSCG00000035238	1.290883822	1.11E-07
ENSSSCG00000027480	1.290502228	1.84E-10
ENSSSCG00000039265	1.290168575	8.87E-05
ENSSSCG00000004668	1.290111726	0.000214351
ENSSSCG00000026748	1.289415236	1.33E-11
ENSSSCG00000030578	1.28933208	7.43E-05
ENSSSCG00000003801	1.287734152	0.00448321
ENSSSCG00000022507	1.287700925	8.93E-06
ENSSSCG00000017613	1.287518558	0.005214953
ENSSSCG00000026001	1.287493067	2.06E-10
ENSSSCG00000034860	1.286341561	0.000659282
ENSSSCG00000036385	1.286287216	6.61E-05
ENSSSCG00000032472	1.286178366	9.70E-07
ENSSSCG00000049011	1.286169406	6.73E-07
ENSSSCG00000036956	1.286073252	9.83E-10
ENSSSCG00000020870	1.285008371	1.91E-07
ENSSSCG00000014376	1.284450029	6.04E-07
ENSSSCG00000031893	1.28381398	3.81E-10
ENSSSCG00000002449	1.283574519	0.007957061
ENSSSCG00000000361	1.28340929	0.000197886
ENSSSCG00000002867	1.282939181	1.36E-08
ENSSSCG00000013066	1.282889962	3.97E-12
ENSSSCG00000002145	1.28217329	2.57E-06
ENSSSCG00000033402	1.281899713	2.33E-06
ENSSSCG00000010822	1.280939187	1.72E-09
ENSSSCG00000003402	1.280656037	4.02E-08
ENSSSCG00000006527	1.280507048	0.000191369
ENSSSCG00000015785	1.28035642	1.63E-05
ENSSSCG00000014068	1.279842217	7.04E-07
ENSSSCG00000004748	1.278960077	7.66E-07
ENSSSCG00000002458	1.278882955	4.83E-06
ENSSSCG00000010686	1.278810614	6.39E-05
ENSSSCG00000028855	1.276988041	2.97E-09

ENSSSCG00000037238	1.276736151	6.24E-06
ENSSSCG00000033526	1.276484841	7.97E-06
ENSSSCG00000008693	1.275318601	5.24E-09
ENSSSCG00000017067	1.275011467	7.71E-09
ENSSSCG00000006538	1.274636658	1.95E-06
ENSSSCG00000014994	1.273851749	1.73E-07
ENSSSCG00000017796	1.27342808	1.08E-06
ENSSSCG00000024562	1.273394751	2.68E-13
ENSSSCG00000033626	1.273377202	6.48E-05
ENSSSCG00000038579	1.273348473	0.000483504
ENSSSCG00000002530	1.273163411	0.000234915
ENSSSCG00000034952	1.273154381	1.28E-06
ENSSSCG00000017755	1.273130667	1.08E-07
ENSSSCG00000031847	1.27284436	6.37E-10
ENSSSCG00000008434	1.27270259	9.80E-06
ENSSSCG00000000116	1.271188565	0.001915759
ENSSSCG00000006559	1.269720853	0.004146333
ENSSSCG00000011739	1.26967752	1.77E-06
ENSSSCG00000037445	1.269139237	0.001491947
ENSSSCG00000008571	1.268599881	5.31E-10
ENSSSCG00000034049	1.268342933	3.05E-05
ENSSSCG00000042480	1.268098231	0.011236822
ENSSSCG00000002768	1.267631396	5.44E-07
ENSSSCG00000035061	1.266498245	0.001578675
ENSSSCG00000006892	1.266470776	1.69E-08
ENSSSCG00000000732	1.2664462	1.58E-09
ENSSSCG00000000994	1.265968048	2.73E-06
ENSSSCG00000039336	1.264453058	4.45E-06
ENSSSCG00000002907	1.264042765	0.001561954
ENSSSCG00000000599	1.263453528	2.67E-06
ENSSSCG00000038987	1.262649382	3.01E-05
ENSSSCG00000017489	1.262400017	4.07E-12
ENSSSCG00000031152	1.262068604	4.97E-07
ENSSSCG00000040857	1.261569296	6.61E-05
ENSSSCG00000024193	1.260981914	7.98E-05
ENSSSCG00000029626	1.260965462	0.000902441
ENSSSCG00000038777	1.260651097	0.004434262
ENSSSCG00000007308	1.260152538	0.004621486
ENSSSCG00000008226	1.26000289	2.57E-07
ENSSSCG00000016750	1.259990455	2.26E-06
ENSSSCG00000039830	1.259259702	4.75E-07
ENSSSCG00000024144	1.259253335	0.000701171
ENSSSCG00000039331	1.258986018	3.53E-05
ENSSSCG00000048578	1.258502559	0.013540875
ENSSSCG00000014214	1.257941386	0.005006534
ENSSSCG00000001866	1.257692498	0.000131763
ENSSSCG00000009009	1.257512022	0.000992955
ENSSSCG00000006219	1.257075218	7.39E-06
ENSSSCG00000012968	1.256821754	0.002611548
ENSSSCG00000003973	1.256640278	1.64E-12
ENSSSCG00000013992	1.256278947	1.67E-07
ENSSSCG00000001756	1.255787995	1.23E-06
ENSSSCG00000008756	1.255590752	1.15E-08
ENSSSCG00000027628	1.255100235	2.43E-09
ENSSSCG00000005012	1.254289662	2.39E-09
ENSSSCG00000000547	1.254008405	2.91E-06
ENSSSCG00000012880	1.253277138	6.77E-07
ENSSSCG00000017906	1.25285972	1.03E-06

ENSSSCG00000004657	1.252747068	2.13E-05
ENSSSCG00000001372	1.252596911	3.13E-05
ENSSSCG00000006140	1.252501916	0.010630398
ENSSSCG00000040528	1.252265031	3.67E-07
ENSSSCG00000028788	1.251693041	1.61E-05
ENSSSCG00000035059	1.25137389	5.77E-10
ENSSSCG00000016104	1.250626005	0.000545841
ENSSSCG00000017561	1.250494002	3.78E-07
ENSSSCG00000038237	1.250229765	1.87E-07
ENSSSCG00000048111	1.250181521	0.000527662
ENSSSCG00000015050	1.24994477	3.83E-09
ENSSSCG00000006475	1.24963815	8.48E-05
ENSSSCG00000007356	1.249354506	3.36E-05
ENSSSCG00000015108	1.249053416	4.90E-05
ENSSSCG00000010589	1.247840892	2.31E-07
ENSSSCG00000029547	1.247775389	1.58E-06
ENSSSCG00000016715	1.247548387	1.46E-07
ENSSSCG00000041364	1.247141929	1.76E-08
ENSSSCG00000011710	1.246980682	3.40E-10
ENSSSCG00000003189	1.246409069	1.56E-05
ENSSSCG00000013913	1.24594527	7.54E-10
ENSSSCG00000031174	1.245831033	6.99E-05
ENSSSCG00000005976	1.245784152	0.00052202
ENSSSCG00000025830	1.245255254	2.24E-11
ENSSSCG00000012943	1.244749246	0.000161884
ENSSSCG00000017733	1.244363586	1.74E-07
ENSSSCG00000021310	1.243741618	0.000324357
ENSSSCG00000028317	1.24351779	0.000393557
ENSSSCG00000020842	1.243469047	8.24E-06
ENSSSCG00000011003	1.24346649	0.001433464
ENSSSCG00000013551	1.243445785	1.50E-05
ENSSSCG00000026092	1.242648364	1.61E-09
ENSSSCG00000024776	1.241933009	1.06E-06
ENSSSCG00000011572	1.241608853	1.10E-08
ENSSSCG00000006848	1.241057756	2.43E-06
ENSSSCG00000014406	1.24094541	1.01E-05
ENSSSCG00000004055	1.240903979	0.001286624
ENSSSCG00000038967	1.240753229	1.10E-07
ENSSSCG00000006156	1.240323708	5.96E-07
ENSSSCG00000013473	1.24028447	1.72E-08
ENSSSCG00000008587	1.240026694	0.000273903
ENSSSCG00000028420	1.239906507	1.10E-07
ENSSSCG00000012325	1.239308974	1.81E-11
ENSSSCG00000013282	1.238838973	4.33E-08
ENSSSCG00000000133	1.238763548	0.002019393
ENSSSCG00000039555	1.2383683	1.24E-10
ENSSSCG00000017799	1.238037342	7.01E-07
ENSSSCG00000037602	1.237363733	1.25E-05
ENSSSCG00000037046	1.23722501	2.23E-06
ENSSSCG00000004518	1.237060129	0.001132662
ENSSSCG00000025060	1.236487086	1.27E-07
ENSSSCG00000017414	1.236294402	5.80E-11
ENSSSCG00000037426	1.235707889	0.000287684
ENSSSCG00000007854	1.234752913	0.000270862
ENSSSCG00000034302	1.234568772	2.22E-09
ENSSSCG00000012280	1.234547017	0.001022124
ENSSSCG00000023267	1.234410777	0.000528347
ENSSSCG00000000739	1.23419419	8.47E-07

ENSSSCG00000040183	1.234141874	8.68E-05
ENSSSCG00000003612	1.233971036	1.27E-05
ENSSSCG00000010956	1.233939808	6.57E-08
ENSSSCG00000010999	1.233790279	7.00E-10
ENSSSCG00000027701	1.233697701	1.75E-05
ENSSSCG00000028924	1.233563262	0.000390642
ENSSSCG00000024655	1.233223524	7.34E-06
ENSSSCG00000023362	1.233091124	1.35E-08
ENSSSCG00000034059	1.232986511	0.000161689
ENSSSCG00000035204	1.232520482	0.017058222
ENSSSCG00000010759	1.231838777	1.43E-05
ENSSSCG00000016045	1.231801996	2.10E-06
ENSSSCG00000028981	1.231311641	1.40E-06
ENSSSCG00000000024	1.230793761	0.000245779
ENSSSCG00000031882	1.230611957	1.22E-07
ENSSSCG00000004622	1.230508933	3.41E-06
ENSSSCG00000040373	1.229346461	1.39E-06
ENSSSCG00000045860	1.228990372	0.001648132
ENSSSCG00000031612	1.227723306	3.82E-06
ENSSSCG00000039885	1.227556868	5.28E-07
ENSSSCG00000040642	1.227464878	1.60E-07
ENSSSCG00000009137	1.227351555	1.74E-06
ENSSSCG00000045508	1.227313716	2.04E-06
ENSSSCG00000001476	1.226282586	0.000379497
ENSSSCG00000011157	1.22628037	1.09E-11
ENSSSCG00000005241	1.22617762	3.93E-05
ENSSSCG00000025675	1.225535378	5.34E-06
ENSSSCG00000000841	1.225316479	2.23E-09
ENSSSCG00000017671	1.225093734	0.000103931
ENSSSCG00000016140	1.22481702	0.000887216
ENSSSCG00000021180	1.223982152	0.000225559
ENSSSCG00000014058	1.223958422	1.49E-05
ENSSSCG00000008270	1.223344127	1.96E-09
ENSSSCG00000009766	1.223052498	2.36E-08
ENSSSCG00000004233	1.222780687	5.07E-05
ENSSSCG00000012902	1.222715898	0.000405397
ENSSSCG00000012772	1.222634931	0.00074081
ENSSSCG00000036192	1.222463493	4.50E-06
ENSSSCG00000031083	1.222200726	1.26E-11
ENSSSCG00000016044	1.222109141	2.87E-06
ENSSSCG00000008900	1.222042727	1.45E-07
ENSSSCG00000016738	1.22193673	1.33E-05
ENSSSCG00000050998	1.221882887	0.000273266
ENSSSCG00000033033	1.221834586	0.001479457
ENSSSCG00000009395	1.221767814	2.22E-07
ENSSSCG00000035836	1.221375476	0.001746363
ENSSSCG00000009185	1.220937564	4.92E-06
ENSSSCG00000039370	1.22085948	2.78E-06
ENSSSCG00000012926	1.22039131	0.008530501
ENSSSCG00000036614	1.220243051	0.000213198
ENSSSCG00000026713	1.219618245	0.005296153
ENSSSCG00000027016	1.218671293	5.22E-05
ENSSSCG00000011876	1.21853382	6.68E-08
ENSSSCG00000009840	1.217461048	3.93E-09
ENSSSCG00000002307	1.217404853	2.89E-05
ENSSSCG00000037513	1.216987519	6.33E-09
ENSSSCG00000005367	1.21662059	0.003531331
ENSSSCG00000006619	1.216596777	8.68E-07

ENSSSCG00000009872	1.216547214	4.90E-07
ENSSSCG00000003216	1.21580409	7.44E-07
ENSSSCG00000032833	1.214736677	0.000942951
ENSSSCG00000036014	1.214630062	0.000302241
ENSSSCG00000000657	1.214555635	4.11E-05
ENSSSCG00000021494	1.214432796	9.32E-07
ENSSSCG00000002908	1.214238565	1.93E-05
ENSSSCG00000030681	1.214027665	3.87E-06
ENSSSCG00000027778	1.213821626	7.82E-07
ENSSSCG00000003152	1.213425924	1.38E-05
ENSSSCG00000008641	1.213341656	4.19E-05
ENSSSCG00000006287	1.213046792	1.07E-05
ENSSSCG00000035945	1.212862523	0.000160976
ENSSSCG00000003918	1.212819704	4.51E-05
ENSSSCG00000037439	1.212707998	0.001255533
ENSSSCG00000011813	1.212642421	1.32E-08
ENSSSCG00000006730	1.212607085	5.28E-06
ENSSSCG00000006380	1.212528567	5.54E-05
ENSSSCG00000014905	1.212317357	3.14E-06
ENSSSCG00000000694	1.212212822	1.07E-05
ENSSSCG00000050093	1.212165139	1.16E-05
ENSSSCG00000016714	1.212028601	7.30E-05
ENSSSCG00000003926	1.211780636	1.15E-07
ENSSSCG00000010904	1.211114035	1.33E-09
ENSSSCG00000036115	1.211001236	2.50E-09
ENSSSCG00000013509	1.210814928	3.98E-11
ENSSSCG00000001658	1.210363298	0.000187373
ENSSSCG00000004047	1.21004681	9.30E-05
ENSSSCG00000009129	1.209912622	2.75E-10
ENSSSCG00000026731	1.209297931	2.04E-05
ENSSSCG00000048091	1.209257832	0.000125335
ENSSSCG00000007287	1.209012235	0.000141984
ENSSSCG00000031144	1.207488911	0.000402261
ENSSSCG00000027973	1.206446694	7.52E-10
ENSSSCG00000003187	1.20625078	5.34E-07
ENSSSCG00000034014	1.206076819	0.001204286
ENSSSCG00000011764	1.205853784	0.000260026
ENSSSCG00000034019	1.205824568	0.002428668
ENSSSCG00000007692	1.205601999	1.73E-07
ENSSSCG00000013302	1.205519604	5.81E-11
ENSSSCG00000005787	1.2042059	6.12E-09
ENSSSCG00000016846	1.2041905	3.98E-08
ENSSSCG00000046255	1.203767487	4.91E-09
ENSSSCG00000028202	1.203719664	5.33E-08
ENSSSCG00000011494	1.203662293	1.52E-07
ENSSSCG00000009072	1.203251116	3.57E-07
ENSSSCG00000034039	1.202571477	1.81E-07
ENSSSCG00000008816	1.202478938	8.10E-07
ENSSSCG00000013005	1.202457069	2.48E-05
ENSSSCG00000002243	1.20226275	0.007510227
ENSSSCG00000011950	1.201374008	5.27E-07
ENSSSCG00000023793	1.201368294	4.30E-06
ENSSSCG00000040344	1.200940105	8.65E-08
ENSSSCG00000038398	1.200165676	1.11E-05
ENSSSCG00000017091	1.200000523	8.05E-10
ENSSSCG00000008677	1.19968519	4.79E-06
ENSSSCG00000024983	1.199612104	1.57E-05
ENSSSCG00000024970	1.199536066	0.007317653

ENSSSCG00000039703	1.1992499	0.000152895
ENSSSCG00000009978	1.199138341	2.52E-08
ENSSSCG00000009043	1.19872252	2.99E-07
ENSSSCG00000025766	1.198265476	0.00058271
ENSSSCG00000037755	1.198038824	0.000453732
ENSSSCG00000002036	1.197861046	0.004260955
ENSSSCG00000038260	1.197788102	0.00576713
ENSSSCG00000008772	1.197291663	3.18E-05
ENSSSCG00000022559	1.196359745	3.53E-06
ENSSSCG00000026959	1.196328912	1.61E-07
ENSSSCG00000028638	1.195348619	1.81E-09
ENSSSCG00000023677	1.195310537	0.000824636
ENSSSCG00000016986	1.195224723	1.59E-05
ENSSSCG00000017745	1.195142101	4.07E-10
ENSSSCG00000010240	1.194790565	7.55E-05
ENSSSCG00000002020	1.194732381	4.57E-06
ENSSSCG00000036960	1.194419392	0.00069661
ENSSSCG00000028600	1.194170098	3.59E-07
ENSSSCG00000007586	1.194163373	1.46E-05
ENSSSCG00000034105	1.193879016	1.59E-09
ENSSSCG00000027072	1.193420852	0.000821378
ENSSSCG00000005507	1.192770808	0.000397849
ENSSSCG00000038708	1.192083485	4.20E-06
ENSSSCG00000030510	1.192040278	0.000328
ENSSSCG00000007383	1.191771507	1.25E-05
ENSSSCG00000016207	1.191711136	7.46E-10
ENSSSCG00000012622	1.191645533	0.000120625
ENSSSCG00000040103	1.191352654	0.000594712
ENSSSCG00000023715	1.191149615	2.59E-06
ENSSSCG00000007458	1.190333535	0.002872928
ENSSSCG00000021210	1.189859802	0.000332407
ENSSSCG00000039468	1.189856006	2.75E-09
ENSSSCG00000002410	1.18935093	0.00120602
ENSSSCG00000007767	1.188965713	2.16E-05
ENSSSCG00000006263	1.188908993	3.16E-06
ENSSSCG00000005436	1.188784074	1.21E-07
ENSSSCG00000044051	1.188197408	0.016829665
ENSSSCG00000034994	1.188091911	2.79E-05
ENSSSCG00000009903	1.187952363	3.18E-05
ENSSSCG00000006309	1.187869562	8.43E-08
ENSSSCG00000016732	1.187616888	5.98E-05
ENSSSCG00000031698	1.187364968	0.000438698
ENSSSCG00000000119	1.187348239	1.03E-10
ENSSSCG00000042107	1.187187659	0.001402085
ENSSSCG00000008638	1.187125613	1.00E-08
ENSSSCG00000008058	1.187020393	2.97E-06
ENSSSCG00000007736	1.186940404	6.08E-11
ENSSSCG00000015535	1.186830199	4.21E-07
ENSSSCG00000021663	1.186388878	1.21E-05
ENSSSCG00000023762	1.185929397	1.28E-05
ENSSSCG00000004983	1.18582725	1.69E-06
ENSSSCG00000017385	1.185638691	1.53E-09
ENSSSCG00000013030	1.185497726	0.000710617
ENSSSCG00000008773	1.185470574	1.75E-06
ENSSSCG00000010801	1.184371103	5.72E-07
ENSSSCG00000016173	1.184229012	0.000259249
ENSSSCG00000017008	1.184041876	0.000153971
ENSSSCG00000024481	1.183689529	4.24E-05

ENSSSCG00000006735	1.18300656	1.11E-07
ENSSSCG00000003091	1.182903055	4.25E-06
ENSSSCG00000025478	1.182799818	0.000454527
ENSSSCG00000001852	1.182499336	2.28E-07
ENSSSCG00000017232	1.182431637	8.37E-07
ENSSSCG00000039673	1.182200305	0.003251778
ENSSSCG00000012250	1.181924085	4.41E-06
ENSSSCG00000003780	1.181346	1.05E-07
ENSSSCG00000012063	1.181195695	1.08E-06
ENSSSCG00000032223	1.181024	6.05E-07
ENSSSCG00000017514	1.18099967	1.18E-05
ENSSSCG00000028157	1.180806567	2.96E-06
ENSSSCG00000005510	1.180069259	1.04E-09
ENSSSCG00000031838	1.179284616	0.003505321
ENSSSCG00000029519	1.178976115	7.56E-05
ENSSSCG00000026229	1.178972355	0.000958394
ENSSSCG00000045776	1.178691046	0.003990452
ENSSSCG00000033987	1.178509855	6.42E-05
ENSSSCG00000010984	1.177978584	0.000174677
ENSSSCG00000012486	1.177640348	6.57E-08
ENSSSCG00000011516	1.177315383	3.95E-07
ENSSSCG00000012686	1.177104599	1.05E-07
ENSSSCG00000014538	1.176935205	2.02E-10
ENSSSCG00000008126	1.176861926	0.000700179
ENSSSCG00000026194	1.17682916	1.30E-09
ENSSSCG00000010457	1.176586461	1.60E-06
ENSSSCG00000031103	1.175875888	2.56E-05
ENSSSCG00000033301	1.175704163	0.002772974
ENSSSCG00000029300	1.175185791	0.002447493
ENSSSCG00000016079	1.174887294	1.35E-05
ENSSSCG00000012878	1.174745544	0.006034184
ENSSSCG00000007314	1.173317422	2.12E-08
ENSSSCG00000029518	1.173259842	3.12E-08
ENSSSCG00000004044	1.17324579	0.000773913
ENSSSCG00000026761	1.17324218	6.61E-07
ENSSSCG00000023173	1.172646632	4.47E-06
ENSSSCG00000035209	1.17189025	1.07E-05
ENSSSCG00000032073	1.171557009	0.000636723
ENSSSCG00000009440	1.171289227	7.48E-06
ENSSSCG00000036307	1.171009841	2.04E-06
ENSSSCG00000005478	1.170463009	4.06E-10
ENSSSCG00000005679	1.170252947	1.49E-07
ENSSSCG00000030957	1.170120046	4.64E-05
ENSSSCG00000042874	1.169929584	3.68E-06
ENSSSCG00000024384	1.169838903	2.21E-08
ENSSSCG00000010648	1.169369308	0.005282308
ENSSSCG00000036828	1.169277822	6.59E-06
ENSSSCG00000010755	1.169208372	4.20E-09
ENSSSCG00000016010	1.168718932	0.0071827
ENSSSCG00000033089	1.168536375	1.21E-08
ENSSSCG00000012886	1.168401931	1.41E-07
ENSSSCG00000016564	1.168287055	1.65E-07
ENSSSCG00000040294	1.168198952	0.000497609
ENSSSCG00000011239	1.168094463	0.000151778
ENSSSCG00000017378	1.167438596	1.21E-09
ENSSSCG00000006678	1.167345612	5.81E-06
ENSSSCG00000001577	1.167130627	9.50E-08
ENSSSCG00000007076	1.167122107	1.99E-05

ENSSSCG00000017886	1.167082021	6.92E-08
ENSSSCG00000012014	1.166530243	8.98E-06
ENSSSCG00000001971	1.166480383	0.014582823
ENSSSCG00000007004	1.166454404	3.40E-09
ENSSSCG00000005651	1.166264131	2.01E-05
ENSSSCG00000001430	1.16614731	2.25E-12
ENSSSCG00000010504	1.166027953	1.29E-10
ENSSSCG00000002008	1.165053039	5.47E-09
ENSSSCG00000021250	1.164994954	1.81E-09
ENSSSCG00000011215	1.16489981	3.45E-06
ENSSSCG00000013297	1.164785849	2.64E-07
ENSSSCG00000003566	1.164244783	5.99E-07
ENSSSCG00000009345	1.164240235	3.02E-07
ENSSSCG00000007896	1.163883381	1.57E-10
ENSSSCG00000024242	1.163676351	3.85E-08
ENSSSCG00000029918	1.163466809	4.81E-06
ENSSSCG00000002622	1.16316024	0.00091581
ENSSSCG00000035747	1.162347182	9.91E-05
ENSSSCG00000013498	1.161784174	0.00269001
ENSSSCG00000009879	1.161050199	6.70E-08
ENSSSCG00000012534	1.16097061	1.44E-07
ENSSSCG00000035410	1.160957958	4.74E-10
ENSSSCG00000002776	1.160852262	1.76E-05
ENSSSCG00000038506	1.160334814	1.47E-09
ENSSSCG00000031212	1.16012495	0.00055747
ENSSSCG00000045223	1.159895383	0.012337243
ENSSSCG00000022900	1.159066106	3.53E-09
ENSSSCG00000000105	1.158968628	0.000168378
ENSSSCG00000017876	1.158921915	6.32E-08
ENSSSCG00000023118	1.158385997	2.46E-05
ENSSSCG00000004020	1.158361309	8.78E-05
ENSSSCG00000003377	1.157583845	2.72E-06
ENSSSCG00000037516	1.156460433	0.00415382
ENSSSCG00000016988	1.156304152	1.05E-06
ENSSSCG00000009412	1.156022885	6.43E-08
ENSSSCG00000000798	1.155953384	4.81E-05
ENSSSCG00000011316	1.155868722	7.10E-05
ENSSSCG00000016100	1.15509186	0.000932434
ENSSSCG00000003707	1.154995496	0.000159461
ENSSSCG00000013622	1.154190124	0.001328464
ENSSSCG00000002353	1.154184207	0.0102433
ENSSSCG00000000553	1.154002677	7.67E-05
ENSSSCG00000031706	1.153855882	0.012734949
ENSSSCG00000005174	1.153712476	1.88E-05
ENSSSCG00000015498	1.153682847	0.009147295
ENSSSCG00000038950	1.153634451	9.07E-07
ENSSSCG00000004370	1.153376481	7.86E-09
ENSSSCG00000025028	1.152982085	4.53E-06
ENSSSCG00000001780	1.152244133	0.000284474
ENSSSCG00000039139	1.152014864	2.72E-06
ENSSSCG00000003288	1.151462681	0.000735365
ENSSSCG00000032686	1.150977992	1.54E-07
ENSSSCG00000002000	1.150487618	2.19E-09
ENSSSCG00000036136	1.150113787	4.31E-09
ENSSSCG00000002722	1.150083865	4.57E-10
ENSSSCG00000035256	1.149358139	6.96E-10
ENSSSCG00000039425	1.149201637	0.001648619
ENSSSCG00000009679	1.149178548	0.007677126

ENSSSCG00000008217	1.149100726	1.71E-08
ENSSSCG00000037481	1.148891018	2.78E-05
ENSSSCG00000028856	1.148113123	1.00E-07
ENSSSCG00000026055	1.148081146	0.000203944
ENSSSCG00000038902	1.147085691	0.001181978
ENSSSCG00000001392	1.146878246	1.41E-07
ENSSSCG00000045485	1.145951355	0.006468316
ENSSSCG00000037307	1.145195132	6.44E-07
ENSSSCG00000025651	1.145058285	7.55E-06
ENSSSCG00000022396	1.144644738	1.30E-05
ENSSSCG00000034927	1.144482863	9.02E-09
ENSSSCG00000006333	1.143895743	1.14E-05
ENSSSCG00000040961	1.143628671	0.006227019
ENSSSCG00000011296	1.143514633	0.000439006
ENSSSCG00000034632	1.143421334	0.001851745
ENSSSCG00000004578	1.143397594	4.68E-06
ENSSSCG00000021411	1.143201968	0.000558422
ENSSSCG00000033500	1.143182101	0.002260343
ENSSSCG00000033593	1.143039328	2.13E-07
ENSSSCG00000039230	1.142325382	6.07E-05
ENSSSCG00000017495	1.142271191	0.002507989
ENSSSCG00000025355	1.141921655	1.69E-05
ENSSSCG00000032942	1.141827882	4.65E-08
ENSSSCG00000014100	1.141799192	0.00295135
ENSSSCG00000006063	1.141321281	0.000151367
ENSSSCG00000006098	1.141084994	4.99E-07
ENSSSCG00000013592	1.141055449	1.73E-07
ENSSSCG00000001219	1.140807649	9.24E-08
ENSSSCG00000031877	1.140800031	0.000326183
ENSSSCG00000013758	1.140340795	0.012984951
ENSSSCG00000004548	1.138364842	3.63E-06
ENSSSCG00000006192	1.138293744	0.000211896
ENSSSCG00000017591	1.137644696	0.004305702
ENSSSCG00000004765	1.137291691	2.47E-08
ENSSSCG00000004446	1.137105322	0.001812049
ENSSSCG00000050388	1.136887158	8.88E-05
ENSSSCG00000003439	1.136741019	5.83E-06
ENSSSCG00000009777	1.136629062	1.20E-08
ENSSSCG00000038178	1.136538246	0.018030574
ENSSSCG00000005423	1.136085967	0.003550585
ENSSSCG00000036056	1.135967629	3.03E-07
ENSSSCG00000037575	1.135841001	0.002957083
ENSSSCG00000042630	1.135312762	5.67E-06
ENSSSCG00000035293	1.135291643	2.09E-05
ENSSSCG00000011435	1.134603217	0.000234747
ENSSSCG00000038772	1.134108175	1.79E-05
ENSSSCG00000020777	1.133654691	2.06E-08
ENSSSCG00000010121	1.133632217	7.38E-06
ENSSSCG00000013305	1.133069465	6.33E-07
ENSSSCG00000005590	1.132856748	0.000657025
ENSSSCG00000011881	1.13275603	9.98E-06
ENSSSCG00000008559	1.132433255	8.65E-11
ENSSSCG00000000890	1.132225089	2.72E-06
ENSSSCG00000025248	1.132062118	4.80E-07
ENSSSCG00000007733	1.13173448	6.38E-05
ENSSSCG00000031433	1.131544691	1.99E-05
ENSSSCG00000008098	1.131134828	1.92E-06
ENSSSCG00000023719	1.130553165	3.89E-05

ENSSSCG00000017379	1.13050273	0.000646146
ENSSSCG00000005449	1.129044342	0.000508096
ENSSSCG00000002762	1.128886102	9.66E-09
ENSSSCG00000017386	1.127772295	1.75E-07
ENSSSCG00000016983	1.127624491	1.08E-06
ENSSSCG00000040089	1.127587524	0.004684486
ENSSSCG00000013028	1.127462723	1.04E-05
ENSSSCG00000013349	1.12745017	7.58E-06
ENSSSCG00000012837	1.127448753	0.000303312
ENSSSCG00000004331	1.127379602	0.001965185
ENSSSCG00000028414	1.127276561	0.003231247
ENSSSCG00000013861	1.127187102	7.91E-08
ENSSSCG00000028066	1.127107117	0.000474328
ENSSSCG00000022080	1.127084608	0.000110979
ENSSSCG00000038453	1.12678665	0.000473689
ENSSSCG00000011340	1.126718172	4.14E-06
ENSSSCG00000031876	1.126667031	0.000873497
ENSSSCG00000015931	1.126428738	0.001893945
ENSSSCG00000013886	1.125737778	7.65E-06
ENSSSCG00000038036	1.125682601	0.012934504
ENSSSCG00000009617	1.124456048	0.000286683
ENSSSCG00000036887	1.123885714	5.13E-06
ENSSSCG00000037568	1.123883207	1.90E-05
ENSSSCG00000016018	1.123563245	0.000368902
ENSSSCG00000002143	1.123022854	1.91E-07
ENSSSCG00000009061	1.122912492	1.09E-07
ENSSSCG00000031128	1.12266025	9.88E-05
ENSSSCG00000009405	1.122613315	2.03E-05
ENSSSCG00000021325	1.122287135	3.08E-08
ENSSSCG00000045531	1.122111085	0.002644902
ENSSSCG00000014965	1.121758591	2.00E-06
ENSSSCG00000003169	1.121586383	0.000896401
ENSSSCG00000038968	1.121537692	3.39E-07
ENSSSCG00000011081	1.121326148	7.06E-06
ENSSSCG00000016893	1.121259993	0.006710907
ENSSSCG00000029097	1.121070448	8.63E-07
ENSSSCG00000014141	1.120734738	0.003233888
ENSSSCG00000001024	1.119225242	1.52E-09
ENSSSCG00000028504	1.119152578	6.47E-05
ENSSSCG00000040396	1.11881744	0.004701937
ENSSSCG00000038172	1.118657718	0.00037995
ENSSSCG00000028249	1.118378494	4.70E-09
ENSSSCG00000012979	1.117703791	0.007817753
ENSSSCG00000024126	1.117406146	3.08E-08
ENSSSCG00000002357	1.117347637	9.04E-06
ENSSSCG00000039295	1.117190329	0.000324102
ENSSSCG00000012840	1.117119973	0.001606975
ENSSSCG00000006209	1.116177119	4.38E-07
ENSSSCG00000033030	1.115895786	0.000575845
ENSSSCG00000022300	1.115845102	1.51E-08
ENSSSCG00000022128	1.115415587	4.02E-06
ENSSSCG00000006523	1.115047279	0.001780798
ENSSSCG00000003628	1.114511566	2.06E-05
ENSSSCG00000003085	1.114059193	2.87E-08
ENSSSCG00000005361	1.11371173	0.000524771
ENSSSCG00000040833	1.113397471	0.00034752
ENSSSCG00000015231	1.113109382	1.84E-05
ENSSSCG00000012427	1.112767607	0.00015996

ENSSSCG00000004311	1.112438186	1.28E-06
ENSSSCG00000011518	1.111961135	6.41E-06
ENSSSCG00000030478	1.111829985	0.000144974
ENSSSCG00000017308	1.111564173	1.71E-05
ENSSSCG00000037053	1.111189427	0.001566091
ENSSSCG00000000874	1.110791237	0.002217632
ENSSSCG00000022719	1.11051504	0.007698007
ENSSSCG00000009853	1.110329188	2.49E-09
ENSSSCG00000036476	1.110219187	2.46E-06
ENSSSCG00000008213	1.110125904	0.000661796
ENSSSCG00000029174	1.109589611	0.001722149
ENSSSCG00000014091	1.109417793	0.006513697
ENSSSCG00000027457	1.109032097	2.60E-07
ENSSSCG00000021383	1.108436632	1.53E-05
ENSSSCG00000020868	1.108165469	2.40E-06
ENSSSCG00000001810	1.108063552	1.18E-06
ENSSSCG00000003966	1.107746341	6.40E-07
ENSSSCG00000005969	1.107704454	1.17E-06
ENSSSCG00000038726	1.107452619	0.002312179
ENSSSCG00000049234	1.107434845	0.000134658
ENSSSCG00000034147	1.107180565	0.015211076
ENSSSCG00000012202	1.107117324	2.18E-07
ENSSSCG00000006237	1.107055176	3.23E-07
ENSSSCG00000033786	1.105871578	5.88E-05
ENSSSCG00000016053	1.105472542	0.003532502
ENSSSCG00000015999	1.105438885	0.002086427
ENSSSCG00000012890	1.105431746	2.18E-07
ENSSSCG00000028178	1.105336752	1.53E-07
ENSSSCG00000025454	1.105310106	1.24E-09
ENSSSCG00000017094	1.105288204	1.83E-08
ENSSSCG00000031271	1.105032212	3.01E-06
ENSSSCG00000015689	1.104960208	9.75E-06
ENSSSCG00000037264	1.104537973	1.53E-07
ENSSSCG00000017494	1.104529174	6.23E-07
ENSSSCG00000010465	1.104126098	4.73E-05
ENSSSCG00000028492	1.103653249	0.000895226
ENSSSCG00000014094	1.103582881	5.40E-07
ENSSSCG00000033687	1.103542801	0.000884434
ENSSSCG00000007342	1.103348873	5.55E-08
ENSSSCG00000016323	1.102488612	1.45E-05
ENSSSCG00000026268	1.10220049	0.007096519
ENSSSCG00000027272	1.102011341	4.28E-08
ENSSSCG00000011552	1.101483784	0.001288521
ENSSSCG00000003434	1.101004205	2.63E-05
ENSSSCG00000010521	1.100879681	0.000488937
ENSSSCG00000009240	1.100408889	0.000457981
ENSSSCG00000036595	1.100297986	0.001751786
ENSSSCG00000039201	1.099912419	2.54E-06
ENSSSCG00000017832	1.099691029	6.99E-05
ENSSSCG00000013048	1.099637505	0.000331346
ENSSSCG00000038394	1.09948596	8.51E-06
ENSSSCG00000011961	1.09857864	1.57E-06
ENSSSCG00000009226	1.098283709	0.000716728
ENSSSCG00000021040	1.098125725	1.23E-05
ENSSSCG00000000064	1.097909726	9.27E-10
ENSSSCG00000006061	1.097848191	4.51E-05
ENSSSCG00000017869	1.097314126	2.16E-09
ENSSSCG00000007064	1.096991293	0.000266484

ENSSSCG00000006137	1.096541281	0.000213515
ENSSSCG00000027426	1.096214579	4.98E-05
ENSSSCG00000005092	1.095788096	5.37E-05
ENSSSCG00000009612	1.094495995	0.001470018
ENSSSCG00000034151	1.09418835	0.000157561
ENSSSCG00000005215	1.093759342	6.38E-05
ENSSSCG00000009944	1.093593738	2.91E-07
ENSSSCG00000011741	1.093350305	0.001895797
ENSSSCG00000011309	1.093038402	2.56E-06
ENSSSCG00000025455	1.092270654	2.09E-06
ENSSSCG00000035182	1.092227594	2.50E-05
ENSSSCG00000022299	1.091565727	2.48E-05
ENSSSCG00000040621	1.091554142	3.16E-05
ENSSSCG00000029385	1.091272071	0.000107961
ENSSSCG00000008282	1.091245236	0.015567021
ENSSSCG00000010508	1.090654318	3.73E-05
ENSSSCG00000021440	1.089255278	0.000101002
ENSSSCG00000038042	1.088738143	4.46E-05
ENSSSCG00000000071	1.088647662	4.36E-07
ENSSSCG00000035995	1.088497089	0.003845868
ENSSSCG00000002269	1.088089233	6.54E-08
ENSSSCG00000002135	1.087928291	5.39E-05
ENSSSCG00000036967	1.087869345	7.48E-05
ENSSSCG00000005528	1.087791535	0.003554036
ENSSSCG00000017669	1.087715727	0.00269514
ENSSSCG00000038947	1.087629219	3.33E-06
ENSSSCG00000038660	1.086771576	0.001328353
ENSSSCG00000001512	1.086220152	0.000855229
ENSSSCG00000005055	1.085358073	0.002297779
ENSSSCG00000036824	1.084007787	0.000562503
ENSSSCG00000036308	1.083918802	1.03E-05
ENSSSCG00000003464	1.08363081	5.04E-07
ENSSSCG00000010468	1.082517133	0.000442277
ENSSSCG00000008238	1.082109118	3.49E-06
ENSSSCG00000042527	1.082058375	0.008151813
ENSSSCG00000022545	1.081683916	1.56E-05
ENSSSCG00000017907	1.081575807	0.002430122
ENSSSCG00000015650	1.08151097	2.65E-06
ENSSSCG00000025174	1.081476777	1.21E-08
ENSSSCG00000006543	1.081279972	0.000391526
ENSSSCG00000010734	1.081229787	3.47E-05
ENSSSCG00000004687	1.080830288	0.000504467
ENSSSCG00000008874	1.080480802	0.003057526
ENSSSCG00000016193	1.080364175	3.70E-07
ENSSSCG00000001398	1.080063174	2.30E-05
ENSSSCG00000010289	1.079982565	6.26E-06
ENSSSCG00000033506	1.079850492	0.001776899
ENSSSCG00000027287	1.079534364	1.25E-06
ENSSSCG00000027506	1.078726529	2.30E-06
ENSSSCG00000034858	1.078684744	0.002204584
ENSSSCG00000006239	1.078073649	0.002360422
ENSSSCG00000024222	1.077090917	9.70E-08
ENSSSCG00000000757	1.07696859	5.62E-10
ENSSSCG00000025791	1.076702277	9.87E-06
ENSSSCG00000016101	1.076630293	8.99E-09
ENSSSCG00000008334	1.076243762	0.000409814
ENSSSCG00000010794	1.075934972	2.59E-05
ENSSSCG00000030089	1.075905364	1.70E-05

ENSSSCG00000008534	1.075643875	1.44E-08
ENSSSCG00000023033	1.075563909	2.22E-09
ENSSSCG00000029830	1.075491806	0.002461442
ENSSSCG00000009186	1.075303698	1.88E-08
ENSSSCG00000016206	1.075105312	0.000262766
ENSSSCG00000035123	1.075102362	0.009790271
ENSSSCG00000002805	1.07461979	1.37E-06
ENSSSCG00000017355	1.074571739	0.000647145
ENSSSCG00000050110	1.07426049	1.44E-05
ENSSSCG00000015963	1.074253606	9.11E-05
ENSSSCG00000007352	1.073935832	9.01E-06
ENSSSCG00000035082	1.073742824	9.07E-05
ENSSSCG00000009622	1.073556043	5.80E-10
ENSSSCG00000027847	1.073378521	0.000907978
ENSSSCG00000032062	1.073154818	6.38E-05
ENSSSCG00000031498	1.073070005	0.000135854
ENSSSCG00000001474	1.073007567	6.02E-06
ENSSSCG00000007469	1.072874241	0.000218925
ENSSSCG00000005656	1.072566273	9.72E-07
ENSSSCG00000028949	1.071291712	2.28E-05
ENSSSCG00000009177	1.071263072	2.13E-05
ENSSSCG00000002460	1.071129122	1.09E-08
ENSSSCG00000007203	1.070894404	5.99E-10
ENSSSCG00000038251	1.070878188	0.000979929
ENSSSCG00000036944	1.070840824	4.76E-06
ENSSSCG00000011901	1.070432326	8.74E-06
ENSSSCG00000032165	1.070057226	0.008434097
ENSSSCG00000000399	1.070006495	0.00063844
ENSSSCG00000033323	1.06842989	9.24E-05
ENSSSCG00000042788	1.068164512	0.001901899
ENSSSCG00000031450	1.067679767	0.000160038
ENSSSCG00000040943	1.067290919	2.23E-05
ENSSSCG00000016374	1.067236099	8.66E-06
ENSSSCG00000006260	1.066999274	0.000204475
ENSSSCG00000028513	1.066840985	2.75E-06
ENSSSCG00000009779	1.066704885	2.29E-06
ENSSSCG00000030467	1.066599504	0.00212972
ENSSSCG00000036620	1.066391225	9.15E-05
ENSSSCG00000006644	1.06637582	1.32E-06
ENSSSCG00000005768	1.066295474	0.000494014
ENSSSCG00000010743	1.066017292	0.000330218
ENSSSCG00000005737	1.065981825	0.009099921
ENSSSCG00000012961	1.065954303	0.003451691
ENSSSCG00000027509	1.065223587	1.93E-05
ENSSSCG00000002965	1.06513133	0.00802869
ENSSSCG00000013293	1.065103083	0.003293703
ENSSSCG00000005399	1.064973837	0.000230298
ENSSSCG00000001394	1.064659455	6.81E-09
ENSSSCG00000008812	1.064578682	0.000173086
ENSSSCG00000013768	1.064539385	0.008522132
ENSSSCG00000006900	1.064495417	0.00059208
ENSSSCG00000016577	1.064371725	0.005049009
ENSSSCG00000006363	1.063904988	0.000122197
ENSSSCG00000000169	1.063815966	2.56E-05
ENSSSCG00000024373	1.062781893	2.29E-06
ENSSSCG00000010923	1.062504747	0.000112205
ENSSSCG00000008550	1.062451586	4.22E-08
ENSSSCG00000038487	1.062175817	1.53E-05

ENSSSCG00000031639	1.06209394	5.54E-07
ENSSSCG00000012621	1.061581309	9.50E-05
ENSSSCG00000013615	1.061542797	0.000160969
ENSSSCG00000011540	1.060387633	0.008387046
ENSSSCG00000016009	1.060105952	5.08E-06
ENSSSCG00000051054	1.059343341	0.001870109
ENSSSCG00000040185	1.05916937	2.54E-05
ENSSSCG00000010176	1.059039651	1.42E-06
ENSSSCG00000029857	1.058550528	9.18E-07
ENSSSCG00000027974	1.058116774	0.000260527
ENSSSCG00000033465	1.057963863	0.000355707
ENSSSCG00000029698	1.057518127	0.000123457
ENSSSCG00000038917	1.05716724	0.001447924
ENSSSCG00000018055	1.05700875	0.006167966
ENSSSCG00000007609	1.056919953	1.36E-08
ENSSSCG00000010016	1.056608214	7.74E-05
ENSSSCG00000011312	1.056425745	7.43E-05
ENSSSCG00000027911	1.056357945	0.011187345
ENSSSCG00000010564	1.056195704	0.000111688
ENSSSCG00000022048	1.055880051	7.87E-06
ENSSSCG00000040439	1.055562599	0.00014979
ENSSSCG00000013655	1.055471487	1.82E-05
ENSSSCG00000039663	1.055423107	0.000145059
ENSSSCG00000051552	1.0548878	6.65E-06
ENSSSCG00000010607	1.054164447	0.006230846
ENSSSCG00000024245	1.053814925	0.003410424
ENSSSCG00000010201	1.0536588	3.28E-05
ENSSSCG00000000037	1.053580994	4.15E-08
ENSSSCG00000009349	1.053383817	0.000124403
ENSSSCG00000013054	1.053056294	3.48E-06
ENSSSCG00000008180	1.052784336	9.76E-07
ENSSSCG00000024070	1.052687012	0.00116949
ENSSSCG00000011106	1.052365726	2.76E-05
ENSSSCG00000013470	1.052288952	0.000353477
ENSSSCG00000046723	1.052257371	0.016682736
ENSSSCG00000012127	1.05224284	0.010858055
ENSSSCG00000031360	1.052043438	8.80E-08
ENSSSCG00000030428	1.051645301	0.000131734
ENSSSCG00000035719	1.05156262	0.006180507
ENSSSCG00000010493	1.051545127	3.67E-06
ENSSSCG00000014031	1.051454882	6.74E-07
ENSSSCG00000031918	1.051316365	0.003706132
ENSSSCG00000042089	1.050968852	0.017231952
ENSSSCG00000026697	1.050119801	0.000122427
ENSSSCG00000009788	1.049826555	0.004808137
ENSSSCG00000000500	1.049627624	1.62E-06
ENSSSCG00000000908	1.049294614	0.000243954
ENSSSCG00000032674	1.048630856	0.007711145
ENSSSCG00000029949	1.047898411	0.006771922
ENSSSCG00000001710	1.047880213	1.52E-06
ENSSSCG00000012021	1.047181483	0.00011181
ENSSSCG00000028481	1.047036069	0.000704714
ENSSSCG00000010445	1.04661634	0.002369616
ENSSSCG00000004700	1.046425573	4.23E-05
ENSSSCG00000002740	1.046291179	1.32E-06
ENSSSCG00000014559	1.045555971	0.00154584
ENSSSCG00000007119	1.045494735	0.001605603
ENSSSCG00000022512	1.044993644	0.000412822

ENSSSCG00000013421	1.044796377	2.61E-09
ENSSSCG00000010401	1.04477514	1.83E-05
ENSSSCG00000024419	1.043894458	5.96E-07
ENSSSCG00000006926	1.043583302	0.00012229
ENSSSCG00000039202	1.043488778	0.000259849
ENSSSCG00000015040	1.042973614	0.009183082
ENSSSCG00000028512	1.042339279	1.03E-05
ENSSSCG00000000580	1.042031064	2.09E-06
ENSSSCG00000005532	1.041834356	0.00031866
ENSSSCG00000026829	1.041717213	1.34E-05
ENSSSCG00000010529	1.040665004	0.008899081
ENSSSCG00000010645	1.040081654	0.005037185
ENSSSCG00000016036	1.039806677	2.99E-07
ENSSSCG00000004385	1.039772767	5.55E-05
ENSSSCG00000016708	1.03871916	1.48E-05
ENSSSCG00000031509	1.036820778	0.000461788
ENSSSCG00000007812	1.03645075	2.76E-07
ENSSSCG00000007932	1.035734477	0.002779576
ENSSSCG00000005494	1.035303161	0.000419787
ENSSSCG00000028869	1.034968009	6.74E-06
ENSSSCG00000017012	1.034756281	2.15E-07
ENSSSCG00000017421	1.034641873	3.77E-06
ENSSSCG00000007315	1.034262318	0.000142596
ENSSSCG00000012241	1.033781653	0.000495469
ENSSSCG00000000920	1.032737672	0.000502772
ENSSSCG00000040599	1.032593694	0.000215209
ENSSSCG00000036423	1.032426948	2.68E-09
ENSSSCG00000038900	1.031816353	0.000110158
ENSSSCG00000039338	1.031569364	0.000194405
ENSSSCG00000036742	1.031489299	0.000608712
ENSSSCG00000027903	1.031473149	0.002492666
ENSSSCG00000012074	1.031437965	0.000488773
ENSSSCG00000008924	1.031302002	4.05E-05
ENSSSCG00000037381	1.030993193	0.000313293
ENSSSCG00000028113	1.030823122	0.000145845
ENSSSCG00000027792	1.030791235	2.71E-05
ENSSSCG00000022005	1.030263486	0.000656219
ENSSSCG00000039235	1.030235253	0.000137325
ENSSSCG00000004057	1.029825314	7.29E-06
ENSSSCG00000014073	1.029044576	0.000518824
ENSSSCG00000029230	1.027956702	3.62E-06
ENSSSCG00000017191	1.027695221	9.72E-10
ENSSSCG00000024661	1.027688164	0.000802406
ENSSSCG00000035650	1.027674875	3.53E-09
ENSSSCG00000000164	1.027127766	0.00019397
ENSSSCG00000020783	1.025771712	2.62E-05
ENSSSCG00000011336	1.025392418	0.000994388
ENSSSCG00000045404	1.024948083	0.013416291
ENSSSCG00000006107	1.024501456	0.000145713
ENSSSCG00000016331	1.024392621	0.006337083
ENSSSCG00000020701	1.023946052	0.007148217
ENSSSCG00000032284	1.023890286	5.64E-06
ENSSSCG00000002841	1.023838802	2.54E-08
ENSSSCG00000006995	1.023495213	0.001253949
ENSSSCG00000007943	1.02289217	0.002514055
ENSSSCG00000036213	1.022818786	0.000929329
ENSSSCG00000029484	1.022584062	8.90E-05
ENSSSCG00000022912	1.022523313	8.56E-05

ENSSSCG00000016412	1.022193431	3.00E-07
ENSSSCG00000039348	1.022156317	1.24E-06
ENSSSCG00000016768	1.021991746	7.71E-05
ENSSSCG00000009650	1.021401791	0.015299735
ENSSSCG00000037772	1.019089905	5.17E-05
ENSSSCG00000010972	1.019082553	0.00079206
ENSSSCG00000010908	1.018707838	0.002320379
ENSSSCG00000027779	1.018142046	2.16E-07
ENSSSCG00000007681	1.018083666	8.29E-06
ENSSSCG00000015955	1.017780551	0.00010364
ENSSSCG00000013659	1.017475871	0.000792519
ENSSSCG00000030042	1.016674371	0.000193878
ENSSSCG00000023599	1.016633402	0.003368646
ENSSSCG00000018007	1.016520881	0.003657804
ENSSSCG00000012757	1.016250446	0.000419972
ENSSSCG00000017513	1.015863983	1.62E-05
ENSSSCG00000032556	1.015286661	3.90E-05
ENSSSCG00000011127	1.015266568	0.000510649
ENSSSCG00000014255	1.014665671	5.15E-05
ENSSSCG00000000273	1.01443241	0.000820219
ENSSSCG00000006301	1.014342473	0.000131827
ENSSSCG00000007481	1.014233062	0.00052493
ENSSSCG00000004552	1.014038272	4.97E-07
ENSSSCG00000031991	1.013885933	0.001179551
ENSSSCG00000034387	1.013674615	0.008110585
ENSSSCG00000012146	1.013382524	0.000199372
ENSSSCG00000038861	1.013040014	0.000975857
ENSSSCG00000041220	1.012518302	0.005418649
ENSSSCG00000006468	1.012287366	6.06E-07
ENSSSCG00000016907	1.012152183	1.23E-05
ENSSSCG00000046892	1.011842546	0.000367434
ENSSSCG00000035034	1.011778222	0.00102769
ENSSSCG00000002374	1.011660301	4.09E-08
ENSSSCG00000021005	1.01126524	4.95E-06
ENSSSCG00000038203	1.011029748	0.000546089
ENSSSCG00000040359	1.010139064	0.001999688
ENSSSCG00000010703	1.010137028	0.000258399
ENSSSCG00000025729	1.009637549	0.01243349
ENSSSCG00000001527	1.009524377	2.04E-06
ENSSSCG00000004464	1.009340324	1.46E-07
ENSSSCG00000038362	1.009132765	3.55E-05
ENSSSCG00000011514	1.008994522	0.000102121
ENSSSCG00000008188	1.007979814	1.83E-06
ENSSSCG00000013284	1.007878051	0.000145174
ENSSSCG00000011068	1.007490664	2.98E-06
ENSSSCG00000014199	1.007318541	6.80E-05
ENSSSCG00000027381	1.007043189	2.43E-05
ENSSSCG00000000080	1.006910793	7.55E-05
ENSSSCG00000041487	1.006354235	0.002080757
ENSSSCG00000012939	1.006203371	0.000927644
ENSSSCG00000021628	1.005694835	0.002763036
ENSSSCG00000008548	1.005549285	2.79E-05
ENSSSCG00000005837	1.005011111	0.000436083
ENSSSCG00000014080	1.004336385	0.000496528
ENSSSCG00000004371	1.004283115	0.016579558
ENSSSCG00000008569	1.004103483	2.62E-05
ENSSSCG00000004120	1.003915526	0.001062651
ENSSSCG00000011673	1.003764854	0.0014814

ENSSSCG00000009633	1.003524259	0.000236638
ENSSSCG00000011415	1.003242134	1.61E-05
ENSSSCG00000010973	1.002475636	0.002441554
ENSSSCG00000027224	1.002075608	1.39E-07
ENSSSCG00000004342	1.001681196	0.00029103
ENSSSCG00000016134	1.001076742	5.02E-06
ENSSSCG00000008921	1.000985841	0.000118695
ENSSSCG00000014371	1.000635003	0.014234365
ENSSSCG00000036870	1.00062174	3.26E-05
ENSSSCG00000000202	1.000351612	0.000362344
ENSSSCG00000006213	-1.001526276	0.004676419
ENSSSCG00000035400	-1.001636938	0.00379158
ENSSSCG00000007939	-1.00316456	0.000197791
ENSSSCG00000006864	-1.004071584	0.002863485
ENSSSCG00000015853	-1.004743686	0.006898144
ENSSSCG00000047112	-1.005277865	0.000637852
ENSSSCG00000022506	-1.005325626	5.98E-07
ENSSSCG00000047067	-1.005834399	0.012522208
ENSSSCG00000004441	-1.006314415	3.28E-05
ENSSSCG00000003024	-1.00653087	0.000265414
ENSSSCG00000038598	-1.006665457	0.003682733
ENSSSCG00000028293	-1.007368493	1.47E-05
ENSSSCG00000032151	-1.007791695	0.000414254
ENSSSCG00000013023	-1.009253479	2.51E-06
ENSSSCG00000031337	-1.009937781	6.55E-06
ENSSSCG00000011923	-1.010884336	0.000211658
ENSSSCG00000004698	-1.011200899	9.60E-05
ENSSSCG00000013742	-1.011265596	1.26E-05
ENSSSCG00000037791	-1.011456477	0.000150535
ENSSSCG00000004281	-1.011953921	0.000393227
ENSSSCG00000004561	-1.012636551	0.004774422
ENSSSCG00000016164	-1.012744497	1.78E-06
ENSSSCG00000011775	-1.012758916	0.000719668
ENSSSCG00000016368	-1.013018643	9.55E-06
ENSSSCG00000020785	-1.013050111	0.000186385
ENSSSCG00000001504	-1.013558273	0.006469461
ENSSSCG00000012597	-1.013990228	0.000767902
ENSSSCG00000016290	-1.014366981	0.005361034
ENSSSCG00000032661	-1.017250058	0.000549179
ENSSSCG00000028944	-1.018468517	2.24E-08
ENSSSCG00000013455	-1.018943956	0.001370811
ENSSSCG00000003971	-1.019436314	7.62E-06
ENSSSCG00000023857	-1.019977791	0.008901272
ENSSSCG00000043415	-1.021209629	0.017370115
ENSSSCG00000004209	-1.022108832	0.001594322
ENSSSCG00000007682	-1.023493567	0.000295436
ENSSSCG00000015770	-1.023619789	7.81E-05
ENSSSCG00000026045	-1.024914913	8.23E-05
ENSSSCG00000008593	-1.024961685	0.014670157
ENSSSCG00000012150	-1.02507081	0.005264063
ENSSSCG00000009833	-1.025812249	0.000127505
ENSSSCG00000004275	-1.026887634	0.001704703
ENSSSCG00000015290	-1.026935879	3.55E-06
ENSSSCG00000016069	-1.028122108	2.57E-07
ENSSSCG00000024950	-1.028755306	0.000406773
ENSSSCG00000010862	-1.029028461	0.007474854
ENSSSCG00000007542	-1.029081517	0.003248022
ENSSSCG00000011014	-1.029781534	0.000797348

ENSSSCG00000003990	-1.030480081	0.000510231
ENSSSCG00000045081	-1.030606153	0.012915828
ENSSSCG00000002640	-1.030719289	2.38E-08
ENSSSCG00000032017	-1.031230933	0.000108452
ENSSSCG00000041918	-1.031253197	0.000617686
ENSSSCG00000024818	-1.031263982	1.44E-05
ENSSSCG00000003376	-1.032192282	5.90E-06
ENSSSCG00000024263	-1.032875016	0.010700329
ENSSSCG00000002633	-1.033150188	7.87E-06
ENSSSCG00000035697	-1.033188951	0.01167423
ENSSSCG00000038712	-1.033391996	0.00026894
ENSSSCG00000041213	-1.033491584	0.013888592
ENSSSCG00000007252	-1.034235354	0.002739722
ENSSSCG00000032113	-1.034847356	0.000233636
ENSSSCG00000049991	-1.036939871	0.000105177
ENSSSCG00000009169	-1.037311441	8.82E-06
ENSSSCG00000009429	-1.038343008	0.000656668
ENSSSCG00000039245	-1.038876877	0.007356064
ENSSSCG00000010825	-1.03950427	0.000354695
ENSSSCG00000016784	-1.040338683	0.000272451
ENSSSCG00000004332	-1.040377676	0.000995217
ENSSSCG00000040125	-1.04044131	0.002319921
ENSSSCG00000012917	-1.040560724	2.83E-07
ENSSSCG00000006162	-1.041076206	0.000605083
ENSSSCG00000045295	-1.042305214	0.014111262
ENSSSCG00000049961	-1.043215233	0.000991545
ENSSSCG00000049840	-1.04366588	0.000105311
ENSSSCG00000022963	-1.044005198	0.000431347
ENSSSCG00000010969	-1.044149883	0.000235966
ENSSSCG00000028461	-1.045900206	1.26E-05
ENSSSCG00000040849	-1.046057761	1.67E-05
ENSSSCG00000016756	-1.046200648	0.010246733
ENSSSCG00000036520	-1.047033646	0.00572141
ENSSSCG00000008624	-1.048527459	0.00031776
ENSSSCG00000008185	-1.050234528	0.000512541
ENSSSCG00000007074	-1.050253282	1.76E-05
ENSSSCG00000026583	-1.05109851	3.01E-07
ENSSSCG00000029849	-1.051702025	1.02E-07
ENSSSCG00000031129	-1.052029756	1.01E-05
ENSSSCG00000038044	-1.05263442	7.89E-05
ENSSSCG00000001720	-1.052869285	0.002390768
ENSSSCG00000014029	-1.052902492	0.008478355
ENSSSCG00000030362	-1.053267894	0.002856458
ENSSSCG00000029860	-1.053625168	2.47E-07
ENSSSCG00000006285	-1.053905612	0.000195132
ENSSSCG00000031228	-1.054213012	0.002898068
ENSSSCG00000008613	-1.054461935	1.16E-05
ENSSSCG00000007344	-1.054513035	0.000200596
ENSSSCG00000008262	-1.05456005	5.27E-06
ENSSSCG00000005045	-1.05488056	1.41E-05
ENSSSCG00000017578	-1.055991357	4.59E-05
ENSSSCG00000014800	-1.056483782	0.001865634
ENSSSCG00000034493	-1.056722032	0.00024691
ENSSSCG00000013495	-1.056732305	8.47E-06
ENSSSCG00000044220	-1.057087066	0.008704726
ENSSSCG00000006318	-1.058115083	0.000382845
ENSSSCG00000012532	-1.058359472	0.01154866
ENSSSCG00000011811	-1.059024633	0.00166124

ENSSSCG00000009844	-1.059949979	0.015330376
ENSSSCG00000009326	-1.060177167	0.000111021
ENSSSCG00000048583	-1.060529517	0.012429296
ENSSSCG00000035479	-1.061013715	0.000633501
ENSSSCG00000015426	-1.061529204	0.010563926
ENSSSCG00000024418	-1.062648907	0.016002025
ENSSSCG00000002921	-1.062674825	3.57E-06
ENSSSCG00000029284	-1.062728974	0.005733134
ENSSSCG00000027677	-1.063117782	0.002057794
ENSSSCG00000027331	-1.065540521	0.002337068
ENSSSCG00000009377	-1.067679747	0.00669165
ENSSSCG00000026367	-1.069055469	0.001519482
ENSSSCG00000017142	-1.069969212	1.97E-06
ENSSSCG00000004829	-1.070012166	0.001899125
ENSSSCG00000022462	-1.070112678	0.001002824
ENSSSCG00000002525	-1.070555715	1.25E-07
ENSSSCG00000040607	-1.070726624	8.53E-05
ENSSSCG00000051668	-1.071082411	0.006868372
ENSSSCG00000017337	-1.072243342	0.000187048
ENSSSCG00000004543	-1.072544802	0.002237617
ENSSSCG00000043418	-1.074223632	0.008545857
ENSSSCG00000013839	-1.074307505	9.74E-06
ENSSSCG00000015545	-1.074653322	1.84E-08
ENSSSCG00000012173	-1.074671345	0.000564906
ENSSSCG00000026116	-1.075884757	1.23E-07
ENSSSCG00000038296	-1.078850005	0.001318295
ENSSSCG00000005524	-1.07975659	0.006954661
ENSSSCG00000037832	-1.080004565	6.01E-05
ENSSSCG00000038844	-1.081226535	0.001225972
ENSSSCG00000031488	-1.08200503	6.38E-11
ENSSSCG00000035581	-1.08284028	0.006360496
ENSSSCG00000048337	-1.083818006	0.002407425
ENSSSCG00000037697	-1.084189134	0.005146284
ENSSSCG00000013339	-1.08503067	0.00676038
ENSSSCG00000013784	-1.085866731	0.001615973
ENSSSCG00000043623	-1.086254742	0.014414499
ENSSSCG00000011443	-1.08701306	5.31E-06
ENSSSCG00000004478	-1.087070603	0.001009302
ENSSSCG00000016992	-1.087313482	0.000324614
ENSSSCG00000050172	-1.087425087	7.89E-07
ENSSSCG00000037487	-1.087555103	0.004565116
ENSSSCG00000041008	-1.090786921	0.011502298
ENSSSCG00000014575	-1.091170399	0.007561919
ENSSSCG00000034980	-1.091357362	4.71E-06
ENSSSCG00000015802	-1.091556623	0.003133937
ENSSSCG00000050597	-1.091806689	0.002071461
ENSSSCG00000026547	-1.091812418	5.04E-06
ENSSSCG00000015656	-1.091899217	4.04E-05
ENSSSCG00000025653	-1.092070441	5.60E-08
ENSSSCG00000039824	-1.096994386	2.89E-08
ENSSSCG00000006698	-1.097827317	0.000248015
ENSSSCG00000039618	-1.097839114	0.001754787
ENSSSCG00000014114	-1.097862827	1.01E-05
ENSSSCG00000013351	-1.098390537	0.016946987
ENSSSCG00000007541	-1.099038316	6.05E-06
ENSSSCG00000027812	-1.099705972	4.27E-07
ENSSSCG00000007085	-1.100526715	0.000561131
ENSSSCG00000044223	-1.10150997	0.018642252

ENSSSCG00000008096	-1.101590426	0.001039925
ENSSSCG00000027053	-1.105831864	0.000111959
ENSSSCG00000010180	-1.107394684	5.36E-08
ENSSSCG00000036445	-1.111595645	5.82E-07
ENSSSCG00000021397	-1.111887414	0.00084192
ENSSSCG00000033937	-1.112417538	0.005424079
ENSSSCG00000010538	-1.112562447	3.69E-05
ENSSSCG00000015487	-1.112787126	0.017996353
ENSSSCG00000007636	-1.112868567	2.79E-05
ENSSSCG00000001787	-1.113221697	3.99E-10
ENSSSCG00000003147	-1.11407636	0.01353961
ENSSSCG00000012292	-1.114097939	0.003212759
ENSSSCG00000027314	-1.11553071	3.09E-07
ENSSSCG00000007093	-1.11740279	6.40E-07
ENSSSCG00000012264	-1.117773573	2.66E-05
ENSSSCG00000006452	-1.11804355	2.80E-05
ENSSSCG00000037559	-1.119096799	0.000612474
ENSSSCG00000000036	-1.1215932	1.78E-05
ENSSSCG00000005224	-1.12177346	0.002622636
ENSSSCG00000033759	-1.121824611	3.42E-08
ENSSSCG00000009230	-1.122699559	1.75E-05
ENSSSCG00000023611	-1.123459835	1.09E-05
ENSSSCG00000030921	-1.123817927	0.002373801
ENSSSCG00000045182	-1.12415655	0.013372551
ENSSSCG00000008111	-1.12494948	0.003495564
ENSSSCG00000040095	-1.125216452	1.12E-09
ENSSSCG00000010447	-1.12626247	5.23E-06
ENSSSCG00000017500	-1.126313634	0.006875369
ENSSSCG00000024088	-1.127261171	0.001987723
ENSSSCG00000011434	-1.127593429	2.62E-09
ENSSSCG00000008266	-1.127615555	5.33E-07
ENSSSCG00000041783	-1.127673117	5.66E-06
ENSSSCG00000042694	-1.127923329	0.003973314
ENSSSCG00000040838	-1.128214084	0.007597606
ENSSSCG00000040328	-1.128453662	0.000369769
ENSSSCG00000016444	-1.128809089	0.001092537
ENSSSCG00000029598	-1.129104691	0.000319755
ENSSSCG00000040118	-1.129604434	0.001447008
ENSSSCG00000031142	-1.130930681	0.000355467
ENSSSCG00000046561	-1.132353986	0.002124341
ENSSSCG00000036575	-1.133302999	6.30E-06
ENSSSCG00000039874	-1.133344643	0.003677076
ENSSSCG00000009388	-1.133424631	0.005633431
ENSSSCG00000011899	-1.134585289	5.03E-09
ENSSSCG00000035222	-1.134932187	0.002351731
ENSSSCG00000025425	-1.135019477	7.39E-09
ENSSSCG00000028572	-1.135025492	1.07E-08
ENSSSCG00000009148	-1.136048164	4.29E-08
ENSSSCG00000027093	-1.137554327	0.004060254
ENSSSCG00000045672	-1.137812975	0.003231276
ENSSSCG00000043201	-1.138043186	0.00268222
ENSSSCG00000016432	-1.138285006	3.68E-05
ENSSSCG00000009792	-1.138407326	0.010905433
ENSSSCG00000010879	-1.139339025	0.00017696
ENSSSCG00000013335	-1.13986483	0.0090466
ENSSSCG00000009956	-1.140682326	5.34E-08
ENSSSCG00000010534	-1.141186971	2.94E-07
ENSSSCG00000037682	-1.141542499	2.19E-05

ENSSSCG00000007181	-1.141680509	0.017268673
ENSSSCG00000032984	-1.142079119	1.58E-06
ENSSSCG00000002025	-1.142201088	2.67E-05
ENSSSCG00000015570	-1.143767632	1.44E-08
ENSSSCG00000021069	-1.145941238	7.56E-05
ENSSSCG00000011589	-1.145947661	0.00117847
ENSSSCG00000008835	-1.146268678	0.0101269
ENSSSCG00000051426	-1.147390039	0.010126921
ENSSSCG00000012859	-1.147770045	2.61E-08
ENSSSCG00000009605	-1.14782494	0.00227495
ENSSSCG00000035093	-1.148354368	4.83E-06
ENSSSCG00000001543	-1.148662346	0.014708727
ENSSSCG00000014039	-1.14983834	2.78E-09
ENSSSCG00000032146	-1.151933611	2.18E-05
ENSSSCG00000013235	-1.152351651	1.12E-07
ENSSSCG00000000263	-1.152565292	3.03E-06
ENSSSCG00000044439	-1.153381801	0.009813596
ENSSSCG00000026994	-1.153650991	1.68E-06
ENSSSCG00000002786	-1.153894369	0.003147352
ENSSSCG00000042932	-1.153925075	1.94E-08
ENSSSCG00000001395	-1.154037173	8.90E-07
ENSSSCG00000008041	-1.154320553	1.85E-06
ENSSSCG00000004207	-1.154534126	0.000149491
ENSSSCG00000050230	-1.154663798	0.000363536
ENSSSCG00000012278	-1.155199565	5.14E-05
ENSSSCG00000001819	-1.155445909	4.38E-05
ENSSSCG00000013236	-1.156239458	0.013976916
ENSSSCG00000014043	-1.156698814	6.59E-07
ENSSSCG00000009111	-1.156976123	0.000210379
ENSSSCG00000000896	-1.159195157	0.001782947
ENSSSCG00000006568	-1.160320247	1.87E-07
ENSSSCG00000006862	-1.161065316	6.87E-06
ENSSSCG00000011663	-1.161256989	4.23E-05
ENSSSCG00000036446	-1.162049382	0.000470411
ENSSSCG00000011056	-1.163364415	2.66E-05
ENSSSCG00000005190	-1.163493988	0.000614309
ENSSSCG00000015810	-1.164409062	0.010142659
ENSSSCG00000006664	-1.164998756	0.000229953
ENSSSCG00000007528	-1.165752423	0.015029096
ENSSSCG00000011788	-1.165919027	1.28E-07
ENSSSCG00000022828	-1.166039665	6.82E-05
ENSSSCG00000050368	-1.167072305	0.000704442
ENSSSCG00000006788	-1.168700302	5.26E-05
ENSSSCG00000002857	-1.16895761	0.000541864
ENSSSCG00000007958	-1.169205686	6.26E-10
ENSSSCG00000015116	-1.169709238	6.91E-08
ENSSSCG00000008965	-1.170550088	0.002342806
ENSSSCG00000006940	-1.170603624	3.23E-05
ENSSSCG00000048237	-1.170909934	0.00309974
ENSSSCG00000007149	-1.172147919	0.000572248
ENSSSCG00000048968	-1.174143751	6.13E-05
ENSSSCG00000007979	-1.175913207	3.44E-05
ENSSSCG00000003584	-1.175932128	4.29E-07
ENSSSCG00000049042	-1.175943057	0.002189972
ENSSSCG00000014391	-1.177350425	2.64E-10
ENSSSCG00000035430	-1.177592042	5.46E-07
ENSSSCG00000003940	-1.179414863	7.50E-05
ENSSSCG00000015350	-1.179703501	0.012703324

ENSSSCG00000034978	-1.180104144	1.25E-07
ENSSSCG00000007517	-1.180661814	0.000138542
ENSSSCG00000032078	-1.181570921	2.55E-10
ENSSSCG00000006161	-1.182524195	4.79E-09
ENSSSCG00000011353	-1.183806338	3.76E-12
ENSSSCG00000009874	-1.184197827	1.03E-06
ENSSSCG00000043146	-1.18500177	0.013591556
ENSSSCG00000000848	-1.185845946	0.00329587
ENSSSCG00000048556	-1.186637623	0.000612069
ENSSSCG00000008281	-1.187165712	0.002291164
ENSSSCG00000011675	-1.188597274	0.011224192
ENSSSCG00000049217	-1.189183689	0.000692659
ENSSSCG00000003070	-1.189916504	1.19E-05
ENSSSCG00000018092	-1.190278934	0.006434979
ENSSSCG00000018029	-1.190529641	0.000349188
ENSSSCG00000009022	-1.190782299	0.00428714
ENSSSCG00000044648	-1.191718407	0.000525213
ENSSSCG00000007147	-1.191889265	3.39E-05
ENSSSCG00000038886	-1.19444312	0.00012717
ENSSSCG00000030065	-1.194616397	7.79E-06
ENSSSCG00000032413	-1.194768525	5.08E-05
ENSSSCG00000005313	-1.195610678	0.000225303
ENSSSCG00000032417	-1.196039482	0.000154983
ENSSSCG00000010861	-1.196493002	1.09E-09
ENSSSCG00000000916	-1.197035117	0.006910823
ENSSSCG00000040942	-1.197128334	0.000541838
ENSSSCG00000004223	-1.197325813	0.007712708
ENSSSCG00000038110	-1.198154031	0.001244831
ENSSSCG00000013088	-1.198631125	0.000419231
ENSSSCG00000029624	-1.198774433	3.18E-12
ENSSSCG00000033641	-1.19890334	0.008913929
ENSSSCG00000034272	-1.199778164	2.74E-06
ENSSSCG00000009152	-1.200390871	0.010390986
ENSSSCG00000011377	-1.201046985	1.61E-05
ENSSSCG00000043314	-1.201833685	0.004326815
ENSSSCG00000037880	-1.202159453	5.41E-06
ENSSSCG00000035078	-1.202305529	1.91E-06
ENSSSCG00000013295	-1.202510565	7.82E-08
ENSSSCG00000007568	-1.202742644	8.30E-09
ENSSSCG00000048954	-1.203467124	0.007020308
ENSSSCG00000026536	-1.20411483	1.10E-10
ENSSSCG00000043116	-1.204501305	3.38E-07
ENSSSCG00000003711	-1.205123474	8.61E-05
ENSSSCG00000034545	-1.205269895	6.62E-05
ENSSSCG00000016868	-1.205463163	2.12E-06
ENSSSCG00000006781	-1.206532453	7.32E-06
ENSSSCG00000013393	-1.206574335	0.014492249
ENSSSCG00000015792	-1.206577951	0.004301052
ENSSSCG00000041431	-1.206646536	0.016914551
ENSSSCG00000033997	-1.207241657	0.006502432
ENSSSCG00000022678	-1.207768296	0.017051271
ENSSSCG00000047790	-1.207803951	0.004966662
ENSSSCG00000000040	-1.208952192	7.14E-07
ENSSSCG00000012284	-1.209070668	2.31E-08
ENSSSCG00000051147	-1.209074326	0.002218621
ENSSSCG00000016567	-1.209564966	0.00153531
ENSSSCG00000012759	-1.210424935	5.82E-05
ENSSSCG00000014539	-1.210795525	1.72E-06

ENSSSCG00000004405	-1.210857698	1.10E-08
ENSSSCG00000029600	-1.211168432	6.13E-05
ENSSSCG00000007155	-1.213117369	0.000223397
ENSSSCG00000010665	-1.213276782	7.42E-07
ENSSSCG00000024827	-1.214379777	5.18E-10
ENSSSCG00000010209	-1.214390215	0.005719114
ENSSSCG00000033009	-1.214408447	1.71E-09
ENSSSCG00000050540	-1.215265671	2.03E-07
ENSSSCG00000042607	-1.216019077	0.001387071
ENSSSCG00000028925	-1.216706871	4.12E-08
ENSSSCG00000010494	-1.216708345	0.007250218
ENSSSCG00000007778	-1.217494123	0.008838371
ENSSSCG00000015951	-1.218483103	5.60E-05
ENSSSCG00000009827	-1.219236645	1.06E-07
ENSSSCG00000004896	-1.219517608	3.12E-07
ENSSSCG00000044922	-1.220101408	4.88E-08
ENSSSCG00000004347	-1.221105511	3.14E-05
ENSSSCG00000038313	-1.223610086	6.32E-06
ENSSSCG00000016554	-1.223780669	0.000266179
ENSSSCG00000030283	-1.224461216	2.49E-07
ENSSSCG00000047617	-1.22715064	0.000107859
ENSSSCG00000043584	-1.227437068	0.000180069
ENSSSCG00000009048	-1.227585867	2.70E-06
ENSSSCG00000008021	-1.228557551	1.08E-08
ENSSSCG00000040524	-1.229978321	3.32E-05
ENSSSCG00000005178	-1.230935844	0.000582968
ENSSSCG00000031715	-1.2314697	0.000163601
ENSSSCG00000000810	-1.231672766	3.58E-06
ENSSSCG00000013595	-1.231763553	1.08E-07
ENSSSCG00000049997	-1.233251494	4.21E-07
ENSSSCG00000041667	-1.234309501	0.000404731
ENSSSCG00000036747	-1.235195477	5.91E-07
ENSSSCG00000046146	-1.235682767	0.004738272
ENSSSCG00000022202	-1.23690831	0.000380943
ENSSSCG00000036679	-1.237184749	0.000568876
ENSSSCG00000038281	-1.237564168	0.015876769
ENSSSCG00000022447	-1.239629095	0.006320663
ENSSSCG00000007520	-1.240075502	1.33E-11
ENSSSCG00000034440	-1.241137317	0.00143843
ENSSSCG00000044150	-1.242701926	3.98E-06
ENSSSCG00000049902	-1.243528225	0.002102048
ENSSSCG00000049229	-1.244699638	0.017262765
ENSSSCG00000038604	-1.245196146	5.34E-08
ENSSSCG00000029590	-1.245386355	4.21E-06
ENSSSCG00000025254	-1.245983541	1.17E-08
ENSSSCG00000004408	-1.246918902	2.52E-05
ENSSSCG00000010638	-1.247438855	3.07E-08
ENSSSCG00000044618	-1.2474746	0.012099575
ENSSSCG00000001849	-1.248020645	3.41E-07
ENSSSCG00000022611	-1.248048702	2.29E-08
ENSSSCG00000011497	-1.249515263	2.01E-05
ENSSSCG00000007572	-1.25052276	3.65E-07
ENSSSCG00000033817	-1.25098626	0.014878877
ENSSSCG00000017338	-1.251067254	2.45E-06
ENSSSCG00000001485	-1.251865042	0.017780774
ENSSSCG00000003344	-1.252174284	0.000225215
ENSSSCG00000037821	-1.252433275	0.002365996
ENSSSCG00000029875	-1.253097068	4.59E-07

ENSSSCG00000040548	-1.253128763	0.000349925
ENSSSCG00000002660	-1.253172613	7.28E-07
ENSSSCG00000020858	-1.253694605	0.001417411
ENSSSCG00000024341	-1.254476452	8.98E-09
ENSSSCG00000029165	-1.255813625	4.54E-06
ENSSSCG00000027928	-1.256000157	5.65E-05
ENSSSCG00000048545	-1.256052902	0.002378475
ENSSSCG00000011111	-1.257430955	7.42E-07
ENSSSCG00000037792	-1.258919435	0.011975297
ENSSSCG00000007674	-1.259143251	0.00779979
ENSSSCG00000023142	-1.259458079	0.001817236
ENSSSCG00000046947	-1.26033196	0.001813019
ENSSSCG00000023862	-1.261138199	0.000147194
ENSSSCG00000012411	-1.26137884	0.016802338
ENSSSCG00000025545	-1.261538555	1.45E-08
ENSSSCG00000046962	-1.261564846	0.002412089
ENSSSCG00000034027	-1.262386184	0.000342143
ENSSSCG00000013919	-1.263605687	0.000245047
ENSSSCG00000050634	-1.263778916	0.000385925
ENSSSCG00000050821	-1.264409615	0.002591866
ENSSSCG00000004672	-1.26474363	1.87E-06
ENSSSCG00000051342	-1.265041844	0.001282497
ENSSSCG00000008813	-1.265987897	0.000677121
ENSSSCG00000038180	-1.266115128	0.000188706
ENSSSCG00000003054	-1.266579228	0.000366624
ENSSSCG00000012768	-1.266974097	4.37E-09
ENSSSCG00000050966	-1.267097943	0.017281621
ENSSSCG00000041536	-1.267121819	1.54E-05
ENSSSCG00000027877	-1.267150098	8.69E-05
ENSSSCG00000031787	-1.267272559	0.000334336
ENSSSCG00000038539	-1.268027087	0.002553739
ENSSSCG00000015284	-1.26997245	2.14E-10
ENSSSCG00000009836	-1.270530407	1.19E-11
ENSSSCG00000014900	-1.27073814	0.001017053
ENSSSCG00000020758	-1.270991309	0.000125477
ENSSSCG00000008903	-1.272025413	0.000572455
ENSSSCG00000023434	-1.272176705	2.18E-05
ENSSSCG00000009361	-1.273039828	0.002815681
ENSSSCG00000002815	-1.273097361	2.83E-10
ENSSSCG00000030048	-1.273138655	0.00095484
ENSSSCG00000026991	-1.274334129	0.001044038
ENSSSCG00000000811	-1.276018966	1.04E-10
ENSSSCG00000032162	-1.276542203	7.06E-07
ENSSSCG00000017927	-1.2765919	2.73E-06
ENSSSCG00000040037	-1.276746254	2.45E-07
ENSSSCG00000013978	-1.277586534	3.76E-09
ENSSSCG00000008745	-1.278093937	0.000487668
ENSSSCG00000004549	-1.278512186	0.00268915
ENSSSCG00000012323	-1.279619482	5.00E-07
ENSSSCG00000012699	-1.280786163	2.83E-09
ENSSSCG00000031713	-1.280819864	0.012211629
ENSSSCG00000030153	-1.283506333	2.33E-11
ENSSSCG00000012903	-1.284448826	1.87E-09
ENSSSCG00000043344	-1.285028917	0.004197679
ENSSSCG00000043527	-1.289148563	4.87E-05
ENSSSCG00000047227	-1.289315114	0.001950013
ENSSSCG00000014819	-1.289655677	1.13E-10
ENSSSCG00000024960	-1.289889033	1.29E-05

ENSSSCG00000049587	-1.290083283	0.008176941
ENSSSCG00000002863	-1.290752321	0.001262882
ENSSSCG00000037105	-1.29134979	2.24E-10
ENSSSCG00000011077	-1.291384386	4.07E-05
ENSSSCG00000013010	-1.291697733	0.000687097
ENSSSCG00000048060	-1.292326998	0.000261824
ENSSSCG00000033054	-1.29271279	9.21E-05
ENSSSCG00000034630	-1.292962477	0.000490732
ENSSSCG00000028144	-1.293508933	0.000975118
ENSSSCG00000015519	-1.29373075	0.000242265
ENSSSCG00000000370	-1.294034922	2.46E-10
ENSSSCG00000015780	-1.295194476	0.000157265
ENSSSCG00000034749	-1.296279106	0.001021551
ENSSSCG00000050097	-1.296928815	0.000477148
ENSSSCG00000039802	-1.29728306	0.000648482
ENSSSCG00000027700	-1.298780269	1.24E-09
ENSSSCG00000043622	-1.299351401	0.005904259
ENSSSCG00000035887	-1.299846072	0.000102287
ENSSSCG00000031514	-1.300309857	0.006737852
ENSSSCG00000008134	-1.301902186	0.006136321
ENSSSCG00000022722	-1.3021268	4.26E-10
ENSSSCG00000023027	-1.302340097	7.61E-08
ENSSSCG00000034660	-1.304335854	0.006090813
ENSSSCG00000046945	-1.30438703	2.09E-06
ENSSSCG00000006357	-1.304939106	0.001188306
ENSSSCG00000012824	-1.305485167	2.02E-12
ENSSSCG00000029285	-1.308449434	2.05E-07
ENSSSCG00000005627	-1.310732442	0.000130488
ENSSSCG00000010746	-1.311074946	1.29E-06
ENSSSCG00000005832	-1.311967152	3.73E-06
ENSSSCG00000017565	-1.313162109	9.34E-06
ENSSSCG00000028092	-1.313761095	1.04E-06
ENSSSCG00000005893	-1.315204634	2.45E-08
ENSSSCG00000014274	-1.316623002	0.000357339
ENSSSCG00000050471	-1.318270458	0.004715703
ENSSSCG00000041715	-1.318905566	2.92E-05
ENSSSCG00000003708	-1.319897291	1.68E-05
ENSSSCG00000043288	-1.320036369	0.003395176
ENSSSCG00000010144	-1.320113414	0.000223827
ENSSSCG00000015250	-1.320641253	0.000117168
ENSSSCG00000005308	-1.321139496	2.32E-05
ENSSSCG00000013879	-1.323255948	1.05E-10
ENSSSCG00000007239	-1.323459591	1.14E-05
ENSSSCG00000049173	-1.323638505	0.01681152
ENSSSCG00000045597	-1.325473278	0.000100752
ENSSSCG00000005916	-1.325772852	2.42E-08
ENSSSCG00000048379	-1.326123689	0.005069483
ENSSSCG00000017116	-1.326727718	5.06E-07
ENSSSCG00000030229	-1.327004573	1.01E-05
ENSSSCG00000026516	-1.327343342	1.21E-06
ENSSSCG00000044517	-1.327563034	0.003723346
ENSSSCG00000034308	-1.328091506	0.000427641
ENSSSCG00000045701	-1.328181577	0.00269321
ENSSSCG00000031924	-1.32822965	0.000119203
ENSSSCG00000008263	-1.329654535	0.001892129
ENSSSCG00000018075	-1.330119644	7.90E-07
ENSSSCG00000030696	-1.330186908	1.52E-06
ENSSSCG00000032313	-1.330260759	1.40E-10

ENSSSCG00000008753	-1.331235028	4.77E-06
ENSSSCG00000029331	-1.333264463	1.03E-12
ENSSSCG00000035649	-1.3345317	7.21E-05
ENSSSCG00000024312	-1.335334984	0.001221672
ENSSSCG00000021067	-1.336013771	0.000506671
ENSSSCG00000017063	-1.336283775	0.003489607
ENSSSCG00000037532	-1.336645367	2.63E-05
ENSSSCG00000039041	-1.338302376	2.85E-10
ENSSSCG00000028623	-1.338562205	2.87E-07
ENSSSCG00000001404	-1.338686324	2.42E-07
ENSSSCG00000015959	-1.339655509	0.017379037
ENSSSCG00000010068	-1.339845431	1.07E-05
ENSSSCG00000010925	-1.340065456	0.000129835
ENSSSCG00000045813	-1.340561921	0.00640953
ENSSSCG00000015908	-1.340563231	1.17E-06
ENSSSCG00000003142	-1.340795784	1.56E-07
ENSSSCG00000022672	-1.341147189	2.21E-05
ENSSSCG00000014242	-1.343230216	1.81E-05
ENSSSCG00000016618	-1.343250644	2.13E-05
ENSSSCG00000041764	-1.347845164	0.006376551
ENSSSCG00000045229	-1.348387689	0.001349734
ENSSSCG00000021439	-1.349117064	2.74E-11
ENSSSCG00000036822	-1.349663657	6.72E-06
ENSSSCG00000025687	-1.350183136	0.000197637
ENSSSCG00000016976	-1.350218851	6.74E-08
ENSSSCG00000032106	-1.350685956	9.45E-08
ENSSSCG00000035218	-1.350898616	1.23E-05
ENSSSCG00000047705	-1.35288499	0.00392417
ENSSSCG00000002425	-1.353174766	2.60E-06
ENSSSCG00000000214	-1.353445298	7.14E-07
ENSSSCG00000007649	-1.355227579	0.015460414
ENSSSCG00000017562	-1.356533615	0.003909851
ENSSSCG00000012744	-1.35670034	0.000960043
ENSSSCG00000011147	-1.357170536	1.76E-06
ENSSSCG00000023714	-1.357175792	2.80E-07
ENSSSCG00000021519	-1.358090576	2.66E-09
ENSSSCG00000012001	-1.358723556	0.000177394
ENSSSCG00000007145	-1.358948529	7.40E-06
ENSSSCG00000020657	-1.35902904	1.19E-06
ENSSSCG00000015357	-1.359378575	0.001426569
ENSSSCG00000006397	-1.360184404	5.85E-07
ENSSSCG00000010101	-1.361575857	1.11E-05
ENSSSCG00000001612	-1.361993857	0.014157368
ENSSSCG00000047521	-1.362902026	0.011373574
ENSSSCG00000046337	-1.363388805	0.00333799
ENSSSCG00000011495	-1.364171138	0.001449474
ENSSSCG00000007334	-1.36434582	5.75E-07
ENSSSCG00000000185	-1.36507829	0.000216655
ENSSSCG00000017951	-1.36583743	0.000272313
ENSSSCG00000001499	-1.365917445	0.000800319
ENSSSCG00000051239	-1.366270185	0.000551756
ENSSSCG00000011618	-1.367761908	5.85E-05
ENSSSCG00000001506	-1.368134525	2.35E-09
ENSSSCG00000013248	-1.368611271	0.01449709
ENSSSCG00000014316	-1.368708826	1.69E-08
ENSSSCG00000007100	-1.369825184	1.52E-07
ENSSSCG00000003322	-1.370250366	4.34E-08
ENSSSCG00000038404	-1.371162935	0.000116309

ENSSSCG00000014854	-1.371548952	0.008802214
ENSSSCG00000011681	-1.372696514	0.000599176
ENSSSCG00000050725	-1.374349891	6.91E-07
ENSSSCG00000043781	-1.374509551	2.29E-05
ENSSSCG00000004661	-1.375046386	2.92E-05
ENSSSCG00000016557	-1.375097946	0.000904687
ENSSSCG00000026850	-1.376967649	3.31E-05
ENSSSCG00000009245	-1.377197361	0.000136823
ENSSSCG00000031801	-1.377704525	0.004513049
ENSSSCG00000028995	-1.377807704	4.88E-07
ENSSSCG00000041766	-1.377970188	3.75E-07
ENSSSCG00000014581	-1.378432187	2.18E-08
ENSSSCG00000027198	-1.378499325	7.59E-07
ENSSSCG00000011928	-1.378527657	0.002890965
ENSSSCG00000000941	-1.379778661	7.47E-08
ENSSSCG00000023483	-1.380195484	4.48E-07
ENSSSCG00000037015	-1.381340353	6.83E-06
ENSSSCG00000007678	-1.381997335	1.44E-05
ENSSSCG00000013545	-1.382240554	2.31E-06
ENSSSCG00000046007	-1.382345883	2.45E-06
ENSSSCG00000007527	-1.383583405	3.24E-07
ENSSSCG00000005657	-1.385391691	0.000231266
ENSSSCG00000005941	-1.385659868	0.003415931
ENSSSCG00000010473	-1.386097065	6.59E-08
ENSSSCG00000049367	-1.387115689	0.01632772
ENSSSCG00000013018	-1.387384017	0.000365582
ENSSSCG00000013403	-1.38783496	0.005244832
ENSSSCG00000010728	-1.387837516	3.30E-05
ENSSSCG00000002333	-1.388690633	3.54E-06
ENSSSCG00000049618	-1.389234324	6.47E-08
ENSSSCG00000013720	-1.389243964	8.00E-15
ENSSSCG00000033260	-1.389306402	0.005275872
ENSSSCG00000040763	-1.389978483	0.000168869
ENSSSCG00000032612	-1.390552519	4.91E-06
ENSSSCG00000035147	-1.391094913	2.18E-05
ENSSSCG00000008378	-1.391289052	1.92E-05
ENSSSCG00000024569	-1.391660639	1.69E-09
ENSSSCG00000016913	-1.392178935	0.012149764
ENSSSCG00000033351	-1.393064399	3.24E-07
ENSSSCG00000014083	-1.393967222	0.002098527
ENSSSCG00000041370	-1.394420458	0.009086808
ENSSSCG00000013777	-1.39546614	1.47E-12
ENSSSCG00000042625	-1.396692332	0.001773137
ENSSSCG00000029228	-1.40135036	8.87E-12
ENSSSCG00000006936	-1.402023277	1.98E-05
ENSSSCG00000026585	-1.403200952	4.05E-06
ENSSSCG00000016034	-1.40338027	9.70E-09
ENSSSCG00000035507	-1.403649848	0.001098917
ENSSSCG00000034879	-1.404043136	0.003385657
ENSSSCG00000009951	-1.404199168	0.007291943
ENSSSCG00000030626	-1.404564608	0.003794409
ENSSSCG00000021793	-1.406354233	1.93E-08
ENSSSCG00000031537	-1.406873169	5.14E-05
ENSSSCG00000016943	-1.408205253	1.26E-06
ENSSSCG00000038801	-1.409608291	5.19E-07
ENSSSCG00000032673	-1.410082381	9.60E-07
ENSSSCG00000036442	-1.411702382	0.000421417
ENSSSCG00000006810	-1.41174485	0.002564286

ENSSSCG00000045634	-1.411934368	0.014696805
ENSSSCG00000038927	-1.411989867	2.62E-06
ENSSSCG00000039956	-1.412833205	0.000674937
ENSSSCG00000031633	-1.412954628	2.84E-05
ENSSSCG00000010533	-1.413003877	8.18E-10
ENSSSCG00000002028	-1.414040166	0.010537633
ENSSSCG00000007659	-1.414278004	0.000234733
ENSSSCG00000012126	-1.416019441	2.46E-05
ENSSSCG00000002412	-1.416854678	3.19E-07
ENSSSCG00000002292	-1.417563302	0.002691048
ENSSSCG00000032585	-1.419918557	4.34E-10
ENSSSCG00000045563	-1.420384703	1.59E-05
ENSSSCG00000049823	-1.420584948	0.001047891
ENSSSCG00000001537	-1.421004457	6.77E-11
ENSSSCG00000036237	-1.42127902	4.60E-07
ENSSSCG00000024022	-1.422904121	3.64E-08
ENSSSCG00000041922	-1.423942271	0.002261002
ENSSSCG00000042867	-1.425435964	0.010526787
ENSSSCG00000040822	-1.425917524	0.002603076
ENSSSCG00000048201	-1.426150241	3.17E-06
ENSSSCG00000011023	-1.42716765	2.04E-06
ENSSSCG00000049976	-1.427414218	0.000869414
ENSSSCG00000042355	-1.427608916	0.000309141
ENSSSCG00000001459	-1.427686938	7.90E-13
ENSSSCG00000031531	-1.427877652	0.005680224
ENSSSCG00000009090	-1.429067231	1.69E-07
ENSSSCG00000051070	-1.429177911	0.008052355
ENSSSCG00000005225	-1.431528542	2.18E-07
ENSSSCG00000024800	-1.43205325	0.010375082
ENSSSCG00000023133	-1.434425244	1.56E-06
ENSSSCG00000017938	-1.434787525	0.000131966
ENSSSCG00000041410	-1.43616349	2.65E-06
ENSSSCG00000041490	-1.436285933	0.006870277
ENSSSCG00000042472	-1.436501681	8.80E-07
ENSSSCG00000012300	-1.437056963	0.002026431
ENSSSCG00000012595	-1.437997494	0.001553872
ENSSSCG00000006894	-1.438765611	3.49E-07
ENSSSCG00000048170	-1.439827481	2.00E-06
ENSSSCG00000015045	-1.440055269	0.017506395
ENSSSCG00000050067	-1.440688215	7.31E-06
ENSSSCG00000047657	-1.440719847	0.003749802
ENSSSCG00000009007	-1.440740614	2.31E-09
ENSSSCG00000015387	-1.441063806	8.71E-12
ENSSSCG00000004082	-1.441380321	4.76E-05
ENSSSCG00000015144	-1.442840298	6.78E-12
ENSSSCG00000006897	-1.444063584	2.72E-08
ENSSSCG00000016117	-1.445063519	0.000238264
ENSSSCG00000049494	-1.445486504	0.003154229
ENSSSCG00000010052	-1.446473263	2.37E-10
ENSSSCG00000003854	-1.447082157	7.95E-06
ENSSSCG00000007618	-1.447410348	1.30E-09
ENSSSCG00000009486	-1.447938095	0.014219531
ENSSSCG00000021041	-1.447966674	5.68E-08
ENSSSCG00000004216	-1.448968426	3.30E-07
ENSSSCG00000008721	-1.450040738	2.18E-07
ENSSSCG00000040406	-1.451411498	7.59E-06
ENSSSCG00000048358	-1.451702999	3.36E-06
ENSSSCG00000027667	-1.452840233	2.38E-05

ENSSSCG00000012667	-1.453899578	0.002163806
ENSSSCG00000029375	-1.454395118	3.48E-08
ENSSSCG00000018045	-1.454439588	5.73E-17
ENSSSCG00000005713	-1.45452423	5.47E-07
ENSSSCG00000025686	-1.454745143	2.60E-10
ENSSSCG00000046593	-1.455802304	0.007655839
ENSSSCG00000012548	-1.456760232	0.012615074
ENSSSCG00000049923	-1.45849959	0.014500386
ENSSSCG00000015538	-1.458854192	9.57E-07
ENSSSCG00000041595	-1.459325792	0.014018409
ENSSSCG00000002507	-1.459647563	6.88E-09
ENSSSCG00000046263	-1.460104882	0.000200606
ENSSSCG00000009379	-1.4612655	0.00132811
ENSSSCG00000012125	-1.461616983	6.18E-08
ENSSSCG00000034191	-1.461625637	0.011537141
ENSSSCG00000051061	-1.461776837	1.20E-07
ENSSSCG00000001535	-1.462535341	9.97E-13
ENSSSCG00000047724	-1.464900247	0.01193727
ENSSSCG00000002554	-1.465439888	2.58E-05
ENSSSCG00000039066	-1.466160887	0.001107926
ENSSSCG00000005910	-1.467586939	0.001351922
ENSSSCG00000011553	-1.467945536	1.41E-10
ENSSSCG00000029843	-1.467993867	8.05E-09
ENSSSCG00000013252	-1.468112871	0.001198
ENSSSCG00000005502	-1.469712849	0.002987426
ENSSSCG00000034339	-1.469736316	2.32E-06
ENSSSCG00000007425	-1.470367031	2.26E-06
ENSSSCG00000049338	-1.470782648	2.44E-09
ENSSSCG00000030877	-1.471256863	4.00E-05
ENSSSCG00000047188	-1.471537323	0.010750771
ENSSSCG00000051091	-1.471581112	9.99E-06
ENSSSCG00000005235	-1.472226674	0.000341017
ENSSSCG00000051397	-1.472853982	0.004538389
ENSSSCG00000026699	-1.473867294	1.30E-07
ENSSSCG00000048710	-1.473972807	0.001116195
ENSSSCG00000016843	-1.47496247	5.71E-06
ENSSSCG00000015353	-1.475586803	0.000200342
ENSSSCG00000034607	-1.476996058	1.46E-05
ENSSSCG00000017128	-1.477648705	1.18E-10
ENSSSCG00000011941	-1.47769277	1.83E-08
ENSSSCG00000041163	-1.47805906	2.07E-08
ENSSSCG00000008318	-1.479433454	0.000840082
ENSSSCG00000011521	-1.479463835	0.01831197
ENSSSCG00000029365	-1.47981639	0.014545346
ENSSSCG00000050149	-1.480040151	9.29E-05
ENSSSCG00000036665	-1.480702746	1.35E-12
ENSSSCG00000015266	-1.48111938	7.43E-12
ENSSSCG00000017751	-1.482350446	0.002586371
ENSSSCG00000049970	-1.482571852	3.19E-06
ENSSSCG00000027466	-1.482681789	3.54E-08
ENSSSCG00000023229	-1.482963696	4.92E-12
ENSSSCG00000013715	-1.483819421	1.06E-09
ENSSSCG00000038580	-1.484632699	1.69E-12
ENSSSCG00000023162	-1.485047647	7.17E-09
ENSSSCG00000045156	-1.486189522	0.002629867
ENSSSCG00000021105	-1.486695024	8.12E-05
ENSSSCG00000010640	-1.487246903	0.00015136
ENSSSCG00000015595	-1.487857681	1.47E-09

ENSSSCG00000004412	-1.488077747	1.15E-14
ENSSSCG00000036086	-1.488877783	0.00022528
ENSSSCG00000049220	-1.488953902	5.76E-05
ENSSSCG00000016199	-1.489015684	1.54E-05
ENSSSCG00000008150	-1.489597322	0.006483954
ENSSSCG00000029170	-1.49053055	3.47E-06
ENSSSCG00000014599	-1.49060018	0.000394472
ENSSSCG00000036549	-1.490745949	1.30E-09
ENSSSCG00000015211	-1.491097371	1.72E-05
ENSSSCG00000022209	-1.492878998	0.006014319
ENSSSCG00000041180	-1.493605934	0.000313158
ENSSSCG00000045895	-1.495004849	1.05E-05
ENSSSCG00000037364	-1.49510217	0.00010559
ENSSSCG00000014766	-1.495592471	2.53E-05
ENSSSCG00000035060	-1.496046352	7.65E-05
ENSSSCG00000003236	-1.496927371	1.05E-13
ENSSSCG00000005203	-1.497968181	3.83E-06
ENSSSCG00000007067	-1.498048291	3.98E-08
ENSSSCG00000008614	-1.498094649	1.24E-15
ENSSSCG00000046442	-1.499063343	2.03E-09
ENSSSCG00000000875	-1.499821595	3.04E-06
ENSSSCG00000004402	-1.500118812	0.012637662
ENSSSCG00000031300	-1.500621632	1.67E-08
ENSSSCG00000030172	-1.500837439	6.46E-07
ENSSSCG00000037096	-1.503094391	0.000120602
ENSSSCG00000005122	-1.503116402	3.86E-05
ENSSSCG00000021222	-1.503151908	0.000479367
ENSSSCG00000037067	-1.505411478	1.44E-14
ENSSSCG00000039860	-1.506597036	1.70E-10
ENSSSCG00000037674	-1.508409204	7.09E-09
ENSSSCG00000001202	-1.509068449	6.67E-07
ENSSSCG00000022318	-1.510123563	3.24E-05
ENSSSCG00000010340	-1.510318381	1.01E-06
ENSSSCG00000004520	-1.510482997	0.018209419
ENSSSCG00000036123	-1.511058748	0.009213448
ENSSSCG00000047544	-1.511383434	0.00220572
ENSSSCG00000048820	-1.511817201	0.012964327
ENSSSCG00000010581	-1.511942642	0.000617794
ENSSSCG00000017143	-1.512129417	1.55E-11
ENSSSCG00000041758	-1.513043332	0.002489489
ENSSSCG00000017990	-1.513488494	1.76E-07
ENSSSCG00000000682	-1.51366596	0.000112539
ENSSSCG00000044001	-1.514954926	6.46E-05
ENSSSCG00000007058	-1.51601698	3.46E-07
ENSSSCG00000051566	-1.517333819	0.017439159
ENSSSCG00000038693	-1.517974586	1.43E-07
ENSSSCG00000027665	-1.51851642	1.12E-11
ENSSSCG00000040503	-1.519219576	5.95E-09
ENSSSCG00000030337	-1.519623273	0.000959839
ENSSSCG00000016665	-1.519949458	7.07E-05
ENSSSCG00000050065	-1.519966914	0.012304181
ENSSSCG00000051610	-1.520009004	0.00092789
ENSSSCG00000023909	-1.520016588	0.000153847
ENSSSCG00000018084	-1.520778741	0.000101434
ENSSSCG00000011717	-1.52171595	0.005570615
ENSSSCG00000045569	-1.523168923	0.004771753
ENSSSCG00000004570	-1.523823528	4.53E-13
ENSSSCG00000035554	-1.524348002	0.014347835

ENSSSCG00000040789	-1.524864904	0.011434416
ENSSSCG00000003986	-1.525068955	6.66E-10
ENSSSCG00000028101	-1.526700369	5.84E-08
ENSSSCG00000038711	-1.526823227	2.15E-13
ENSSSCG00000048584	-1.527119587	0.000499562
ENSSSCG00000038811	-1.527183597	0.002296477
ENSSSCG00000015368	-1.527606672	1.27E-12
ENSSSCG00000042027	-1.527700698	0.00101051
ENSSSCG00000025430	-1.527824217	4.10E-12
ENSSSCG00000012112	-1.527974762	0.000226247
ENSSSCG00000003995	-1.5280378	4.13E-08
ENSSSCG00000012838	-1.528317912	2.64E-07
ENSSSCG00000051345	-1.529642448	8.62E-06
ENSSSCG00000028218	-1.530418264	0.000527099
ENSSSCG00000001657	-1.531158457	1.13E-09
ENSSSCG00000030833	-1.532168851	0.001146897
ENSSSCG00000040769	-1.532440751	0.000122123
ENSSSCG00000042938	-1.532805418	0.014925668
ENSSSCG00000014097	-1.533148443	3.87E-06
ENSSSCG00000043089	-1.536178524	8.43E-08
ENSSSCG00000027197	-1.536246494	2.71E-14
ENSSSCG00000027124	-1.538125335	7.99E-07
ENSSSCG00000014832	-1.538310846	0.008886608
ENSSSCG00000041500	-1.538390942	0.003728138
ENSSSCG00000006143	-1.540091914	8.25E-06
ENSSSCG00000032583	-1.541311347	7.53E-17
ENSSSCG00000022083	-1.542328486	0.004844781
ENSSSCG00000047264	-1.543120965	0.000465004
ENSSSCG00000023788	-1.543819585	0.000423944
ENSSSCG00000016569	-1.546221912	1.41E-08
ENSSSCG00000024810	-1.546962287	2.10E-10
ENSSSCG00000003881	-1.547046679	0.001571838
ENSSSCG00000003768	-1.549076448	7.47E-05
ENSSSCG00000026098	-1.549364002	6.39E-05
ENSSSCG00000001667	-1.549585997	6.43E-05
ENSSSCG00000004898	-1.549848631	1.27E-09
ENSSSCG00000045015	-1.550602149	0.001544279
ENSSSCG00000007987	-1.550983179	2.18E-07
ENSSSCG00000015638	-1.551928546	1.53E-17
ENSSSCG00000046331	-1.552832069	0.0003487
ENSSSCG00000033456	-1.553781851	7.84E-07
ENSSSCG00000015083	-1.553997242	1.05E-08
ENSSSCG00000020941	-1.554897498	3.20E-05
ENSSSCG00000040985	-1.555487358	6.99E-11
ENSSSCG00000029576	-1.555644714	1.17E-12
ENSSSCG00000018069	-1.556635062	1.10E-11
ENSSSCG00000035952	-1.556944242	4.06E-11
ENSSSCG00000014336	-1.561169775	7.23E-09
ENSSSCG00000015917	-1.56226528	0.000326805
ENSSSCG00000008694	-1.562416551	9.36E-07
ENSSSCG00000014224	-1.56331655	1.16E-07
ENSSSCG00000031149	-1.564743138	0.004556039
ENSSSCG00000051548	-1.564848212	7.31E-10
ENSSSCG00000024396	-1.565278861	4.38E-08
ENSSSCG00000023924	-1.565873673	9.56E-05
ENSSSCG00000023814	-1.566988147	0.000264295
ENSSSCG00000047487	-1.567812381	0.003275489
ENSSSCG00000005997	-1.568047603	0.000127746

ENSSSCG00000004744	-1.569811142	7.23E-09
ENSSSCG00000041636	-1.56984553	0.002079129
ENSSSCG00000039217	-1.570454004	0.002117601
ENSSSCG00000028148	-1.571319583	4.22E-07
ENSSSCG00000040579	-1.571635509	1.35E-12
ENSSSCG00000045507	-1.571794307	0.000655812
ENSSSCG00000002267	-1.572151729	1.83E-05
ENSSSCG00000004379	-1.572514679	5.44E-06
ENSSSCG00000047129	-1.573662394	3.16E-10
ENSSSCG00000021888	-1.574917981	1.31E-06
ENSSSCG00000016570	-1.575317195	3.13E-09
ENSSSCG00000040911	-1.575606042	0.002811805
ENSSSCG00000042951	-1.576244226	0.000185386
ENSSSCG00000043940	-1.577562254	2.71E-09
ENSSSCG00000041360	-1.577688672	0.001470387
ENSSSCG00000033546	-1.577740355	3.03E-07
ENSSSCG00000042473	-1.57857226	0.003320246
ENSSSCG00000003821	-1.579147672	4.47E-05
ENSSSCG00000048534	-1.579788932	0.010966546
ENSSSCG00000011294	-1.581300566	0.006181675
ENSSSCG00000012049	-1.583976741	0.000308426
ENSSSCG00000039159	-1.586078351	1.06E-06
ENSSSCG00000014904	-1.586091319	0.002151043
ENSSSCG00000046685	-1.586562772	0.000197379
ENSSSCG00000051329	-1.586960113	4.51E-08
ENSSSCG00000041701	-1.587915732	0.000984821
ENSSSCG00000031831	-1.588676475	3.20E-10
ENSSSCG00000000660	-1.588830907	4.87E-09
ENSSSCG00000040728	-1.590100742	0.004083822
ENSSSCG00000015882	-1.590935328	1.50E-13
ENSSSCG00000024223	-1.591783952	3.05E-09
ENSSSCG00000001052	-1.592039829	1.08E-10
ENSSSCG00000018065	-1.592172277	5.67E-10
ENSSSCG00000009824	-1.592328375	0.000155584
ENSSSCG00000041238	-1.59263826	5.49E-07
ENSSSCG00000051715	-1.592965395	8.99E-07
ENSSSCG00000025260	-1.593111839	3.39E-05
ENSSSCG00000002037	-1.593743327	8.22E-14
ENSSSCG00000047463	-1.593882425	0.018285617
ENSSSCG00000004010	-1.594166633	5.85E-10
ENSSSCG00000047591	-1.594722087	0.008054801
ENSSSCG00000032354	-1.596478317	1.98E-13
ENSSSCG00000002935	-1.596650311	1.45E-11
ENSSSCG00000032902	-1.597706134	1.82E-06
ENSSSCG00000044242	-1.599886698	0.003020931
ENSSSCG00000010426	-1.600216903	1.21E-08
ENSSSCG00000048062	-1.600296129	0.000204145
ENSSSCG00000000062	-1.600784786	0.000153876
ENSSSCG00000002275	-1.60114804	0.00039504
ENSSSCG00000029744	-1.604006714	0.015866024
ENSSSCG00000042941	-1.606016616	0.000644227
ENSSSCG00000050163	-1.606587277	0.011308925
ENSSSCG00000050663	-1.607641388	0.003493812
ENSSSCG00000038452	-1.60839476	0.000147184
ENSSSCG00000037892	-1.608699993	0.018637562
ENSSSCG00000002531	-1.609121758	0.0001712
ENSSSCG00000000517	-1.609294859	5.80E-05
ENSSSCG00000008646	-1.60959578	2.24E-06

ENSSSCG00000047383	-1.609598634	7.53E-08
ENSSSCG00000048932	-1.609879083	5.34E-05
ENSSSCG00000008576	-1.609988281	1.97E-14
ENSSSCG00000034567	-1.611673087	0.000485725
ENSSSCG00000041144	-1.613187658	0.007047855
ENSSSCG00000007288	-1.613630564	1.77E-12
ENSSSCG00000007955	-1.614016873	3.63E-16
ENSSSCG00000044899	-1.61432254	0.001421817
ENSSSCG00000048791	-1.614955288	1.23E-06
ENSSSCG00000015937	-1.615358161	8.66E-05
ENSSSCG00000013658	-1.616177239	1.66E-13
ENSSSCG00000029395	-1.617512793	0.00011232
ENSSSCG00000040109	-1.618030604	3.89E-12
ENSSSCG00000032036	-1.619567073	0.011584353
ENSSSCG00000004586	-1.620447983	0.001103398
ENSSSCG00000051391	-1.62218989	0.002981753
ENSSSCG00000011249	-1.622551362	0.002743277
ENSSSCG00000007648	-1.624103181	0.000338901
ENSSSCG00000034178	-1.624485561	9.64E-05
ENSSSCG00000034569	-1.624912247	5.72E-05
ENSSSCG00000005346	-1.626094915	1.15E-14
ENSSSCG00000009060	-1.626291537	9.65E-05
ENSSSCG00000028635	-1.626547955	6.17E-15
ENSSSCG00000010046	-1.626615196	9.26E-07
ENSSSCG00000047017	-1.627375749	0.001343814
ENSSSCG00000017880	-1.627505756	0.011220857
ENSSSCG00000051675	-1.627662173	0.008270115
ENSSSCG00000011782	-1.627992288	1.07E-08
ENSSSCG00000032522	-1.62882868	9.52E-09
ENSSSCG00000011867	-1.629400423	5.77E-10
ENSSSCG00000001486	-1.629651302	7.42E-13
ENSSSCG00000007949	-1.630366861	0.01068015
ENSSSCG00000017238	-1.634455958	8.39E-16
ENSSSCG00000031361	-1.634593901	1.39E-11
ENSSSCG00000048651	-1.634959092	0.000280166
ENSSSCG00000028048	-1.635429759	3.82E-06
ENSSSCG00000006693	-1.639034801	1.33E-05
ENSSSCG00000035590	-1.64029107	0.018506114
ENSSSCG00000041869	-1.641152909	3.03E-06
ENSSSCG00000004779	-1.642704288	5.81E-12
ENSSSCG00000005316	-1.642917393	2.81E-09
ENSSSCG00000047767	-1.64293088	0.000187765
ENSSSCG00000001716	-1.643306259	2.73E-11
ENSSSCG00000038025	-1.643324	2.00E-12
ENSSSCG00000047595	-1.64338131	0.013068089
ENSSSCG00000043406	-1.643899207	2.52E-05
ENSSSCG00000006947	-1.644039595	0.0006867
ENSSSCG00000015283	-1.644222853	9.83E-19
ENSSSCG00000037432	-1.645584372	0.018715534
ENSSSCG00000041405	-1.646103632	0.003321945
ENSSSCG00000041461	-1.646753021	0.000940044
ENSSSCG00000045392	-1.647333517	0.000943958
ENSSSCG00000033059	-1.647398351	0.000975111
ENSSSCG00000036534	-1.647877449	0.003107916
ENSSSCG00000017511	-1.648048858	0.001328878
ENSSSCG00000002424	-1.648947541	3.91E-06
ENSSSCG00000006525	-1.649151062	0.007032303
ENSSSCG00000027745	-1.649921813	2.08E-10

ENSSSCG00000002276	-1.65003632	1.84E-15
ENSSSCG00000040105	-1.650488847	0.008781181
ENSSSCG00000003088	-1.651152204	2.27E-05
ENSSSCG00000040448	-1.651220992	0.00408211
ENSSSCG00000016613	-1.651892653	4.14E-10
ENSSSCG00000027941	-1.652318003	0.000316743
ENSSSCG00000011915	-1.653630197	0.007336396
ENSSSCG00000047147	-1.653691688	0.000124714
ENSSSCG00000025208	-1.654727707	0.000767084
ENSSSCG00000027741	-1.655546217	4.05E-08
ENSSSCG00000050618	-1.655639577	9.80E-10
ENSSSCG00000010651	-1.656195415	9.16E-16
ENSSSCG00000032785	-1.656399435	0.010083781
ENSSSCG00000015866	-1.658008953	3.49E-05
ENSSSCG00000051794	-1.659043038	1.36E-12
ENSSSCG00000000680	-1.659066775	2.57E-05
ENSSSCG00000007171	-1.660549474	1.72E-06
ENSSSCG00000016831	-1.662304958	0.000636858
ENSSSCG00000008147	-1.663421632	1.69E-14
ENSSSCG00000047634	-1.664412173	0.002170855
ENSSSCG00000013865	-1.664964634	0.000282223
ENSSSCG00000006344	-1.665590977	0.003424517
ENSSSCG00000051231	-1.669082794	5.69E-05
ENSSSCG00000011598	-1.670022094	7.64E-09
ENSSSCG00000014428	-1.670284611	0.000428972
ENSSSCG00000016453	-1.671105507	6.89E-08
ENSSSCG00000042150	-1.671854277	0.000181284
ENSSSCG00000002741	-1.673811675	2.61E-08
ENSSSCG00000021255	-1.674152304	3.11E-09
ENSSSCG00000008888	-1.674390021	0.005888447
ENSSSCG00000039395	-1.677143082	2.62E-05
ENSSSCG00000004529	-1.677721859	2.16E-15
ENSSSCG00000009666	-1.679589916	8.55E-08
ENSSSCG00000050954	-1.68025113	6.40E-07
ENSSSCG00000000546	-1.680482077	7.92E-07
ENSSSCG00000048279	-1.680571304	0.000512946
ENSSSCG00000015287	-1.680573729	7.68E-11
ENSSSCG00000037865	-1.680622364	8.62E-05
ENSSSCG00000023156	-1.681018476	0.00994983
ENSSSCG00000010577	-1.681149753	0.002807252
ENSSSCG00000040419	-1.681466309	9.24E-06
ENSSSCG00000005616	-1.682549852	1.04E-09
ENSSSCG00000042982	-1.684864988	1.47E-07
ENSSSCG00000011401	-1.685579791	5.74E-07
ENSSSCG00000038384	-1.685823317	0.000146014
ENSSSCG00000015448	-1.687207284	0.000458124
ENSSSCG00000023627	-1.689687693	0.000540451
ENSSSCG00000050565	-1.690011875	0.003900399
ENSSSCG00000014047	-1.691781991	1.73E-12
ENSSSCG00000038557	-1.692038271	0.005577454
ENSSSCG00000007901	-1.693089688	6.60E-16
ENSSSCG00000034211	-1.693668	2.23E-13
ENSSSCG00000000455	-1.694195533	2.24E-09
ENSSSCG00000030112	-1.695353812	0.000765185
ENSSSCG00000049521	-1.695617257	0.001364687
ENSSSCG00000017540	-1.695891539	0.013271184
ENSSSCG00000043202	-1.696118824	0.002111125
ENSSSCG00000051243	-1.697223227	2.79E-09

ENSSSCG00000004795	-1.697242402	1.65E-08
ENSSSCG00000017415	-1.697245577	8.54E-05
ENSSSCG00000018078	-1.698051888	8.59E-09
ENSSSCG00000033122	-1.698579145	0.000893721
ENSSSCG00000042021	-1.699942153	0.012141297
ENSSSCG00000016437	-1.701296539	1.08E-06
ENSSSCG00000028892	-1.702048271	9.68E-10
ENSSSCG00000045707	-1.702533388	0.004164419
ENSSSCG00000005588	-1.70482312	1.55E-09
ENSSSCG00000042200	-1.705603084	0.00072202
ENSSSCG00000006635	-1.705933416	1.48E-08
ENSSSCG00000050979	-1.706125152	9.47E-08
ENSSSCG00000015878	-1.707459371	1.92E-12
ENSSSCG00000047765	-1.708556836	0.006965841
ENSSSCG00000045226	-1.710734823	9.46E-06
ENSSSCG00000037098	-1.71181295	0.003706737
ENSSSCG00000012637	-1.711903867	4.03E-06
ENSSSCG00000046400	-1.712015795	1.43E-05
ENSSSCG00000031703	-1.712426575	0.000356811
ENSSSCG00000025271	-1.713718886	2.10E-05
ENSSSCG00000025106	-1.713914208	1.01E-06
ENSSSCG00000049481	-1.714614892	0.001000234
ENSSSCG00000031321	-1.715182526	9.57E-14
ENSSSCG00000043523	-1.71549792	0.005496527
ENSSSCG00000035849	-1.715849228	0.002241933
ENSSSCG00000035750	-1.71763911	1.72E-06
ENSSSCG00000044472	-1.718222173	1.97E-12
ENSSSCG00000005284	-1.718396747	1.24E-09
ENSSSCG00000037001	-1.718453274	0.000121722
ENSSSCG00000004401	-1.719136941	0.011367398
ENSSSCG00000049426	-1.719257215	2.13E-07
ENSSSCG00000047720	-1.719366612	0.001532939
ENSSSCG00000002003	-1.719815367	9.94E-05
ENSSSCG00000048765	-1.719920027	0.000160502
ENSSSCG00000043840	-1.719995569	0.000492646
ENSSSCG00000015039	-1.72002543	8.43E-07
ENSSSCG00000016599	-1.720418117	0.007117578
ENSSSCG00000051065	-1.720747307	0.001685034
ENSSSCG00000039013	-1.721587617	0.000235774
ENSSSCG00000046746	-1.7225092	0.002994993
ENSSSCG00000008535	-1.72265903	2.94E-08
ENSSSCG00000048909	-1.722867765	0.000335276
ENSSSCG00000015203	-1.725124098	1.93E-11
ENSSSCG00000038829	-1.725804244	2.82E-10
ENSSSCG00000038323	-1.726124376	1.47E-06
ENSSSCG00000011729	-1.728660405	8.91E-06
ENSSSCG00000041745	-1.73060761	5.07E-09
ENSSSCG00000040184	-1.730710583	3.67E-05
ENSSSCG00000036804	-1.731216686	3.87E-06
ENSSSCG00000022849	-1.731400629	1.28E-08
ENSSSCG00000006183	-1.732215245	4.22E-10
ENSSSCG00000042769	-1.735819929	0.001601854
ENSSSCG00000014853	-1.736919752	4.12E-11
ENSSSCG00000005762	-1.738630033	1.58E-12
ENSSSCG00000039704	-1.73952188	0.000177557
ENSSSCG00000034990	-1.740918905	1.26E-10
ENSSSCG00000022989	-1.740919644	0.004564597
ENSSSCG00000034516	-1.741644063	1.51E-11

ENSSSCG00000021569	-1.741976765	3.26E-15
ENSSSCG00000034566	-1.743115843	8.59E-05
ENSSSCG00000036060	-1.743300766	2.15E-06
ENSSSCG00000047757	-1.743780624	3.45E-13
ENSSSCG00000024403	-1.745598539	2.22E-10
ENSSSCG00000021612	-1.746273113	0.000187092
ENSSSCG00000006631	-1.746278803	0.00246282
ENSSSCG00000004886	-1.746764102	0.017450139
ENSSSCG00000041438	-1.747416967	9.56E-06
ENSSSCG00000036452	-1.748408769	0.002410486
ENSSSCG00000009489	-1.750083636	1.21E-05
ENSSSCG00000051118	-1.750582696	0.00790491
ENSSSCG00000025005	-1.751187626	4.76E-10
ENSSSCG00000004199	-1.752473223	1.69E-14
ENSSSCG00000011895	-1.753948887	0.001298053
ENSSSCG00000008854	-1.754991718	4.95E-06
ENSSSCG00000026386	-1.755325735	1.84E-06
ENSSSCG00000003478	-1.757137517	3.22E-12
ENSSSCG00000003161	-1.759018985	2.66E-06
ENSSSCG00000048669	-1.760885979	6.83E-05
ENSSSCG00000004676	-1.761730428	2.14E-05
ENSSSCG00000004109	-1.761970007	4.04E-16
ENSSSCG00000025652	-1.762241867	5.06E-10
ENSSSCG00000009859	-1.762592792	3.28E-12
ENSSSCG00000026180	-1.762751515	6.22E-07
ENSSSCG00000005487	-1.763683499	4.06E-12
ENSSSCG00000026592	-1.763908261	1.39E-17
ENSSSCG00000011326	-1.764364643	3.96E-10
ENSSSCG00000013024	-1.764618164	0.002269578
ENSSSCG00000005110	-1.764908842	0.000205242
ENSSSCG00000017062	-1.764908983	1.39E-06
ENSSSCG00000022988	-1.765683585	1.88E-06
ENSSSCG00000013273	-1.766016747	2.43E-13
ENSSSCG00000040498	-1.766912681	0.000664111
ENSSSCG00000013546	-1.769280106	1.75E-24
ENSSSCG00000003709	-1.769759466	0.004680894
ENSSSCG00000027101	-1.771434083	1.00E-08
ENSSSCG00000032969	-1.772813043	2.88E-11
ENSSSCG00000018091	-1.772916375	2.46E-06
ENSSSCG00000045963	-1.774067803	0.008007479
ENSSSCG00000016083	-1.77408943	1.28E-05
ENSSSCG00000011723	-1.774450983	3.42E-05
ENSSSCG00000027916	-1.776430699	3.47E-17
ENSSSCG00000006958	-1.777166674	1.66E-06
ENSSSCG00000000531	-1.777596359	0.002349272
ENSSSCG00000033327	-1.780561453	1.62E-15
ENSSSCG00000045622	-1.781512842	0.003230912
ENSSSCG00000022676	-1.781745116	2.16E-05
ENSSSCG00000017818	-1.784014804	2.19E-10
ENSSSCG00000016448	-1.784923079	1.51E-15
ENSSSCG00000012238	-1.784997441	0.00225473
ENSSSCG00000016033	-1.785970121	0.000259761
ENSSSCG00000022986	-1.786158452	5.52E-05
ENSSSCG00000022919	-1.788126004	0.000299723
ENSSSCG00000006200	-1.789325473	0.000300276
ENSSSCG00000023848	-1.789645803	0.000981887
ENSSSCG00000011451	-1.79061388	0.009298986
ENSSSCG00000027378	-1.798586502	9.87E-07

ENSSSCG00000041231	-1.798740012	1.90E-06
ENSSSCG00000011885	-1.798931435	3.03E-09
ENSSSCG00000051012	-1.798999505	0.002684836
ENSSSCG00000047594	-1.801394446	0.000711921
ENSSSCG00000011587	-1.802765215	9.87E-05
ENSSSCG00000031889	-1.805824426	0.00021812
ENSSSCG00000030246	-1.806246818	5.03E-19
ENSSSCG00000037905	-1.80766838	6.86E-20
ENSSSCG00000010966	-1.808800051	2.19E-11
ENSSSCG00000024351	-1.809443133	3.43E-05
ENSSSCG00000026404	-1.810016877	0.012082361
ENSSSCG00000036013	-1.811680374	0.000342212
ENSSSCG00000000299	-1.812676379	3.41E-16
ENSSSCG00000037900	-1.81326927	3.65E-17
ENSSSCG00000042225	-1.813796377	0.000840583
ENSSSCG00000042092	-1.815629174	0.000797333
ENSSSCG00000005840	-1.816858046	0.000498493
ENSSSCG00000022429	-1.817204389	3.01E-11
ENSSSCG00000051680	-1.81742395	1.18E-05
ENSSSCG00000010133	-1.817632047	7.12E-13
ENSSSCG00000035189	-1.819111895	7.82E-11
ENSSSCG00000048314	-1.819535091	2.38E-08
ENSSSCG00000011346	-1.820706796	7.63E-19
ENSSSCG00000027796	-1.82075601	0.000133424
ENSSSCG00000041675	-1.821403054	0.000637055
ENSSSCG00000003148	-1.821445246	3.96E-09
ENSSSCG00000012911	-1.822039724	1.13E-11
ENSSSCG00000001517	-1.822092268	0.000134054
ENSSSCG00000010698	-1.822689136	1.55E-06
ENSSSCG00000045644	-1.823257916	0.012936834
ENSSSCG00000044280	-1.825003501	5.18E-08
ENSSSCG00000008392	-1.825374714	1.57E-17
ENSSSCG00000046740	-1.825951242	2.14E-05
ENSSSCG00000000709	-1.826001213	0.001679725
ENSSSCG00000046897	-1.826909087	1.29E-06
ENSSSCG00000026653	-1.828932451	5.21E-09
ENSSSCG00000012680	-1.829480447	0.009336914
ENSSSCG00000039161	-1.830165949	4.32E-13
ENSSSCG00000041324	-1.830760039	0.000328833
ENSSSCG00000051263	-1.830874959	0.008938131
ENSSSCG00000025238	-1.831066879	0.002806129
ENSSSCG00000037257	-1.832516766	3.31E-05
ENSSSCG00000045152	-1.833597535	1.10E-05
ENSSSCG00000040275	-1.838393328	1.78E-06
ENSSSCG00000014157	-1.840397825	1.56E-15
ENSSSCG00000042356	-1.841405269	9.47E-08
ENSSSCG00000021956	-1.84167038	4.87E-13
ENSSSCG00000044995	-1.844105257	0.000108632
ENSSSCG00000004714	-1.844247788	0.000641744
ENSSSCG00000047357	-1.845871899	0.000686343
ENSSSCG00000048254	-1.846288712	0.000397373
ENSSSCG00000001439	-1.84654674	4.27E-11
ENSSSCG00000031819	-1.846651853	8.61E-17
ENSSSCG00000044312	-1.846842401	0.00251724
ENSSSCG00000033232	-1.847893809	7.20E-12
ENSSSCG00000010593	-1.848988967	0.005815643
ENSSSCG00000005194	-1.849090183	0.010455231
ENSSSCG00000008294	-1.851158912	7.27E-06

ENSSSCG00000041710	-1.852064256	0.000296715
ENSSSCG00000037337	-1.853586156	2.32E-05
ENSSSCG00000027300	-1.855418702	0.001695527
ENSSSCG00000044254	-1.856610144	3.02E-05
ENSSSCG00000023028	-1.856796392	1.80E-06
ENSSSCG00000027505	-1.857664416	1.20E-05
ENSSSCG00000028327	-1.857738845	1.19E-15
ENSSSCG00000018034	-1.85853133	1.57E-06
ENSSSCG00000029553	-1.859388319	1.67E-08
ENSSSCG00000036933	-1.862419563	2.80E-13
ENSSSCG00000000588	-1.862805853	6.10E-06
ENSSSCG00000017994	-1.864344258	0.00845571
ENSSSCG00000051032	-1.864476613	0.008591097
ENSSSCG00000048752	-1.864691618	4.52E-05
ENSSSCG00000006082	-1.865026824	0.002109125
ENSSSCG00000017638	-1.866425758	6.09E-09
ENSSSCG00000043562	-1.866783796	8.46E-11
ENSSSCG00000002893	-1.867534169	2.34E-18
ENSSSCG00000044583	-1.868937937	0.002326787
ENSSSCG00000013086	-1.869230775	2.30E-13
ENSSSCG00000042294	-1.869774504	0.000908296
ENSSSCG00000013374	-1.869797825	0.005200211
ENSSSCG00000014007	-1.873383791	2.37E-05
ENSSSCG00000000983	-1.874405948	1.99E-20
ENSSSCG00000018081	-1.87476077	2.05E-13
ENSSSCG00000037539	-1.877045251	0.003806357
ENSSSCG00000022112	-1.878441863	7.02E-09
ENSSSCG00000024588	-1.878616105	1.92E-12
ENSSSCG00000000435	-1.878987948	6.32E-10
ENSSSCG00000015879	-1.881441526	8.34E-06
ENSSSCG00000047413	-1.881502339	1.46E-06
ENSSSCG00000010878	-1.881559353	2.27E-05
ENSSSCG00000035249	-1.883043194	1.15E-06
ENSSSCG00000015756	-1.886853085	0.00163562
ENSSSCG00000037766	-1.887536943	6.29E-08
ENSSSCG00000001131	-1.88793823	5.30E-14
ENSSSCG00000010071	-1.887941675	8.31E-13
ENSSSCG00000014441	-1.892080205	5.94E-20
ENSSSCG00000042849	-1.893385256	0.005089201
ENSSSCG00000015984	-1.894779236	0.002277732
ENSSSCG00000011477	-1.895464959	2.42E-06
ENSSSCG00000006233	-1.896927497	2.79E-11
ENSSSCG00000049203	-1.897049117	0.000184693
ENSSSCG00000035213	-1.898571929	6.55E-13
ENSSSCG00000011263	-1.899508962	4.48E-15
ENSSSCG00000006811	-1.899613958	2.59E-08
ENSSSCG00000022256	-1.900104362	3.04E-12
ENSSSCG00000003731	-1.900571285	7.15E-15
ENSSSCG00000032660	-1.901473143	6.38E-05
ENSSSCG00000003975	-1.901636467	8.05E-09
ENSSSCG00000044010	-1.902518294	9.80E-05
ENSSSCG00000044642	-1.90289749	0.001151386
ENSSSCG00000033217	-1.902908892	0.00591406
ENSSSCG00000010761	-1.904282724	7.93E-06
ENSSSCG00000009764	-1.906045471	5.59E-05
ENSSSCG00000015896	-1.906550421	2.85E-09
ENSSSCG00000034328	-1.909059712	2.43E-05
ENSSSCG00000044764	-1.910317226	5.02E-07

ENSSSCG00000008991	-1.910703311	0.005133322
ENSSSCG00000016245	-1.911396927	1.97E-08
ENSSSCG00000039561	-1.91282063	0.000930643
ENSSSCG00000045352	-1.914272305	0.003249444
ENSSSCG00000046838	-1.914345252	0.000438654
ENSSSCG00000015342	-1.914380339	8.18E-06
ENSSSCG00000013440	-1.915970957	0.004372274
ENSSSCG00000037479	-1.916379254	2.03E-08
ENSSSCG00000026729	-1.916999155	1.65E-18
ENSSSCG00000035152	-1.920112691	1.06E-18
ENSSSCG00000012975	-1.92094437	1.06E-06
ENSSSCG00000024552	-1.921538144	0.000214339
ENSSSCG00000013568	-1.922202306	2.73E-06
ENSSSCG00000032657	-1.923210152	0.000523299
ENSSSCG00000008606	-1.923378342	0.004599079
ENSSSCG00000017257	-1.923517407	1.86E-08
ENSSSCG00000042944	-1.923576541	0.017508355
ENSSSCG00000033003	-1.924302734	0.002677817
ENSSSCG00000003399	-1.92584068	0.007865437
ENSSSCG00000050642	-1.926375741	0.005276856
ENSSSCG00000031336	-1.928980501	1.50E-13
ENSSSCG00000045946	-1.929929692	0.012903631
ENSSSCG00000004202	-1.9303851	7.74E-11
ENSSSCG00000013618	-1.93079534	3.07E-08
ENSSSCG00000001440	-1.931197655	2.00E-09
ENSSSCG00000032341	-1.931223614	1.05E-05
ENSSSCG00000049943	-1.93194696	2.26E-06
ENSSSCG00000045470	-1.931964785	0.008307481
ENSSSCG00000050177	-1.933756632	6.90E-05
ENSSSCG00000004699	-1.934997613	4.86E-14
ENSSSCG00000007231	-1.935965484	0.001200813
ENSSSCG00000011355	-1.935978447	5.65E-19
ENSSSCG00000042717	-1.936830774	0.007787899
ENSSSCG00000047972	-1.939409517	0.00093827
ENSSSCG00000034609	-1.94042595	4.27E-06
ENSSSCG00000004644	-1.940568584	4.07E-05
ENSSSCG00000045495	-1.945004659	0.007243816
ENSSSCG00000015584	-1.946305496	0.016153594
ENSSSCG00000003302	-1.947736844	0.007084545
ENSSSCG00000049359	-1.947815799	1.28E-05
ENSSSCG00000021374	-1.949462764	4.34E-07
ENSSSCG00000044906	-1.950265834	0.003467966
ENSSSCG00000046599	-1.952431096	0.000385015
ENSSSCG00000022246	-1.952675874	4.52E-13
ENSSSCG00000031074	-1.953443059	8.58E-07
ENSSSCG00000042711	-1.955926075	0.012458233
ENSSSCG00000011878	-1.957318366	0.001019497
ENSSSCG00000051390	-1.958062469	0.001763294
ENSSSCG00000038701	-1.958257299	8.82E-13
ENSSSCG00000035983	-1.960302241	1.75E-09
ENSSSCG00000033542	-1.961846679	9.01E-06
ENSSSCG00000023915	-1.962555439	0.001822552
ENSSSCG00000043766	-1.96256453	1.62E-08
ENSSSCG00000018082	-1.963467834	1.35E-18
ENSSSCG00000012362	-1.963842471	8.03E-05
ENSSSCG00000039034	-1.964396833	1.61E-06
ENSSSCG00000036524	-1.965927864	6.56E-19
ENSSSCG00000017520	-1.966814413	0.007339212

ENSSSCG00000042279	-1.96739098	0.000850789
ENSSSCG00000031957	-1.967732077	0.000136142
ENSSSCG00000027952	-1.969037327	4.12E-14
ENSSSCG00000041198	-1.971394809	0.007639166
ENSSSCG00000037608	-1.971429934	1.30E-14
ENSSSCG00000012828	-1.975455842	8.96E-18
ENSSSCG00000004384	-1.975522861	3.53E-07
ENSSSCG00000003702	-1.977239198	1.12E-06
ENSSSCG00000014909	-1.977332035	5.40E-20
ENSSSCG00000049491	-1.979645228	0.01830574
ENSSSCG00000042379	-1.98063816	5.26E-05
ENSSSCG00000042811	-1.983517045	0.0028668
ENSSSCG00000011412	-1.983642598	3.34E-08
ENSSSCG00000049279	-1.984743655	0.007441042
ENSSSCG00000006413	-1.986811384	3.43E-07
ENSSSCG00000031356	-1.987380604	4.10E-10
ENSSSCG00000005457	-1.98803745	1.91E-21
ENSSSCG00000027417	-1.98883595	1.49E-21
ENSSSCG00000003351	-1.988878622	8.55E-06
ENSSSCG00000017534	-1.991561989	1.34E-09
ENSSSCG00000015037	-1.994863879	1.51E-08
ENSSSCG00000023592	-1.995569637	0.001060748
ENSSSCG00000000029	-1.996083466	5.71E-10
ENSSSCG00000013782	-1.996274131	0.001208071
ENSSSCG00000016751	-1.996485629	1.53E-08
ENSSSCG00000012153	-1.997643506	2.11E-05
ENSSSCG00000033624	-1.998510553	7.97E-11
ENSSSCG00000021601	-1.999060635	4.16E-09
ENSSSCG00000047865	-1.999855276	0.015279002
ENSSSCG00000028076	-2.000206681	9.37E-06
ENSSSCG00000003592	-2.00117185	2.03E-33
ENSSSCG00000008020	-2.002327811	6.62E-15
ENSSSCG00000008402	-2.00244279	7.68E-10
ENSSSCG00000015383	-2.002656399	2.56E-12
ENSSSCG00000032301	-2.002721592	0.004572788
ENSSSCG00000012376	-2.004911931	2.24E-13
ENSSSCG00000047907	-2.005410142	7.28E-05
ENSSSCG00000032403	-2.006714096	1.89E-05
ENSSSCG00000014943	-2.006784418	0.007399525
ENSSSCG00000000179	-2.007439124	2.83E-05
ENSSSCG00000041931	-2.008474035	0.01535083
ENSSSCG00000049692	-2.008505404	0.018640319
ENSSSCG00000014034	-2.008926783	3.33E-13
ENSSSCG00000003654	-2.014308062	1.51E-08
ENSSSCG00000013004	-2.015110814	0.000263557
ENSSSCG00000038077	-2.016546059	6.83E-06
ENSSSCG00000043745	-2.017207765	0.002517995
ENSSSCG00000043814	-2.020286556	8.22E-08
ENSSSCG00000003917	-2.020545892	1.32E-21
ENSSSCG00000001068	-2.020804437	0.000882184
ENSSSCG00000043571	-2.020946732	0.013525983
ENSSSCG00000049526	-2.021282755	0.011727625
ENSSSCG00000051075	-2.022028336	0.000383584
ENSSSCG00000003219	-2.022450771	1.07E-06
ENSSSCG00000014055	-2.022529674	0.00127313
ENSSSCG00000025188	-2.024544234	0.004869503
ENSSSCG00000005655	-2.025299712	6.56E-15
ENSSSCG00000044125	-2.027057762	0.015996902

ENSSSCG00000039368	-2.029692638	4.97E-14
ENSSSCG00000004458	-2.029744673	5.32E-10
ENSSSCG00000004628	-2.029806637	0.000904562
ENSSSCG00000048133	-2.032498538	0.002402237
ENSSSCG00000033286	-2.033125369	4.18E-06
ENSSSCG00000017885	-2.033429561	0.001575672
ENSSSCG00000022017	-2.03774619	1.81E-05
ENSSSCG00000026478	-2.037848707	3.61E-05
ENSSSCG00000049322	-2.037952295	0.013890319
ENSSSCG00000018087	-2.038267742	1.03E-21
ENSSSCG00000049552	-2.039656507	0.014402343
ENSSSCG00000031176	-2.042764964	1.71E-10
ENSSSCG00000004250	-2.043569139	0.017810432
ENSSSCG00000017873	-2.046211199	1.35E-32
ENSSSCG00000050207	-2.046572031	0.002087481
ENSSSCG00000031465	-2.046901147	0.004462806
ENSSSCG00000051601	-2.047309659	9.43E-07
ENSSSCG00000022161	-2.047845488	1.29E-23
ENSSSCG00000001458	-2.048523947	1.17E-09
ENSSSCG00000017053	-2.048649703	2.90E-05
ENSSSCG00000022738	-2.050039808	2.62E-13
ENSSSCG00000043155	-2.051515998	0.001766062
ENSSSCG00000017643	-2.054867241	6.60E-25
ENSSSCG00000037997	-2.054954234	5.26E-08
ENSSSCG00000029291	-2.057915661	1.39E-08
ENSSSCG00000043867	-2.058518749	0.003506818
ENSSSCG00000024293	-2.058520051	0.000534708
ENSSSCG00000033394	-2.058540182	0.001776094
ENSSSCG00000029227	-2.059226383	4.71E-11
ENSSSCG00000048677	-2.060072527	0.006624151
ENSSSCG00000017367	-2.060940551	5.74E-06
ENSSSCG00000001705	-2.061598797	5.31E-06
ENSSSCG00000007382	-2.063246625	0.007222025
ENSSSCG00000006245	-2.06328156	0.013793662
ENSSSCG00000050883	-2.064981986	0.002463329
ENSSSCG00000046103	-2.065854074	0.000103184
ENSSSCG00000013900	-2.067398604	2.04E-25
ENSSSCG00000014093	-2.067919905	1.58E-12
ENSSSCG00000050470	-2.068319041	0.001046784
ENSSSCG00000027178	-2.073103552	0.008069991
ENSSSCG00000000216	-2.074469337	5.88E-06
ENSSSCG00000038327	-2.075186197	2.97E-12
ENSSSCG00000026457	-2.077659936	0.002524526
ENSSSCG00000029651	-2.079420001	2.71E-06
ENSSSCG00000048325	-2.08028086	2.25E-06
ENSSSCG00000039414	-2.080888681	1.32E-07
ENSSSCG00000009672	-2.083364205	1.52E-06
ENSSSCG00000043957	-2.083643881	0.000160704
ENSSSCG00000043011	-2.084777803	9.02E-08
ENSSSCG00000033760	-2.08494249	1.49E-07
ENSSSCG00000042133	-2.085484547	0.00099239
ENSSSCG00000007644	-2.086143618	2.42E-22
ENSSSCG00000047516	-2.086516946	0.000860528
ENSSSCG00000042125	-2.08809326	9.92E-06
ENSSSCG00000014410	-2.090938884	0.006635305
ENSSSCG00000011538	-2.092580919	3.87E-14
ENSSSCG00000012724	-2.093362731	0.013280206
ENSSSCG00000024837	-2.096805827	4.53E-11

ENSSSCG00000050546	-2.098055778	0.00196175
ENSSSCG00000026417	-2.10062858	1.42E-06
ENSSSCG00000028051	-2.100821733	0.000119995
ENSSSCG00000051681	-2.101063215	1.86E-05
ENSSSCG00000009765	-2.101505585	4.58E-07
ENSSSCG00000033420	-2.102463377	1.95E-09
ENSSSCG00000017044	-2.102468647	0.003651145
ENSSSCG00000015753	-2.102837081	1.51E-09
ENSSSCG00000002520	-2.103735345	9.04E-16
ENSSSCG00000044418	-2.105767679	0.000369453
ENSSSCG00000021741	-2.10771351	6.53E-13
ENSSSCG00000051431	-2.108913511	0.011706217
ENSSSCG00000025345	-2.109915553	0.002920953
ENSSSCG00000003076	-2.109918551	1.71E-17
ENSSSCG00000026473	-2.112605509	3.24E-06
ENSSSCG00000018050	-2.113227931	1.61E-09
ENSSSCG00000049983	-2.114487176	0.000608461
ENSSSCG00000032416	-2.116094997	1.80E-11
ENSSSCG00000005922	-2.119751472	2.77E-07
ENSSSCG00000029541	-2.120201205	1.46E-19
ENSSSCG00000016652	-2.12072286	4.44E-06
ENSSSCG00000035595	-2.122245634	8.32E-12
ENSSSCG00000010559	-2.122920225	0.000245299
ENSSSCG00000017204	-2.124177278	7.37E-08
ENSSSCG00000045684	-2.126769499	0.002127803
ENSSSCG00000016991	-2.1281254	5.39E-10
ENSSSCG00000048122	-2.131099697	2.72E-08
ENSSSCG00000017607	-2.137437122	1.44E-10
ENSSSCG00000050816	-2.137692966	0.000111281
ENSSSCG00000050772	-2.137820924	2.36E-08
ENSSSCG00000038228	-2.138474104	0.000686565
ENSSSCG00000036261	-2.139460877	0.002737419
ENSSSCG00000050989	-2.139554041	0.001649414
ENSSSCG00000016705	-2.140435156	0.008699472
ENSSSCG00000018086	-2.141707028	3.68E-06
ENSSSCG00000030358	-2.142511456	4.65E-24
ENSSSCG00000041136	-2.144528998	0.001068081
ENSSSCG00000004758	-2.146151018	9.72E-08
ENSSSCG00000049170	-2.149692698	8.30E-06
ENSSSCG00000004008	-2.153592087	0.000107863
ENSSSCG00000037334	-2.153751796	3.22E-15
ENSSSCG00000039193	-2.154585219	0.007176998
ENSSSCG00000007650	-2.155764511	1.73E-06
ENSSSCG00000042398	-2.159606737	0.000228686
ENSSSCG00000050913	-2.160163269	0.001388966
ENSSSCG00000026924	-2.160551911	2.70E-13
ENSSSCG00000012787	-2.160588959	5.14E-10
ENSSSCG00000044115	-2.160668013	0.007937714
ENSSSCG00000014128	-2.162336175	0.006394678
ENSSSCG00000001572	-2.163056528	6.58E-17
ENSSSCG00000021075	-2.165296483	3.76E-05
ENSSSCG00000038264	-2.166630049	0.003259984
ENSSSCG00000031694	-2.166790097	0.000176673
ENSSSCG00000046446	-2.168167602	1.20E-05
ENSSSCG00000041984	-2.168420258	0.006615593
ENSSSCG00000013145	-2.169449105	7.99E-21
ENSSSCG00000051121	-2.1701146	0.013567696
ENSSSCG00000036996	-2.17202253	0.010307089

ENSSSCG00000015281	-2.173994384	4.52E-10
ENSSSCG00000051506	-2.174644723	1.18E-10
ENSSSCG00000035043	-2.174914868	1.18E-10
ENSSSCG00000010653	-2.175322971	0.001459272
ENSSSCG00000039841	-2.176677311	0.001524416
ENSSSCG00000027091	-2.177395732	0.000126734
ENSSSCG00000045723	-2.178596767	0.002173301
ENSSSCG00000050923	-2.180976437	0.008803227
ENSSSCG00000014672	-2.181671677	1.53E-33
ENSSSCG00000003715	-2.182999002	9.76E-05
ENSSSCG00000007094	-2.184088506	3.17E-15
ENSSSCG00000004973	-2.188510149	3.24E-09
ENSSSCG00000044206	-2.188842202	1.78E-11
ENSSSCG00000045069	-2.189212541	1.78E-09
ENSSSCG00000046607	-2.190903279	9.57E-09
ENSSSCG00000051266	-2.192279245	3.29E-13
ENSSSCG00000026345	-2.192642537	5.31E-05
ENSSSCG00000034776	-2.193281976	0.000325609
ENSSSCG00000007807	-2.195070115	6.39E-33
ENSSSCG00000010169	-2.196482368	4.90E-10
ENSSSCG00000035477	-2.198368687	0.003685338
ENSSSCG00000051710	-2.201642509	1.33E-13
ENSSSCG00000045050	-2.20330112	5.15E-05
ENSSSCG00000048896	-2.203694756	0.001867286
ENSSSCG00000010943	-2.205134476	8.83E-21
ENSSSCG00000000205	-2.205315995	2.04E-06
ENSSSCG00000005855	-2.206096141	8.39E-11
ENSSSCG00000009661	-2.206481068	7.73E-09
ENSSSCG00000028015	-2.207375288	3.05E-06
ENSSSCG00000009616	-2.207495463	3.83E-05
ENSSSCG00000046392	-2.208140986	0.001823389
ENSSSCG00000001567	-2.20867785	0.015749558
ENSSSCG00000000204	-2.208772608	1.80E-19
ENSSSCG00000014626	-2.209572713	2.36E-05
ENSSSCG00000035527	-2.211096733	6.03E-24
ENSSSCG00000042146	-2.212009968	0.010013362
ENSSSCG00000046159	-2.215486405	0.004401535
ENSSSCG00000017726	-2.216522519	0.011569165
ENSSSCG00000051030	-2.218200413	0.001787512
ENSSSCG00000032803	-2.219258751	2.03E-09
ENSSSCG00000046594	-2.220991951	0.001390801
ENSSSCG00000048334	-2.221822359	0.001807852
ENSSSCG00000008799	-2.222699942	1.70E-09
ENSSSCG00000010293	-2.224798091	0.008737728
ENSSSCG00000017101	-2.224829319	3.93E-09
ENSSSCG00000049685	-2.226818836	7.80E-05
ENSSSCG00000009322	-2.228545154	2.91E-27
ENSSSCG00000011216	-2.230256189	0.000596971
ENSSSCG00000006546	-2.230422884	1.94E-09
ENSSSCG00000043378	-2.231022867	2.38E-08
ENSSSCG00000000418	-2.231991163	0.001776156
ENSSSCG00000038706	-2.233592417	1.52E-12
ENSSSCG00000034891	-2.234128743	1.74E-05
ENSSSCG00000006159	-2.234714693	1.27E-07
ENSSSCG00000009613	-2.236294598	3.23E-06
ENSSSCG00000000605	-2.236442043	4.11E-09
ENSSSCG00000016131	-2.238846457	0.000784348
ENSSSCG00000041108	-2.240260844	3.08E-07

ENSSSCG00000013341	-2.240316796	0.002101865
ENSSSCG00000010592	-2.242413863	7.54E-08
ENSSSCG00000012780	-2.242792652	4.89E-13
ENSSSCG00000034441	-2.244044795	2.54E-20
ENSSSCG00000044568	-2.245080482	4.33E-08
ENSSSCG00000015332	-2.248068953	0.009488492
ENSSSCG00000018094	-2.250913292	6.67E-22
ENSSSCG00000003524	-2.252713779	4.37E-10
ENSSSCG00000039921	-2.252722431	2.02E-07
ENSSSCG00000043817	-2.253177365	1.19E-21
ENSSSCG00000023992	-2.253737898	4.01E-11
ENSSSCG00000044895	-2.25807351	0.000995351
ENSSSCG00000001901	-2.260342461	6.36E-08
ENSSSCG00000047029	-2.260821824	2.14E-06
ENSSSCG00000046730	-2.261924126	0.001408424
ENSSSCG00000042956	-2.262925238	0.000296516
ENSSSCG00000045393	-2.264392936	0.01168002
ENSSSCG00000025644	-2.265196776	1.62E-17
ENSSSCG00000043390	-2.265544691	1.87E-05
ENSSSCG00000039322	-2.265750253	2.22E-08
ENSSSCG00000038600	-2.268746992	2.17E-05
ENSSSCG00000017296	-2.269220589	1.21E-14
ENSSSCG00000016295	-2.269441464	0.016747928
ENSSSCG00000016579	-2.270096942	0.000239013
ENSSSCG00000010535	-2.270372204	0.002225746
ENSSSCG00000049038	-2.271152234	0.002004069
ENSSSCG00000040416	-2.272531284	3.04E-18
ENSSSCG00000027118	-2.272792126	7.28E-07
ENSSSCG00000038993	-2.27642952	0.003454692
ENSSSCG00000007199	-2.27745074	0.017224899
ENSSSCG00000011299	-2.277470651	6.10E-10
ENSSSCG00000009222	-2.281322713	1.22E-06
ENSSSCG00000010256	-2.283008668	5.44E-05
ENSSSCG00000011192	-2.283236634	4.48E-17
ENSSSCG00000050845	-2.285055759	5.58E-13
ENSSSCG00000002955	-2.285628571	3.85E-16
ENSSSCG00000041904	-2.285794138	0.001428044
ENSSSCG00000050703	-2.286120498	0.005019198
ENSSSCG00000042886	-2.287836064	5.08E-07
ENSSSCG00000009966	-2.287907401	5.18E-09
ENSSSCG00000041656	-2.293914986	0.006274573
ENSSSCG00000044610	-2.295149174	0.004076585
ENSSSCG00000033175	-2.295262234	4.66E-13
ENSSSCG00000006171	-2.295348499	0.00429784
ENSSSCG00000029388	-2.298582351	3.55E-10
ENSSSCG00000000857	-2.298716779	0.007642649
ENSSSCG00000048240	-2.300279172	0.006411837
ENSSSCG00000006243	-2.303153954	1.87E-05
ENSSSCG00000007849	-2.303971101	0.005200546
ENSSSCG00000046868	-2.306275276	0.010499315
ENSSSCG00000000741	-2.308093472	2.92E-08
ENSSSCG00000045551	-2.310366904	0.005511552
ENSSSCG00000021309	-2.313324712	1.40E-24
ENSSSCG00000028549	-2.314312593	1.18E-11
ENSSSCG00000000607	-2.314363945	6.93E-22
ENSSSCG00000001472	-2.31689424	2.66E-25
ENSSSCG00000042679	-2.321419627	0.014861208
ENSSSCG00000042618	-2.322029173	0.01580876

ENSSSCG00000006002	-2.322541318	9.88E-07
ENSSSCG00000049357	-2.322795328	0.001382304
ENSSSCG00000042241	-2.322804666	0.001667382
ENSSSCG00000038627	-2.325985469	0.015570318
ENSSSCG00000027935	-2.326001887	3.66E-06
ENSSSCG00000051307	-2.326282745	0.005239932
ENSSSCG00000049048	-2.326535089	5.50E-07
ENSSSCG00000017988	-2.327315131	1.11E-21
ENSSSCG00000013286	-2.327402003	1.10E-06
ENSSSCG00000042468	-2.331094319	1.70E-08
ENSSSCG00000003218	-2.331109819	1.55E-13
ENSSSCG00000009627	-2.333247906	5.35E-13
ENSSSCG00000047214	-2.334580707	7.64E-07
ENSSSCG00000050563	-2.335134739	0.003760678
ENSSSCG00000046261	-2.336693641	0.002745189
ENSSSCG00000032503	-2.339342621	0.001245514
ENSSSCG00000004215	-2.342039488	0.00271335
ENSSSCG00000002700	-2.344472031	0.007770309
ENSSSCG00000010639	-2.344734895	0.004824934
ENSSSCG00000022675	-2.345885851	2.14E-23
ENSSSCG00000044815	-2.349545677	3.08E-09
ENSSSCG00000014827	-2.34961293	6.84E-05
ENSSSCG00000030303	-2.35000539	8.19E-11
ENSSSCG00000051195	-2.351520464	0.000144026
ENSSSCG00000041824	-2.354391149	0.015327267
ENSSSCG00000017036	-2.354805616	5.08E-20
ENSSSCG00000023273	-2.355604344	0.000300815
ENSSSCG00000046658	-2.356232867	1.01E-08
ENSSSCG00000010212	-2.356665884	2.74E-10
ENSSSCG00000028139	-2.357152643	5.24E-08
ENSSSCG00000028529	-2.357599685	1.79E-12
ENSSSCG00000032019	-2.357636211	5.67E-13
ENSSSCG00000048120	-2.361353115	0.000577651
ENSSSCG00000049987	-2.362224933	0.002029775
ENSSSCG00000040629	-2.363174804	1.68E-15
ENSSSCG00000049107	-2.36397675	0.000975272
ENSSSCG00000051092	-2.364057585	0.009042576
ENSSSCG00000001988	-2.364606852	1.34E-41
ENSSSCG00000046002	-2.364650926	3.95E-05
ENSSSCG00000047701	-2.365832862	5.69E-05
ENSSSCG00000031503	-2.372620933	1.81E-10
ENSSSCG00000022309	-2.376841039	5.43E-21
ENSSSCG00000004962	-2.376927949	5.06E-15
ENSSSCG00000005828	-2.377345176	5.52E-10
ENSSSCG00000009221	-2.382063669	0.007856073
ENSSSCG00000009957	-2.38316129	0.006203559
ENSSSCG00000027454	-2.386436195	0.014874597
ENSSSCG00000011599	-2.386814197	7.36E-10
ENSSSCG00000022724	-2.387636682	0.007446993
ENSSSCG00000044456	-2.389950669	0.017996265
ENSSSCG00000047932	-2.392031994	1.56E-07
ENSSSCG00000024678	-2.392562125	1.35E-12
ENSSSCG00000016244	-2.393777881	2.90E-12
ENSSSCG00000031916	-2.39389897	3.60E-22
ENSSSCG00000013614	-2.399565231	4.20E-11
ENSSSCG00000004608	-2.400379495	0.00443709
ENSSSCG00000049014	-2.401811415	1.79E-05
ENSSSCG00000029606	-2.4028515	2.42E-15

ENSSSCG00000032443	-2.405666875	6.34E-23
ENSSSCG00000016522	-2.407462626	4.78E-11
ENSSSCG00000043215	-2.408031763	0.000102807
ENSSSCG00000022039	-2.408585015	8.38E-19
ENSSSCG00000007008	-2.40915576	0.002476831
ENSSSCG00000034633	-2.410356096	0.000140859
ENSSSCG00000049051	-2.414780849	0.002779347
ENSSSCG00000050858	-2.415524995	0.008451192
ENSSSCG00000041587	-2.41701047	4.29E-06
ENSSSCG00000037970	-2.418892139	5.20E-06
ENSSSCG00000002383	-2.419875185	3.99E-18
ENSSSCG00000050145	-2.42070284	0.005765499
ENSSSCG00000036223	-2.421235691	2.01E-08
ENSSSCG00000049690	-2.424533271	7.72E-05
ENSSSCG00000044102	-2.425739575	0.000544346
ENSSSCG00000016704	-2.425762938	0.001929041
ENSSSCG00000051549	-2.426098541	8.09E-05
ENSSSCG00000033185	-2.426232291	4.12E-12
ENSSSCG00000032330	-2.427351392	1.35E-17
ENSSSCG00000005311	-2.430224378	2.65E-21
ENSSSCG00000042730	-2.43239417	0.00201118
ENSSSCG00000043687	-2.432591635	1.11E-13
ENSSSCG00000042639	-2.432908418	0.009499371
ENSSSCG00000044437	-2.434328597	0.00013917
ENSSSCG00000027849	-2.43531097	1.29E-19
ENSSSCG00000048563	-2.435609022	0.016020899
ENSSSCG00000012485	-2.44015768	4.82E-09
ENSSSCG00000045358	-2.442864709	0.009686132
ENSSSCG00000044588	-2.444252625	3.01E-09
ENSSSCG00000033512	-2.446369173	1.41E-13
ENSSSCG00000044939	-2.446595242	2.53E-15
ENSSSCG00000028488	-2.448071667	3.08E-07
ENSSSCG00000008051	-2.450388281	2.10E-26
ENSSSCG00000026517	-2.454073046	6.19E-17
ENSSSCG00000034727	-2.454851835	4.55E-10
ENSSSCG00000045621	-2.455512324	0.000233829
ENSSSCG00000043359	-2.456459243	0.014779094
ENSSSCG00000004309	-2.458144398	0.008148921
ENSSSCG00000048816	-2.45856701	0.013536214
ENSSSCG00000045752	-2.461067189	9.11E-06
ENSSSCG00000015795	-2.462333365	6.51E-09
ENSSSCG00000009500	-2.463225724	3.48E-07
ENSSSCG00000045443	-2.467241882	4.54E-05
ENSSSCG00000003950	-2.468589074	4.24E-25
ENSSSCG00000050584	-2.471575153	0.017998405
ENSSSCG00000016426	-2.472565345	7.02E-29
ENSSSCG00000024134	-2.472796432	1.04E-16
ENSSSCG00000004530	-2.473286423	0.010065818
ENSSSCG00000039978	-2.473629096	1.68E-22
ENSSSCG00000014851	-2.47595297	4.33E-35
ENSSSCG00000033412	-2.476157558	9.06E-09
ENSSSCG00000011453	-2.477416376	6.20E-10
ENSSSCG00000028996	-2.478712748	1.24E-09
ENSSSCG00000000665	-2.482398724	7.48E-11
ENSSSCG00000026155	-2.483595055	4.25E-11
ENSSSCG00000002932	-2.486820883	9.30E-16
ENSSSCG00000050958	-2.488256355	6.29E-21
ENSSSCG00000035371	-2.488294488	5.48E-15

ENSSSCG00000004195	-2.491299164	2.78E-06
ENSSSCG00000007823	-2.494173016	0.003860388
ENSSSCG00000006890	-2.495981681	3.82E-09
ENSSSCG00000049077	-2.496459119	1.35E-13
ENSSSCG00000010926	-2.497939665	6.35E-25
ENSSSCG00000040746	-2.49838244	0.000441416
ENSSSCG00000015556	-2.498917674	2.41E-12
ENSSSCG00000046429	-2.503380602	0.000198183
ENSSSCG00000011455	-2.507984396	1.57E-24
ENSSSCG00000048856	-2.508264032	8.78E-09
ENSSSCG00000047204	-2.508807611	7.18E-07
ENSSSCG00000026387	-2.510478795	9.79E-08
ENSSSCG00000002788	-2.514868453	9.44E-05
ENSSSCG00000046097	-2.51577312	3.56E-07
ENSSSCG00000008135	-2.517090267	1.69E-12
ENSSSCG00000042787	-2.517480153	2.48E-14
ENSSSCG00000051575	-2.517860701	0.011550713
ENSSSCG00000050939	-2.518036401	1.73E-06
ENSSSCG00000044551	-2.518293673	0.003544167
ENSSSCG00000016330	-2.518303891	4.32E-28
ENSSSCG00000016402	-2.519601132	1.43E-05
ENSSSCG00000002026	-2.519664667	9.53E-11
ENSSSCG00000017251	-2.520107586	1.53E-13
ENSSSCG00000007170	-2.520394605	8.00E-11
ENSSSCG00000049223	-2.520881763	0.002018259
ENSSSCG00000027157	-2.523861352	4.88E-28
ENSSSCG00000046510	-2.525935399	0.002572113
ENSSSCG00000034069	-2.528750691	2.21E-05
ENSSSCG00000012941	-2.52950065	0.015206552
ENSSSCG00000035617	-2.530554375	5.46E-08
ENSSSCG00000003760	-2.533099912	2.54E-07
ENSSSCG00000033539	-2.533600788	5.82E-06
ENSSSCG00000022129	-2.535413112	1.20E-23
ENSSSCG00000022550	-2.53676727	1.33E-10
ENSSSCG00000008488	-2.539437773	2.69E-15
ENSSSCG00000011527	-2.542504107	2.88E-09
ENSSSCG00000022195	-2.542803213	4.45E-15
ENSSSCG00000038374	-2.542832941	1.16E-30
ENSSSCG00000015445	-2.542837045	0.012185663
ENSSSCG00000015862	-2.544543734	2.02E-23
ENSSSCG00000051016	-2.548643791	3.55E-07
ENSSSCG00000046798	-2.548876793	0.001104829
ENSSSCG00000046197	-2.552950914	6.63E-06
ENSSSCG00000041248	-2.55338092	3.26E-05
ENSSSCG00000040366	-2.553644429	2.22E-05
ENSSSCG00000038514	-2.553995953	1.93E-14
ENSSSCG00000051590	-2.554335081	0.001122089
ENSSSCG00000015911	-2.555289508	0.000806254
ENSSSCG00000038969	-2.556156189	3.18E-32
ENSSSCG00000016892	-2.55790228	0.000322849
ENSSSCG00000001582	-2.560551292	0.001055339
ENSSSCG00000000610	-2.568457841	3.25E-06
ENSSSCG00000042469	-2.572878256	0.000423582
ENSSSCG00000044539	-2.57609016	0.006869464
ENSSSCG00000045382	-2.577742375	7.62E-12
ENSSSCG00000017539	-2.578959274	1.13E-15
ENSSSCG00000012572	-2.579524029	2.25E-09
ENSSSCG00000037360	-2.579723472	3.00E-15

ENSSSCG00000037987	-2.580355303	0.016284469
ENSSSCG00000047660	-2.582370547	3.48E-05
ENSSSCG00000048212	-2.582716673	0.008208078
ENSSSCG00000029029	-2.58309586	5.99E-13
ENSSSCG00000042900	-2.584107526	5.50E-07
ENSSSCG00000014257	-2.585195545	8.34E-07
ENSSSCG00000017046	-2.595421822	4.06E-15
ENSSSCG00000013584	-2.595577537	0.00076103
ENSSSCG00000028350	-2.59595743	0.000562193
ENSSSCG00000001500	-2.596727176	2.15E-19
ENSSSCG00000007978	-2.597238358	4.24E-10
ENSSSCG00000036504	-2.599238037	1.37E-23
ENSSSCG00000016130	-2.599799582	0.008995071
ENSSSCG00000031796	-2.600240427	0.005919234
ENSSSCG00000050431	-2.601838787	0.000130987
ENSSSCG00000010428	-2.602676518	2.56E-06
ENSSSCG00000047843	-2.604999391	0.007422961
ENSSSCG00000045235	-2.605160419	0.016165497
ENSSSCG00000012002	-2.606647351	2.92E-17
ENSSSCG00000043131	-2.607920882	2.52E-05
ENSSSCG00000051196	-2.610157203	2.40E-07
ENSSSCG00000017879	-2.614136551	1.28E-24
ENSSSCG00000006121	-2.615007984	3.34E-05
ENSSSCG00000007986	-2.615266127	5.37E-05
ENSSSCG00000027351	-2.615523338	1.93E-13
ENSSSCG00000049288	-2.623827852	1.19E-08
ENSSSCG00000043395	-2.624701329	0.005447668
ENSSSCG00000042643	-2.630906454	4.85E-05
ENSSSCG00000012347	-2.633064302	6.58E-20
ENSSSCG00000034826	-2.633386879	6.61E-05
ENSSSCG00000016925	-2.634005605	2.15E-25
ENSSSCG00000001242	-2.636442996	1.03E-44
ENSSSCG00000023661	-2.638917556	0.000597881
ENSSSCG00000029960	-2.642138484	1.50E-07
ENSSSCG00000034266	-2.642985387	1.78E-09
ENSSSCG00000005699	-2.64333529	2.71E-06
ENSSSCG00000016428	-2.64623439	0.010773651
ENSSSCG00000036248	-2.646796653	0.000184293
ENSSSCG00000006187	-2.647548147	5.31E-30
ENSSSCG00000012286	-2.650677668	1.32E-22
ENSSSCG00000028674	-2.653827071	5.09E-24
ENSSSCG00000021997	-2.654449587	3.25E-11
ENSSSCG00000045353	-2.655850301	2.36E-12
ENSSSCG00000030511	-2.657285388	0.002670306
ENSSSCG00000008081	-2.657703544	0.001262616
ENSSSCG00000011400	-2.658206943	9.30E-14
ENSSSCG00000001806	-2.659295518	1.37E-05
ENSSSCG00000039271	-2.660657644	0.016353947
ENSSSCG00000049034	-2.660921669	0.006213457
ENSSSCG00000018656	-2.662311192	0.007536434
ENSSSCG00000009831	-2.664239437	5.84E-15
ENSSSCG00000017233	-2.664506628	1.25E-09
ENSSSCG00000023225	-2.667790484	0.001416505
ENSSSCG00000003455	-2.670875204	4.27E-10
ENSSSCG00000015901	-2.673376584	4.39E-10
ENSSSCG00000032190	-2.673798767	1.02E-06
ENSSSCG00000045667	-2.673952988	0.000196605
ENSSSCG00000005360	-2.67570071	7.08E-15

ENSSSCG00000000271	-2.679483543	4.65E-07
ENSSSCG00000016917	-2.680546068	0.000794989
ENSSSCG00000047248	-2.68100076	0.001564236
ENSSSCG00000006390	-2.684262162	3.13E-14
ENSSSCG00000050752	-2.686175363	0.004637729
ENSSSCG00000042989	-2.687795664	0.015363625
ENSSSCG00000036846	-2.688997903	0.000202661
ENSSSCG00000038710	-2.691401882	4.65E-30
ENSSSCG00000039095	-2.691643879	0.016825927
ENSSSCG00000040001	-2.692509567	1.53E-12
ENSSSCG00000005474	-2.693373538	0.0094665
ENSSSCG00000024191	-2.698285979	0.011231253
ENSSSCG00000009011	-2.698878249	4.60E-17
ENSSSCG00000010449	-2.700677827	2.40E-05
ENSSSCG00000044269	-2.701209653	0.016272645
ENSSSCG00000027890	-2.702132384	3.94E-20
ENSSSCG00000051039	-2.70415258	0.011910862
ENSSSCG00000051339	-2.705056151	0.005480479
ENSSSCG00000033043	-2.709195809	1.12E-06
ENSSSCG00000035927	-2.709765198	4.43E-06
ENSSSCG00000017349	-2.712426508	4.88E-23
ENSSSCG00000010055	-2.713986385	2.46E-17
ENSSSCG00000035525	-2.714478264	6.86E-07
ENSSSCG00000030655	-2.717136514	1.08E-07
ENSSSCG00000042006	-2.717296873	0.00085444
ENSSSCG00000047729	-2.718651593	0.003196354
ENSSSCG00000006396	-2.719529925	1.25E-13
ENSSSCG00000005756	-2.723597166	0.000285009
ENSSSCG00000011090	-2.723808803	1.09E-26
ENSSSCG00000043141	-2.725131656	0.006174083
ENSSSCG00000038869	-2.727440724	3.23E-05
ENSSSCG00000037918	-2.730926803	7.48E-06
ENSSSCG00000033093	-2.735677484	7.99E-23
ENSSSCG00000006160	-2.736714565	6.42E-36
ENSSSCG00000000854	-2.737291347	3.37E-16
ENSSSCG00000008169	-2.738869502	2.03E-30
ENSSSCG00000028237	-2.743453633	3.78E-06
ENSSSCG00000011892	-2.744366053	1.19E-10
ENSSSCG00000035711	-2.744864511	7.48E-07
ENSSSCG00000044004	-2.74711988	3.64E-36
ENSSSCG00000003156	-2.748769041	0.003101257
ENSSSCG00000033958	-2.749244976	4.48E-06
ENSSSCG00000048537	-2.756398258	4.64E-07
ENSSSCG00000012993	-2.758797479	0.000147312
ENSSSCG00000003368	-2.761947174	6.70E-09
ENSSSCG00000004143	-2.766876369	7.49E-07
ENSSSCG00000013073	-2.767371146	2.16E-36
ENSSSCG00000000146	-2.767496176	5.98E-15
ENSSSCG00000050553	-2.767973505	0.011015559
ENSSSCG00000050719	-2.770922291	0.003219688
ENSSSCG00000035445	-2.776340683	0.000184549
ENSSSCG00000032423	-2.778306947	0.000119107
ENSSSCG00000011890	-2.783003358	1.67E-08
ENSSSCG00000038584	-2.783241964	0.000368161
ENSSSCG00000050313	-2.783440213	0.006748785
ENSSSCG00000016676	-2.783712931	9.32E-06
ENSSSCG00000025711	-2.786542197	8.76E-22
ENSSSCG00000010210	-2.788632153	5.65E-06

ENSSSCG00000033268	-2.796442988	0.000444388
ENSSSCG00000042173	-2.797176274	1.28E-08
ENSSSCG00000041690	-2.799937994	2.14E-09
ENSSSCG00000033001	-2.802548432	1.73E-30
ENSSSCG00000051003	-2.80773176	0.002440643
ENSSSCG00000049185	-2.807786821	1.65E-05
ENSSSCG00000045794	-2.813659856	4.71E-29
ENSSSCG00000041149	-2.821895305	0.000573444
ENSSSCG00000026130	-2.823468915	0.012795648
ENSSSCG00000044703	-2.827900522	0.010082973
ENSSSCG00000036420	-2.828137293	0.002983292
ENSSSCG00000029281	-2.830537535	7.68E-09
ENSSSCG00000004545	-2.833959482	4.80E-05
ENSSSCG00000025834	-2.834744651	6.15E-07
ENSSSCG00000040053	-2.8384363	0.00012293
ENSSSCG00000036741	-2.83911935	0.000518692
ENSSSCG00000036572	-2.839135815	8.68E-13
ENSSSCG00000050888	-2.840668897	0.012880388
ENSSSCG00000015084	-2.840867879	0.00372534
ENSSSCG00000045560	-2.844169692	0.004265878
ENSSSCG00000021132	-2.844213157	0.005945077
ENSSSCG00000012512	-2.846847671	6.07E-06
ENSSSCG00000028784	-2.847297061	6.97E-09
ENSSSCG00000011397	-2.851771942	0.015403973
ENSSSCG00000049770	-2.851942512	0.001004766
ENSSSCG00000050564	-2.852763856	0.003294743
ENSSSCG00000015522	-2.856641299	1.32E-07
ENSSSCG00000041585	-2.859597886	1.28E-07
ENSSSCG00000045440	-2.86153084	0.001599634
ENSSSCG00000022390	-2.869979994	0.001049439
ENSSSCG00000005512	-2.870786261	0.000804996
ENSSSCG00000012700	-2.874989711	4.73E-19
ENSSSCG00000044072	-2.875813081	5.51E-08
ENSSSCG00000016129	-2.876234743	7.64E-08
ENSSSCG00000035996	-2.877369065	0.008128759
ENSSSCG00000042706	-2.87821313	2.71E-15
ENSSSCG00000028741	-2.881675748	6.71E-05
ENSSSCG00000041875	-2.885123093	0.014385832
ENSSSCG00000027991	-2.886904493	1.53E-11
ENSSSCG00000012944	-2.888362865	3.97E-15
ENSSSCG00000017121	-2.888523204	1.10E-28
ENSSSCG00000048680	-2.888638193	8.66E-07
ENSSSCG00000023806	-2.88960807	6.28E-12
ENSSSCG00000002341	-2.889890931	6.67E-20
ENSSSCG00000015766	-2.893366584	3.89E-12
ENSSSCG00000025243	-2.893543307	8.06E-09
ENSSSCG00000031011	-2.893648687	0.001398045
ENSSSCG00000047139	-2.894227336	0.006095036
ENSSSCG00000039633	-2.900521909	2.32E-12
ENSSSCG00000035025	-2.904613777	2.86E-07
ENSSSCG00000045950	-2.906554925	0.011485961
ENSSSCG00000031849	-2.907635558	0.014759923
ENSSSCG00000024668	-2.909080481	0.000221106
ENSSSCG00000023498	-2.910653448	8.88E-16
ENSSSCG00000033685	-2.916335995	3.57E-09
ENSSSCG00000028047	-2.920544952	2.07E-13
ENSSSCG00000023710	-2.925508364	3.70E-12
ENSSSCG00000015515	-2.926216722	0.018698559

ENSSSCG00000010647	-2.928425063	8.27E-08
ENSSSCG00000042191	-2.929026278	1.05E-06
ENSSSCG00000000664	-2.932226154	0.003873031
ENSSSCG00000048782	-2.93226549	0.009112304
ENSSSCG00000049333	-2.93449257	2.85E-05
ENSSSCG00000016685	-2.939189406	1.47E-23
ENSSSCG00000004052	-2.944596673	9.60E-06
ENSSSCG00000042541	-2.945268318	0.000207665
ENSSSCG00000051370	-2.945659474	0.007619723
ENSSSCG00000033245	-2.956196124	0.017407202
ENSSSCG00000044704	-2.956723579	0.000743852
ENSSSCG00000033469	-2.956757166	0.000289274
ENSSSCG00000035416	-2.957908544	8.30E-31
ENSSSCG00000035027	-2.959339351	7.80E-35
ENSSSCG00000045016	-2.960033808	1.75E-08
ENSSSCG00000050022	-2.965930429	0.012521128
ENSSSCG00000020895	-2.96922285	5.15E-06
ENSSSCG00000047293	-2.971716672	0.001264827
ENSSSCG00000033745	-2.971933754	0.000276334
ENSSSCG00000026210	-2.972610019	2.13E-43
ENSSSCG00000036589	-2.974885627	7.32E-15
ENSSSCG00000042737	-2.977836341	8.33E-14
ENSSSCG00000048513	-2.982902042	9.21E-09
ENSSSCG00000047946	-2.986255819	0.001601097
ENSSSCG00000012712	-2.986285934	5.23E-06
ENSSSCG00000004630	-2.988697199	6.02E-10
ENSSSCG00000034827	-2.995561769	5.15E-18
ENSSSCG00000023082	-2.996338294	3.31E-29
ENSSSCG00000047491	-2.99759901	0.018522403
ENSSSCG00000026564	-3.002614124	1.74E-05
ENSSSCG00000022247	-3.003424067	1.65E-05
ENSSSCG00000042402	-3.007677072	5.97E-05
ENSSSCG00000045740	-3.00886259	0.016844853
ENSSSCG00000047414	-3.008879079	5.78E-05
ENSSSCG00000035020	-3.010965985	0.003420979
ENSSSCG00000042069	-3.015064475	0.010698699
ENSSSCG00000007765	-3.016775735	0.000280288
ENSSSCG00000045763	-3.017169048	0.007038061
ENSSSCG00000007691	-3.023259771	0.000177879
ENSSSCG00000015662	-3.024485131	4.72E-26
ENSSSCG00000042232	-3.024524012	0.000279813
ENSSSCG00000032662	-3.027278149	3.15E-12
ENSSSCG00000033941	-3.034020225	0.00073172
ENSSSCG00000050854	-3.035686982	0.015106565
ENSSSCG00000041348	-3.03569649	0.001750793
ENSSSCG00000001045	-3.035905556	0.000519014
ENSSSCG00000045846	-3.037617506	0.001724487
ENSSSCG00000005593	-3.038503628	2.96E-20
ENSSSCG00000007501	-3.038890339	0.000162359
ENSSSCG00000008712	-3.042053506	1.24E-08
ENSSSCG00000041603	-3.048900389	1.13E-07
ENSSSCG00000049339	-3.054245577	0.001741157
ENSSSCG00000050870	-3.055169844	0.010469778
ENSSSCG00000013506	-3.05850434	2.13E-11
ENSSSCG00000016603	-3.061141228	0.012511591
ENSSSCG00000051250	-3.061446156	0.000383025
ENSSSCG00000024000	-3.062436868	4.02E-11
ENSSSCG00000015543	-3.065002019	9.48E-11

ENSSSCG00000012906	-3.065591322	0.000108194
ENSSSCG00000050490	-3.067607641	4.92E-06
ENSSSCG00000038147	-3.07410614	1.24E-06
ENSSSCG00000043154	-3.076430242	0.010548811
ENSSSCG00000031199	-3.076580246	1.33E-06
ENSSSCG00000041612	-3.085524152	3.13E-10
ENSSSCG00000029815	-3.09087199	9.21E-23
ENSSSCG00000023001	-3.092250318	2.73E-23
ENSSSCG00000042376	-3.093371811	0.017281143
ENSSSCG00000041735	-3.097576966	1.47E-07
ENSSSCG00000040110	-3.099510056	6.69E-17
ENSSSCG00000032433	-3.10119688	0.017027907
ENSSSCG00000015086	-3.1071965	1.24E-11
ENSSSCG00000001473	-3.109169513	1.52E-11
ENSSSCG00000051156	-3.110803618	0.004376008
ENSSSCG00000020988	-3.111090082	1.97E-11
ENSSSCG00000016574	-3.111979939	9.43E-40
ENSSSCG00000016472	-3.115362133	0.00010947
ENSSSCG00000015716	-3.116373118	1.51E-12
ENSSSCG00000005221	-3.120866297	1.74E-15
ENSSSCG00000049184	-3.120972465	0.000758366
ENSSSCG00000004632	-3.122652493	0.007057402
ENSSSCG00000046277	-3.125359622	0.000376531
ENSSSCG00000000789	-3.126153118	0.013464875
ENSSSCG00000016706	-3.134460854	0.005700275
ENSSSCG00000004890	-3.142987666	0.009303251
ENSSSCG00000044858	-3.143085491	0.00232781
ENSSSCG00000032852	-3.155795004	1.19E-16
ENSSSCG00000025176	-3.162202797	1.83E-13
ENSSSCG00000047343	-3.162671054	0.000367259
ENSSSCG00000047519	-3.163324381	0.006013709
ENSSSCG00000039261	-3.164497018	7.62E-14
ENSSSCG00000042842	-3.165523778	1.54E-09
ENSSSCG00000050791	-3.170888887	0.001994289
ENSSSCG00000012789	-3.171984701	8.19E-14
ENSSSCG00000050944	-3.174459957	0.006527468
ENSSSCG00000016664	-3.175924184	5.80E-05
ENSSSCG00000006935	-3.181437677	1.22E-05
ENSSSCG00000031959	-3.184579747	1.08E-08
ENSSSCG00000046611	-3.190751581	3.55E-05
ENSSSCG00000007009	-3.191269598	6.68E-26
ENSSSCG00000011888	-3.191564663	1.53E-05
ENSSSCG00000004849	-3.193162517	1.40E-05
ENSSSCG00000051462	-3.193342219	2.82E-08
ENSSSCG00000042799	-3.200309446	0.007815374
ENSSSCG00000029160	-3.203191301	1.30E-16
ENSSSCG00000043941	-3.208962504	0.006960358
ENSSSCG00000006328	-3.210972453	3.86E-10
ENSSSCG00000012818	-3.211859452	1.09E-20
ENSSSCG00000040632	-3.212332376	7.46E-05
ENSSSCG00000006462	-3.2134699	2.67E-07
ENSSSCG00000040752	-3.213861566	5.88E-21
ENSSSCG00000046546	-3.217860616	1.17E-15
ENSSSCG00000040147	-3.218064761	3.27E-13
ENSSSCG00000051130	-3.220920472	0.004411056
ENSSSCG00000028274	-3.221902786	3.29E-07
ENSSSCG00000039215	-3.222479764	0.000854608
ENSSSCG00000011280	-3.231105644	3.00E-05

ENSSSCG00000042663	-3.231378604	1.61E-14
ENSSSCG00000036893	-3.231591003	7.20E-06
ENSSSCG00000048886	-3.232741483	4.44E-10
ENSSSCG00000031640	-3.233826328	1.94E-36
ENSSSCG00000038162	-3.234898359	8.04E-34
ENSSSCG00000023451	-3.243633574	0.00237672
ENSSSCG00000015548	-3.247446747	0.000593788
ENSSSCG00000006456	-3.25349967	1.33E-24
ENSSSCG00000049280	-3.254291235	0.017415124
ENSSSCG00000002747	-3.257061604	0.016643455
ENSSSCG00000008664	-3.260137564	0.000641316
ENSSSCG00000030026	-3.263087815	6.76E-10
ENSSSCG00000035299	-3.267222259	1.20E-10
ENSSSCG00000003078	-3.272488731	0.013512993
ENSSSCG00000041050	-3.276426787	0.013981533
ENSSSCG00000042577	-3.276614003	0.006194631
ENSSSCG00000043063	-3.279380427	1.25E-09
ENSSSCG00000006458	-3.284930613	3.09E-32
ENSSSCG00000051592	-3.289939011	0.011725321
ENSSSCG00000008449	-3.290960331	0.001220517
ENSSSCG00000021812	-3.292988917	7.19E-43
ENSSSCG00000031716	-3.293476702	2.37E-16
ENSSSCG00000042267	-3.294752493	3.30E-11
ENSSSCG00000026689	-3.295846134	5.39E-16
ENSSSCG00000007727	-3.299724711	6.13E-26
ENSSSCG00000016639	-3.300859841	1.97E-05
ENSSSCG00000028338	-3.302012354	2.50E-34
ENSSSCG00000005609	-3.302404942	1.99E-32
ENSSSCG00000015747	-3.307306888	4.04E-12
ENSSSCG00000040850	-3.309458248	6.35E-05
ENSSSCG00000050566	-3.314896036	1.09E-17
ENSSSCG00000011186	-3.320747186	4.80E-14
ENSSSCG00000003366	-3.32412835	1.17E-23
ENSSSCG00000042175	-3.335406433	0.005180282
ENSSSCG00000023693	-3.345115187	1.01E-07
ENSSSCG00000050344	-3.346241868	5.19E-08
ENSSSCG00000030206	-3.34695286	0.000645415
ENSSSCG00000030345	-3.349391362	1.45E-17
ENSSSCG00000049957	-3.362690935	8.13E-07
ENSSSCG00000007498	-3.366012215	1.39E-10
ENSSSCG00000044347	-3.367401066	0.004237431
ENSSSCG00000051705	-3.374820313	0.002252855
ENSSSCG00000003733	-3.378850229	3.39E-18
ENSSSCG00000028341	-3.389445153	1.90E-05
ENSSSCG00000049586	-3.392400665	7.08E-07
ENSSSCG00000030821	-3.39424526	3.39E-23
ENSSSCG00000033544	-3.409004546	7.21E-15
ENSSSCG00000003081	-3.410541379	2.50E-22
ENSSSCG00000036052	-3.41104704	2.81E-12
ENSSSCG00000001403	-3.412916546	6.05E-29
ENSSSCG00000042713	-3.417394348	9.16E-17
ENSSSCG00000045570	-3.419316469	2.13E-12
ENSSSCG00000036029	-3.423026781	1.77E-19
ENSSSCG00000045628	-3.426441517	0.004301507
ENSSSCG00000022445	-3.428598662	0.000627964
ENSSSCG00000050972	-3.433873746	0.010092162
ENSSSCG00000013934	-3.434054973	1.44E-06
ENSSSCG00000042584	-3.4354261	0.002213306

ENSSSCG00000041571	-3.438945423	0.000937304
ENSSSCG00000003721	-3.445846804	0.000412871
ENSSSCG00000045237	-3.445902307	0.002810974
ENSSSCG00000037852	-3.445978244	6.41E-20
ENSSSCG00000050742	-3.446142993	8.19E-21
ENSSSCG00000014933	-3.448400355	4.84E-17
ENSSSCG00000001478	-3.458021487	0.008989093
ENSSSCG00000044412	-3.460850568	0.017388784
ENSSSCG00000028166	-3.462503751	7.96E-11
ENSSSCG00000041966	-3.466683117	6.97E-23
ENSSSCG00000009197	-3.467380175	0.003669633
ENSSSCG00000011579	-3.470705919	3.70E-38
ENSSSCG00000009738	-3.47422421	0.012096977
ENSSSCG00000049653	-3.474435426	0.006823976
ENSSSCG00000001672	-3.478789949	4.98E-28
ENSSSCG00000041037	-3.482746528	0.001071306
ENSSSCG00000031272	-3.488075514	0.00080901
ENSSSCG00000037188	-3.489509285	0.016904132
ENSSSCG00000050815	-3.493414222	4.74E-09
ENSSSCG00000051707	-3.499574718	1.37E-07
ENSSSCG00000044231	-3.499621832	0.000103324
ENSSSCG00000003722	-3.500749523	1.76E-28
ENSSSCG00000004803	-3.503983065	1.19E-11
ENSSSCG00000048349	-3.505111446	0.000667456
ENSSSCG00000024784	-3.505625501	2.58E-06
ENSSSCG00000022383	-3.510842763	0.005208955
ENSSSCG00000038043	-3.512892593	1.77E-35
ENSSSCG00000044190	-3.51457985	0.004850997
ENSSSCG00000047770	-3.524605055	2.81E-07
ENSSSCG00000002536	-3.526591302	6.34E-15
ENSSSCG00000047850	-3.527393892	0.002243414
ENSSSCG00000015906	-3.52859234	0.005249724
ENSSSCG00000049314	-3.531227719	0.002394383
ENSSSCG00000046131	-3.537164728	0.006368347
ENSSSCG00000007185	-3.540801197	1.24E-27
ENSSSCG00000033171	-3.545291712	6.43E-35
ENSSSCG00000005249	-3.566351442	9.52E-34
ENSSSCG00000046273	-3.566679855	8.54E-05
ENSSSCG00000005979	-3.569237403	0.000652168
ENSSSCG00000041070	-3.572497571	0.000770964
ENSSSCG00000045544	-3.573647582	0.004451688
ENSSSCG00000011522	-3.577349223	4.68E-08
ENSSSCG00000049117	-3.577362482	4.77E-25
ENSSSCG00000025924	-3.577500657	1.50E-28
ENSSSCG00000030565	-3.578952143	1.38E-13
ENSSSCG00000031858	-3.580857339	0.000360309
ENSSSCG00000032383	-3.585308453	1.48E-30
ENSSSCG00000049099	-3.590473041	0.00409854
ENSSSCG00000021651	-3.595334018	3.45E-05
ENSSSCG00000011766	-3.600920049	0.000460471
ENSSSCG00000006415	-3.604274962	4.27E-16
ENSSSCG00000036499	-3.606423959	1.19E-20
ENSSSCG00000044367	-3.606780023	0.014819464
ENSSSCG00000034335	-3.608832409	3.63E-15
ENSSSCG00000042555	-3.612204538	0.015301848
ENSSSCG00000005106	-3.621566593	1.52E-18
ENSSSCG00000042831	-3.63167495	0.003431171
ENSSSCG00000045867	-3.637244743	0.001394214

ENSSSCG00000048843	-3.641524924	0.003863033
ENSSSCG00000046860	-3.642401154	0.016348262
ENSSSCG00000051184	-3.644185154	0.002020768
ENSSSCG00000031906	-3.654387452	0.003306717
ENSSSCG00000046044	-3.657005516	0.000881062
ENSSSCG00000036475	-3.663529917	1.31E-13
ENSSSCG00000034592	-3.669136754	0.000812719
ENSSSCG00000044907	-3.675885904	0.000692114
ENSSSCG00000042169	-3.680459295	2.17E-14
ENSSSCG00000032167	-3.690400896	0.002385896
ENSSSCG00000038508	-3.691090321	9.49E-32
ENSSSCG00000023852	-3.693917218	3.97E-06
ENSSSCG00000044838	-3.700838153	0.001287609
ENSSSCG00000041957	-3.720560173	0.010885661
ENSSSCG00000032702	-3.724707571	2.02E-07
ENSSSCG00000025681	-3.730851294	5.28E-05
ENSSSCG00000042292	-3.742253953	0.003247986
ENSSSCG00000008584	-3.743086995	0.017778757
ENSSSCG00000001698	-3.752700281	6.55E-06
ENSSSCG00000011872	-3.765348465	2.17E-05
ENSSSCG00000016285	-3.783952234	0.010165823
ENSSSCG00000046901	-3.790600812	0.000565454
ENSSSCG00000044695	-3.798504025	1.62E-06
ENSSSCG00000029656	-3.802868696	0.008940701
ENSSSCG00000034987	-3.814567079	0.009100996
ENSSSCG00000046010	-3.819037645	0.00128136
ENSSSCG00000004931	-3.833292302	2.52E-35
ENSSSCG00000002129	-3.835584958	3.14E-13
ENSSSCG00000029043	-3.843137884	5.49E-08
ENSSSCG00000005456	-3.852011519	2.69E-12
ENSSSCG00000048861	-3.856427599	3.62E-06
ENSSSCG00000004614	-3.859767832	2.38E-05
ENSSSCG00000013261	-3.863782074	8.81E-22
ENSSSCG00000014725	-3.864122003	1.84E-18
ENSSSCG00000037260	-3.866355026	0.002717065
ENSSSCG00000027024	-3.873120111	6.51E-10
ENSSSCG00000026113	-3.873443082	7.22E-06
ENSSSCG00000048269	-3.875100597	0.01085737
ENSSSCG00000024476	-3.87570685	1.01E-46
ENSSSCG00000011622	-3.880444218	1.66E-05
ENSSSCG00000046889	-3.885397555	0.004539451
ENSSSCG00000045169	-3.885583817	0.007379916
ENSSSCG00000006874	-3.887294487	5.02E-28
ENSSSCG00000051027	-3.889193447	0.004887574
ENSSSCG00000047531	-3.889364904	5.24E-06
ENSSSCG00000049453	-3.897182362	0.001132276
ENSSSCG00000049475	-3.898110159	0.014052697
ENSSSCG00000007926	-3.901256995	2.51E-17
ENSSSCG00000034689	-3.919175095	1.12E-36
ENSSSCG00000024814	-3.935502342	0.011525128
ENSSSCG00000043741	-3.938168738	5.10E-05
ENSSSCG00000010222	-3.944501763	3.29E-06
ENSSSCG00000045209	-3.957935184	7.11E-06
ENSSSCG00000035536	-3.966955132	0.000974652
ENSSSCG00000017282	-3.975748826	6.39E-17
ENSSSCG00000045905	-3.976696837	0.000220235
ENSSSCG00000034993	-3.985535229	3.60E-50
ENSSSCG00000022999	-3.986898347	6.54E-10

ENSSSCG00000016093	-3.991978101	0.001209304
ENSSSCG00000002817	-3.993006063	7.82E-26
ENSSSCG00000000585	-3.99659652	0.004770603
ENSSSCG00000047564	-4.001532158	0.001314786
ENSSSCG00000037567	-4.002581815	1.58E-27
ENSSSCG00000040377	-4.00638767	1.45E-31
ENSSSCG00000007909	-4.010510389	5.24E-29
ENSSSCG00000000867	-4.01839135	5.75E-43
ENSSSCG00000007977	-4.025126703	5.86E-24
ENSSSCG00000045084	-4.026441808	1.64E-73
ENSSSCG00000051630	-4.039009101	0.018056186
ENSSSCG00000033910	-4.057431126	2.25E-08
ENSSSCG00000012530	-4.064641496	1.34E-09
ENSSSCG00000042534	-4.065383124	0.00396789
ENSSSCG00000037025	-4.06789097	5.61E-132
ENSSSCG00000044497	-4.075512139	1.61E-05
ENSSSCG00000011208	-4.078002914	0.004095059
ENSSSCG00000003417	-4.096679281	0.005678866
ENSSSCG00000042953	-4.099665367	0.010227611
ENSSSCG00000051365	-4.105922738	7.37E-06
ENSSSCG00000013358	-4.115661832	1.25E-10
ENSSSCG00000004270	-4.116329593	1.50E-06
ENSSSCG00000031616	-4.116683737	7.31E-37
ENSSSCG00000004678	-4.126751047	1.64E-28
ENSSSCG00000000433	-4.131810063	4.36E-58
ENSSSCG00000043135	-4.134303552	0.006431981
ENSSSCG00000051508	-4.146219853	0.013668223
ENSSSCG00000037106	-4.146440552	4.45E-43
ENSSSCG00000047456	-4.149619139	2.53E-16
ENSSSCG00000005504	-4.155231111	0.017592272
ENSSSCG00000037179	-4.158849474	3.54E-39
ENSSSCG00000039522	-4.16428164	1.21E-31
ENSSSCG00000049533	-4.166351343	0.003671373
ENSSSCG00000036017	-4.16714806	5.52E-08
ENSSSCG00000046565	-4.167347608	0.003945541
ENSSSCG00000040826	-4.167768909	3.81E-23
ENSSSCG00000024417	-4.179260469	0.000297441
ENSSSCG00000003146	-4.183640128	3.32E-21
ENSSSCG00000046184	-4.18430407	0.017267452
ENSSSCG00000010142	-4.186902282	6.00E-14
ENSSSCG00000041313	-4.189344503	0.010839291
ENSSSCG00000048320	-4.209610777	0.00225621
ENSSSCG00000016883	-4.218343973	0.006882258
ENSSSCG00000043451	-4.220178661	0.00362025
ENSSSCG00000037430	-4.230125603	1.36E-52
ENSSSCG00000009564	-4.236190819	2.47E-29
ENSSSCG00000047011	-4.241833181	0.000192282
ENSSSCG00000032303	-4.24736067	4.58E-09
ENSSSCG00000044611	-4.249431598	0.018659388
ENSSSCG00000003604	-4.24963584	2.16E-20
ENSSSCG00000044428	-4.266031485	1.81E-05
ENSSSCG00000011380	-4.267269907	0.016634717
ENSSSCG00000002718	-4.269240459	0.003062002
ENSSSCG00000049026	-4.281531969	0.014520768
ENSSSCG00000029240	-4.2948821	0.012774572
ENSSSCG00000049652	-4.2948821	0.012774572
ENSSSCG00000032896	-4.298438475	1.20E-11
ENSSSCG00000004285	-4.315931542	0.000122957

ENSSSCG00000010745	-4.32104827	3.93E-07
ENSSSCG00000047432	-4.336623464	2.32E-14
ENSSSCG00000025273	-4.336897197	1.85E-09
ENSSSCG00000033486	-4.337923772	0.001836951
ENSSSCG00000000144	-4.340823072	4.88E-16
ENSSSCG00000045162	-4.346728835	0.010922659
ENSSSCG00000026237	-4.34952499	0.001547376
ENSSSCG00000038647	-4.353612223	0.01223323
ENSSSCG00000015619	-4.360045338	6.13E-22
ENSSSCG00000023014	-4.379665122	1.84E-19
ENSSSCG00000043870	-4.382565827	3.70E-07
ENSSSCG00000048776	-4.401941006	2.18E-05
ENSSSCG00000010359	-4.402640705	3.21E-11
ENSSSCG00000011277	-4.412085535	3.44E-06
ENSSSCG00000048719	-4.416680531	0.000604209
ENSSSCG00000022105	-4.43134212	4.22E-12
ENSSSCG00000041983	-4.45741258	0.001790762
ENSSSCG00000047560	-4.479926132	0.008114119
ENSSSCG00000042371	-4.492197692	4.31E-12
ENSSSCG00000048848	-4.492778552	0.00721935
ENSSSCG00000023891	-4.501278454	1.25E-19
ENSSSCG00000010384	-4.51103098	0.000211314
ENSSSCG00000045077	-4.51660294	0.007986026
ENSSSCG00000043222	-4.517625344	0.00379105
ENSSSCG00000046830	-4.52816992	0.009159369
ENSSSCG00000034940	-4.528200973	0.010468106
ENSSSCG00000034995	-4.529056407	0.015246686
ENSSSCG00000031740	-4.529056407	0.015246686
ENSSSCG00000043391	-4.549618578	0.004159295
ENSSSCG00000047636	-4.55353684	0.000783191
ENSSSCG00000014431	-4.557060515	1.15E-62
ENSSSCG00000012638	-4.559798671	2.91E-12
ENSSSCG00000051011	-4.578026062	0.006344141
ENSSSCG00000008771	-4.598974425	8.29E-24
ENSSSCG00000041096	-4.616720708	0.01778436
ENSSSCG00000012571	-4.627925458	3.69E-16
ENSSSCG00000000028	-4.651275667	0.004071159
ENSSSCG00000051226	-4.652823093	0.005109394
ENSSSCG00000015294	-4.686904188	1.69E-59
ENSSSCG00000041048	-4.69821379	3.43E-08
ENSSSCG00000043554	-4.699241733	8.52E-07
ENSSSCG00000044542	-4.729401559	3.76E-05
ENSSSCG00000049517	-4.732397189	0.016993723
ENSSSCG00000000516	-4.733269378	0.01864823
ENSSSCG00000047502	-4.737264193	0.006776757
ENSSSCG00000051736	-4.753217541	0.018619792
ENSSSCG00000025993	-4.77691472	7.94E-07
ENSSSCG00000050526	-4.785588258	0.000183659
ENSSSCG00000022569	-4.802627677	3.07E-07
ENSSSCG00000032971	-4.816447366	3.88E-06
ENSSSCG00000001393	-4.816867183	0.015910627
ENSSSCG00000003498	-4.816867183	0.015910627
ENSSSCG00000025294	-4.832585629	0.01547205
ENSSSCG00000027768	-4.83784144	6.15E-20
ENSSSCG00000039961	-4.850700968	6.79E-09
ENSSSCG00000036270	-4.853643397	2.00E-08
ENSSSCG00000044832	-4.860228927	0.000254793
ENSSSCG00000013788	-4.861293647	2.07E-69

ENSSSCG00000050466	-4.864654846	0.003425171
ENSSSCG00000050170	-4.872802332	1.38E-15
ENSSSCG00000039883	-4.881558604	5.75E-89
ENSSSCG00000001832	-4.88418117	0.002470629
ENSSSCG00000043758	-4.888033232	0.014030064
ENSSSCG00000043816	-4.888931537	0.002346167
ENSSSCG00000030227	-4.89389492	0.000109592
ENSSSCG00000017220	-4.894146991	0.014013882
ENSSSCG00000048606	-4.911526852	0.011763635
ENSSSCG00000012551	-4.917710655	9.74E-05
ENSSSCG00000011204	-4.92917375	0.010667504
ENSSSCG00000043286	-4.937349166	0.003013288
ENSSSCG00000049330	-4.944441224	0.011286733
ENSSSCG00000045022	-4.944441224	0.011286733
ENSSSCG00000049462	-4.955216815	1.05E-21
ENSSSCG00000043317	-4.959621134	1.03E-39
ENSSSCG00000022233	-4.968704942	0.015896242
ENSSSCG00000051026	-4.987394626	0.015815875
ENSSSCG00000003126	-4.995562294	0.00136043
ENSSSCG00000048079	-5.027440367	0.008550889
ENSSSCG00000011581	-5.033982098	0.00766218
ENSSSCG00000011060	-5.033982098	0.00766218
ENSSSCG00000015355	-5.034675569	0.0080427
ENSSSCG00000015821	-5.057112241	3.28E-35
ENSSSCG00000035965	-5.064037193	0.009559104
ENSSSCG00000040652	-5.070332211	0.008799594
ENSSSCG00000001979	-5.079306207	9.56E-23
ENSSSCG00000033405	-5.080145875	0.012367389
ENSSSCG00000050322	-5.084187136	0.012280648
ENSSSCG00000046024	-5.104449207	0.012885959
ENSSSCG00000031373	-5.131706251	0.005648974
ENSSSCG00000048047	-5.132384556	0.00595683
ENSSSCG00000032923	-5.140480838	6.60E-19
ENSSSCG00000048660	-5.142334513	0.001433215
ENSSSCG00000050902	-5.14653728	0.004241708
ENSSSCG00000004433	-5.166017471	0.005649146
ENSSSCG00000048906	-5.177803624	0.007412174
ENSSSCG00000034245	-5.184829359	0.000716366
ENSSSCG00000044707	-5.206919942	0.00796902
ENSSSCG00000046111	-5.251681685	0.012575539
ENSSSCG00000017003	-5.256021267	5.06E-47
ENSSSCG00000031795	-5.267070244	0.000584076
ENSSSCG00000003567	-5.268358452	0.0035194
ENSSSCG00000031213	-5.279754236	0.005491641
ENSSSCG00000046485	-5.291927144	0.000463876
ENSSSCG00000047537	-5.293522724	0.007298768
ENSSSCG00000008314	-5.304466885	3.55E-12
ENSSSCG00000050569	-5.307027949	8.67E-05
ENSSSCG00000047191	-5.307809325	0.007176407
ENSSSCG00000043076	-5.319214449	0.001866527
ENSSSCG00000048970	-5.325952739	0.003060251
ENSSSCG00000042431	-5.337500776	0.003399651
ENSSSCG00000043723	-5.337876316	0.004740508
ENSSSCG00000041780	-5.345451401	0.006578405
ENSSSCG00000048203	-5.350930844	0.004828109
ENSSSCG00000048864	-5.351829593	0.013115289
ENSSSCG00000041839	-5.358286666	0.000360684
ENSSSCG00000045640	-5.362961283	0.001938572

ENSSSCG00000036626	-5.378507189	3.61E-10
ENSSSCG00000017585	-5.392154822	0.003116437
ENSSSCG00000002830	-5.403785094	0.002796873
ENSSSCG00000023746	-5.415562706	0.0056278
ENSSSCG00000050296	-5.41760146	0.000222633
ENSSSCG00000049352	-5.418861289	0.001961316
ENSSSCG00000045565	-5.439157325	4.44E-05
ENSSSCG00000049374	-5.443623468	0.00188226
ENSSSCG00000038570	-5.457534653	0.002172096
ENSSSCG00000041446	-5.467294302	0.002037288
ENSSSCG00000040928	-5.467996625	0.002833898
ENSSSCG00000011489	-5.493327641	0.001666587
ENSSSCG00000046359	-5.503660995	0.003294121
ENSSSCG00000047852	-5.504768928	0.001794208
ENSSSCG00000012732	-5.507732424	0.001785326
ENSSSCG00000048424	-5.52166219	0.000149854
ENSSSCG00000043573	-5.565975825	0.00262965
ENSSSCG00000050748	-5.579105819	0.008439735
ENSSSCG00000023859	-5.579678121	0.001312302
ENSSSCG00000039272	-5.581558407	0.000119669
ENSSSCG00000011441	-5.592212693	0.003265158
ENSSSCG00000021003	-5.593697571	0.018369878
ENSSSCG00000031031	-5.596229756	0.000258825
ENSSSCG00000048207	-5.599873556	0.00126511
ENSSSCG00000045208	-5.600951269	0.003272638
ENSSSCG00000051129	-5.624208194	0.000573817
ENSSSCG00000036134	-5.655518055	1.40E-05
ENSSSCG00000023169	-5.689002746	0.001430993
ENSSSCG00000016442	-5.70344382	5.15E-105
ENSSSCG00000042892	-5.722264575	0.001104397
ENSSSCG00000033906	-5.72914608	5.48E-16
ENSSSCG00000046734	-5.737844717	0.001601024
ENSSSCG00000011238	-5.740167829	0.001481876
ENSSSCG00000042283	-5.753871269	0.000648366
ENSSSCG00000012254	-5.761598973	9.78E-117
ENSSSCG00000049959	-5.774514755	0.002374918
ENSSSCG00000041295	-5.815332837	0.000785663
ENSSSCG00000048923	-5.861665627	0.001228531
ENSSSCG00000029370	-5.872972449	0.000297054
ENSSSCG00000047445	-5.873213773	0.001659756
ENSSSCG00000049033	-5.88998193	0.000303898
ENSSSCG00000048004	-5.897059315	0.000153182
ENSSSCG00000042486	-5.903070895	0.0003278
ENSSSCG00000033514	-5.916645357	0.000309463
ENSSSCG00000044965	-5.935386267	0.000743109
ENSSSCG00000035226	-5.946223505	0.000206769
ENSSSCG00000033425	-5.953023344	2.54E-05
ENSSSCG00000043191	-5.954184698	0.00165366
ENSSSCG00000044485	-5.970404641	0.000294467
ENSSSCG00000032058	-5.998051714	1.54E-05
ENSSSCG00000011646	-6.004463779	2.95E-07
ENSSSCG00000006013	-6.036987498	3.27E-20
ENSSSCG00000043112	-6.042733193	1.29E-05
ENSSSCG00000049296	-6.073835946	2.06E-10
ENSSSCG00000050123	-6.07448917	0.000128412
ENSSSCG00000050077	-6.116099805	8.75E-05
ENSSSCG00000043743	-6.165814635	9.45E-05
ENSSSCG00000005519	-6.193444562	7.23E-05

ENSSSCG00000021527	-6.239016057	8.71E-06
ENSSSCG00000043581	-6.267463315	5.16E-05
ENSSSCG00000024697	-6.295129584	2.31E-11
ENSSSCG00000044924	-6.341163063	0.000358026
ENSSSCG00000050133	-6.4395945	9.32E-05
ENSSSCG00000032720	-6.448979297	6.15E-05
ENSSSCG00000041558	-6.45872218	0.000365594
ENSSSCG00000035210	-6.470586174	1.51E-05
ENSSSCG00000051282	-6.496126211	0.000178229
ENSSSCG00000046046	-6.581719885	0.000468012
ENSSSCG00000040795	-6.592303242	9.33E-07
ENSSSCG00000047735	-6.59967011	1.11E-05
ENSSSCG00000050073	-6.641402813	4.97E-06
ENSSSCG00000003123	-6.724019551	3.96E-06
ENSSSCG00000047356	-6.738000592	7.40E-13
ENSSSCG00000031998	-6.73808182	4.10E-07
ENSSSCG00000041916	-6.759816629	3.06E-06
ENSSSCG00000040088	-6.798711344	2.91E-06
ENSSSCG00000046678	-6.809794267	1.88E-06
ENSSSCG00000012075	-6.870646735	1.97E-06
ENSSSCG00000035102	-6.928793638	1.58E-06
ENSSSCG00000005352	-6.934456226	2.03E-58
ENSSSCG00000007262	-7.091516669	3.89E-07
ENSSSCG00000047131	-7.169163346	2.77E-07
ENSSSCG00000049649	-7.193219079	3.09E-08
ENSSSCG00000046005	-7.195428505	2.01E-07
ENSSSCG00000015969	-7.213399848	2.52E-07
ENSSSCG00000032659	-7.21812363	2.32E-08
ENSSSCG00000045536	-7.289691568	1.14E-05
ENSSSCG00000039007	-7.380026981	1.24E-08
ENSSSCG00000031976	-7.481187823	1.48E-30
ENSSSCG00000050487	-7.497043185	3.25E-07
ENSSSCG00000046275	-7.568778393	2.22E-20
ENSSSCG00000002314	-7.611826709	1.31E-115
ENSSSCG00000036190	-7.628378332	1.02E-11
ENSSSCG00000050669	-7.817459812	2.80E-09
ENSSSCG00000049084	-7.858340787	1.60E-18
ENSSSCG00000034799	-7.90370105	1.10E-07
ENSSSCG00000043449	-8.090576113	8.03E-10
ENSSSCG00000039813	-8.211239842	1.46E-09
ENSSSCG00000051349	-8.241090652	6.58E-10
ENSSSCG00000023849	-8.544709481	6.21E-11
ENSSSCG00000007695	-8.578952007	3.02E-11
ENSSSCG00000043322	-8.609166812	1.93E-11
ENSSSCG00000043920	-8.850252664	2.32E-12
ENSSSCG00000031311	-9.004720268	3.03E-11
ENSSSCG00000039791	-9.303785425	1.06E-13
ENSSSCG00000009144	-9.387070003	1.24E-14
ENSSSCG00000046022	-9.623531018	1.04E-14
ENSSSCG00000049232	-10.62041854	9.49E-18
ENSSSCG00000022701	-11.27280576	1.20E-20
ENSSSCG00000033930	-11.58650323	7.81E-22
ENSSSCG00000023258	-11.73509716	1.44E-22
ENSSSCG00000028372	-21.33452235	4.85E-08
ENSSSCG00000003062	-22.09959518	1.57E-08

Supplementary Table 4. The differentially expressed genes between the infected and mock group from inguinal lymph node samples

GeneID	logFC	Pvalue
ENSSSCG00000000492	24.13464256	6.53E-10
ENSSSCG00000030140	9.31213034	3.71E-63
ENSSSCG00000000207	7.646549485	0.000383354
ENSSSCG00000016400	7.633270626	6.75E-09
ENSSSCG00000032662	7.51005036	5.37E-08
ENSSSCG00000008088	7.333469696	2.53E-06
ENSSSCG00000047413	7.14705537	1.62E-08
ENSSSCG00000049577	6.63534369	7.93E-13
ENSSSCG00000003006	6.615552426	1.81E-47
ENSSSCG00000032557	6.609208588	1.05E-80
ENSSSCG00000006545	6.577741504	3.57E-06
ENSSSCG00000009921	6.546307744	1.94E-26
ENSSSCG00000000482	6.402115883	0.000814031
ENSSSCG00000000074	6.383144393	6.97E-05
ENSSSCG00000049097	6.203720536	9.02E-05
ENSSSCG00000024823	6.145236026	0.006733129
ENSSSCG00000016517	6.118713257	3.86E-08
ENSSSCG00000016902	6.096562059	1.38E-07
ENSSSCG00000023305	6.070227202	1.87E-34
ENSSSCG00000049851	5.980188696	1.29E-10
ENSSSCG00000026339	5.89868847	0.000289655
ENSSSCG00000034866	5.877161234	1.28E-24
ENSSSCG00000043809	5.711782416	5.01E-05
ENSSSCG00000039651	5.71083452	8.38E-07
ENSSSCG00000017470	5.69370919	8.66E-07
ENSSSCG00000036157	5.586194468	0.000513902
ENSSSCG00000039158	5.583726791	4.02E-08
ENSSSCG00000000415	5.572701015	0.000552759
ENSSSCG00000028062	5.549530596	7.89E-08
ENSSSCG00000046992	5.500870265	0.00140129
ENSSSCG00000021971	5.489908172	1.29E-10
ENSSSCG00000046697	5.433610732	0.00095723
ENSSSCG00000033387	5.372869201	0.000377001
ENSSSCG00000038521	5.325479338	1.39E-42
ENSSSCG00000004355	5.310386568	0.001946459
ENSSSCG00000051296	5.279907601	0.004609513
ENSSSCG00000044986	5.203560272	1.53E-05
ENSSSCG00000035431	5.160535539	2.08E-07
ENSSSCG00000049582	5.156685945	0.003274558
ENSSSCG00000004180	5.149850078	0.000177317
ENSSSCG00000038965	5.118891985	0.000891562
ENSSSCG00000032643	5.019998178	4.86E-07
ENSSSCG00000028104	4.99673208	0.005574404
ENSSSCG00000008314	4.993158289	0.000700771
ENSSSCG00000032653	4.961379578	0.002067852
ENSSSCG00000016609	4.925887614	2.92E-37
ENSSSCG00000008938	4.912216022	0.000128629
ENSSSCG00000016402	4.797528395	0.000144898
ENSSSCG00000001576	4.757122878	0.002794927
ENSSSCG00000008957	4.757039234	0.000158495
ENSSSCG00000041892	4.744748478	0.000445318
ENSSSCG00000045758	4.654262023	1.33E-08
ENSSSCG00000045298	4.620662358	0.000565609
ENSSSCG00000044133	4.60978045	3.10E-16
ENSSSCG00000046783	4.584520978	2.95E-07
ENSSSCG00000027130	4.564133499	6.71E-26
ENSSSCG00000039834	4.514117599	0.007676466

ENSSSCG00000044109	4.491831721	1.38E-06
ENSSSCG00000026043	4.479832551	9.32E-14
ENSSSCG00000010595	4.475418971	2.88E-11
ENSSSCG00000001748	4.442104046	0.002730213
ENSSSCG00000036669	4.439793064	0.001059792
ENSSSCG00000016903	4.425219791	7.40E-15
ENSSSCG00000024867	4.414005298	9.19E-80
ENSSSCG00000051050	4.379416176	1.67E-06
ENSSSCG00000037567	4.370101683	4.56E-06
ENSSSCG00000039862	4.355216362	9.38E-16
ENSSSCG00000048028	4.340053816	0.008701111
ENSSSCG00000014600	4.337530545	1.88E-32
ENSSSCG00000033578	4.32186546	0.001887941
ENSSSCG00000034961	4.266914291	7.16E-08
ENSSSCG00000033114	4.093927422	4.37E-47
ENSSSCG00000002990	4.093081896	0.000275044
ENSSSCG00000000194	4.074516631	2.54E-05
ENSSSCG00000051298	4.003161588	4.26E-05
ENSSSCG00000030388	3.998874013	3.98E-29
ENSSSCG00000040575	3.995675929	5.11E-66
ENSSSCG00000035928	3.974906966	6.62E-08
ENSSSCG00000033808	3.96866996	0.000454025
ENSSSCG00000038221	3.902109857	0.004349764
ENSSSCG00000037358	3.896228189	0.000548574
ENSSSCG00000017978	3.886519497	0.000151416
ENSSSCG00000041701	3.87369349	3.51E-09
ENSSSCG00000049325	3.862441752	0.002093238
ENSSSCG00000041810	3.860831366	6.35E-07
ENSSSCG00000010614	3.852639575	4.45E-06
ENSSSCG00000032857	3.850069911	7.81E-30
ENSSSCG00000043855	3.849487471	8.25E-06
ENSSSCG00000001978	3.823637884	1.21E-14
ENSSSCG00000003852	3.819968089	0.000275008
ENSSSCG00000020970	3.805419118	0.000647757
ENSSSCG00000013425	3.794884273	0.010427967
ENSSSCG00000000413	3.784564638	3.48E-09
ENSSSCG00000001395	3.76796472	1.56E-05
ENSSSCG00000002847	3.748546117	2.72E-13
ENSSSCG00000038966	3.719745051	2.07E-27
ENSSSCG00000038719	3.634054207	1.81E-19
ENSSSCG00000007596	3.62019751	2.18E-19
ENSSSCG00000043755	3.599818116	0.001755969
ENSSSCG00000040815	3.598440086	1.23E-56
ENSSSCG00000030300	3.579442122	5.86E-11
ENSSSCG00000041588	3.561059253	8.36E-06
ENSSSCG00000039492	3.55591364	7.06E-14
ENSSSCG00000008261	3.555803014	6.70E-05
ENSSSCG00000032777	3.554655096	5.17E-32
ENSSSCG00000049062	3.548695707	0.006041092
ENSSSCG00000031751	3.536444934	3.91E-05
ENSSSCG00000007859	3.532512148	0.004575325
ENSSSCG00000012967	3.512061701	3.91E-08
ENSSSCG00000047061	3.509236712	0.003598936
ENSSSCG00000022417	3.506336693	3.60E-05
ENSSSCG00000033207	3.494927866	2.11E-06
ENSSSCG00000046958	3.487977699	4.58E-08
ENSSSCG00000000080	3.48295723	0.001657988
ENSSSCG00000047108	3.478199355	3.85E-05

ENSSSCG00000032343	3.463618732	1.49E-20
ENSSSCG00000008978	3.45719345	1.95E-16
ENSSSCG00000032221	3.454552327	1.26E-13
ENSSSCG00000023041	3.451624452	0.001781659
ENSSSCG00000038351	3.439031109	4.02E-13
ENSSSCG00000033183	3.438550539	2.62E-11
ENSSSCG00000039252	3.42707085	0.001385542
ENSSSCG00000039798	3.40227141	9.15E-08
ENSSSCG00000007748	3.400362341	1.58E-12
ENSSSCG00000030371	3.378258564	3.23E-09
ENSSSCG00000040162	3.348487434	4.03E-35
ENSSSCG00000005287	3.345182491	5.53E-13
ENSSSCG00000030830	3.341318205	1.10E-10
ENSSSCG00000006013	3.341042043	0.000655863
ENSSSCG00000006590	3.336878128	7.51E-08
ENSSSCG00000017192	3.327402814	4.27E-09
ENSSSCG00000050493	3.321136257	0.000528905
ENSSSCG00000001749	3.308447634	0.000531518
ENSSSCG00000008953	3.307934206	5.91E-07
ENSSSCG00000007007	3.287402436	6.85E-27
ENSSSCG00000006796	3.283702915	2.20E-25
ENSSSCG00000039615	3.277638664	9.13E-08
ENSSSCG00000032769	3.277638664	9.13E-08
ENSSSCG00000041378	3.275759617	0.000789612
ENSSSCG00000006588	3.266045723	3.39E-10
ENSSSCG00000048707	3.254797534	0.004768172
ENSSSCG00000027710	3.253609023	0.000179253
ENSSSCG00000032578	3.234396707	0.000247268
ENSSSCG00000048627	3.232796699	0.000868709
ENSSSCG00000031374	3.220036217	1.21E-09
ENSSSCG00000035379	3.20403852	0.00107075
ENSSSCG00000003669	3.197187841	0.000129633
ENSSSCG00000015340	3.188358809	4.36E-14
ENSSSCG00000015086	3.186293734	2.22E-11
ENSSSCG00000004572	3.177173842	0.006202491
ENSSSCG00000006359	3.159904129	3.97E-19
ENSSSCG00000034119	3.153218083	5.86E-05
ENSSSCG00000040986	3.124560473	1.14E-10
ENSSSCG00000023280	3.11738266	1.79E-08
ENSSSCG00000034723	3.112127784	2.26E-13
ENSSSCG00000035443	3.108418463	0.008782366
ENSSSCG00000013854	3.097256631	0.011772827
ENSSSCG00000022258	3.091426928	8.41E-05
ENSSSCG00000017331	3.08063582	0.000350335
ENSSSCG00000038946	3.057144384	0.000105634
ENSSSCG00000001137	3.054928606	1.91E-10
ENSSSCG00000005486	3.051419874	1.01E-14
ENSSSCG00000006238	3.041254144	0.000102203
ENSSSCG00000037775	3.027588372	1.43E-10
ENSSSCG00000043578	3.023172749	0.000542261
ENSSSCG00000004492	3.004481707	4.91E-40
ENSSSCG00000004195	2.994553008	0.013194103
ENSSSCG00000006966	2.993237569	2.41E-05
ENSSSCG00000040134	2.989602126	4.24E-44
ENSSSCG00000042841	2.988549346	3.43E-11
ENSSSCG00000014168	2.972181955	6.67E-21
ENSSSCG00000017187	2.966699581	0.00105373
ENSSSCG00000042778	2.960962218	0.007353447

ENSSSCG00000042602	2.956940845	6.92E-07
ENSSSCG00000008959	2.954251429	0.001155013
ENSSSCG00000048726	2.950845026	2.48E-05
ENSSSCG00000046471	2.933311591	0.011508555
ENSSSCG00000038916	2.9306091	6.84E-06
ENSSSCG00000031037	2.920716948	5.62E-56
ENSSSCG00000032337	2.916241204	6.35E-10
ENSSSCG00000035935	2.905983445	0.002364188
ENSSSCG00000030582	2.903668441	1.29E-07
ENSSSCG00000045769	2.895850671	0.003506818
ENSSSCG00000042374	2.891559434	0.005874242
ENSSSCG00000026932	2.888252803	0.005007933
ENSSSCG00000010508	2.885686478	9.54E-43
ENSSSCG00000049441	2.879392982	5.61E-05
ENSSSCG00000007008	2.878296484	0.006579593
ENSSSCG00000039672	2.864023143	4.17E-09
ENSSSCG00000004702	2.858445391	5.07E-08
ENSSSCG00000039758	2.840199381	9.22E-05
ENSSSCG00000043763	2.840108847	2.82E-28
ENSSSCG00000009182	2.81696984	0.000208699
ENSSSCG00000041627	2.809169611	8.87E-05
ENSSSCG00000025266	2.803242292	0.000310344
ENSSSCG00000038489	2.791365147	4.46E-10
ENSSSCG00000000002	2.771108549	0.005930366
ENSSSCG00000039553	2.764813079	0.004604386
ENSSSCG00000013575	2.756259811	0.001025362
ENSSSCG00000023531	2.728220081	1.19E-06
ENSSSCG00000012077	2.726818243	3.48E-38
ENSSSCG00000030575	2.718087661	0.0002907
ENSSSCG00000032016	2.717632759	1.15E-08
ENSSSCG00000032860	2.71058895	6.14E-07
ENSSSCG00000046753	2.710149974	0.000238674
ENSSSCG00000025981	2.70846131	1.06E-10
ENSSSCG00000049483	2.707770907	0.004271526
ENSSSCG00000042919	2.692309026	2.87E-11
ENSSSCG00000040931	2.683812374	6.17E-40
ENSSSCG00000004369	2.678815274	5.41E-25
ENSSSCG00000042062	2.668168002	1.22E-06
ENSSSCG00000024179	2.663525099	0.008386812
ENSSSCG00000022405	2.658271871	8.70E-06
ENSSSCG00000024914	2.657492075	1.31E-08
ENSSSCG00000037913	2.65519014	2.78E-08
ENSSSCG00000039419	2.653395061	2.90E-09
ENSSSCG00000042034	2.652055872	0.003170512
ENSSSCG00000002400	2.647868652	0.003944101
ENSSSCG00000015082	2.64034828	0.000167681
ENSSSCG00000000981	2.639599003	1.07E-17
ENSSSCG00000011673	2.635915722	7.24E-18
ENSSSCG00000011391	2.6349187	4.60E-18
ENSSSCG00000029239	2.626545508	1.02E-14
ENSSSCG00000032474	2.619818252	4.98E-24
ENSSSCG00000031595	2.610034688	1.37E-09
ENSSSCG00000016983	2.609533147	2.90E-10
ENSSSCG00000033451	2.597129693	5.85E-17
ENSSSCG00000048050	2.591128578	0.001015051
ENSSSCG00000023684	2.543948057	0.00546326
ENSSSCG00000010084	2.527772899	2.74E-14
ENSSSCG00000002009	2.525670889	1.52E-19

ENSSSCG00000051319	2.50915683	0.005585251
ENSSSCG00000008284	2.505099659	0.003829371
ENSSSCG00000022630	2.50276112	7.35E-16
ENSSSCG00000027621	2.492400165	1.47E-19
ENSSSCG00000006464	2.491116565	0.000357508
ENSSSCG00000010451	2.490714663	8.53E-13
ENSSSCG00000043360	2.483558643	1.39E-05
ENSSSCG00000010568	2.461979491	1.02E-05
ENSSSCG00000040651	2.457880869	3.17E-08
ENSSSCG00000039673	2.442104637	4.27E-08
ENSSSCG00000032914	2.440408648	4.46E-31
ENSSSCG00000009136	2.418594053	0.005735042
ENSSSCG00000038215	2.399345219	2.91E-06
ENSSSCG00000011195	2.396912185	1.08E-09
ENSSSCG00000032996	2.39367011	1.41E-09
ENSSSCG00000003201	2.385374287	3.27E-05
ENSSSCG00000032365	2.382984817	0.011547081
ENSSSCG00000008647	2.37377572	9.69E-46
ENSSSCG00000017103	2.371332712	6.08E-06
ENSSSCG00000034570	2.370961604	6.51E-11
ENSSSCG00000036504	2.368243632	6.80E-07
ENSSSCG00000045358	2.363584027	0.01304636
ENSSSCG00000045833	2.358686725	2.04E-11
ENSSSCG00000035297	2.35689838	6.31E-16
ENSSSCG00000038459	2.348155134	1.93E-06
ENSSSCG00000009334	2.345240358	5.67E-35
ENSSSCG00000017392	2.34235797	1.58E-06
ENSSSCG00000000774	2.334935646	2.89E-37
ENSSSCG00000044948	2.332254948	9.62E-08
ENSSSCG00000007062	2.33096079	0.001480279
ENSSSCG00000048696	2.330942844	0.000257116
ENSSSCG00000007861	2.313585364	5.80E-07
ENSSSCG00000036122	2.30015714	0.009722737
ENSSSCG00000039023	2.295932072	0.001227357
ENSSSCG00000032436	2.29327075	9.79E-37
ENSSSCG00000006477	2.292468806	0.000348545
ENSSSCG00000017285	2.283085998	0.000295764
ENSSSCG00000016449	2.281767483	1.31E-11
ENSSSCG00000039273	2.281376135	0.001685549
ENSSSCG00000040431	2.274130971	0.000354708
ENSSSCG00000012853	2.270465258	2.81E-26
ENSSSCG00000038601	2.269745288	1.25E-05
ENSSSCG00000043470	2.265623063	9.01E-05
ENSSSCG00000021597	2.264370038	1.19E-14
ENSSSCG00000051689	2.263510624	7.77E-07
ENSSSCG00000027045	2.263054314	0.001737239
ENSSSCG00000036383	2.262112282	1.97E-10
ENSSSCG00000047775	2.260346307	0.004882206
ENSSSCG00000002510	2.25720415	0.010882124
ENSSSCG00000034821	2.255000832	9.92E-13
ENSSSCG00000045922	2.25427659	0.006566048
ENSSSCG00000012018	2.246343146	0.000403814
ENSSSCG00000036746	2.24550147	3.01E-07
ENSSSCG00000024311	2.244481584	1.60E-07
ENSSSCG00000011375	2.240101656	3.34E-14
ENSSSCG00000016401	2.236776969	3.23E-16
ENSSSCG00000040317	2.230234703	0.007699124
ENSSSCG00000017416	2.207917669	3.73E-17

ENSSSCG00000007508	2.197838158	4.10E-15
ENSSSCG00000041954	2.190641727	2.17E-26
ENSSSCG00000041360	2.186411036	1.20E-08
ENSSSCG00000017700	2.182711954	1.77E-05
ENSSSCG00000016218	2.179303628	0.000471184
ENSSSCG00000021241	2.177033987	3.05E-05
ENSSSCG00000026067	2.175997911	0.000526334
ENSSSCG00000045512	2.174753987	3.35E-07
ENSSSCG00000002908	2.174243136	1.21E-06
ENSSSCG00000021271	2.174226333	4.80E-05
ENSSSCG00000029761	2.172617613	1.13E-07
ENSSSCG00000012857	2.162648806	1.86E-10
ENSSSCG00000015019	2.161470869	1.06E-18
ENSSSCG00000032591	2.15766458	2.41E-19
ENSSSCG00000022089	2.156238245	1.66E-11
ENSSSCG00000014565	2.15039864	2.32E-20
ENSSSCG00000034131	2.147983932	3.20E-05
ENSSSCG00000045225	2.144937455	8.91E-11
ENSSSCG00000011798	2.14435576	5.51E-15
ENSSSCG00000038912	2.143171051	2.47E-24
ENSSSCG00000046561	2.142132755	0.009402029
ENSSSCG00000017705	2.140057019	2.17E-05
ENSSSCG00000037929	2.133188556	6.78E-06
ENSSSCG00000050281	2.130125084	0.000175873
ENSSSCG00000008550	2.12730789	3.06E-07
ENSSSCG00000046044	2.123898915	0.010719338
ENSSSCG00000033913	2.119459327	5.32E-14
ENSSSCG00000036408	2.113710734	0.006417652
ENSSSCG00000017915	2.103111882	9.23E-10
ENSSSCG00000001573	2.091237022	9.61E-11
ENSSSCG00000039962	2.085682552	9.03E-11
ENSSSCG00000033606	2.08485679	1.94E-10
ENSSSCG00000027607	2.080583254	1.63E-05
ENSSSCG00000001565	2.077554632	7.22E-16
ENSSSCG00000027847	2.074427991	3.72E-12
ENSSSCG00000038610	2.065735337	9.36E-06
ENSSSCG00000009945	2.063448212	8.87E-05
ENSSSCG00000017379	2.063282573	2.06E-09
ENSSSCG00000015453	2.060728883	6.87E-10
ENSSSCG00000039854	2.059702687	0.001463118
ENSSSCG00000032963	2.05718362	1.99E-07
ENSSSCG00000006378	2.056995901	4.38E-17
ENSSSCG00000034215	2.055375693	1.46E-05
ENSSSCG00000041733	2.054656555	0.001189894
ENSSSCG00000040379	2.045812086	0.001053155
ENSSSCG00000006717	2.044772224	3.50E-21
ENSSSCG00000009135	2.042443866	2.57E-06
ENSSSCG00000027855	2.040349473	1.60E-24
ENSSSCG00000017396	2.039662703	1.39E-12
ENSSSCG00000010271	2.031677498	0.000260488
ENSSSCG00000016677	2.030525294	6.58E-07
ENSSSCG00000037475	2.024096741	1.85E-05
ENSSSCG00000002637	2.022583965	0.000190524
ENSSSCG00000032250	2.022220529	0.001482513
ENSSSCG00000042788	2.021991582	3.38E-16
ENSSSCG00000046655	2.021895626	0.00491357
ENSSSCG00000005911	2.021809724	4.48E-06
ENSSSCG00000012076	2.021565151	1.96E-26

ENSSSCG00000000186	2.020616707	7.12E-06
ENSSSCG00000017208	2.020093119	7.64E-11
ENSSSCG00000008648	2.016944734	6.12E-07
ENSSSCG00000041745	2.012575566	2.93E-10
ENSSSCG00000035344	2.009847406	1.63E-32
ENSSSCG00000022301	2.007932827	1.89E-07
ENSSSCG00000002768	2.001374291	1.15E-17
ENSSSCG00000030996	1.999310449	4.56E-08
ENSSSCG00000038545	1.996641084	0.007574021
ENSSSCG00000002855	1.992006303	1.92E-23
ENSSSCG00000000197	1.991611652	0.001330133
ENSSSCG00000010452	1.983748781	8.97E-14
ENSSSCG00000008823	1.98342095	1.66E-05
ENSSSCG00000033041	1.982985429	3.71E-07
ENSSSCG00000000849	1.982212413	5.20E-09
ENSSSCG00000048903	1.981235023	0.001381742
ENSSSCG00000015215	1.980234562	1.58E-28
ENSSSCG00000012277	1.979594367	2.03E-11
ENSSSCG00000000184	1.976006151	0.00119102
ENSSSCG00000030548	1.971824778	1.36E-18
ENSSSCG00000003509	1.970462653	0.002143868
ENSSSCG00000022925	1.968510304	7.81E-06
ENSSSCG00000005661	1.968226946	4.86E-06
ENSSSCG00000007995	1.9677001	2.76E-08
ENSSSCG00000024892	1.967073785	0.001641085
ENSSSCG00000049172	1.964208695	0.000631656
ENSSSCG00000009795	1.963264699	0.006387413
ENSSSCG00000000688	1.962320779	6.96E-07
ENSSSCG00000031106	1.95930427	5.81E-06
ENSSSCG00000016153	1.958755827	0.013188816
ENSSSCG00000006512	1.958622966	0.000135343
ENSSSCG00000026414	1.955360374	2.09E-08
ENSSSCG00000011680	1.95406819	7.94E-05
ENSSSCG00000003399	1.952160844	0.000329483
ENSSSCG00000033993	1.948802754	3.57E-05
ENSSSCG00000000401	1.946571785	0.00103482
ENSSSCG00000015016	1.945457383	4.65E-21
ENSSSCG00000008318	1.939789348	0.004239912
ENSSSCG00000021411	1.936078921	6.35E-07
ENSSSCG00000022786	1.933854907	9.87E-17
ENSSSCG00000041010	1.926817415	4.17E-11
ENSSSCG00000028879	1.925822754	4.23E-11
ENSSSCG00000042281	1.924171948	0.011313504
ENSSSCG00000026587	1.922632993	9.75E-08
ENSSSCG00000012845	1.91524727	6.94E-06
ENSSSCG00000002746	1.9151163	6.15E-07
ENSSSCG00000034044	1.914736131	0.000493958
ENSSSCG00000041968	1.91402134	0.00129573
ENSSSCG00000040183	1.90822279	5.63E-15
ENSSSCG00000023423	1.90810826	3.80E-14
ENSSSCG00000042626	1.907670827	0.005215666
ENSSSCG00000043513	1.907178928	0.004183696
ENSSSCG00000031085	1.906799287	2.64E-08
ENSSSCG00000000685	1.904895104	6.92E-12
ENSSSCG00000010261	1.904629647	2.13E-19
ENSSSCG00000042512	1.904191487	3.36E-10
ENSSSCG00000017723	1.902829601	2.35E-31
ENSSSCG00000007009	1.90155654	0.010127712

ENSSSCG00000015231	1.898081428	1.14E-29
ENSSSCG00000040294	1.895544605	2.08E-14
ENSSSCG00000013637	1.893912834	1.51E-05
ENSSSCG00000043207	1.8923645	1.17E-10
ENSSSCG00000021712	1.884870409	4.45E-26
ENSSSCG00000016817	1.874671796	3.26E-10
ENSSSCG00000016714	1.873387822	2.50E-10
ENSSSCG00000001561	1.871504934	9.26E-11
ENSSSCG00000026423	1.868241347	8.36E-34
ENSSSCG00000006771	1.86751653	1.65E-20
ENSSSCG00000034802	1.86559996	1.40E-27
ENSSSCG00000008878	1.865492737	1.98E-11
ENSSSCG00000044908	1.865467161	6.74E-05
ENSSSCG00000016215	1.860296689	9.36E-16
ENSSSCG00000009469	1.860166027	1.01E-14
ENSSSCG00000010461	1.858680896	9.48E-06
ENSSSCG00000023837	1.856128066	5.37E-11
ENSSSCG00000049264	1.846614375	0.000938555
ENSSSCG00000031532	1.846561457	0.000343114
ENSSSCG00000039300	1.842999684	6.30E-07
ENSSSCG00000021815	1.842540534	2.22E-18
ENSSSCG00000009592	1.84152716	0.000445245
ENSSSCG00000013654	1.840146107	0.012287774
ENSSSCG00000049657	1.837000371	3.52E-06
ENSSSCG00000003763	1.826784778	2.72E-15
ENSSSCG00000002516	1.825200942	2.29E-19
ENSSSCG00000037771	1.824070621	0.006123285
ENSSSCG00000028331	1.823746249	0.007979205
ENSSSCG00000016254	1.821875908	0.000320436
ENSSSCG00000035171	1.821371504	4.66E-05
ENSSSCG00000017255	1.820305413	9.30E-06
ENSSSCG00000015448	1.819876475	0.006216852
ENSSSCG00000005134	1.819587195	7.40E-06
ENSSSCG00000039713	1.819482289	7.71E-10
ENSSSCG00000047395	1.812311811	1.04E-07
ENSSSCG00000037110	1.810345601	0.004795925
ENSSSCG00000036883	1.809400843	3.67E-06
ENSSSCG00000049262	1.802941057	0.001550988
ENSSSCG00000051235	1.802888473	0.004598003
ENSSSCG00000006835	1.802137387	2.05E-08
ENSSSCG00000003973	1.801234654	3.02E-06
ENSSSCG00000029567	1.797894799	0.006290471
ENSSSCG00000033381	1.797873687	1.69E-20
ENSSSCG00000017517	1.795100679	5.21E-28
ENSSSCG00000006398	1.795068818	5.69E-15
ENSSSCG00000015786	1.792786765	7.91E-08
ENSSSCG00000035473	1.792380492	1.78E-05
ENSSSCG00000047029	1.791999014	0.009572497
ENSSSCG00000004912	1.789688893	3.22E-10
ENSSSCG00000021620	1.783681422	7.81E-19
ENSSSCG00000021181	1.778655403	1.25E-08
ENSSSCG00000000743	1.776109733	7.91E-12
ENSSSCG00000012375	1.771501289	2.16E-23
ENSSSCG00000005678	1.770084075	5.93E-05
ENSSSCG00000023379	1.764858885	9.36E-19
ENSSSCG00000012950	1.763167939	4.29E-05
ENSSSCG00000004908	1.762267378	9.97E-12
ENSSSCG00000033741	1.761451609	1.74E-05

ENSSSCG00000043014	1.760494684	7.36E-05
ENSSSCG00000015106	1.760005652	2.34E-15
ENSSSCG00000003968	1.758585692	0.00826275
ENSSSCG00000044013	1.758321992	1.05E-08
ENSSSCG00000003972	1.755976379	0.001232443
ENSSSCG00000011450	1.752836665	0.000111009
ENSSSCG00000013436	1.750627163	5.03E-06
ENSSSCG00000005385	1.749333774	3.28E-10
ENSSSCG00000016513	1.747647938	0.00015827
ENSSSCG00000007589	1.746744234	2.32E-05
ENSSSCG00000045365	1.744976148	0.008898613
ENSSSCG00000013082	1.741109971	9.78E-20
ENSSSCG00000038348	1.741104362	2.01E-06
ENSSSCG00000051557	1.739938723	0.000621909
ENSSSCG00000032315	1.738434303	8.51E-11
ENSSSCG00000029160	1.736653156	0.000719947
ENSSSCG00000037660	1.735394604	3.05E-05
ENSSSCG00000035434	1.735269697	3.19E-10
ENSSSCG00000040386	1.73113589	1.12E-31
ENSSSCG00000006729	1.72808945	0.0001722
ENSSSCG00000016077	1.727954372	2.79E-07
ENSSSCG00000008553	1.719117425	8.14E-23
ENSSSCG00000011714	1.715145426	2.22E-07
ENSSSCG00000034688	1.714908285	0.005712522
ENSSSCG00000039770	1.712980699	2.08E-05
ENSSSCG00000051503	1.710820576	0.003082044
ENSSSCG00000037642	1.709789727	5.91E-19
ENSSSCG00000013408	1.707652919	3.98E-07
ENSSSCG00000037478	1.706882494	0.003800822
ENSSSCG00000040663	1.704936762	4.32E-21
ENSSSCG00000007717	1.704683002	0.003076092
ENSSSCG00000024233	1.701431171	4.52E-07
ENSSSCG00000026680	1.698501038	0.011227639
ENSSSCG00000022380	1.697899419	0.010180992
ENSSSCG00000031633	1.691768594	7.22E-10
ENSSSCG00000043070	1.687037861	0.001658522
ENSSSCG00000046117	1.686552546	0.005645061
ENSSSCG00000036724	1.685905533	2.33E-06
ENSSSCG00000005601	1.682654225	2.58E-12
ENSSSCG00000003105	1.682602967	9.22E-08
ENSSSCG00000047578	1.681200362	0.000102907
ENSSSCG00000008077	1.68044199	9.74E-09
ENSSSCG00000015001	1.677636239	1.42E-05
ENSSSCG00000023178	1.675839742	2.63E-08
ENSSSCG00000002767	1.674753603	1.43E-08
ENSSSCG00000016469	1.67167929	2.04E-07
ENSSSCG00000002849	1.670929941	0.000516805
ENSSSCG00000039162	1.668071418	0.004208083
ENSSSCG00000030165	1.667347003	9.11E-08
ENSSSCG00000032768	1.666064215	7.11E-08
ENSSSCG00000009100	1.66189164	0.010568006
ENSSSCG00000011965	1.657333016	0.000217608
ENSSSCG00000039662	1.655956348	4.20E-10
ENSSSCG00000036824	1.655518225	1.72E-13
ENSSSCG00000026605	1.655348721	0.001291451
ENSSSCG00000011806	1.653503473	3.86E-11
ENSSSCG00000015850	1.653136007	2.41E-05
ENSSSCG00000005490	1.65280105	6.77E-08

ENSSSCG00000014898	1.651423225	7.21E-13
ENSSSCG00000009964	1.651229749	0.000643864
ENSSSCG00000036363	1.648718564	0.007294633
ENSSSCG00000014374	1.64824983	2.83E-06
ENSSSCG00000034216	1.647662958	6.21E-19
ENSSSCG00000006560	1.646108009	2.42E-06
ENSSSCG00000029757	1.644738197	0.002297993
ENSSSCG00000026318	1.64372243	0.001119562
ENSSSCG00000028978	1.643621419	9.05E-05
ENSSSCG00000043852	1.64277288	0.011891592
ENSSSCG00000035337	1.642278232	0.002230554
ENSSSCG00000037431	1.638716334	1.36E-08
ENSSSCG00000006552	1.635826301	2.73E-18
ENSSSCG00000032082	1.635334312	0.005040444
ENSSSCG00000036887	1.634680943	3.68E-08
ENSSSCG00000024388	1.631798056	1.35E-12
ENSSSCG00000017128	1.631540947	5.11E-11
ENSSSCG00000010073	1.63025899	1.18E-07
ENSSSCG00000017758	1.629904264	0.001120626
ENSSSCG00000047841	1.629298759	0.000224617
ENSSSCG00000027226	1.628988013	0.000420608
ENSSSCG00000006867	1.628846995	0.000304781
ENSSSCG00000007423	1.625984139	7.58E-17
ENSSSCG00000005925	1.624348628	5.19E-05
ENSSSCG00000003343	1.621596019	3.69E-05
ENSSSCG00000048147	1.620431575	0.000199324
ENSSSCG00000007753	1.619774348	3.77E-08
ENSSSCG00000038112	1.618774821	0.002441949
ENSSSCG00000043747	1.605513962	4.76E-10
ENSSSCG00000026850	1.605231628	7.88E-05
ENSSSCG00000017544	1.604458475	8.42E-12
ENSSSCG00000027689	1.603563584	6.59E-05
ENSSSCG00000037168	1.602472142	0.00243785
ENSSSCG00000006360	1.602451441	4.97E-08
ENSSSCG00000002135	1.601653203	0.002332
ENSSSCG00000023173	1.601573993	1.48E-07
ENSSSCG00000024344	1.600679814	9.48E-06
ENSSSCG00000040060	1.599949844	0.00034711
ENSSSCG00000036956	1.599367155	7.96E-07
ENSSSCG00000003338	1.59933796	2.86E-05
ENSSSCG00000023177	1.598610966	0.00089558
ENSSSCG00000016720	1.598327359	1.62E-08
ENSSSCG00000006948	1.597523514	0.002094602
ENSSSCG00000003552	1.597326855	8.38E-05
ENSSSCG00000034207	1.593991081	6.42E-08
ENSSSCG00000021161	1.592747747	1.47E-07
ENSSSCG00000037354	1.587377091	0.000176211
ENSSSCG00000030408	1.587047319	8.81E-13
ENSSSCG00000038434	1.584741746	0.000719564
ENSSSCG00000000975	1.58473228	1.33E-05
ENSSSCG00000014830	1.583163532	8.40E-09
ENSSSCG00000027526	1.583041069	3.53E-13
ENSSSCG00000041665	1.579746384	4.24E-05
ENSSSCG00000023318	1.579023873	6.38E-09
ENSSSCG00000011561	1.579011042	3.66E-13
ENSSSCG00000024479	1.577318274	4.64E-05
ENSSSCG00000003135	1.576810004	1.93E-06
ENSSSCG00000008691	1.576345137	3.08E-07

ENSSSCG00000017924	1.574518893	2.60E-15
ENSSSCG00000002007	1.574502692	0.007718802
ENSSSCG00000039635	1.573511061	2.44E-05
ENSSSCG00000005102	1.573193376	1.84E-05
ENSSSCG00000037099	1.571962888	8.00E-11
ENSSSCG00000038401	1.570422933	9.34E-05
ENSSSCG00000027237	1.570016941	0.000261592
ENSSSCG00000017507	1.56932086	0.001880121
ENSSSCG00000003089	1.56784983	4.83E-05
ENSSSCG00000048041	1.567157474	0.004881467
ENSSSCG00000038308	1.56676459	0.000150266
ENSSSCG00000002036	1.566663207	2.05E-10
ENSSSCG00000024872	1.563715152	1.70E-11
ENSSSCG00000017754	1.561270769	2.63E-09
ENSSSCG00000049234	1.560491772	1.54E-14
ENSSSCG00000003616	1.55781355	4.68E-16
ENSSSCG00000006831	1.556832124	0.000300587
ENSSSCG00000028019	1.556758013	1.14E-30
ENSSSCG00000039799	1.55663516	2.38E-15
ENSSSCG00000020915	1.555269432	1.35E-10
ENSSSCG00000011133	1.554007744	2.05E-05
ENSSSCG00000013492	1.552561869	1.31E-13
ENSSSCG00000010655	1.549932812	1.21E-19
ENSSSCG00000038190	1.549640238	1.24E-12
ENSSSCG00000000406	1.548960762	2.87E-06
ENSSSCG00000047727	1.547764821	0.002348736
ENSSSCG00000022797	1.545240991	1.47E-13
ENSSSCG00000016122	1.544875778	1.72E-18
ENSSSCG00000030681	1.541695288	0.004377371
ENSSSCG00000035052	1.541385906	0.002496751
ENSSSCG00000050094	1.540485844	0.002208615
ENSSSCG00000008976	1.539915844	0.00190236
ENSSSCG00000034994	1.538498928	0.000567375
ENSSSCG00000032115	1.537207227	0.006422134
ENSSSCG00000032390	1.536007254	1.51E-18
ENSSSCG00000001505	1.533736085	3.64E-05
ENSSSCG00000013303	1.532215274	0.000663155
ENSSSCG00000033113	1.532140169	1.82E-18
ENSSSCG00000013226	1.530353052	1.26E-11
ENSSSCG00000007552	1.529372279	0.00025505
ENSSSCG00000025423	1.525996811	5.02E-12
ENSSSCG00000008729	1.524911752	0.000476258
ENSSSCG00000033700	1.523519502	5.29E-07
ENSSSCG00000009881	1.523225757	1.49E-16
ENSSSCG00000010576	1.522982307	0.000270449
ENSSSCG00000037782	1.522895402	5.45E-08
ENSSSCG00000024550	1.520091408	7.18E-09
ENSSSCG00000002723	1.520060345	1.10E-09
ENSSSCG00000007228	1.519700772	2.51E-10
ENSSSCG00000002891	1.518022617	9.94E-05
ENSSSCG00000027478	1.517147698	3.42E-08
ENSSSCG00000010086	1.516730953	0.002629578
ENSSSCG00000011451	1.516628791	0.000125653
ENSSSCG00000030561	1.512865964	0.001621093
ENSSSCG00000011675	1.512131541	0.00182796
ENSSSCG00000003600	1.510389229	2.41E-07
ENSSSCG00000024166	1.508941025	4.02E-10
ENSSSCG00000013366	1.508833682	7.03E-07

ENSSSCG00000040236	1.507989509	1.80E-08
ENSSSCG00000006527	1.504637041	1.78E-11
ENSSSCG00000021418	1.501610692	0.000307493
ENSSSCG00000006380	1.500401992	0.007894084
ENSSSCG00000024596	1.497414377	0.00096327
ENSSSCG00000016078	1.496783899	2.32E-07
ENSSSCG00000010495	1.49655948	1.38E-10
ENSSSCG00000038562	1.496552864	1.41E-05
ENSSSCG00000000950	1.496254353	9.53E-23
ENSSSCG00000011621	1.493559856	9.21E-10
ENSSSCG00000036136	1.492488971	0.000529746
ENSSSCG00000012171	1.492207716	6.64E-12
ENSSSCG00000014338	1.491859724	2.43E-05
ENSSSCG00000007355	1.489126168	2.14E-11
ENSSSCG00000006481	1.486936477	0.000121469
ENSSSCG00000003266	1.484196447	0.001196128
ENSSSCG00000010136	1.484164792	6.56E-07
ENSSSCG00000022237	1.483656036	8.21E-11
ENSSSCG00000031321	1.483280526	0.000568575
ENSSSCG00000011620	1.481442398	3.94E-05
ENSSSCG00000015579	1.481412385	0.001064283
ENSSSCG00000034829	1.480042951	0.001179272
ENSSSCG00000037416	1.478494048	1.63E-06
ENSSSCG00000033374	1.478373773	3.51E-05
ENSSSCG00000012823	1.477476212	0.001585752
ENSSSCG00000034147	1.47630105	4.06E-05
ENSSSCG00000030415	1.474915039	1.16E-13
ENSSSCG00000008002	1.473779977	7.62E-13
ENSSSCG00000021610	1.472203162	6.87E-07
ENSSSCG00000030177	1.471997156	0.000137172
ENSSSCG00000002406	1.471329	2.72E-10
ENSSSCG00000011604	1.470074752	3.42E-05
ENSSSCG00000016919	1.469905954	0.000430147
ENSSSCG00000050848	1.467917171	0.005712596
ENSSSCG00000025486	1.467847745	8.00E-06
ENSSSCG00000043560	1.467281724	5.59E-05
ENSSSCG00000021130	1.466785525	0.000102877
ENSSSCG00000007749	1.465454389	1.62E-06
ENSSSCG00000030578	1.46506599	7.60E-13
ENSSSCG00000004033	1.464495438	2.25E-09
ENSSSCG00000011393	1.463641782	4.14E-16
ENSSSCG00000003566	1.461389345	1.49E-07
ENSSSCG00000023557	1.459370251	2.18E-05
ENSSSCG00000012791	1.459367892	0.000494166
ENSSSCG00000001589	1.458639649	0.013016366
ENSSSCG00000013745	1.457900523	2.53E-07
ENSSSCG00000017571	1.457083072	4.19E-11
ENSSSCG00000008496	1.455021552	3.01E-16
ENSSSCG00000008633	1.453935766	9.64E-12
ENSSSCG00000001516	1.453699686	5.40E-25
ENSSSCG00000043494	1.453654662	0.001130798
ENSSSCG00000050463	1.452546034	0.000587347
ENSSSCG00000000722	1.449255698	0.001155995
ENSSSCG00000046259	1.44824345	0.005793001
ENSSSCG00000031888	1.446060046	1.15E-05
ENSSSCG00000035565	1.445707019	1.36E-20
ENSSSCG00000021576	1.444573217	0.004327621
ENSSSCG00000011000	1.443091238	2.26E-15

ENSSSCG00000004330	1.439811505	0.012259041
ENSSSCG00000035048	1.439219355	0.00043403
ENSSSCG00000002706	1.436474429	7.68E-09
ENSSSCG00000008703	1.433954779	6.78E-05
ENSSSCG00000017466	1.428773663	6.50E-05
ENSSSCG00000004826	1.427092677	8.06E-06
ENSSSCG00000033982	1.425097624	6.03E-12
ENSSSCG00000030510	1.424666969	0.000814762
ENSSSCG00000010058	1.424332154	8.31E-06
ENSSSCG00000036224	1.424248816	0.002190008
ENSSSCG00000002138	1.422982773	5.09E-11
ENSSSCG00000034696	1.419438831	0.004559057
ENSSSCG00000016745	1.418690883	3.79E-06
ENSSSCG00000011447	1.418366218	2.08E-10
ENSSSCG00000025965	1.417876867	2.74E-07
ENSSSCG00000024655	1.416956723	1.83E-05
ENSSSCG00000031847	1.41687879	4.27E-05
ENSSSCG00000008590	1.414970098	0.000102355
ENSSSCG00000007133	1.411277415	9.48E-06
ENSSSCG00000008268	1.410937874	9.01E-10
ENSSSCG00000006366	1.410743463	1.36E-05
ENSSSCG00000004657	1.408069535	2.54E-09
ENSSSCG00000038950	1.401502164	0.000129334
ENSSSCG00000013853	1.400149373	8.38E-17
ENSSSCG00000008010	1.398105223	2.28E-09
ENSSSCG00000028889	1.397332133	2.65E-11
ENSSSCG00000023604	1.397141443	1.28E-25
ENSSSCG00000010599	1.397023525	0.000137951
ENSSSCG00000010821	1.396708042	1.76E-06
ENSSSCG00000009137	1.395268816	8.99E-05
ENSSSCG00000009069	1.394921384	0.001866475
ENSSSCG00000016441	1.391175671	3.31E-07
ENSSSCG00000021305	1.389273653	0.000868947
ENSSSCG00000000082	1.387868744	0.004629514
ENSSSCG00000037077	1.387826732	8.78E-10
ENSSSCG00000036015	1.387793115	1.69E-07
ENSSSCG00000027538	1.387666152	8.66E-10
ENSSSCG00000015652	1.387052833	0.000532293
ENSSSCG00000013475	1.386812047	2.67E-06
ENSSSCG00000010575	1.386406739	0.000236075
ENSSSCG00000003582	1.385358766	1.32E-07
ENSSSCG00000026229	1.384796311	3.95E-12
ENSSSCG00000032367	1.383033496	0.000156028
ENSSSCG00000045632	1.382663742	9.53E-08
ENSSSCG00000033050	1.382468059	5.94E-08
ENSSSCG00000049887	1.381414458	1.44E-07
ENSSSCG00000016653	1.380863025	0.000461761
ENSSSCG00000008303	1.38045371	1.62E-07
ENSSSCG00000023850	1.380353654	0.000436182
ENSSSCG00000001232	1.378066401	0.002774801
ENSSSCG00000011061	1.377772155	0.000789558
ENSSSCG00000000171	1.376817255	4.04E-08
ENSSSCG00000038420	1.375966626	0.009261832
ENSSSCG00000046817	1.375732194	2.45E-06
ENSSSCG00000041416	1.375199448	0.003189301
ENSSSCG00000040486	1.374602194	5.43E-07
ENSSSCG00000003861	1.374414756	4.32E-07
ENSSSCG00000013591	1.370727885	0.00105108

ENSSSCG00000014289	1.369718492	1.06E-06
ENSSSCG00000051499	1.368353147	0.00405448
ENSSSCG00000002535	1.368302783	2.43E-16
ENSSSCG00000011893	1.368069069	6.93E-14
ENSSSCG00000031485	1.366904575	0.001461407
ENSSSCG00000034898	1.366768648	6.55E-14
ENSSSCG00000010315	1.364218942	6.08E-05
ENSSSCG00000040352	1.364109066	9.03E-05
ENSSSCG00000004480	1.363797486	1.41E-12
ENSSSCG00000012352	1.363421367	0.002440522
ENSSSCG00000026617	1.36302918	2.96E-05
ENSSSCG00000050795	1.362670213	5.54E-07
ENSSSCG00000012007	1.361914004	2.46E-06
ENSSSCG00000040990	1.360841252	4.99E-05
ENSSSCG00000011560	1.359509829	2.16E-07
ENSSSCG00000021828	1.359349505	4.39E-15
ENSSSCG00000008282	1.357559463	6.92E-05
ENSSSCG00000048948	1.356916311	8.87E-05
ENSSSCG00000010245	1.355388637	0.010596077
ENSSSCG00000034137	1.354940305	6.98E-18
ENSSSCG00000037343	1.354155905	4.06E-08
ENSSSCG00000024018	1.353447425	8.02E-09
ENSSSCG00000013746	1.351936044	2.24E-14
ENSSSCG00000042527	1.351911726	3.53E-05
ENSSSCG00000024692	1.350613012	7.14E-05
ENSSSCG00000030567	1.349072777	5.58E-17
ENSSSCG00000035193	1.348924516	2.79E-08
ENSSSCG00000026297	1.348688006	2.67E-08
ENSSSCG00000028202	1.348365542	2.60E-12
ENSSSCG00000006153	1.348107225	0.000249889
ENSSSCG00000034756	1.347250969	0.000100883
ENSSSCG00000017682	1.347068879	8.99E-06
ENSSSCG00000008693	1.346799018	0.000386506
ENSSSCG00000001509	1.346276544	2.91E-11
ENSSSCG00000046222	1.342697435	0.000871858
ENSSSCG00000001041	1.342611161	0.001297819
ENSSSCG00000011106	1.34229274	3.25E-07
ENSSSCG00000003042	1.341448103	0.000132921
ENSSSCG00000004057	1.341308653	3.44E-14
ENSSSCG00000026904	1.340691318	0.000737213
ENSSSCG00000032060	1.33940884	1.28E-07
ENSSSCG00000034814	1.338448359	0.002586279
ENSSSCG00000031991	1.338085289	1.09E-06
ENSSSCG00000000635	1.337940951	0.000439216
ENSSSCG00000029438	1.336737593	8.73E-08
ENSSSCG00000023298	1.334945916	1.34E-05
ENSSSCG00000025027	1.334863513	0.000611056
ENSSSCG00000040355	1.334190079	3.19E-10
ENSSSCG00000000811	1.33379741	9.01E-08
ENSSSCG00000032422	1.333024607	0.000162003
ENSSSCG00000032234	1.33235507	5.78E-10
ENSSSCG00000013423	1.332169617	3.44E-06
ENSSSCG00000001773	1.330942126	7.46E-16
ENSSSCG00000041731	1.33037228	0.000405783
ENSSSCG00000034716	1.330197778	3.13E-07
ENSSSCG00000000394	1.32953747	4.77E-10
ENSSSCG00000022689	1.329385626	1.66E-05
ENSSSCG00000051454	1.329209465	0.006032075

ENSSSCG00000037465	1.328334523	9.39E-08
ENSSSCG00000010514	1.32830665	0.004884603
ENSSSCG00000025729	1.327534069	5.36E-10
ENSSSCG00000021319	1.327470608	5.55E-06
ENSSSCG00000037120	1.326313055	7.46E-14
ENSSSCG00000043909	1.32523422	1.16E-14
ENSSSCG00000032819	1.324273191	2.11E-10
ENSSSCG00000008157	1.323776456	0.002506803
ENSSSCG00000040875	1.323593024	9.05E-05
ENSSSCG00000002004	1.323203993	3.12E-09
ENSSSCG00000036257	1.322344695	0.000344619
ENSSSCG00000048408	1.322196222	2.11E-10
ENSSSCG00000039494	1.320814182	0.000361569
ENSSSCG00000010795	1.320332653	6.94E-09
ENSSSCG00000026612	1.319413833	0.000187424
ENSSSCG00000002546	1.316624247	1.55E-05
ENSSSCG00000000116	1.316600556	5.42E-05
ENSSSCG00000000749	1.31607783	1.17E-07
ENSSSCG00000007973	1.315703653	0.001307901
ENSSSCG00000011318	1.315151117	1.53E-08
ENSSSCG00000009145	1.31489018	1.29E-10
ENSSSCG00000006812	1.314609627	0.007275871
ENSSSCG00000028962	1.314321879	2.82E-11
ENSSSCG00000004548	1.313899312	0.000127018
ENSSSCG00000035775	1.313773738	0.001847969
ENSSSCG00000029268	1.313650092	1.83E-08
ENSSSCG00000025107	1.313419514	0.000446993
ENSSSCG00000010528	1.312733771	0.000106796
ENSSSCG00000011612	1.312712674	8.42E-14
ENSSSCG00000010608	1.312162238	0.003157409
ENSSSCG00000017971	1.311734624	1.17E-06
ENSSSCG00000014835	1.311178057	9.31E-18
ENSSSCG00000004047	1.31115349	5.26E-11
ENSSSCG00000040010	1.310238778	0.006246161
ENSSSCG00000009720	1.30893242	9.20E-08
ENSSSCG00000010087	1.308285768	2.10E-05
ENSSSCG00000008017	1.307332954	1.02E-10
ENSSSCG00000001990	1.30729907	2.26E-06
ENSSSCG00000034655	1.306655275	2.27E-08
ENSSSCG00000000045	1.306121428	1.08E-06
ENSSSCG00000028465	1.305818849	0.000810084
ENSSSCG00000011449	1.305273159	0.001233715
ENSSSCG00000001513	1.305142483	5.40E-08
ENSSSCG00000033453	1.304459813	8.27E-14
ENSSSCG00000011196	1.304232164	2.28E-09
ENSSSCG00000042921	1.304007643	0.010380975
ENSSSCG00000033655	1.30368886	6.78E-13
ENSSSCG00000038359	1.30336083	0.000492582
ENSSSCG00000035739	1.302562621	4.35E-05
ENSSSCG00000014250	1.301518103	4.40E-06
ENSSSCG00000000133	1.300693711	1.45E-05
ENSSSCG00000041898	1.300280427	0.004853027
ENSSSCG00000048448	1.298225528	0.000121163
ENSSSCG00000002709	1.297522122	6.24E-05
ENSSSCG00000048981	1.29704244	0.000916342
ENSSSCG00000003109	1.296419162	4.16E-09
ENSSSCG00000039867	1.293521819	0.001335559
ENSSSCG00000030108	1.293452116	5.28E-10

ENSSSCG00000047083	1.293181233	2.53E-08
ENSSSCG00000027372	1.293065464	1.48E-09
ENSSSCG00000008195	1.29228021	7.30E-07
ENSSSCG00000005376	1.291216593	0.002399394
ENSSSCG00000024693	1.290788175	4.26E-09
ENSSSCG00000014437	1.289570664	0.000536124
ENSSSCG00000030369	1.289381059	6.93E-05
ENSSSCG00000042077	1.289357258	2.12E-05
ENSSSCG00000020856	1.289346175	5.54E-08
ENSSSCG00000002907	1.287801158	5.60E-09
ENSSSCG00000000696	1.285855697	0.002117726
ENSSSCG00000000396	1.284500493	4.33E-07
ENSSSCG00000039830	1.284148366	3.19E-08
ENSSSCG00000016724	1.283527997	6.27E-09
ENSSSCG00000034487	1.282718542	5.62E-10
ENSSSCG00000034738	1.282352231	0.001149467
ENSSSCG00000007493	1.281455047	3.71E-05
ENSSSCG00000000399	1.28105672	6.32E-11
ENSSSCG00000028964	1.280336078	9.79E-11
ENSSSCG00000025796	1.279107535	0.001631206
ENSSSCG00000004670	1.275202101	4.02E-05
ENSSSCG00000007278	1.273433939	0.000632327
ENSSSCG00000020808	1.271650311	0.000119929
ENSSSCG00000047654	1.271535408	0.000494529
ENSSSCG00000010454	1.270762245	2.83E-06
ENSSSCG00000043043	1.26919081	0.004536608
ENSSSCG00000036709	1.267100333	4.77E-11
ENSSSCG00000000895	1.266362766	0.001749806
ENSSSCG00000009931	1.26543691	0.00222321
ENSSSCG00000045534	1.265312541	8.34E-05
ENSSSCG00000008039	1.263156273	1.02E-05
ENSSSCG00000001931	1.25937961	0.000136156
ENSSSCG00000034876	1.258238371	2.74E-09
ENSSSCG00000008094	1.256561513	5.85E-09
ENSSSCG00000005895	1.256391352	0.003193065
ENSSSCG00000016220	1.255888354	1.66E-07
ENSSSCG00000000084	1.255495532	6.22E-06
ENSSSCG00000050193	1.25349364	0.008536911
ENSSSCG00000051586	1.252704797	0.012594787
ENSSSCG00000035839	1.251542225	1.38E-10
ENSSSCG00000006830	1.251325012	5.86E-07
ENSSSCG00000050939	1.250828401	0.000834604
ENSSSCG00000022149	1.249920149	6.26E-06
ENSSSCG00000011389	1.249737364	2.35E-05
ENSSSCG00000026977	1.249437402	5.98E-13
ENSSSCG00000017835	1.248163846	0.000995568
ENSSSCG00000033566	1.248162144	0.002697137
ENSSSCG00000015337	1.247537004	3.25E-06
ENSSSCG00000016732	1.247277831	7.10E-05
ENSSSCG00000042208	1.246499678	7.64E-08
ENSSSCG00000001679	1.245941225	9.61E-06
ENSSSCG00000017549	1.245571555	0.000160894
ENSSSCG00000017717	1.245401235	1.78E-07
ENSSSCG00000013757	1.245224095	4.87E-08
ENSSSCG00000003419	1.244497308	8.07E-09
ENSSSCG00000017205	1.243556675	1.57E-05
ENSSSCG00000037606	1.243008223	2.71E-07
ENSSSCG00000026455	1.242372751	0.000196637

ENSSSCG00000036983	1.241897912	0.001267774
ENSSSCG00000037513	1.241733543	9.12E-07
ENSSSCG00000034329	1.240994971	8.37E-06
ENSSSCG00000034012	1.240216958	9.27E-10
ENSSSCG00000005662	1.239916188	2.81E-05
ENSSSCG00000035357	1.239468132	0.000140781
ENSSSCG00000002459	1.238647826	1.53E-07
ENSSSCG00000022312	1.238573192	1.82E-07
ENSSSCG00000034280	1.237855377	0.003230499
ENSSSCG00000000024	1.237484924	8.76E-05
ENSSSCG00000051054	1.236701993	1.35E-11
ENSSSCG00000029668	1.236179304	3.54E-07
ENSSSCG00000004048	1.236168281	4.66E-06
ENSSSCG00000033051	1.236068039	6.90E-06
ENSSSCG00000031918	1.23516687	5.57E-05
ENSSSCG00000006469	1.235068946	5.11E-07
ENSSSCG00000004164	1.234182912	1.73E-05
ENSSSCG00000013756	1.233419297	0.002206883
ENSSSCG00000046315	1.22960591	0.004595115
ENSSSCG00000004342	1.226994983	0.000983615
ENSSSCG00000040709	1.22566592	0.000138899
ENSSSCG00000017948	1.224064986	0.000491908
ENSSSCG00000013662	1.223896259	0.001385308
ENSSSCG00000049991	1.223318369	0.00315652
ENSSSCG00000033731	1.223245434	7.17E-09
ENSSSCG00000012854	1.222601926	5.79E-10
ENSSSCG00000024562	1.222194846	3.89E-12
ENSSSCG00000045776	1.221383183	0.001955343
ENSSSCG00000017755	1.22075729	2.78E-07
ENSSSCG00000038006	1.220353585	0.010654866
ENSSSCG00000003931	1.219759364	1.18E-05
ENSSSCG00000033757	1.219136719	1.23E-09
ENSSSCG00000000739	1.219005127	8.65E-08
ENSSSCG00000001252	1.218339101	1.47E-05
ENSSSCG00000005908	1.218281725	0.000347382
ENSSSCG00000017196	1.21801676	3.66E-08
ENSSSCG00000002316	1.217940849	3.18E-05
ENSSSCG00000005105	1.217606174	2.37E-07
ENSSSCG00000001440	1.217599821	5.52E-08
ENSSSCG00000024313	1.217429607	8.79E-06
ENSSSCG00000007803	1.216397151	0.000213072
ENSSSCG00000035991	1.216035671	0.001240759
ENSSSCG00000005777	1.215537478	3.64E-08
ENSSSCG00000010007	1.214235635	4.55E-07
ENSSSCG00000040446	1.21400697	0.001178435
ENSSSCG00000005440	1.213226596	1.74E-09
ENSSSCG00000002050	1.213038694	1.08E-15
ENSSSCG00000026784	1.212720122	5.09E-07
ENSSSCG00000038474	1.211570426	2.16E-06
ENSSSCG00000004542	1.211569224	0.000241282
ENSSSCG00000003914	1.21016637	1.97E-05
ENSSSCG00000015525	1.209937765	3.37E-09
ENSSSCG00000009746	1.209754272	0.000644023
ENSSSCG00000035347	1.208851457	2.42E-05
ENSSSCG00000004394	1.208362712	0.000776748
ENSSSCG00000007715	1.207411418	2.37E-15
ENSSSCG00000017427	1.207354616	0.007082106
ENSSSCG00000003332	1.206850975	1.61E-06

ENSSSCG00000035596	1.205922109	0.001322202
ENSSSCG00000013613	1.205253468	1.49E-08
ENSSSCG00000007896	1.203318441	0.000461751
ENSSSCG00000002431	1.20302934	3.20E-05
ENSSSCG00000040095	1.202242804	2.44E-08
ENSSSCG00000015140	1.201875045	2.31E-09
ENSSSCG00000030277	1.200787275	6.17E-13
ENSSSCG00000028602	1.200193999	0.001769939
ENSSSCG00000035618	1.199649054	8.58E-07
ENSSSCG00000003756	1.198606137	0.005611773
ENSSSCG00000016711	1.198559648	0.001330212
ENSSSCG00000011313	1.198255904	2.76E-05
ENSSSCG00000037096	1.19788587	2.84E-05
ENSSSCG00000026472	1.197180037	0.000280928
ENSSSCG00000038700	1.197038054	7.61E-08
ENSSSCG00000036096	1.19683766	6.27E-07
ENSSSCG00000039731	1.195717276	1.85E-15
ENSSSCG00000007720	1.195457083	2.73E-05
ENSSSCG00000027676	1.194779677	0.001429992
ENSSSCG00000003905	1.194699785	3.60E-07
ENSSSCG00000028167	1.194210974	0.001234239
ENSSSCG00000002952	1.194031456	1.78E-12
ENSSSCG00000020754	1.19377781	0.003040858
ENSSSCG00000013457	1.192579243	5.50E-05
ENSSSCG00000002467	1.192370953	8.90E-05
ENSSSCG00000004125	1.190653975	0.000125449
ENSSSCG00000049958	1.190512162	7.97E-05
ENSSSCG00000042444	1.190388153	0.000503002
ENSSSCG00000013118	1.190137519	4.14E-07
ENSSSCG00000034913	1.18982334	2.15E-05
ENSSSCG00000009904	1.188309563	5.02E-12
ENSSSCG00000007816	1.187464362	0.002812497
ENSSSCG00000050998	1.187043627	8.21E-10
ENSSSCG00000025329	1.186673726	1.04E-05
ENSSSCG00000027426	1.186553227	2.13E-06
ENSSSCG00000036614	1.185453336	9.13E-06
ENSSSCG00000034859	1.185162976	0.00033678
ENSSSCG00000031860	1.184866566	1.93E-10
ENSSSCG00000007556	1.184236695	3.23E-05
ENSSSCG00000028227	1.184039695	8.18E-16
ENSSSCG00000032165	1.183992739	8.25E-06
ENSSSCG00000023783	1.182899376	5.55E-10
ENSSSCG00000008677	1.182385996	3.87E-11
ENSSSCG00000026752	1.180499339	0.002000333
ENSSSCG00000016420	1.180302468	2.30E-13
ENSSSCG00000012758	1.18026237	0.003951227
ENSSSCG00000015897	1.180079121	5.95E-14
ENSSSCG00000026761	1.1798067	0.000288221
ENSSSCG00000021494	1.179263398	3.48E-05
ENSSSCG00000002697	1.17912633	2.44E-08
ENSSSCG00000015600	1.178699453	0.000643732
ENSSSCG00000012161	1.178513892	0.002597392
ENSSSCG00000008055	1.178286304	9.10E-06
ENSSSCG00000011624	1.178236676	4.56E-05
ENSSSCG00000023880	1.178093201	0.000762658
ENSSSCG00000017882	1.177359895	0.010280798
ENSSSCG00000003505	1.176810571	3.71E-10
ENSSSCG00000013936	1.175127652	4.69E-07

ENSSSCG00000035914	1.174384999	1.08E-07
ENSSSCG00000016216	1.173840558	2.69E-14
ENSSSCG00000008512	1.173001869	0.000842843
ENSSSCG00000026082	1.172200297	8.08E-07
ENSSSCG00000035578	1.171803071	4.56E-05
ENSSSCG00000009888	1.171381654	3.97E-16
ENSSSCG00000017614	1.169564318	9.34E-11
ENSSSCG00000013003	1.16950218	6.81E-06
ENSSSCG00000037999	1.169393669	0.001030297
ENSSSCG00000001431	1.168900922	2.74E-06
ENSSSCG00000033179	1.16866472	3.81E-06
ENSSSCG00000001392	1.168494411	6.68E-07
ENSSSCG00000006940	1.167250477	0.000107441
ENSSSCG00000030801	1.167119011	8.74E-08
ENSSSCG00000024189	1.166944378	0.002333969
ENSSSCG00000016970	1.16686243	0.0014498
ENSSSCG00000009622	1.166252505	0.000150496
ENSSSCG00000000265	1.165491897	1.20E-05
ENSSSCG00000029425	1.164783033	4.53E-07
ENSSSCG00000006214	1.164245062	0.003915773
ENSSSCG00000041583	1.163681389	0.006185859
ENSSSCG00000011001	1.163542402	0.000272749
ENSSSCG00000005628	1.162304763	0.00333708
ENSSSCG00000032231	1.160899214	0.010756768
ENSSSCG00000026894	1.160796854	6.88E-05
ENSSSCG00000047099	1.16079108	0.000854347
ENSSSCG00000038717	1.160696938	7.90E-07
ENSSSCG00000012943	1.160478597	8.76E-05
ENSSSCG00000021698	1.158377352	2.58E-05
ENSSSCG00000014850	1.156707977	7.70E-07
ENSSSCG00000023400	1.156537035	8.54E-05
ENSSSCG00000031730	1.156389274	1.30E-07
ENSSSCG00000004946	1.154280046	2.46E-06
ENSSSCG00000034779	1.15385434	0.002164608
ENSSSCG00000017146	1.153765767	3.89E-06
ENSSSCG00000008377	1.15343455	2.73E-06
ENSSSCG00000050368	1.153316608	1.95E-05
ENSSSCG00000000377	1.153002911	0.005061765
ENSSSCG00000026422	1.151509808	0.002091033
ENSSSCG00000027121	1.151494122	0.000565354
ENSSSCG00000028696	1.151289123	1.58E-09
ENSSSCG00000038130	1.15108439	1.39E-09
ENSSSCG00000001817	1.150883154	0.010145463
ENSSSCG00000028007	1.149926271	0.003574421
ENSSSCG00000016275	1.149439629	0.005986601
ENSSSCG00000035693	1.148500735	0.00057385
ENSSSCG00000003551	1.148386484	3.15E-07
ENSSSCG00000033089	1.148197107	1.74E-08
ENSSSCG00000013463	1.147968954	0.000685335
ENSSSCG00000020823	1.147519105	5.33E-08
ENSSSCG00000005619	1.146149444	4.52E-05
ENSSSCG00000016210	1.146128367	7.14E-08
ENSSSCG00000022151	1.145473633	0.00437234
ENSSSCG00000041154	1.14543369	0.004354633
ENSSSCG00000029538	1.145152665	8.77E-10
ENSSSCG00000016859	1.145043485	0.000848881
ENSSSCG00000014304	1.143181666	2.74E-06
ENSSSCG00000037132	1.143115847	0.00014889

ENSSSCG00000003198	1.14306596	0.000328456
ENSSSCG00000039216	1.142474741	6.65E-05
ENSSSCG00000015504	1.142344884	3.28E-05
ENSSSCG00000031132	1.141960555	1.64E-08
ENSSSCG00000031474	1.14160121	0.002610081
ENSSSCG00000005355	1.141135354	8.38E-07
ENSSSCG00000025593	1.141090048	4.16E-10
ENSSSCG00000039217	1.140819945	0.011548341
ENSSSCG00000027110	1.140421164	0.001986084
ENSSSCG00000021383	1.140010333	5.79E-09
ENSSSCG00000028571	1.13970414	1.32E-06
ENSSSCG00000016744	1.139110995	1.14E-06
ENSSSCG00000039160	1.138285751	2.37E-08
ENSSSCG00000016374	1.138043423	2.27E-07
ENSSSCG00000042572	1.137284107	3.84E-06
ENSSSCG00000010067	1.137186326	0.000760481
ENSSSCG00000032411	1.136971114	1.67E-06
ENSSSCG00000000683	1.135576429	1.11E-06
ENSSSCG00000051147	1.134811707	0.000136214
ENSSSCG00000011307	1.134591621	0.001275472
ENSSSCG00000033027	1.134189745	5.02E-07
ENSSSCG00000037066	1.133372732	0.001349935
ENSSSCG00000001510	1.133097845	4.13E-05
ENSSSCG00000032024	1.131901843	0.002723835
ENSSSCG00000032469	1.131675296	8.42E-06
ENSSSCG00000027660	1.131368252	4.98E-11
ENSSSCG00000035995	1.131056854	4.18E-09
ENSSSCG00000028814	1.130167976	0.000267062
ENSSSCG00000038543	1.129632139	7.80E-06
ENSSSCG00000037995	1.12930227	0.001562208
ENSSSCG00000045485	1.129291173	5.56E-11
ENSSSCG00000010589	1.129258164	3.43E-07
ENSSSCG00000023984	1.128954403	0.007672648
ENSSSCG00000023935	1.128884238	4.84E-07
ENSSSCG00000011065	1.128811561	0.001944337
ENSSSCG00000007470	1.128569621	9.59E-05
ENSSSCG00000003288	1.128534195	0.000334325
ENSSSCG00000003134	1.128082102	0.000198519
ENSSSCG00000007687	1.127315141	1.23E-08
ENSSSCG00000010540	1.127125771	6.04E-05
ENSSSCG00000045154	1.12669833	0.001360377
ENSSSCG00000007119	1.125787963	0.000327246
ENSSSCG00000013747	1.125090727	0.000438426
ENSSSCG00000013735	1.125072862	1.37E-06
ENSSSCG00000000745	1.125030673	3.35E-06
ENSSSCG00000017335	1.124485969	1.49E-10
ENSSSCG00000034732	1.12423	0.000917978
ENSSSCG00000027307	1.123557339	6.26E-05
ENSSSCG00000014031	1.122956125	0.001241391
ENSSSCG00000024756	1.122736416	2.22E-10
ENSSSCG00000036852	1.122108667	1.68E-08
ENSSSCG00000012893	1.121546065	1.45E-07
ENSSSCG00000011410	1.121211252	0.000414467
ENSSSCG00000029430	1.1206595	7.57E-06
ENSSSCG00000006487	1.119150789	4.63E-05
ENSSSCG00000023903	1.118439209	7.07E-08
ENSSSCG00000017806	1.118206521	4.14E-05
ENSSSCG00000048111	1.117954561	0.001861138

ENSSSCG00000010477	1.117471618	0.000353289
ENSSSCG00000006543	1.117469621	6.02E-09
ENSSSCG00000010161	1.117273345	5.27E-06
ENSSSCG00000032116	1.116425828	4.06E-07
ENSSSCG00000006902	1.115385538	1.58E-05
ENSSSCG00000015566	1.115325117	0.000119921
ENSSSCG00000005917	1.114435905	0.000497015
ENSSSCG00000000496	1.113674449	2.91E-05
ENSSSCG00000017364	1.113297278	1.87E-06
ENSSSCG00000016041	1.112979253	1.36E-08
ENSSSCG00000006166	1.112959299	1.07E-08
ENSSSCG00000037170	1.110790637	5.57E-05
ENSSSCG00000035820	1.110608256	4.10E-07
ENSSSCG00000017591	1.109712995	5.83E-05
ENSSSCG00000040089	1.109350729	1.19E-05
ENSSSCG00000012844	1.108739571	7.19E-05
ENSSSCG00000039769	1.108407492	4.33E-09
ENSSSCG00000003352	1.10833949	2.52E-07
ENSSSCG00000012890	1.106794529	2.39E-09
ENSSSCG00000028025	1.106704588	1.45E-05
ENSSSCG00000010756	1.106612839	6.80E-08
ENSSSCG00000009298	1.106539207	1.43E-05
ENSSSCG00000014040	1.105329354	3.53E-10
ENSSSCG00000007366	1.10433142	0.00093869
ENSSSCG00000032062	1.103691457	3.09E-06
ENSSSCG00000032067	1.10279238	8.87E-05
ENSSSCG00000006499	1.10267991	2.25E-09
ENSSSCG00000034927	1.102274573	0.000261934
ENSSSCG00000011562	1.10214729	8.46E-14
ENSSSCG00000003348	1.101988875	0.000200767
ENSSSCG00000039592	1.100603851	0.001347903
ENSSSCG00000009178	1.100229897	7.61E-05
ENSSSCG00000004666	1.099664373	1.31E-05
ENSSSCG00000029264	1.099443337	8.61E-05
ENSSSCG00000038914	1.099087837	2.93E-10
ENSSSCG00000051329	1.097951769	1.11E-07
ENSSSCG00000013896	1.097560495	2.22E-08
ENSSSCG00000000838	1.097484318	0.000574451
ENSSSCG00000044256	1.09743713	0.0015014
ENSSSCG00000017471	1.097227528	0.001163732
ENSSSCG00000009653	1.097140937	0.00128767
ENSSSCG00000047660	1.096897818	0.013055321
ENSSSCG00000039551	1.096738171	3.40E-05
ENSSSCG00000024126	1.096344582	0.000204376
ENSSSCG00000031630	1.095875171	9.81E-05
ENSSSCG00000021236	1.095654368	4.19E-12
ENSSSCG00000022322	1.095566965	3.34E-12
ENSSSCG00000004127	1.095382854	0.011994232
ENSSSCG00000023912	1.094993482	1.13E-06
ENSSSCG00000032364	1.094199196	0.000129969
ENSSSCG00000024316	1.093694113	1.05E-05
ENSSSCG00000017114	1.093650394	0.000127336
ENSSSCG00000023078	1.093135621	0.002891018
ENSSSCG00000017261	1.09292207	4.48E-05
ENSSSCG00000039881	1.092119059	1.23E-08
ENSSSCG00000034984	1.092118089	1.51E-07
ENSSSCG00000033860	1.092104624	9.22E-05
ENSSSCG00000031741	1.091849234	4.80E-05

ENSSSCG00000026446	1.091496809	0.000266867
ENSSSCG00000003393	1.091044865	0.000977812
ENSSSCG00000032833	1.091044736	0.001233849
ENSSSCG00000032710	1.090842085	0.001226273
ENSSSCG00000022757	1.089818192	0.002253201
ENSSSCG00000036213	1.08939415	2.26E-08
ENSSSCG00000002753	1.089171805	0.000120639
ENSSSCG00000006219	1.089143025	0.002080927
ENSSSCG00000015227	1.089138784	3.23E-07
ENSSSCG00000000694	1.088421888	9.75E-05
ENSSSCG00000023315	1.087254333	4.89E-08
ENSSSCG00000012485	1.08697167	0.004576178
ENSSSCG00000039148	1.08685364	5.94E-08
ENSSSCG00000002802	1.086603915	3.58E-09
ENSSSCG00000029005	1.086049581	1.94E-06
ENSSSCG00000033332	1.085715257	5.85E-05
ENSSSCG00000016372	1.085318039	2.64E-08
ENSSSCG00000024912	1.085316234	0.000102412
ENSSSCG00000010817	1.085046613	1.40E-07
ENSSSCG00000002020	1.084619466	0.000344106
ENSSSCG00000030577	1.084552379	1.35E-05
ENSSSCG00000010330	1.084232065	0.001088551
ENSSSCG00000017667	1.083203971	2.99E-05
ENSSSCG00000005704	1.082550232	0.001270772
ENSSSCG00000011385	1.081564763	0.005197998
ENSSSCG00000015769	1.081484771	8.23E-06
ENSSSCG00000015784	1.081324504	2.55E-07
ENSSSCG00000006029	1.080297304	0.000990976
ENSSSCG00000031388	1.079810502	0.000101966
ENSSSCG00000021472	1.079374236	1.78E-07
ENSSSCG00000001425	1.078266132	3.70E-06
ENSSSCG00000000021	1.078265531	0.007415803
ENSSSCG00000033653	1.076214446	0.00200303
ENSSSCG00000039416	1.075496524	1.41E-05
ENSSSCG00000013776	1.075376244	0.000128262
ENSSSCG00000001912	1.074766842	6.30E-07
ENSSSCG00000028282	1.074681792	6.52E-05
ENSSSCG00000001346	1.073941984	6.29E-08
ENSSSCG00000035037	1.073407898	0.001109283
ENSSSCG00000024245	1.072267528	0.000899276
ENSSSCG00000020906	1.071786053	0.009964881
ENSSSCG00000015604	1.071626565	0.000261698
ENSSSCG00000008383	1.071393713	1.94E-11
ENSSSCG00000030668	1.070897738	0.004445035
ENSSSCG00000032261	1.070158321	3.86E-07
ENSSSCG00000007554	1.068809866	8.68E-07
ENSSSCG00000003261	1.068437486	0.000548508
ENSSSCG00000015336	1.067939915	1.71E-05
ENSSSCG00000003701	1.067560783	0.002081576
ENSSSCG00000008675	1.067333931	6.76E-06
ENSSSCG00000013046	1.067312545	6.64E-08
ENSSSCG00000023323	1.066796097	7.81E-05
ENSSSCG00000027119	1.065859873	0.008372029
ENSSSCG00000006853	1.065840384	2.18E-06
ENSSSCG00000017264	1.064865112	2.07E-05
ENSSSCG00000036658	1.0647115	2.82E-06
ENSSSCG00000017118	1.063933166	0.000326646
ENSSSCG00000007947	1.063737333	0.000146107

ENSSSCG00000009373	1.062922798	0.009604733
ENSSSCG00000005965	1.062677894	0.005420711
ENSSSCG00000007076	1.062674713	0.000479889
ENSSSCG00000010060	1.061450233	0.011473474
ENSSSCG00000042774	1.060602375	0.001937698
ENSSSCG00000010240	1.060465781	0.003862808
ENSSSCG00000049674	1.060316343	0.002874274
ENSSSCG00000033177	1.060240891	1.06E-06
ENSSSCG00000005344	1.0598468	0.000517912
ENSSSCG00000000623	1.05973244	0.008514596
ENSSSCG00000029627	1.059263539	0.002775329
ENSSSCG00000047060	1.058791903	0.000846128
ENSSSCG00000011676	1.057914732	8.21E-07
ENSSSCG00000011113	1.057867678	0.001144471
ENSSSCG00000009084	1.057610392	7.34E-08
ENSSSCG00000032985	1.05740239	2.26E-06
ENSSSCG00000023482	1.057332854	0.000340838
ENSSSCG00000016379	1.057184875	1.81E-08
ENSSSCG00000015973	1.056967219	1.58E-05
ENSSSCG00000031789	1.055023583	0.00024284
ENSSSCG00000015110	1.05457184	7.75E-08
ENSSSCG00000003334	1.054496036	7.60E-08
ENSSSCG00000011797	1.054197508	0.00343682
ENSSSCG00000031669	1.054173084	5.40E-09
ENSSSCG00000038702	1.051536505	0.000231743
ENSSSCG00000013894	1.051492439	0.00050083
ENSSSCG00000015794	1.050780757	0.000493351
ENSSSCG00000009532	1.050554007	0.000165134
ENSSSCG00000026931	1.049524116	9.24E-05
ENSSSCG00000012978	1.048035341	0.001192139
ENSSSCG00000036064	1.047592991	4.44E-07
ENSSSCG00000004847	1.046911726	0.007049695
ENSSSCG00000031800	1.046589767	0.011625705
ENSSSCG00000033735	1.045642699	0.000548809
ENSSSCG00000002917	1.045614051	0.005965834
ENSSSCG00000011601	1.045602991	0.000271741
ENSSSCG00000008003	1.0447007	4.63E-07
ENSSSCG00000003331	1.044584304	0.004994917
ENSSSCG00000017420	1.04392989	0.000923623
ENSSSCG00000010029	1.043885311	5.26E-05
ENSSSCG00000017957	1.041856128	0.006846983
ENSSSCG00000033311	1.04089903	4.91E-05
ENSSSCG00000002307	1.040638473	0.000444645
ENSSSCG00000048507	1.04044859	0.0049628
ENSSSCG00000008035	1.040428627	0.000113998
ENSSSCG00000016998	1.04024626	0.01264475
ENSSSCG00000003189	1.040067444	0.004483127
ENSSSCG00000011578	1.039781335	0.007336235
ENSSSCG00000002854	1.039764053	8.89E-10
ENSSSCG00000009120	1.03941817	0.002210736
ENSSSCG00000039650	1.037498711	0.001499254
ENSSSCG00000015782	1.036732839	0.000232353
ENSSSCG00000017913	1.036645337	6.62E-07
ENSSSCG00000003943	1.036089467	4.98E-08
ENSSSCG00000005528	1.035281349	4.33E-08
ENSSSCG00000008682	1.035069028	1.45E-09
ENSSSCG00000027840	1.032756435	3.63E-09
ENSSSCG00000036178	1.029745493	4.80E-09

ENSSSCG00000021845	1.029098799	0.000563006
ENSSSCG00000015056	1.02814895	0.001905834
ENSSSCG00000007688	1.028078718	2.37E-06
ENSSSCG00000008304	1.027792778	7.84E-05
ENSSSCG00000039882	1.027549626	4.00E-12
ENSSSCG00000027257	1.025678349	3.86E-07
ENSSSCG00000003098	1.025634961	0.000228777
ENSSSCG00000008311	1.025426456	0.002289261
ENSSSCG00000026302	1.025407056	9.50E-09
ENSSSCG00000024990	1.025225213	6.40E-09
ENSSSCG00000037767	1.024547349	6.88E-06
ENSSSCG00000039224	1.024529402	7.20E-05
ENSSSCG00000006273	1.024458679	8.61E-05
ENSSSCG00000016827	1.024391077	0.002041511
ENSSSCG00000038440	1.023634075	0.00329387
ENSSSCG00000022364	1.023365742	1.24E-08
ENSSSCG00000014371	1.02295947	1.51E-07
ENSSSCG00000031413	1.022677034	5.90E-05
ENSSSCG00000038694	1.022161451	0.00730926
ENSSSCG00000013721	1.022015781	7.55E-10
ENSSSCG00000023176	1.021937753	2.37E-06
ENSSSCG00000045223	1.021913913	0.000529417
ENSSSCG00000026602	1.021749964	0.00010183
ENSSSCG00000009758	1.021674058	0.004205364
ENSSSCG00000038079	1.02159835	0.004929109
ENSSSCG00000003152	1.021221113	0.001968868
ENSSSCG00000006480	1.021181663	1.24E-05
ENSSSCG00000022404	1.021017848	0.000157998
ENSSSCG00000001701	1.020459239	0.003344525
ENSSSCG00000015730	1.020410115	0.001708079
ENSSSCG00000047338	1.020088157	0.008045742
ENSSSCG00000002719	1.019808684	0.000524114
ENSSSCG00000016226	1.019635124	0.007795003
ENSSSCG00000010742	1.019481731	0.000127359
ENSSSCG00000039896	1.018959632	5.35E-05
ENSSSCG00000011876	1.017920824	0.00098454
ENSSSCG00000029059	1.017035336	0.001440176
ENSSSCG00000010320	1.016106351	0.001709864
ENSSSCG00000013524	1.01576304	2.18E-05
ENSSSCG00000012280	1.014619561	0.000188316
ENSSSCG00000009246	1.014490316	0.000749673
ENSSSCG00000037734	1.014267659	2.23E-06
ENSSSCG00000003214	1.013604097	6.59E-05
ENSSSCG00000040325	1.013595483	2.64E-08
ENSSSCG00000000031	1.01187915	5.01E-07
ENSSSCG00000000158	1.010986216	0.00018719
ENSSSCG00000030888	1.009605605	1.99E-11
ENSSSCG00000003197	1.009460015	1.65E-07
ENSSSCG00000030255	1.008657203	0.000138011
ENSSSCG00000015108	1.008598694	4.87E-08
ENSSSCG00000017230	1.00817726	0.005764618
ENSSSCG00000022370	1.008067451	4.48E-05
ENSSSCG00000013048	1.007872959	2.08E-06
ENSSSCG00000040396	1.007808197	5.60E-10
ENSSSCG00000013304	1.007107425	0.007433134
ENSSSCG00000011258	1.00647395	2.70E-05
ENSSSCG00000032340	1.006266735	0.000119721
ENSSSCG00000017868	1.00618183	2.12E-06

ENSSSCG00000013633	1.006176574	0.001382759
ENSSSCG00000012378	1.005691815	9.49E-09
ENSSSCG00000037238	1.004913597	9.51E-05
ENSSSCG00000012534	1.004234608	5.99E-08
ENSSSCG00000034107	1.004127828	0.004702367
ENSSSCG00000011051	1.004077761	0.000543213
ENSSSCG00000011356	1.003041036	1.01E-06
ENSSSCG00000026264	1.003014537	0.005818524
ENSSSCG00000017104	1.002978198	0.001478879
ENSSSCG00000017140	1.002916076	0.000434338
ENSSSCG00000017197	1.002744593	2.28E-05
ENSSSCG00000008016	1.002418803	3.74E-06
ENSSSCG00000007543	1.002322132	0.000144797
ENSSSCG00000021834	1.002011261	0.00069111
ENSSSCG00000025588	1.001557432	0.000490299
ENSSSCG00000039007	3.08E-11	6.91E-148
ENSSSCG00000011699	-1.000089671	1.36E-06
ENSSSCG00000022953	-1.000102496	0.006822199
ENSSSCG00000033383	-1.000150988	0.005236996
ENSSSCG00000017546	-1.000225035	0.002350979
ENSSSCG00000021656	-1.000309798	0.003522837
ENSSSCG00000051266	-1.000393857	0.000344651
ENSSSCG00000027466	-1.001176627	0.000176478
ENSSSCG00000006810	-1.001427221	0.008011862
ENSSSCG00000001727	-1.001472659	0.000186842
ENSSSCG00000012789	-1.001693497	8.57E-05
ENSSSCG00000015407	-1.002631024	0.006735401
ENSSSCG00000006303	-1.002862344	9.95E-07
ENSSSCG00000006083	-1.003022477	5.22E-10
ENSSSCG00000014184	-1.003023891	5.53E-05
ENSSSCG00000011296	-1.003476794	0.001444003
ENSSSCG00000004598	-1.003636762	0.000845339
ENSSSCG00000017873	-1.003765183	0.0001731
ENSSSCG00000036766	-1.003886912	0.000878992
ENSSSCG00000012408	-1.005859163	0.009957858
ENSSSCG00000008617	-1.006829284	0.004071911
ENSSSCG00000003708	-1.007014473	1.41E-05
ENSSSCG00000039245	-1.007133355	0.003359446
ENSSSCG00000005166	-1.007558982	0.00369642
ENSSSCG00000014110	-1.008499811	0.008459313
ENSSSCG00000034739	-1.009099735	0.004628753
ENSSSCG00000016444	-1.009175594	2.67E-06
ENSSSCG00000031348	-1.010862051	2.79E-06
ENSSSCG00000012591	-1.010899437	0.00705397
ENSSSCG00000010621	-1.011018505	0.000184349
ENSSSCG00000016022	-1.011493233	0.011055614
ENSSSCG00000049587	-1.011525528	0.000715772
ENSSSCG00000025238	-1.011618266	0.00071928
ENSSSCG00000006381	-1.01211777	0.00075832
ENSSSCG00000010281	-1.013199775	0.00408867
ENSSSCG00000015297	-1.013418354	0.001084947
ENSSSCG00000012634	-1.013505121	3.79E-07
ENSSSCG00000030585	-1.013835097	0.002219994
ENSSSCG00000036877	-1.014033302	1.28E-06
ENSSSCG00000008169	-1.014316186	4.60E-06
ENSSSCG00000004347	-1.01438234	7.48E-09
ENSSSCG00000011076	-1.014626463	1.46E-05
ENSSSCG00000027434	-1.015107544	0.001668821

ENSSSCG00000009408	-1.015488294	1.56E-10
ENSSSCG00000050739	-1.015762091	0.00146615
ENSSSCG00000038009	-1.016072861	9.20E-05
ENSSSCG00000006383	-1.016632807	0.006609959
ENSSSCG00000004752	-1.017078888	0.013167416
ENSSSCG00000009053	-1.017535252	0.000192164
ENSSSCG00000005190	-1.017791826	5.48E-07
ENSSSCG00000035911	-1.01946699	0.00072595
ENSSSCG00000006338	-1.019522154	0.002500495
ENSSSCG00000023351	-1.021155247	7.93E-06
ENSSSCG00000046685	-1.021287708	0.008557593
ENSSSCG00000009326	-1.021408795	9.29E-06
ENSSSCG00000044716	-1.02150993	0.006970412
ENSSSCG00000031429	-1.021757527	0.000921129
ENSSSCG00000009237	-1.021771931	1.14E-06
ENSSSCG00000009929	-1.022388258	0.003699542
ENSSSCG00000011811	-1.022553056	1.17E-05
ENSSSCG00000029805	-1.02285075	0.000174856
ENSSSCG00000006901	-1.022982639	1.28E-07
ENSSSCG00000001921	-1.023396358	7.58E-06
ENSSSCG00000031175	-1.023474582	0.000614121
ENSSSCG00000026699	-1.023957027	9.96E-06
ENSSSCG00000005701	-1.024005372	0.000118027
ENSSSCG00000046097	-1.024483909	0.000100328
ENSSSCG00000006862	-1.024525206	8.69E-07
ENSSSCG00000038693	-1.024732171	1.06E-05
ENSSSCG00000005041	-1.024785763	3.92E-06
ENSSSCG00000002829	-1.026406938	3.51E-05
ENSSSCG00000040946	-1.026869559	4.57E-06
ENSSSCG00000041490	-1.027380026	7.34E-05
ENSSSCG00000023243	-1.027597081	7.21E-06
ENSSSCG00000024591	-1.027779227	4.22E-05
ENSSSCG00000008844	-1.028174278	0.002515208
ENSSSCG00000016756	-1.029294333	5.78E-06
ENSSSCG00000021731	-1.029302535	6.93E-06
ENSSSCG00000038730	-1.029480402	2.25E-05
ENSSSCG00000047250	-1.029570643	0.004164671
ENSSSCG00000028239	-1.030321111	1.16E-06
ENSSSCG00000011896	-1.030376409	0.00566107
ENSSSCG00000014838	-1.030481541	1.47E-07
ENSSSCG00000016141	-1.031472992	2.32E-06
ENSSSCG00000016554	-1.033292667	7.79E-10
ENSSSCG00000026571	-1.036157789	4.56E-07
ENSSSCG00000011892	-1.036614787	0.000999179
ENSSSCG00000023235	-1.036894451	0.0002407
ENSSSCG00000010494	-1.036941808	0.002127204
ENSSSCG00000017990	-1.038221727	0.000374035
ENSSSCG00000028662	-1.039128155	7.74E-05
ENSSSCG00000002254	-1.039314157	3.16E-05
ENSSSCG00000009818	-1.039746006	0.000360597
ENSSSCG00000004822	-1.039916715	0.004255243
ENSSSCG00000038180	-1.040018006	3.06E-05
ENSSSCG00000003731	-1.040220475	2.45E-07
ENSSSCG00000022506	-1.041373487	0.000125731
ENSSSCG00000024396	-1.042298076	3.28E-09
ENSSSCG00000029507	-1.042524865	6.83E-05
ENSSSCG00000035849	-1.043318435	0.005760232
ENSSSCG00000046168	-1.043704936	0.000114228

ENSSSCG00000012001	-1.045282461	2.07E-08
ENSSSCG00000001966	-1.045751604	2.68E-05
ENSSSCG00000007814	-1.046047809	4.96E-06
ENSSSCG00000031592	-1.046146305	0.003510096
ENSSSCG00000015770	-1.046219293	7.38E-07
ENSSSCG00000022126	-1.046597833	3.01E-09
ENSSSCG00000013409	-1.046667693	8.15E-08
ENSSSCG00000037562	-1.047275341	0.003164451
ENSSSCG00000037762	-1.04730533	9.22E-07
ENSSSCG00000024043	-1.047566908	9.90E-06
ENSSSCG00000006781	-1.047826008	0.003053891
ENSSSCG00000012253	-1.048164344	1.03E-05
ENSSSCG00000013865	-1.048990311	0.001153192
ENSSSCG00000042910	-1.049021817	2.73E-07
ENSSSCG00000006987	-1.049096563	0.009651714
ENSSSCG00000001063	-1.049193551	1.53E-09
ENSSSCG00000043604	-1.04957697	5.54E-05
ENSSSCG00000039054	-1.05050566	1.02E-06
ENSSSCG00000035675	-1.050980328	0.0072522
ENSSSCG00000012618	-1.051775074	0.006575049
ENSSSCG00000016074	-1.052069036	2.69E-05
ENSSSCG00000040188	-1.053063387	0.000470821
ENSSSCG00000039442	-1.053540994	0.001700399
ENSSSCG00000010640	-1.054418538	4.40E-07
ENSSSCG00000004850	-1.055918176	0.00021055
ENSSSCG00000022903	-1.056443446	4.11E-08
ENSSSCG00000033260	-1.056532863	0.007830307
ENSSSCG00000032637	-1.057363246	0.005892653
ENSSSCG00000011959	-1.059270526	5.96E-10
ENSSSCG00000028052	-1.060213609	0.000664309
ENSSSCG00000013113	-1.060615553	4.47E-05
ENSSSCG00000011074	-1.060798499	1.79E-06
ENSSSCG00000016956	-1.060817479	2.89E-06
ENSSSCG00000020878	-1.062950359	2.41E-07
ENSSSCG00000045831	-1.064614459	0.000329876
ENSSSCG00000013241	-1.065095702	4.29E-05
ENSSSCG00000013174	-1.065467329	0.000616789
ENSSSCG00000008866	-1.065740307	0.001412611
ENSSSCG00000013313	-1.067190668	2.28E-05
ENSSSCG00000025406	-1.067255572	1.30E-08
ENSSSCG00000007542	-1.067472287	2.31E-05
ENSSSCG00000027975	-1.067641217	0.001938732
ENSSSCG00000029687	-1.068393991	0.001980458
ENSSSCG00000035557	-1.070003454	0.000523598
ENSSSCG00000021638	-1.070297392	0.000531576
ENSSSCG00000002752	-1.071116287	4.13E-05
ENSSSCG00000002276	-1.07117759	3.32E-05
ENSSSCG00000014321	-1.071207246	0.00137444
ENSSSCG00000006709	-1.071373882	7.79E-05
ENSSSCG00000023709	-1.071846997	0.0001723
ENSSSCG00000025130	-1.072206233	2.84E-07
ENSSSCG00000004441	-1.072895467	4.84E-11
ENSSSCG00000011023	-1.073120388	4.78E-06
ENSSSCG00000006547	-1.073301373	0.00077238
ENSSSCG00000003651	-1.073610911	0.000186786
ENSSSCG00000012434	-1.073845437	3.61E-07
ENSSSCG00000035196	-1.075201409	1.52E-08
ENSSSCG00000011745	-1.075303281	1.22E-06

ENSSSCG00000009676	-1.07594461	6.57E-13
ENSSSCG00000015244	-1.076346203	3.48E-08
ENSSSCG00000012141	-1.076593984	0.000879943
ENSSSCG00000005828	-1.077160429	0.000178423
ENSSSCG00000006872	-1.077293747	3.93E-07
ENSSSCG00000022618	-1.077661353	3.85E-06
ENSSSCG00000001011	-1.077678742	8.24E-06
ENSSSCG00000040109	-1.078087837	1.10E-06
ENSSSCG00000004156	-1.078552985	3.00E-06
ENSSSCG00000015866	-1.078962632	2.80E-06
ENSSSCG00000028148	-1.079169772	0.01044172
ENSSSCG00000032071	-1.079620822	6.09E-06
ENSSSCG00000039203	-1.080429747	0.003466801
ENSSSCG00000033763	-1.081266028	6.62E-06
ENSSSCG00000003113	-1.081337656	5.27E-07
ENSSSCG00000002863	-1.081423474	0.003457511
ENSSSCG00000038745	-1.081559976	0.000737025
ENSSSCG00000021654	-1.081818431	0.001314247
ENSSSCG00000049170	-1.083237309	0.009700485
ENSSSCG00000005343	-1.083388993	5.53E-08
ENSSSCG00000034838	-1.083474441	0.005368907
ENSSSCG00000031856	-1.083915644	2.71E-05
ENSSSCG00000029860	-1.084252127	2.33E-05
ENSSSCG00000045622	-1.085268621	0.004246704
ENSSSCG00000035757	-1.086621453	1.33E-06
ENSSSCG00000012416	-1.08748256	1.44E-05
ENSSSCG00000022173	-1.087612383	0.001423226
ENSSSCG00000005607	-1.087969144	2.20E-05
ENSSSCG00000015878	-1.088746878	9.06E-05
ENSSSCG00000034036	-1.088801589	0.001919679
ENSSSCG00000008185	-1.08954465	0.009769921
ENSSSCG00000003139	-1.09006402	0.000163543
ENSSSCG00000015136	-1.092703188	0.000372797
ENSSSCG00000016052	-1.09388432	2.93E-06
ENSSSCG00000022333	-1.094653036	0.001633095
ENSSSCG00000022345	-1.094835329	3.71E-05
ENSSSCG00000017752	-1.095290289	4.78E-13
ENSSSCG00000023665	-1.095438688	0.000262113
ENSSSCG00000033059	-1.096266248	0.008754811
ENSSSCG00000036756	-1.096312013	2.13E-06
ENSSSCG00000012508	-1.096536772	5.14E-06
ENSSSCG00000001458	-1.098165056	2.84E-07
ENSSSCG00000051615	-1.098613083	0.004344592
ENSSSCG00000023992	-1.099351844	8.20E-05
ENSSSCG00000010544	-1.099772838	2.30E-06
ENSSSCG00000045426	-1.10219728	0.0004112
ENSSSCG00000011765	-1.102550379	0.001230796
ENSSSCG00000000625	-1.102707557	9.75E-08
ENSSSCG00000011234	-1.103287879	8.49E-05
ENSSSCG00000016119	-1.1036902	4.56E-09
ENSSSCG00000040013	-1.103703155	0.001277451
ENSSSCG00000017367	-1.104299611	0.002711483
ENSSSCG00000008147	-1.105017423	0.002209781
ENSSSCG00000006453	-1.105079594	0.000326468
ENSSSCG00000041715	-1.107339884	7.22E-06
ENSSSCG00000004479	-1.107754051	0.006312011
ENSSSCG00000011686	-1.108688742	0.000359401
ENSSSCG00000007700	-1.110451332	0.000377334

ENSSSCG00000004478	-1.110508338	3.05E-08
ENSSSCG00000021359	-1.11089903	0.000559343
ENSSSCG00000003768	-1.111119855	0.010171378
ENSSSCG00000039194	-1.11131435	0.003185505
ENSSSCG00000010046	-1.112061161	0.000212773
ENSSSCG00000011322	-1.114351635	0.007065225
ENSSSCG00000023434	-1.114654851	8.07E-08
ENSSSCG00000048374	-1.115006615	0.000206436
ENSSSCG00000002428	-1.115080952	6.83E-05
ENSSSCG00000003825	-1.115745358	1.81E-10
ENSSSCG00000033880	-1.116191452	0.000108961
ENSSSCG00000012350	-1.116740912	0.000644973
ENSSSCG00000011916	-1.116965424	2.46E-06
ENSSSCG00000033385	-1.117130119	8.95E-07
ENSSSCG00000048558	-1.117134703	0.001991089
ENSSSCG00000021068	-1.117241667	4.06E-10
ENSSSCG00000021946	-1.118712814	1.35E-05
ENSSSCG00000003148	-1.118756962	0.002718607
ENSSSCG00000025087	-1.118789853	1.25E-05
ENSSSCG00000007155	-1.119179565	2.40E-05
ENSSSCG00000017095	-1.119332404	1.17E-06
ENSSSCG00000006287	-1.11940869	0.006443456
ENSSSCG00000023376	-1.119543748	0.000134503
ENSSSCG00000046072	-1.119784846	0.012608352
ENSSSCG00000039393	-1.120837249	8.28E-07
ENSSSCG00000040465	-1.121253765	0.010698052
ENSSSCG00000016574	-1.121600287	0.003235276
ENSSSCG00000012741	-1.121722113	0.003716221
ENSSSCG00000021862	-1.122046977	2.95E-08
ENSSSCG00000003486	-1.123076519	0.003100826
ENSSSCG00000013073	-1.123215752	3.58E-07
ENSSSCG00000007116	-1.123268594	0.0025514
ENSSSCG00000029331	-1.123478111	2.01E-07
ENSSSCG00000027278	-1.123492232	8.68E-06
ENSSSCG00000013295	-1.123670171	1.16E-09
ENSSSCG00000004209	-1.124102905	9.63E-09
ENSSSCG00000038469	-1.124126792	1.17E-05
ENSSSCG00000042607	-1.124358276	0.003580925
ENSSSCG00000006306	-1.124442353	8.89E-10
ENSSSCG00000008980	-1.124704259	7.09E-05
ENSSSCG00000015131	-1.125022577	0.000489995
ENSSSCG00000040556	-1.125059235	0.000836142
ENSSSCG00000015413	-1.125110297	0.004231648
ENSSSCG00000016843	-1.126416057	0.000570853
ENSSSCG00000010814	-1.126463497	0.000223754
ENSSSCG00000015328	-1.126566594	0.005432023
ENSSSCG00000011791	-1.127061234	0.000139937
ENSSSCG00000000559	-1.127879702	1.25E-05
ENSSSCG00000007949	-1.129699179	0.002947742
ENSSSCG00000002051	-1.130183624	5.05E-06
ENSSSCG00000009627	-1.130916511	2.18E-07
ENSSSCG00000024048	-1.131139155	0.00073984
ENSSSCG00000012137	-1.131166059	0.000240203
ENSSSCG00000037257	-1.131439045	0.000272478
ENSSSCG00000039161	-1.131737555	2.75E-06
ENSSSCG00000038269	-1.132734468	0.002074921
ENSSSCG00000038556	-1.133254104	0.01119244
ENSSSCG00000002932	-1.133402025	0.000107074

ENSSSCG00000031356	-1.13353464	5.58E-07
ENSSSCG00000037970	-1.134192428	0.012863786
ENSSSCG00000014833	-1.135129828	1.37E-07
ENSSSCG00000000528	-1.135383518	9.22E-06
ENSSSCG00000003603	-1.135588738	0.005771979
ENSSSCG00000026383	-1.1370491	5.19E-07
ENSSSCG00000037406	-1.138529699	0.001104639
ENSSSCG00000049048	-1.13886407	0.009275017
ENSSSCG00000016083	-1.13906507	0.002274374
ENSSSCG00000016164	-1.139116058	4.97E-07
ENSSSCG00000017624	-1.139629103	0.003795789
ENSSSCG00000011681	-1.140209167	0.000324829
ENSSSCG00000011576	-1.140541917	0.00065393
ENSSSCG00000022168	-1.143097115	1.37E-10
ENSSSCG00000031713	-1.144406892	0.000786088
ENSSSCG00000007478	-1.146025094	0.00478635
ENSSSCG00000011592	-1.146064498	0.002181392
ENSSSCG00000010853	-1.147473734	0.005583304
ENSSSCG00000007094	-1.147838142	2.41E-05
ENSSSCG00000014091	-1.148243384	0.00048075
ENSSSCG00000008642	-1.148289684	1.72E-10
ENSSSCG00000026583	-1.149772585	7.77E-11
ENSSSCG00000034727	-1.150710522	1.31E-05
ENSSSCG00000010239	-1.151533092	1.16E-09
ENSSSCG00000021255	-1.152099382	0.000359338
ENSSSCG00000016557	-1.153036538	0.000520764
ENSSSCG00000015403	-1.153150002	0.000399969
ENSSSCG00000002764	-1.153164052	0.013018514
ENSSSCG00000006912	-1.153289385	1.60E-06
ENSSSCG00000006642	-1.153655346	0.000256092
ENSSSCG00000014161	-1.154668034	3.84E-05
ENSSSCG00000008475	-1.155221052	6.39E-05
ENSSSCG00000026004	-1.155371773	0.000579725
ENSSSCG00000001782	-1.155405539	6.24E-05
ENSSSCG00000020984	-1.155739116	4.81E-06
ENSSSCG00000007565	-1.156164956	0.000285651
ENSSSCG00000005030	-1.156711937	5.85E-10
ENSSSCG00000016031	-1.158816483	4.58E-05
ENSSSCG00000022318	-1.15967507	3.88E-07
ENSSSCG00000006001	-1.160288385	6.85E-08
ENSSSCG00000036822	-1.160855472	0.001649655
ENSSSCG00000051710	-1.160883158	0.000977975
ENSSSCG00000005180	-1.160922358	1.81E-07
ENSSSCG00000028359	-1.161338887	0.001684482
ENSSSCG00000028974	-1.161344477	4.02E-07
ENSSSCG00000005382	-1.161800144	8.32E-07
ENSSSCG00000035887	-1.16191439	1.28E-05
ENSSSCG00000044960	-1.162539666	0.007806744
ENSSSCG00000021606	-1.163486525	2.44E-05
ENSSSCG00000035421	-1.163624398	0.005093111
ENSSSCG00000050974	-1.163895709	0.000143123
ENSSSCG00000023028	-1.166305422	0.000456012
ENSSSCG00000023001	-1.166976567	0.000126794
ENSSSCG00000049122	-1.16797119	0.002055543
ENSSSCG00000021490	-1.167989711	0.004615084
ENSSSCG00000011925	-1.169789669	6.30E-07
ENSSSCG00000016317	-1.170493876	1.47E-05
ENSSSCG00000042379	-1.172012992	0.008549117

ENSSSCG00000002039	-1.172859151	1.68E-13
ENSSSCG00000031337	-1.172895796	1.03E-09
ENSSSCG00000044437	-1.173014627	0.005599851
ENSSSCG00000051491	-1.173347664	0.010383772
ENSSSCG00000006039	-1.173764641	1.03E-07
ENSSSCG00000005609	-1.173772717	4.33E-06
ENSSSCG00000010485	-1.175076898	0.000162128
ENSSSCG00000010757	-1.175095035	8.99E-08
ENSSSCG00000013393	-1.175322125	0.001235204
ENSSSCG00000045593	-1.176373639	2.56E-05
ENSSSCG00000045836	-1.176453013	0.000175587
ENSSSCG00000046723	-1.176742591	2.31E-05
ENSSSCG00000022230	-1.177545723	5.69E-09
ENSSSCG00000032741	-1.177676746	7.18E-06
ENSSSCG00000013106	-1.178938737	0.000437922
ENSSSCG00000000735	-1.180122468	5.38E-05
ENSSSCG00000025876	-1.180274488	7.77E-07
ENSSSCG00000009060	-1.180468473	0.000852229
ENSSSCG00000005641	-1.180718576	4.10E-06
ENSSSCG00000017376	-1.181117964	3.03E-08
ENSSSCG00000039368	-1.182561739	0.000107671
ENSSSCG00000009832	-1.182800625	0.002082469
ENSSSCG00000023957	-1.183620621	0.001222021
ENSSSCG00000035195	-1.184362587	0.000385624
ENSSSCG00000028108	-1.184993036	0.001273579
ENSSSCG00000032196	-1.185060786	5.65E-14
ENSSSCG00000008604	-1.185795626	2.46E-09
ENSSSCG00000026196	-1.185859561	1.18E-06
ENSSSCG00000005235	-1.188774617	0.000632808
ENSSSCG00000002507	-1.188855962	0.000469519
ENSSSCG00000037019	-1.18894022	0.002742662
ENSSSCG00000011485	-1.190744854	2.45E-10
ENSSSCG00000008051	-1.190799959	3.64E-13
ENSSSCG00000001718	-1.191653686	7.24E-05
ENSSSCG00000009968	-1.192673459	0.00042216
ENSSSCG00000005981	-1.193222664	0.001284015
ENSSSCG00000030597	-1.193227654	0.000698698
ENSSSCG00000004205	-1.193540243	8.61E-08
ENSSSCG00000015368	-1.193859852	9.90E-06
ENSSSCG00000027230	-1.193995431	2.90E-05
ENSSSCG00000021343	-1.195019347	3.69E-08
ENSSSCG00000011885	-1.195218958	5.62E-08
ENSSSCG00000001572	-1.196200774	7.03E-08
ENSSSCG00000034765	-1.196535663	2.75E-05
ENSSSCG00000037557	-1.196625525	1.97E-05
ENSSSCG00000021515	-1.196698799	0.007002614
ENSSSCG00000011663	-1.196764296	0.004330035
ENSSSCG00000003090	-1.197776828	1.32E-09
ENSSSCG00000002341	-1.1978486	0.000865711
ENSSSCG00000008981	-1.200224412	0.000215896
ENSSSCG00000034348	-1.200878824	0.000969947
ENSSSCG00000023862	-1.200887797	0.000316607
ENSSSCG00000009245	-1.201207361	0.000941954
ENSSSCG00000032674	-1.201655273	5.55E-05
ENSSSCG00000017389	-1.204750734	1.04E-06
ENSSSCG00000047084	-1.206366067	0.002654033
ENSSSCG00000033949	-1.206498248	3.59E-05
ENSSSCG00000015795	-1.206768454	0.012485823

ENSSSCG00000039426	-1.207071761	6.33E-09
ENSSSCG00000009833	-1.207221457	2.43E-08
ENSSSCG00000036139	-1.208758919	0.002207038
ENSSSCG00000032522	-1.209064394	3.14E-05
ENSSSCG00000001242	-1.209176395	6.78E-08
ENSSSCG00000003806	-1.209862423	1.92E-08
ENSSSCG00000002424	-1.209896744	7.83E-05
ENSSSCG00000002919	-1.211872868	0.000108711
ENSSSCG00000050000	-1.212565145	0.001493258
ENSSSCG00000024108	-1.213712453	9.06E-07
ENSSSCG00000016831	-1.213781788	0.000983382
ENSSSCG00000032984	-1.214127324	2.82E-11
ENSSSCG00000005751	-1.214362692	0.001024662
ENSSSCG00000034768	-1.215165073	1.00E-08
ENSSSCG00000010204	-1.215408389	5.25E-07
ENSSSCG00000010400	-1.215635524	3.88E-06
ENSSSCG00000028699	-1.215844774	0.000235509
ENSSSCG00000022429	-1.216645465	0.000598206
ENSSSCG00000017282	-1.216656893	5.04E-06
ENSSSCG00000026592	-1.217853703	1.56E-08
ENSSSCG00000001499	-1.217952823	0.002045617
ENSSSCG00000023195	-1.218105834	0.000818175
ENSSSCG00000008991	-1.218511199	6.41E-05
ENSSSCG00000035828	-1.218760091	0.006208234
ENSSSCG00000005423	-1.219193612	1.05E-13
ENSSSCG00000031794	-1.220411164	8.23E-05
ENSSSCG00000014121	-1.221048157	5.27E-05
ENSSSCG00000036553	-1.221917426	0.000498034
ENSSSCG00000025049	-1.221981655	4.52E-05
ENSSSCG00000000884	-1.222263174	3.54E-07
ENSSSCG00000038374	-1.222605005	3.36E-10
ENSSSCG00000015144	-1.222921198	0.001937801
ENSSSCG00000010277	-1.223445092	1.17E-06
ENSSSCG00000001755	-1.224054288	4.58E-05
ENSSSCG00000035511	-1.224168894	4.59E-08
ENSSSCG00000010322	-1.224464327	3.63E-05
ENSSSCG00000050436	-1.224737133	6.10E-05
ENSSSCG00000049524	-1.225049197	4.37E-06
ENSSSCG00000002041	-1.225314498	3.35E-07
ENSSSCG00000039287	-1.22549914	2.20E-09
ENSSSCG00000025686	-1.226763204	7.99E-12
ENSSSCG00000020858	-1.22946812	1.15E-05
ENSSSCG00000006289	-1.229903119	7.36E-06
ENSSSCG00000004628	-1.232170233	2.54E-05
ENSSSCG00000004980	-1.232426132	1.35E-06
ENSSSCG00000004390	-1.232690226	2.74E-08
ENSSSCG00000040706	-1.233209117	0.001366106
ENSSSCG00000013311	-1.234452596	0.008493397
ENSSSCG00000007056	-1.234506285	0.005896503
ENSSSCG00000036549	-1.234788705	1.80E-08
ENSSSCG00000009231	-1.234950223	0.009154343
ENSSSCG00000000874	-1.236344814	0.001751688
ENSSSCG00000012156	-1.236455597	5.94E-08
ENSSSCG00000008970	-1.236566427	0.00077187
ENSSSCG00000028529	-1.238074742	1.83E-08
ENSSSCG00000009747	-1.239487274	2.76E-05
ENSSSCG00000016898	-1.240265859	5.23E-06
ENSSSCG00000039745	-1.241669482	0.003734361

ENSSSCG00000004275	-1.242361291	5.60E-08
ENSSSCG00000008832	-1.242392696	3.58E-06
ENSSSCG00000015691	-1.243849913	0.001036299
ENSSSCG00000006369	-1.244265994	5.08E-05
ENSSSCG00000005503	-1.244441724	1.67E-06
ENSSSCG00000041370	-1.24505351	2.34E-06
ENSSSCG00000008737	-1.24592049	0.008326105
ENSSSCG00000039279	-1.246648244	0.00657668
ENSSSCG00000021562	-1.247195128	8.94E-08
ENSSSCG00000048686	-1.248560541	0.005139725
ENSSSCG00000002520	-1.248916377	2.56E-07
ENSSSCG00000027777	-1.249120854	0.000199328
ENSSSCG00000009403	-1.250215233	1.54E-06
ENSSSCG00000008456	-1.250970152	5.19E-06
ENSSSCG00000029186	-1.251951266	0.008413322
ENSSSCG00000016925	-1.25297818	0.000246456
ENSSSCG00000050719	-1.253713851	0.00748467
ENSSSCG00000003410	-1.254040709	6.69E-18
ENSSSCG00000009379	-1.255615224	0.001828159
ENSSSCG00000018058	-1.255968801	1.21E-05
ENSSSCG00000005338	-1.257662434	0.00016728
ENSSSCG00000038918	-1.258592601	5.26E-06
ENSSSCG00000016174	-1.258686722	5.25E-08
ENSSSCG00000013720	-1.259998993	4.16E-07
ENSSSCG00000015256	-1.260423775	3.99E-09
ENSSSCG00000014232	-1.261473863	2.00E-08
ENSSSCG00000015879	-1.262598836	0.004463348
ENSSSCG00000036223	-1.265593484	0.000454661
ENSSSCG00000017159	-1.266249523	0.000242419
ENSSSCG00000015901	-1.266880894	0.010558518
ENSSSCG00000036446	-1.267045284	0.000759016
ENSSSCG00000004875	-1.267511643	1.99E-12
ENSSSCG00000003147	-1.268571639	0.006142772
ENSSSCG00000035983	-1.269611725	0.000168343
ENSSSCG00000027093	-1.27009033	0.000161533
ENSSSCG00000029715	-1.270965246	0.000485263
ENSSSCG00000036360	-1.272262634	5.80E-08
ENSSSCG00000031905	-1.272387199	0.008528646
ENSSSCG00000016703	-1.272946538	9.20E-05
ENSSSCG00000006452	-1.274551244	1.69E-08
ENSSSCG00000011014	-1.275432485	0.000112798
ENSSSCG00000012759	-1.275751672	0.007248077
ENSSSCG00000013018	-1.276135682	1.24E-05
ENSSSCG00000005375	-1.277960857	0.004031078
ENSSSCG00000043173	-1.279666275	0.011087873
ENSSSCG00000001025	-1.279694567	4.15E-10
ENSSSCG00000017617	-1.280915889	8.20E-09
ENSSSCG00000004586	-1.280930577	0.002495013
ENSSSCG00000003145	-1.280991127	0.002289757
ENSSSCG00000022993	-1.281614076	7.76E-05
ENSSSCG00000009233	-1.28315009	0.000124662
ENSSSCG00000027467	-1.283376711	0.000841079
ENSSSCG00000004601	-1.283379613	0.006439104
ENSSSCG00000042618	-1.284246398	0.001145192
ENSSSCG00000017265	-1.284266277	4.75E-07
ENSSSCG00000030065	-1.285078526	3.35E-06
ENSSSCG00000006878	-1.28824656	3.09E-07
ENSSSCG00000000681	-1.288301029	4.74E-08

ENSSSCG00000020701	-1.288777493	3.83E-07
ENSSSCG00000004149	-1.288833565	3.33E-05
ENSSSCG00000033010	-1.288962871	0.007406971
ENSSSCG00000021053	-1.289624354	0.008941573
ENSSSCG00000038993	-1.290693566	1.43E-05
ENSSSCG00000010962	-1.292297809	0.003241866
ENSSSCG00000011579	-1.292320842	0.00019412
ENSSSCG00000030560	-1.293158508	2.99E-06
ENSSSCG00000014187	-1.293548411	0.000437566
ENSSSCG00000009338	-1.294365608	1.06E-05
ENSSSCG00000035147	-1.294628493	2.16E-08
ENSSSCG00000025243	-1.295050013	0.007392352
ENSSSCG00000036022	-1.295377878	1.14E-06
ENSSSCG00000010276	-1.295491297	4.17E-06
ENSSSCG00000016850	-1.295690067	3.46E-10
ENSSSCG00000009106	-1.296098937	2.81E-09
ENSSSCG00000016841	-1.297121661	0.000847602
ENSSSCG00000038811	-1.297271197	3.15E-06
ENSSSCG00000022635	-1.297580274	0.000272926
ENSSSCG00000001698	-1.297821096	0.007935512
ENSSSCG00000027198	-1.298408285	5.65E-06
ENSSSCG00000004570	-1.299802743	5.26E-05
ENSSSCG00000002026	-1.300234182	3.30E-05
ENSSSCG00000005498	-1.301680736	0.000257921
ENSSSCG00000001403	-1.302907182	3.11E-05
ENSSSCG00000041189	-1.302915885	0.000322035
ENSSSCG00000026753	-1.303068128	1.20E-10
ENSSSCG00000016754	-1.303317983	0.000812027
ENSSSCG00000048329	-1.303798348	0.002554259
ENSSSCG00000016672	-1.305101514	0.000568025
ENSSSCG00000034178	-1.305135917	1.19E-06
ENSSSCG00000006213	-1.306340101	0.000607718
ENSSSCG00000004207	-1.306528415	8.38E-09
ENSSSCG00000000602	-1.306632729	2.42E-05
ENSSSCG00000010862	-1.307686876	4.74E-19
ENSSSCG00000015398	-1.308896563	5.13E-12
ENSSSCG00000004165	-1.308946882	2.91E-08
ENSSSCG00000022592	-1.309556223	0.011181234
ENSSSCG00000044492	-1.309775036	0.00773237
ENSSSCG00000029558	-1.309818812	0.009027767
ENSSSCG00000026517	-1.310401495	0.000325953
ENSSSCG00000022705	-1.310668425	1.48E-07
ENSSSCG00000026861	-1.311495538	9.06E-06
ENSSSCG00000010768	-1.312661259	0.010771672
ENSSSCG00000011168	-1.313873006	0.005253964
ENSSSCG00000011169	-1.313993338	3.54E-06
ENSSSCG00000046811	-1.31494647	0.001403991
ENSSSCG00000034056	-1.315372324	0.006416526
ENSSSCG00000036314	-1.315702207	7.54E-10
ENSSSCG00000009115	-1.316548343	4.69E-05
ENSSSCG00000037890	-1.316581433	0.000699554
ENSSSCG00000011416	-1.316875481	0.000497388
ENSSSCG00000006458	-1.318860423	1.37E-08
ENSSSCG00000010196	-1.319432156	5.40E-07
ENSSSCG00000045496	-1.320009771	8.52E-06
ENSSSCG00000004607	-1.32100021	1.55E-05
ENSSSCG00000016332	-1.321078821	2.06E-16
ENSSSCG00000048537	-1.321281643	0.008946728

ENSSSCG00000039706	-1.32173426	1.19E-06
ENSSSCG00000034630	-1.321931002	1.81E-07
ENSSSCG00000031589	-1.322245965	0.005775231
ENSSSCG00000027124	-1.322266685	4.68E-09
ENSSSCG00000003705	-1.323207878	3.14E-12
ENSSSCG00000004968	-1.323571244	4.22E-05
ENSSSCG00000003950	-1.324092249	9.42E-05
ENSSSCG00000010071	-1.325515858	9.95E-09
ENSSSCG00000050618	-1.325649972	0.002399261
ENSSSCG00000045892	-1.326071939	3.51E-08
ENSSSCG00000005122	-1.326468174	1.66E-05
ENSSSCG00000037832	-1.32689844	1.91E-07
ENSSSCG00000046599	-1.327697804	0.011251542
ENSSSCG00000040967	-1.328259736	8.56E-05
ENSSSCG00000015825	-1.328886923	4.10E-06
ENSSSCG00000008241	-1.329496777	8.75E-07
ENSSSCG00000039193	-1.32976178	4.00E-07
ENSSSCG00000006308	-1.3303442	2.42E-06
ENSSSCG00000037852	-1.331049735	3.20E-06
ENSSSCG00000017797	-1.332394087	0.001854709
ENSSSCG00000032355	-1.332545167	2.45E-15
ENSSSCG00000028996	-1.33446608	1.10E-07
ENSSSCG00000034754	-1.334764197	0.000390036
ENSSSCG00000007239	-1.334925381	5.97E-05
ENSSSCG00000042541	-1.335885557	0.008466334
ENSSSCG00000037583	-1.337727041	3.50E-05
ENSSSCG00000017082	-1.337918826	2.54E-05
ENSSSCG00000034879	-1.338099075	2.54E-06
ENSSSCG00000006302	-1.338188214	0.001789384
ENSSSCG00000048120	-1.338933773	0.006533649
ENSSSCG00000017164	-1.339486147	1.87E-05
ENSSSCG00000016784	-1.340387975	9.18E-13
ENSSSCG00000029305	-1.341320212	0.009030133
ENSSSCG00000001620	-1.341596606	6.46E-07
ENSSSCG00000029395	-1.342379702	5.37E-05
ENSSSCG00000026098	-1.343024901	3.37E-06
ENSSSCG00000009817	-1.344060422	0.002332802
ENSSSCG00000011047	-1.344112807	2.26E-05
ENSSSCG00000004199	-1.344219055	1.18E-08
ENSSSCG00000022296	-1.344378841	0.007900695
ENSSSCG00000001407	-1.344455861	0.000629402
ENSSSCG00000044694	-1.344523949	0.000243413
ENSSSCG00000030511	-1.345637127	1.77E-09
ENSSSCG00000035582	-1.346352659	2.11E-06
ENSSSCG00000008340	-1.346376803	6.04E-20
ENSSSCG00000051336	-1.346464353	0.000354933
ENSSSCG00000004744	-1.347691863	0.006062304
ENSSSCG00000015882	-1.348325176	2.73E-09
ENSSSCG00000029460	-1.352025069	1.13E-07
ENSSSCG00000047463	-1.353244541	0.007936944
ENSSSCG00000010169	-1.35354374	8.42E-05
ENSSSCG00000043201	-1.354671473	5.10E-05
ENSSSCG00000016111	-1.358133566	4.63E-06
ENSSSCG00000037015	-1.358414346	1.10E-16
ENSSSCG00000010199	-1.358717027	0.001165593
ENSSSCG00000024635	-1.358888845	1.14E-06
ENSSSCG00000004658	-1.359278688	4.43E-08
ENSSSCG00000048979	-1.359486863	0.013037668

ENSSSCG00000021207	-1.359546107	0.007867087
ENSSSCG00000050958	-1.360546984	3.62E-09
ENSSSCG00000048462	-1.362526768	2.40E-06
ENSSSCG00000000435	-1.363215931	7.38E-06
ENSSSCG00000037508	-1.36364711	1.12E-05
ENSSSCG00000050398	-1.365212128	0.000327784
ENSSSCG00000042020	-1.36530037	4.70E-05
ENSSSCG00000030018	-1.365682703	0.000206838
ENSSSCG00000016691	-1.366433575	5.70E-08
ENSSSCG00000007909	-1.368177165	0.001721996
ENSSSCG00000004672	-1.368692098	4.75E-06
ENSSSCG00000005832	-1.369304233	5.69E-09
ENSSSCG00000008535	-1.36934254	0.000644042
ENSSSCG00000000853	-1.369570092	3.60E-06
ENSSSCG00000016992	-1.370724187	0.008909912
ENSSSCG00000014933	-1.370985524	8.18E-05
ENSSSCG00000007575	-1.371284142	0.00071124
ENSSSCG00000047595	-1.371503052	0.001156358
ENSSSCG00000020873	-1.371556608	3.34E-06
ENSSSCG00000004008	-1.371888031	0.011055557
ENSSSCG00000050878	-1.372243944	0.000155391
ENSSSCG00000026516	-1.372485489	0.00033392
ENSSSCG00000013351	-1.372644416	1.36E-09
ENSSSCG00000046945	-1.37275699	1.19E-07
ENSSSCG00000036941	-1.374779187	7.27E-07
ENSSSCG00000006262	-1.375829284	0.001380769
ENSSSCG00000015801	-1.376086849	6.92E-06
ENSSSCG00000012151	-1.377905064	3.18E-06
ENSSSCG00000011477	-1.379367711	1.99E-05
ENSSSCG00000003986	-1.382146504	1.10E-08
ENSSSCG00000004101	-1.382306166	5.21E-07
ENSSSCG00000012667	-1.383577792	9.54E-06
ENSSSCG00000043630	-1.384002738	0.009116763
ENSSSCG00000004898	-1.384523465	5.65E-05
ENSSSCG00000015125	-1.38502791	8.10E-05
ENSSSCG00000029073	-1.385717979	1.22E-05
ENSSSCG00000015862	-1.385772594	1.38E-05
ENSSSCG00000003702	-1.386142697	4.80E-12
ENSSSCG00000036744	-1.387531305	8.32E-06
ENSSSCG00000049494	-1.389625988	0.006964973
ENSSSCG00000009101	-1.389880083	4.58E-05
ENSSSCG00000034308	-1.390613021	6.75E-05
ENSSSCG00000012519	-1.391388618	1.73E-06
ENSSSCG00000035477	-1.391486395	0.002972886
ENSSSCG00000016337	-1.391620699	0.009254585
ENSSSCG00000005178	-1.392971063	1.17E-07
ENSSSCG00000046027	-1.393451174	0.005617034
ENSSSCG00000015612	-1.393869621	3.56E-05
ENSSSCG00000010476	-1.394120703	0.00342064
ENSSSCG00000012104	-1.394305029	8.09E-05
ENSSSCG00000045069	-1.394503196	1.81E-07
ENSSSCG00000040793	-1.395894581	1.45E-05
ENSSSCG00000006038	-1.395920379	2.77E-13
ENSSSCG00000039807	-1.396128797	3.10E-05
ENSSSCG00000038296	-1.396192821	0.000247514
ENSSSCG00000034776	-1.397100956	0.001487684
ENSSSCG00000011056	-1.397170044	3.54E-09
ENSSSCG00000006456	-1.397820717	3.46E-15

ENSSSCG00000012920	-1.398100758	8.07E-05
ENSSSCG00000023478	-1.398159731	0.000142878
ENSSSCG00000012818	-1.398278362	9.65E-11
ENSSSCG00000036622	-1.398535489	1.07E-08
ENSSSCG00000040053	-1.399221713	0.000229339
ENSSSCG00000010459	-1.399488679	2.46E-05
ENSSSCG00000009765	-1.400882101	2.07E-07
ENSSSCG00000049635	-1.40187716	0.006791391
ENSSSCG00000012944	-1.402609993	5.44E-06
ENSSSCG00000008096	-1.40261503	9.20E-15
ENSSSCG00000011495	-1.404537767	3.84E-06
ENSSSCG00000003821	-1.404764392	0.004995583
ENSSSCG00000012112	-1.405434221	0.000192056
ENSSSCG00000036049	-1.405544944	0.000127874
ENSSSCG00000021328	-1.405690682	0.010642723
ENSSSCG00000046984	-1.406238417	6.76E-05
ENSSSCG00000048222	-1.407163011	0.009190084
ENSSSCG00000030309	-1.407685486	9.39E-07
ENSSSCG00000015396	-1.409745622	2.37E-15
ENSSSCG00000021601	-1.410039796	1.86E-09
ENSSSCG00000007492	-1.412167185	1.12E-07
ENSSSCG00000006171	-1.412483749	0.00149713
ENSSSCG00000028671	-1.412979767	0.003669922
ENSSSCG00000011103	-1.413213121	4.39E-11
ENSSSCG00000012132	-1.413342792	0.008411967
ENSSSCG00000016701	-1.413406912	0.000431566
ENSSSCG00000023520	-1.413627377	0.00359585
ENSSSCG00000031288	-1.4138769	8.96E-15
ENSSSCG00000012985	-1.414144482	0.000157646
ENSSSCG00000051601	-1.4143326	3.87E-05
ENSSSCG00000007473	-1.414965073	1.43E-05
ENSSSCG00000011160	-1.416143265	0.003033794
ENSSSCG00000015270	-1.416505177	0.000341322
ENSSSCG00000006995	-1.416753553	1.81E-05
ENSSSCG00000008294	-1.416905825	0.000326845
ENSSSCG00000015545	-1.416949956	1.27E-09
ENSSSCG00000025644	-1.41929505	0.001248633
ENSSSCG00000006958	-1.419738989	1.26E-06
ENSSSCG00000027053	-1.419771271	0.000470206
ENSSSCG00000034633	-1.420088291	0.012305871
ENSSSCG00000029815	-1.420493846	9.07E-11
ENSSSCG00000020657	-1.420814454	0.002487738
ENSSSCG00000012110	-1.421640159	0.000216038
ENSSSCG00000035941	-1.421819712	0.000863916
ENSSSCG00000011110	-1.423786373	0.000623344
ENSSSCG00000039761	-1.423947341	2.15E-06
ENSSSCG00000008406	-1.424128647	0.000219454
ENSSSCG00000034948	-1.424260903	6.85E-07
ENSSSCG00000000867	-1.424422151	6.28E-12
ENSSSCG00000044825	-1.425483013	0.002130014
ENSSSCG00000002366	-1.426960538	2.60E-08
ENSSSCG00000026754	-1.427170205	4.94E-05
ENSSSCG00000027017	-1.428750478	1.45E-17
ENSSSCG00000010212	-1.429698971	2.61E-09
ENSSSCG00000004388	-1.432457311	3.63E-05
ENSSSCG00000000092	-1.434758699	0.005245423
ENSSSCG00000041142	-1.435041965	0.006382845
ENSSSCG00000016816	-1.435109991	9.83E-06

ENSSSCG00000006911	-1.435397669	4.99E-21
ENSSSCG00000003921	-1.435572127	2.63E-06
ENSSSCG00000011683	-1.436907155	2.83E-12
ENSSSCG00000000146	-1.437798868	1.83E-06
ENSSSCG00000049630	-1.438495716	0.000170925
ENSSSCG00000031149	-1.441119567	0.000140387
ENSSSCG00000038491	-1.441753874	2.46E-07
ENSSSCG00000005250	-1.442547207	0.003904248
ENSSSCG00000006115	-1.442558551	0.000381561
ENSSSCG00000012512	-1.442794675	0.002521267
ENSSSCG00000013556	-1.445237881	7.17E-05
ENSSSCG00000023596	-1.447133773	0.005757971
ENSSSCG00000016117	-1.447216307	3.69E-11
ENSSSCG00000027550	-1.447796506	0.000162181
ENSSSCG00000021158	-1.448274782	0.000748946
ENSSSCG00000033232	-1.448286649	3.30E-10
ENSSSCG00000012649	-1.448645042	3.10E-09
ENSSSCG00000033624	-1.449509844	7.82E-06
ENSSSCG00000005224	-1.450493566	1.28E-06
ENSSSCG00000024022	-1.451308946	1.20E-10
ENSSSCG00000035069	-1.452490943	0.003651497
ENSSSCG00000011404	-1.453831878	3.23E-09
ENSSSCG00000011355	-1.455052228	9.99E-12
ENSSSCG00000016810	-1.455118163	2.80E-06
ENSSSCG00000009426	-1.456233763	0.000346655
ENSSSCG00000002292	-1.456764398	0.000219966
ENSSSCG00000036751	-1.456972902	0.000843357
ENSSSCG00000001616	-1.458326909	7.89E-05
ENSSSCG00000039793	-1.458440246	0.000796037
ENSSSCG00000006524	-1.458836444	0.0007224
ENSSSCG00000011933	-1.459237327	5.98E-08
ENSSSCG00000005203	-1.460748097	5.57E-10
ENSSSCG00000049813	-1.460924226	1.04E-06
ENSSSCG00000004132	-1.462280902	3.12E-09
ENSSSCG00000010593	-1.46275571	0.002061742
ENSSSCG00000031733	-1.463812274	0.000392181
ENSSSCG00000009151	-1.464709007	1.81E-08
ENSSSCG00000024065	-1.464950838	3.05E-15
ENSSSCG00000035293	-1.467105441	0.002086848
ENSSSCG00000000801	-1.467106622	0.007149256
ENSSSCG00000004401	-1.469094547	0.000142102
ENSSSCG00000009018	-1.469223887	7.24E-13
ENSSSCG00000036761	-1.469484165	0.002537416
ENSSSCG00000037821	-1.470152771	4.27E-09
ENSSSCG00000022196	-1.470692681	0.005836495
ENSSSCG00000032956	-1.470827016	3.96E-10
ENSSSCG00000006376	-1.472973341	2.48E-06
ENSSSCG00000013286	-1.473928957	2.74E-06
ENSSSCG00000050459	-1.474003598	0.000157962
ENSSSCG00000029458	-1.474030919	1.34E-06
ENSSSCG00000012594	-1.474841789	0.000517282
ENSSSCG00000031723	-1.47653212	6.75E-05
ENSSSCG00000016567	-1.476771867	4.67E-05
ENSSSCG00000041933	-1.476823041	0.003581004
ENSSSCG00000047513	-1.477040975	0.001322142
ENSSSCG00000022504	-1.478541205	5.46E-09
ENSSSCG00000021793	-1.47950625	3.92E-10
ENSSSCG00000009300	-1.479920183	3.92E-08

ENSSSCG00000008721	-1.480154452	1.92E-06
ENSSSCG00000003761	-1.486510462	1.19E-09
ENSSSCG00000046546	-1.487103684	1.58E-05
ENSSSCG00000016782	-1.487173317	4.74E-08
ENSSSCG00000040166	-1.487297208	2.05E-10
ENSSSCG00000008593	-1.488017248	6.45E-06
ENSSSCG00000005593	-1.488329802	1.66E-08
ENSSSCG00000010665	-1.488839276	3.85E-07
ENSSSCG00000028549	-1.489715821	0.000353083
ENSSSCG00000036656	-1.490663251	3.98E-07
ENSSSCG00000032341	-1.491340798	8.60E-05
ENSSSCG00000011538	-1.491858018	0.000959482
ENSSSCG00000013010	-1.493072741	4.41E-09
ENSSSCG00000014979	-1.493741872	0.000938492
ENSSSCG00000004336	-1.493825665	4.30E-05
ENSSSCG00000049941	-1.495007916	0.001393509
ENSSSCG00000007917	-1.495138443	0.003042864
ENSSSCG00000038144	-1.495173458	1.84E-15
ENSSSCG00000013380	-1.495268741	9.63E-09
ENSSSCG00000012595	-1.495453775	0.000299012
ENSSSCG00000001977	-1.495594972	7.79E-07
ENSSSCG00000010340	-1.498960791	4.18E-17
ENSSSCG00000009407	-1.499223828	6.29E-14
ENSSSCG00000022092	-1.500401529	8.46E-14
ENSSSCG00000043715	-1.50141751	0.008648084
ENSSSCG00000023627	-1.501700168	1.88E-05
ENSSSCG00000049399	-1.502369713	0.001021859
ENSSSCG00000004736	-1.502589004	0.006626062
ENSSSCG00000049283	-1.504667189	3.22E-09
ENSSSCG00000028004	-1.504708067	2.17E-09
ENSSSCG00000015814	-1.50530518	3.77E-12
ENSSSCG00000013236	-1.506058035	6.14E-05
ENSSSCG00000024975	-1.506208737	2.17E-10
ENSSSCG00000015383	-1.507027213	5.52E-08
ENSSSCG00000016131	-1.507495724	0.007713557
ENSSSCG00000005455	-1.508424724	0.004054226
ENSSSCG00000030303	-1.509335439	2.56E-09
ENSSSCG00000029866	-1.509896947	0.00457296
ENSSSCG00000017537	-1.51036065	0.003086833
ENSSSCG00000041144	-1.510945486	0.004092786
ENSSSCG00000038839	-1.511245988	5.71E-07
ENSSSCG00000004024	-1.512002714	1.33E-07
ENSSSCG00000036123	-1.512751438	0.009532932
ENSSSCG00000009114	-1.515608478	7.38E-07
ENSSSCG00000000433	-1.517748716	2.20E-07
ENSSSCG00000046429	-1.518126471	0.00356499
ENSSSCG00000015756	-1.518146338	0.002066206
ENSSSCG00000038162	-1.519033136	0.000397131
ENSSSCG00000009429	-1.522616075	2.00E-08
ENSSSCG00000025602	-1.523305232	1.97E-09
ENSSSCG00000023666	-1.523553733	3.22E-07
ENSSSCG00000026991	-1.524874278	5.05E-06
ENSSSCG00000010925	-1.527041607	9.82E-09
ENSSSCG00000012182	-1.528141625	0.000820542
ENSSSCG00000027232	-1.528307282	8.50E-05
ENSSSCG00000036091	-1.529262306	1.58E-09
ENSSSCG00000021612	-1.529289464	4.57E-07
ENSSSCG00000027967	-1.529368932	8.00E-08

ENSSSCG00000040822	-1.52960095	0.008239229
ENSSSCG00000015211	-1.530385196	2.72E-10
ENSSSCG00000004661	-1.53057665	0.007127855
ENSSSCG00000025208	-1.5308883	0.008017852
ENSSSCG00000015522	-1.535045276	0.003776236
ENSSSCG00000022933	-1.535156607	4.02E-05
ENSSSCG00000034976	-1.535715383	1.80E-15
ENSSSCG00000031536	-1.538273965	3.92E-06
ENSSSCG00000006331	-1.538363257	4.54E-14
ENSSSCG00000009490	-1.538633747	1.11E-10
ENSSSCG00000008319	-1.53866786	0.012973531
ENSSSCG00000049589	-1.539153046	0.0001636
ENSSSCG00000043770	-1.53978177	0.000253344
ENSSSCG00000003619	-1.541767991	0.001320185
ENSSSCG00000024818	-1.543376388	5.54E-09
ENSSSCG00000034491	-1.544336719	3.35E-05
ENSSSCG00000040838	-1.546575264	8.71E-06
ENSSSCG00000048909	-1.547671486	0.002949433
ENSSSCG00000010256	-1.549747924	0.005181761
ENSSSCG00000044105	-1.54984595	7.68E-05
ENSSSCG00000031716	-1.550487529	3.35E-13
ENSSSCG00000023788	-1.551128368	5.47E-05
ENSSSCG00000003351	-1.552448785	8.63E-06
ENSSSCG00000017204	-1.552900057	2.39E-05
ENSSSCG00000002368	-1.553730889	6.64E-09
ENSSSCG00000021911	-1.554237961	8.81E-09
ENSSSCG00000050397	-1.555856591	0.000403569
ENSSSCG00000015937	-1.556403973	6.93E-05
ENSSSCG00000024954	-1.557877778	1.10E-08
ENSSSCG00000005762	-1.558771126	0.000156239
ENSSSCG00000010447	-1.560233438	0.000553021
ENSSSCG00000029606	-1.56063409	0.000140169
ENSSSCG00000015196	-1.562333051	8.85E-14
ENSSSCG00000012002	-1.564401792	7.65E-08
ENSSSCG00000004584	-1.566103315	0.003228621
ENSSSCG00000012155	-1.566606593	1.18E-06
ENSSSCG00000013344	-1.567646688	1.74E-05
ENSSSCG00000031706	-1.56769635	4.40E-07
ENSSSCG00000012564	-1.567890443	4.99E-07
ENSSSCG00000022467	-1.568981603	0.000969566
ENSSSCG00000028691	-1.570047168	0.000377978
ENSSSCG00000045050	-1.570945436	1.35E-11
ENSSSCG00000040798	-1.57146485	0.005006392
ENSSSCG00000005071	-1.571649875	0.004360734
ENSSSCG00000026473	-1.571697997	0.000771175
ENSSSCG00000023749	-1.57308682	6.22E-12
ENSSSCG00000032709	-1.573172542	8.68E-13
ENSSSCG00000017257	-1.573482128	4.80E-10
ENSSSCG00000038414	-1.574398882	0.00571137
ENSSSCG00000006981	-1.576692162	1.84E-06
ENSSSCG00000004318	-1.577339473	0.000798626
ENSSSCG00000003386	-1.57769069	5.75E-17
ENSSSCG00000022236	-1.578151608	1.31E-09
ENSSSCG00000009230	-1.58174337	7.74E-07
ENSSSCG00000021105	-1.582209115	7.71E-08
ENSSSCG00000011030	-1.583307236	0.000540168
ENSSSCG00000016628	-1.584413339	0.001690776
ENSSSCG00000015402	-1.58564648	2.12E-09

ENSSSCG00000000292	-1.586044317	0.001663946
ENSSSCG00000015747	-1.586101577	0.000229564
ENSSSCG00000025109	-1.58694095	1.98E-06
ENSSSCG00000006197	-1.587270182	8.62E-06
ENSSSCG00000021309	-1.587298539	1.22E-16
ENSSSCG00000003881	-1.592820017	6.39E-06
ENSSSCG00000048896	-1.593808276	2.85E-05
ENSSSCG00000038383	-1.594237102	6.09E-10
ENSSSCG00000037766	-1.594763045	2.44E-05
ENSSSCG00000013754	-1.596036485	0.002373223
ENSSSCG00000009217	-1.596036893	2.74E-06
ENSSSCG00000037991	-1.59621868	5.35E-10
ENSSSCG00000049586	-1.596963959	0.011704318
ENSSSCG00000026547	-1.600586618	2.44E-10
ENSSSCG00000021874	-1.601415162	2.21E-08
ENSSSCG00000001901	-1.601957895	0.000127084
ENSSSCG00000032444	-1.60265083	3.53E-06
ENSSSCG00000028144	-1.602736099	1.61E-05
ENSSSCG00000015780	-1.604135526	0.000522271
ENSSSCG00000028536	-1.604299207	1.71E-17
ENSSSCG00000002795	-1.60454413	4.59E-26
ENSSSCG00000003715	-1.604953671	1.38E-08
ENSSSCG00000025306	-1.605525993	3.52E-05
ENSSSCG00000013273	-1.606292624	1.07E-09
ENSSSCG00000016035	-1.606424673	6.59E-17
ENSSSCG00000016686	-1.607661545	0.00184658
ENSSSCG00000041170	-1.608035985	9.01E-05
ENSSSCG00000043563	-1.610442792	5.55E-06
ENSSSCG00000009330	-1.611770977	0.00226717
ENSSSCG00000050772	-1.613157346	2.06E-06
ENSSSCG00000017300	-1.614428627	2.81E-08
ENSSSCG00000025924	-1.615138137	0.002136542
ENSSSCG00000011260	-1.615619854	0.005779812
ENSSSCG00000025417	-1.61647807	6.45E-14
ENSSSCG00000037137	-1.616554288	4.03E-11
ENSSSCG00000027331	-1.618029838	0.000243512
ENSSSCG00000013978	-1.618478173	4.72E-11
ENSSSCG00000024800	-1.619508119	6.80E-07
ENSSSCG00000026180	-1.620278779	2.88E-08
ENSSSCG00000026427	-1.62088013	1.36E-05
ENSSSCG00000001723	-1.625668709	7.95E-09
ENSSSCG00000033175	-1.626464483	7.34E-19
ENSSSCG00000005094	-1.62666619	0.000905925
ENSSSCG00000028101	-1.628189543	1.17E-09
ENSSSCG00000002737	-1.628416609	1.28E-10
ENSSSCG00000001849	-1.631088383	4.00E-05
ENSSSCG00000034973	-1.632240524	3.40E-08
ENSSSCG00000040377	-1.633647529	1.25E-09
ENSSSCG00000046476	-1.63525629	0.005557808
ENSSSCG00000029199	-1.635387476	0.000206561
ENSSSCG00000024614	-1.635470212	2.52E-10
ENSSSCG00000041324	-1.635988919	2.14E-05
ENSSSCG00000036033	-1.636558637	8.13E-05
ENSSSCG00000020963	-1.637380494	0.001135959
ENSSSCG00000000549	-1.638874265	4.19E-24
ENSSSCG00000014766	-1.639084663	0.00015816
ENSSSCG00000042021	-1.641045646	0.000217611
ENSSSCG00000039175	-1.641822597	9.76E-11

ENSSSCG00000003088	-1.641983888	2.65E-18
ENSSSCG00000041436	-1.642182319	0.005004751
ENSSSCG00000033937	-1.643196108	5.52E-09
ENSSSCG00000032852	-1.643816358	1.28E-12
ENSSSCG00000015874	-1.646136404	1.61E-11
ENSSSCG00000012437	-1.646586337	1.09E-12
ENSSSCG00000015774	-1.646801227	4.52E-10
ENSSSCG00000001582	-1.647046123	0.013218389
ENSSSCG00000000687	-1.647866974	1.32E-09
ENSSSCG00000044546	-1.649749691	7.45E-06
ENSSSCG00000027505	-1.652772962	0.002423213
ENSSSCG00000008488	-1.654138768	4.16E-11
ENSSSCG00000013102	-1.65452918	7.85E-06
ENSSSCG00000008899	-1.654571837	9.10E-05
ENSSSCG00000000784	-1.659133451	1.57E-24
ENSSSCG00000011819	-1.65920799	2.69E-08
ENSSSCG00000035152	-1.659222338	1.55E-14
ENSSSCG00000002379	-1.665075977	0.0008486
ENSSSCG00000013278	-1.665432545	1.10E-13
ENSSSCG00000004404	-1.666016618	2.24E-06
ENSSSCG00000012838	-1.667168553	2.11E-05
ENSSSCG00000036758	-1.668177577	8.41E-06
ENSSSCG00000004973	-1.67019898	2.13E-08
ENSSSCG00000007100	-1.67370743	0.001402312
ENSSSCG00000011782	-1.675215162	1.42E-10
ENSSSCG00000021511	-1.675963353	0.000269755
ENSSSCG00000039947	-1.677832806	1.06E-06
ENSSSCG00000010949	-1.677931603	4.83E-05
ENSSSCG00000009860	-1.678646754	4.16E-07
ENSSSCG00000028443	-1.679371331	8.16E-10
ENSSSCG00000025286	-1.681025258	0.000174877
ENSSSCG00000038536	-1.681751848	4.28E-05
ENSSSCG00000042312	-1.682372249	1.09E-10
ENSSSCG00000044675	-1.683162271	9.09E-05
ENSSSCG00000024205	-1.684220992	0.00016682
ENSSSCG00000010359	-1.688655868	0.012255391
ENSSSCG00000017818	-1.688708272	3.30E-15
ENSSSCG00000025126	-1.689120863	3.75E-06
ENSSSCG00000020736	-1.690321726	0.005617588
ENSSSCG00000043992	-1.691329679	9.72E-06
ENSSSCG00000000665	-1.692436714	5.45E-05
ENSSSCG00000008888	-1.693499701	0.00061964
ENSSSCG00000028530	-1.694278994	0.000674676
ENSSSCG00000043867	-1.695014626	0.000171091
ENSSSCG00000010048	-1.695052997	0.013161623
ENSSSCG00000006296	-1.695675211	3.63E-08
ENSSSCG00000008646	-1.695896321	1.94E-17
ENSSSCG00000010529	-1.696235792	8.29E-12
ENSSSCG00000004962	-1.696237648	1.46E-08
ENSSSCG00000049358	-1.697969147	0.006129521
ENSSSCG00000002868	-1.701951243	3.06E-13
ENSSSCG00000039373	-1.702245438	4.06E-14
ENSSSCG00000034607	-1.703510192	3.66E-09
ENSSSCG00000041698	-1.70502969	0.001646661
ENSSSCG00000046749	-1.705223173	0.005444774
ENSSSCG00000021222	-1.706014108	1.21E-05
ENSSSCG00000030088	-1.708010556	2.48E-12
ENSSSCG00000016705	-1.709523652	1.83E-05

ENSSSCG00000035371	-1.709553476	1.07E-08
ENSSSCG00000049971	-1.710127632	0.004637206
ENSSSCG00000040198	-1.710833068	0.000421018
ENSSSCG00000030432	-1.714119583	7.79E-05
ENSSSCG00000008103	-1.715103323	3.43E-05
ENSSSCG00000008510	-1.716052541	1.22E-11
ENSSSCG00000006390	-1.716571579	8.31E-10
ENSSSCG00000002708	-1.717991372	3.18E-06
ENSSSCG00000011511	-1.718347954	5.27E-17
ENSSSCG00000003547	-1.719546619	6.75E-06
ENSSSCG00000011514	-1.720016104	2.23E-15
ENSSSCG00000011020	-1.722161491	1.13E-07
ENSSSCG00000010559	-1.722569792	0.000384941
ENSSSCG00000012627	-1.725005084	1.78E-06
ENSSSCG00000002954	-1.72589086	2.01E-07
ENSSSCG00000006082	-1.72966687	0.000256221
ENSSSCG00000035617	-1.730306536	0.005454225
ENSSSCG00000024299	-1.730586526	2.01E-05
ENSSSCG00000023273	-1.730808306	3.27E-05
ENSSSCG00000037697	-1.731721957	1.30E-10
ENSSSCG00000011813	-1.733481959	0.000360438
ENSSSCG00000011577	-1.73417953	9.83E-14
ENSSSCG00000024000	-1.734489216	7.35E-05
ENSSSCG00000011037	-1.734941594	7.29E-06
ENSSSCG00000023762	-1.735365439	1.21E-05
ENSSSCG00000029227	-1.735474143	8.31E-16
ENSSSCG00000051511	-1.737573565	9.24E-05
ENSSSCG00000023933	-1.738151269	0.003213464
ENSSSCG00000004012	-1.73856473	1.59E-08
ENSSSCG00000044010	-1.74129551	1.06E-05
ENSSSCG00000024671	-1.742595459	0.000217323
ENSSSCG00000017355	-1.742787194	2.14E-08
ENSSSCG00000006947	-1.744665041	9.94E-06
ENSSSCG00000016690	-1.745182767	0.00214489
ENSSSCG00000035400	-1.746982769	2.67E-16
ENSSSCG00000028804	-1.748129735	0.001214925
ENSSSCG00000020758	-1.749720921	5.92E-08
ENSSSCG00000001107	-1.750094201	5.67E-06
ENSSSCG00000015540	-1.752011268	5.40E-15
ENSSSCG00000009221	-1.752081086	6.94E-06
ENSSSCG00000009331	-1.754036201	1.45E-05
ENSSSCG00000003709	-1.75649468	2.97E-06
ENSSSCG00000004193	-1.758570677	2.83E-17
ENSSSCG00000024403	-1.758942646	6.53E-12
ENSSSCG00000038148	-1.759273286	1.72E-06
ENSSSCG00000023479	-1.760633319	0.001843035
ENSSSCG00000004087	-1.76350625	2.00E-10
ENSSSCG00000032058	-1.764900037	0.009637294
ENSSSCG00000006172	-1.766273669	0.000118559
ENSSSCG00000015412	-1.768730173	0.005262868
ENSSSCG00000022390	-1.768981801	0.006996438
ENSSSCG00000001726	-1.769161693	0.004726171
ENSSSCG00000015556	-1.769205744	9.82E-07
ENSSSCG00000033807	-1.770781572	0.002172658
ENSSSCG00000000216	-1.771959552	3.17E-07
ENSSSCG00000036007	-1.772381435	1.19E-06
ENSSSCG00000039926	-1.774270018	0.00110846
ENSSSCG00000017511	-1.775314247	0.001293036

ENSSSCG00000014310	-1.775800532	1.53E-09
ENSSSCG00000009322	-1.775874933	1.17E-06
ENSSSCG00000034440	-1.776290102	0.001136203
ENSSSCG00000010800	-1.776835445	3.56E-07
ENSSSCG00000040445	-1.778767063	4.48E-09
ENSSSCG00000040461	-1.779018677	0.002084146
ENSSSCG00000014182	-1.77904994	1.11E-05
ENSSSCG00000022989	-1.77933162	0.000485434
ENSSSCG00000000680	-1.781295629	0.000277278
ENSSSCG00000041105	-1.781443215	0.00071781
ENSSSCG00000010974	-1.783441179	6.31E-07
ENSSSCG00000041180	-1.786032644	5.72E-08
ENSSSCG00000035218	-1.786598163	3.97E-06
ENSSSCG00000023848	-1.786679438	2.38E-07
ENSSSCG00000012911	-1.787948705	9.32E-07
ENSSSCG00000005186	-1.791745864	2.67E-14
ENSSSCG00000006702	-1.792199412	4.57E-14
ENSSSCG00000015357	-1.792379214	4.38E-15
ENSSSCG00000006648	-1.792528992	1.78E-09
ENSSSCG00000002866	-1.796609427	7.22E-07
ENSSSCG00000002788	-1.797009941	2.23E-06
ENSSSCG00000027882	-1.79742053	0.000457497
ENSSSCG00000016866	-1.797426199	4.16E-05
ENSSSCG00000012278	-1.798584994	5.46E-18
ENSSSCG00000013401	-1.799058392	3.31E-09
ENSSSCG00000008073	-1.799823222	3.78E-08
ENSSSCG00000015815	-1.800066527	1.29E-05
ENSSSCG00000012481	-1.801361862	2.54E-15
ENSSSCG00000045249	-1.802315338	0.000471663
ENSSSCG00000037334	-1.80249564	4.93E-06
ENSSSCG00000011129	-1.802530784	0.001135521
ENSSSCG00000030821	-1.802625171	0.000897201
ENSSSCG00000041254	-1.803726147	5.42E-05
ENSSSCG00000000206	-1.805159408	0.012136647
ENSSSCG00000015379	-1.805568061	0.001294716
ENSSSCG00000017908	-1.805661322	0.000159844
ENSSSCG00000042873	-1.806430543	2.31E-06
ENSSSCG00000005055	-1.806713286	4.22E-08
ENSSSCG00000015271	-1.806778787	0.000366349
ENSSSCG00000036190	-1.807395722	1.00E-06
ENSSSCG00000040850	-1.810904029	3.61E-05
ENSSSCG00000008771	-1.811019468	8.13E-05
ENSSSCG00000023584	-1.812727459	1.25E-05
ENSSSCG00000021374	-1.816217881	0.000251363
ENSSSCG00000036618	-1.816229503	0.000168954
ENSSSCG00000049462	-1.816625793	1.07E-06
ENSSSCG00000025176	-1.817411498	0.000541489
ENSSSCG00000000456	-1.818786794	2.37E-05
ENSSSCG00000003146	-1.818985162	2.69E-05
ENSSSCG00000036135	-1.81989778	5.06E-06
ENSSSCG00000001963	-1.820270982	2.89E-11
ENSSSCG00000008504	-1.824637232	4.78E-13
ENSSSCG00000024071	-1.82578269	3.89E-09
ENSSSCG00000015810	-1.827145707	3.95E-08
ENSSSCG00000015329	-1.827868011	5.19E-12
ENSSSCG00000006087	-1.828934096	1.41E-17
ENSSSCG00000008799	-1.828984731	2.59E-10
ENSSSCG00000011712	-1.830369697	4.49E-07

ENSSSCG00000048314	-1.830486542	0.000297757
ENSSSCG00000040416	-1.830870952	3.91E-16
ENSSSCG00000017380	-1.8308876	2.63E-11
ENSSSCG00000036379	-1.831386369	0.00403517
ENSSSCG00000028327	-1.832391687	1.15E-09
ENSSSCG00000024088	-1.833639712	3.20E-09
ENSSSCG00000016285	-1.834197686	0.013288412
ENSSSCG00000006173	-1.83604208	0.00239689
ENSSSCG00000012027	-1.836862343	1.71E-09
ENSSSCG00000035387	-1.837003616	0.0001622
ENSSSCG00000030172	-1.83771794	3.78E-05
ENSSSCG00000011915	-1.837804024	7.28E-07
ENSSSCG00000011878	-1.840108813	7.14E-13
ENSSSCG00000000455	-1.84047612	1.17E-14
ENSSSCG00000009152	-1.840622218	1.32E-26
ENSSSCG00000008486	-1.842059403	3.65E-07
ENSSSCG00000030013	-1.842925251	0.000432426
ENSSSCG00000029838	-1.843194704	0.006193429
ENSSSCG00000006415	-1.846972248	0.00365285
ENSSSCG00000025822	-1.847225257	0.000195181
ENSSSCG00000010450	-1.850445645	1.18E-17
ENSSSCG00000048770	-1.850898958	0.002846256
ENSSSCG00000006247	-1.853117195	0.00236535
ENSSSCG00000020717	-1.853272627	2.19E-06
ENSSSCG00000006344	-1.854206201	2.34E-05
ENSSSCG00000007572	-1.854408553	2.76E-15
ENSSSCG00000011400	-1.85462339	2.45E-14
ENSSSCG00000044246	-1.855834226	9.08E-06
ENSSSCG00000032282	-1.856040399	4.36E-09
ENSSSCG00000032197	-1.859759045	0.003687394
ENSSSCG00000012153	-1.86127685	8.22E-05
ENSSSCG00000046619	-1.862930011	0.004479973
ENSSSCG00000004795	-1.86444637	6.20E-22
ENSSSCG00000033093	-1.864912511	8.72E-06
ENSSSCG00000002443	-1.86514343	1.02E-13
ENSSSCG00000038598	-1.865622219	0.00034917
ENSSSCG00000017637	-1.865878239	0.010560994
ENSSSCG00000004485	-1.865984009	0.000130173
ENSSSCG00000033185	-1.868490555	1.76E-06
ENSSSCG00000006183	-1.871098611	8.72E-06
ENSSSCG00000036851	-1.872760403	1.29E-13
ENSSSCG00000032433	-1.873559503	0.000535742
ENSSSCG00000014943	-1.874368516	0.00023518
ENSSSCG00000051107	-1.874923787	0.000298516
ENSSSCG00000046327	-1.878666131	0.011047526
ENSSSCG00000009751	-1.879172219	4.00E-10
ENSSSCG00000010209	-1.879479039	0.00039076
ENSSSCG00000047020	-1.881049433	2.01E-06
ENSSSCG00000014117	-1.88257513	0.000378923
ENSSSCG00000015036	-1.885408611	2.00E-09
ENSSSCG00000050888	-1.887024101	0.004843245
ENSSSCG00000015507	-1.892537111	0.007511933
ENSSSCG00000003521	-1.892936284	2.38E-06
ENSSSCG00000036933	-1.893139521	1.70E-07
ENSSSCG00000015326	-1.894004937	2.18E-06
ENSSSCG00000009966	-1.894244439	0.00057428
ENSSSCG00000042332	-1.895184956	7.16E-05
ENSSSCG00000005266	-1.896235387	0.000210746

ENSSSCG00000024496	-1.896483509	0.008526876
ENSSSCG00000047673	-1.898301003	0.008568116
ENSSSCG00000017144	-1.898760773	8.32E-05
ENSSSCG00000035445	-1.898936892	0.000149631
ENSSSCG00000003539	-1.900441003	5.75E-16
ENSSSCG00000024958	-1.902436613	5.62E-09
ENSSSCG00000025005	-1.903623015	7.26E-11
ENSSSCG00000006982	-1.903667928	1.25E-09
ENSSSCG00000011521	-1.904937024	2.60E-10
ENSSSCG00000007727	-1.908311353	8.11E-09
ENSSSCG00000021647	-1.909272234	8.24E-06
ENSSSCG00000004453	-1.915191142	0.000410439
ENSSSCG00000046841	-1.916866217	0.011758158
ENSSSCG00000050866	-1.918558825	0.003745402
ENSSSCG00000045074	-1.918815807	0.006569019
ENSSSCG00000004714	-1.927628846	8.51E-06
ENSSSCG00000037905	-1.928635533	5.04E-08
ENSSSCG00000041248	-1.929071478	0.000567553
ENSSSCG00000007140	-1.931445347	2.46E-21
ENSSSCG00000047677	-1.933983257	3.23E-05
ENSSSCG00000013658	-1.93565079	2.87E-08
ENSSSCG00000004402	-1.935794192	0.006058476
ENSSSCG00000010654	-1.935891875	1.83E-11
ENSSSCG00000034191	-1.936696827	0.000248244
ENSSSCG00000016260	-1.936943376	3.26E-07
ENSSSCG00000014672	-1.938804772	6.17E-21
ENSSSCG00000032612	-1.942127444	2.00E-11
ENSSSCG00000015619	-1.942136305	0.000416211
ENSSSCG00000010745	-1.942327691	8.95E-05
ENSSSCG00000016442	-1.946684369	1.50E-09
ENSSSCG00000005360	-1.94730646	4.88E-06
ENSSSCG00000047137	-1.947761779	0.010868744
ENSSSCG00000026819	-1.94810876	4.89E-15
ENSSSCG00000016625	-1.95006404	2.29E-17
ENSSSCG00000045950	-1.951250827	0.005625901
ENSSSCG00000011216	-1.955564489	5.65E-06
ENSSSCG00000039333	-1.957226832	0.002530219
ENSSSCG00000010816	-1.959303873	2.96E-20
ENSSSCG00000031346	-1.960158945	0.00047013
ENSSSCG00000026084	-1.962319631	7.82E-13
ENSSSCG00000037425	-1.965194839	4.71E-05
ENSSSCG00000005020	-1.96614072	0.002857154
ENSSSCG00000013940	-1.969013715	7.70E-07
ENSSSCG00000017307	-1.971811944	0.000634325
ENSSSCG00000016184	-1.972254149	5.98E-07
ENSSSCG00000001076	-1.97344211	3.13E-08
ENSSSCG00000007034	-1.975656928	7.05E-09
ENSSSCG00000031788	-1.975841184	5.34E-07
ENSSSCG00000024067	-1.977702818	8.86E-10
ENSSSCG00000003722	-1.978967866	2.71E-10
ENSSSCG00000034198	-1.98068256	0.002820406
ENSSSCG00000004191	-1.981649221	3.18E-05
ENSSSCG00000037997	-1.982081272	9.43E-12
ENSSSCG00000005997	-1.983218476	0.000405308
ENSSSCG00000017068	-1.983606301	7.06E-17
ENSSSCG00000009357	-1.983712723	1.00E-20
ENSSSCG00000013360	-1.98486854	2.29E-18
ENSSSCG00000036265	-1.985074797	0.000760629

ENSSSCG00000050679	-1.990484328	0.002575186
ENSSSCG00000045152	-1.99366231	2.89E-09
ENSSSCG00000045839	-1.994103223	1.83E-05
ENSSSCG00000038844	-1.994920311	8.61E-05
ENSSSCG00000001567	-1.999525576	1.39E-06
ENSSSCG00000001570	-1.999879756	0.000357985
ENSSSCG00000033352	-2.002997534	1.40E-05
ENSSSCG00000015294	-2.00508414	5.64E-12
ENSSSCG00000042937	-2.005239533	0.007855592
ENSSSCG00000013248	-2.005884866	2.48E-07
ENSSSCG00000014123	-2.007692816	2.00E-11
ENSSSCG00000024492	-2.009005326	0.000302481
ENSSSCG00000017012	-2.010317176	0.000220104
ENSSSCG00000005608	-2.010808293	3.71E-09
ENSSSCG00000011928	-2.011934558	1.01E-09
ENSSSCG00000020735	-2.012775628	0.003041873
ENSSSCG00000051365	-2.019226679	2.83E-06
ENSSSCG00000050137	-2.022414628	9.99E-06
ENSSSCG00000027872	-2.024291771	4.46E-05
ENSSSCG00000025772	-2.025687167	5.70E-05
ENSSSCG00000022895	-2.02838921	3.15E-23
ENSSSCG00000009535	-2.032105954	1.37E-16
ENSSSCG00000000006	-2.033617773	9.71E-06
ENSSSCG00000012452	-2.034222154	7.64E-16
ENSSSCG00000016504	-2.035211204	1.10E-10
ENSSSCG00000005284	-2.037104371	4.26E-05
ENSSSCG00000039271	-2.039532731	0.000157065
ENSSSCG00000032241	-2.040338039	2.37E-08
ENSSSCG00000027144	-2.041031555	0.004078794
ENSSSCG00000016123	-2.045073344	1.93E-08
ENSSSCG00000000010	-2.046585145	7.95E-07
ENSSSCG00000000857	-2.046941861	5.17E-07
ENSSSCG00000049185	-2.050615654	5.04E-06
ENSSSCG00000025992	-2.053819814	2.93E-12
ENSSSCG00000028204	-2.054001149	0.005931023
ENSSSCG00000006350	-2.056670023	1.56E-12
ENSSSCG00000047770	-2.057464746	7.99E-07
ENSSSCG00000043778	-2.058929633	0.002104425
ENSSSCG00000021818	-2.061553696	0.000470086
ENSSSCG00000037987	-2.064225458	0.007159759
ENSSSCG00000005475	-2.064761043	0.000435631
ENSSSCG00000001478	-2.066455048	0.005887618
ENSSSCG00000023784	-2.067240524	3.51E-05
ENSSSCG00000037466	-2.06752012	4.37E-07
ENSSSCG00000042146	-2.068094748	0.00552911
ENSSSCG00000023498	-2.068713408	8.91E-13
ENSSSCG00000029771	-2.070979585	1.13E-07
ENSSSCG00000032162	-2.072557883	0.000122112
ENSSSCG00000045590	-2.074700864	0.0001236
ENSSSCG00000045681	-2.075564888	0.00574895
ENSSSCG00000012467	-2.076747525	5.37E-06
ENSSSCG00000004931	-2.078021141	3.53E-11
ENSSSCG00000032438	-2.079207558	0.000156553
ENSSSCG00000006132	-2.080186751	0.003526337
ENSSSCG00000048827	-2.081239975	0.009830391
ENSSSCG00000014235	-2.084514742	5.23E-11
ENSSSCG00000005164	-2.087113634	3.92E-08
ENSSSCG00000046226	-2.089646649	0.00279721

ENSSSCG00000010426	-2.089669127	4.37E-15
ENSSSCG00000001715	-2.090737096	1.83E-05
ENSSSCG00000008919	-2.09085288	0.000207231
ENSSSCG00000011397	-2.091102126	6.04E-06
ENSSSCG00000011269	-2.092252578	2.21E-05
ENSSSCG00000025711	-2.093521613	1.34E-17
ENSSSCG00000009011	-2.095777663	7.85E-16
ENSSSCG00000013335	-2.096385069	5.11E-06
ENSSSCG00000002814	-2.097343483	1.36E-05
ENSSSCG00000033355	-2.097371918	0.002701962
ENSSSCG00000011527	-2.101175658	0.000292561
ENSSSCG00000015035	-2.1034234	0.001712504
ENSSSCG00000037351	-2.104447519	2.78E-06
ENSSSCG00000037195	-2.106081006	0.000822595
ENSSSCG00000022661	-2.108644843	9.99E-13
ENSSSCG00000023229	-2.108966095	9.56E-21
ENSSSCG00000017604	-2.110608887	7.02E-12
ENSSSCG00000050870	-2.114948831	0.000455276
ENSSSCG00000013787	-2.117768582	1.70E-05
ENSSSCG00000010746	-2.120367303	2.68E-23
ENSSSCG00000005222	-2.122780074	5.89E-08
ENSSSCG00000038192	-2.124762786	0.010405028
ENSSSCG00000028135	-2.124793477	1.67E-18
ENSSSCG00000007436	-2.126683064	3.98E-07
ENSSSCG00000008835	-2.127787837	4.62E-12
ENSSSCG00000023924	-2.130696941	4.39E-08
ENSSSCG00000023084	-2.133637019	9.02E-09
ENSSSCG00000041926	-2.133978702	0.002883721
ENSSSCG00000003604	-2.134436612	2.10E-07
ENSSSCG00000035495	-2.135454633	2.86E-09
ENSSSCG00000028338	-2.136080368	1.76E-16
ENSSSCG00000040275	-2.136324616	1.60E-20
ENSSSCG00000015002	-2.137405582	1.28E-07
ENSSSCG00000022446	-2.138702375	0.000154761
ENSSSCG00000043488	-2.13963961	0.004022671
ENSSSCG00000051680	-2.140177034	7.97E-11
ENSSSCG00000014034	-2.141015768	5.78E-14
ENSSSCG00000031267	-2.146997684	0.004410896
ENSSSCG00000008749	-2.150305091	6.57E-13
ENSSSCG00000042822	-2.150341968	3.70E-05
ENSSSCG00000035891	-2.151166694	2.66E-05
ENSSSCG00000038418	-2.155428988	0.00104599
ENSSSCG00000014047	-2.157976773	3.11E-06
ENSSSCG00000016652	-2.162019563	1.19E-06
ENSSSCG00000050342	-2.163140401	0.007846798
ENSSSCG00000015238	-2.163972301	2.32E-07
ENSSSCG00000015766	-2.165185851	3.41E-07
ENSSSCG00000048240	-2.168447943	2.20E-08
ENSSSCG00000017036	-2.169319634	0.011291409
ENSSSCG00000008618	-2.170039195	3.35E-05
ENSSSCG00000011463	-2.173169607	4.55E-10
ENSSSCG00000000848	-2.174050604	3.54E-10
ENSSSCG00000001483	-2.177319364	0.001020356
ENSSSCG00000045205	-2.179064233	1.70E-06
ENSSSCG00000009645	-2.183519836	7.84E-06
ENSSSCG00000000062	-2.184146886	2.77E-06
ENSSSCG00000016555	-2.184323994	0.009667589
ENSSSCG00000037539	-2.185159023	9.91E-07

ENSSSCG00000011609	-2.185938081	1.10E-05
ENSSSCG00000001804	-2.186317265	8.58E-08
ENSSSCG00000024552	-2.188586926	0.003699945
ENSSSCG00000007926	-2.190486956	0.000214076
ENSSSCG00000031694	-2.191918058	0.00013289
ENSSSCG00000001906	-2.192516761	0.004376989
ENSSSCG00000039056	-2.192549998	1.62E-14
ENSSSCG00000026499	-2.193151946	2.90E-06
ENSSSCG00000014097	-2.19572614	9.15E-08
ENSSSCG00000027745	-2.195942103	2.52E-18
ENSSSCG00000000601	-2.197953603	0.000287237
ENSSSCG00000023014	-2.199274024	0.000297791
ENSSSCG00000011443	-2.20007817	8.46E-20
ENSSSCG00000049475	-2.200799551	1.78E-06
ENSSSCG00000046137	-2.202035727	0.002613013
ENSSSCG00000016002	-2.216112861	5.02E-06
ENSSSCG00000000516	-2.222200458	0.001628809
ENSSSCG00000007198	-2.228027073	0.000760096
ENSSSCG00000041565	-2.23253622	0.011411164
ENSSSCG00000035091	-2.232611879	1.03E-13
ENSSSCG00000007760	-2.234142882	0.007579017
ENSSSCG00000007139	-2.234471115	0.007090701
ENSSSCG00000016665	-2.238968831	5.37E-36
ENSSSCG00000008853	-2.240333954	3.62E-07
ENSSSCG00000012362	-2.241833681	1.26E-11
ENSSSCG00000042325	-2.242051054	0.004288219
ENSSSCG00000011002	-2.242456195	9.32E-07
ENSSSCG00000017063	-2.245765974	0.002462214
ENSSSCG00000035554	-2.246802626	3.95E-06
ENSSSCG00000033394	-2.254177491	1.23E-07
ENSSSCG00000026478	-2.255413023	0.000238153
ENSSSCG00000033268	-2.256944231	2.77E-05
ENSSSCG00000036441	-2.259707355	0.002349958
ENSSSCG00000015584	-2.262507388	1.29E-19
ENSSSCG00000049212	-2.263732597	0.00574744
ENSSSCG00000004534	-2.266526184	0.011212099
ENSSSCG00000013145	-2.267649435	2.91E-20
ENSSSCG00000008124	-2.268216614	4.29E-08
ENSSSCG00000044695	-2.272119437	8.49E-06
ENSSSCG00000025687	-2.274401758	1.82E-07
ENSSSCG00000043410	-2.274775772	0.000556537
ENSSSCG00000009489	-2.278437822	1.74E-25
ENSSSCG00000008961	-2.278789966	7.02E-06
ENSSSCG00000048556	-2.278837412	1.89E-17
ENSSSCG00000039986	-2.279416815	5.12E-05
ENSSSCG00000031649	-2.279886512	3.56E-08
ENSSSCG00000045353	-2.280051089	3.05E-09
ENSSSCG00000035025	-2.2804055	5.17E-08
ENSSSCG00000048917	-2.280865403	6.54E-05
ENSSSCG00000014984	-2.281514967	0.008481662
ENSSSCG00000004961	-2.284806349	3.31E-05
ENSSSCG00000004484	-2.288885964	1.62E-21
ENSSSCG00000044610	-2.291719321	0.008092239
ENSSSCG00000033146	-2.299121539	1.94E-08
ENSSSCG00000038616	-2.299735641	0.007102059
ENSSSCG00000014897	-2.302491768	0.000171039
ENSSSCG00000005257	-2.30636441	1.59E-07
ENSSSCG00000005975	-2.307491369	1.67E-30

ENSSSCG00000040746	-2.30831862	3.13E-20
ENSSSCG00000044883	-2.30944082	0.000363017
ENSSSCG00000035661	-2.310815314	0.003086874
ENSSSCG00000010142	-2.314592715	4.16E-08
ENSSSCG00000022568	-2.318626764	0.000319521
ENSSSCG00000006737	-2.318823892	1.39E-12
ENSSSCG00000011766	-2.319332119	5.82E-06
ENSSSCG00000014314	-2.320455397	0.000215284
ENSSSCG00000015657	-2.32075898	4.09E-12
ENSSSCG00000012034	-2.323153372	2.21E-17
ENSSSCG00000013584	-2.323720377	0.004666035
ENSSSCG00000047149	-2.325627671	4.48E-16
ENSSSCG00000015375	-2.330620258	8.20E-09
ENSSSCG00000010011	-2.333744169	1.36E-19
ENSSSCG00000043775	-2.333885334	0.001487775
ENSSSCG00000000478	-2.339803069	0.000598938
ENSSSCG00000011750	-2.341883826	3.45E-14
ENSSSCG00000016397	-2.343632959	0.010320447
ENSSSCG00000036052	-2.345309793	0.009391363
ENSSSCG00000040956	-2.347853285	1.11E-08
ENSSSCG00000010959	-2.35017748	1.37E-10
ENSSSCG00000023451	-2.350405878	0.0059599
ENSSSCG00000034853	-2.350441406	1.43E-12
ENSSSCG00000050540	-2.35197328	1.54E-28
ENSSSCG00000040366	-2.353330213	1.43E-14
ENSSSCG00000033001	-2.354433021	2.76E-14
ENSSSCG00000026564	-2.35532097	0.001267341
ENSSSCG00000009699	-2.356370819	2.94E-24
ENSSSCG00000006357	-2.363662557	7.62E-08
ENSSSCG00000007022	-2.364038098	0.00145436
ENSSSCG00000041127	-2.36436022	0.000616733
ENSSSCG00000040288	-2.366328772	0.000143177
ENSSSCG00000036342	-2.371417322	7.57E-10
ENSSSCG00000014256	-2.372520623	3.53E-05
ENSSSCG00000041863	-2.37258877	0.00237515
ENSSSCG00000026129	-2.372725036	0.000371024
ENSSSCG00000000734	-2.373358072	6.90E-08
ENSSSCG00000017258	-2.374808464	6.01E-17
ENSSSCG00000005243	-2.377002252	7.46E-22
ENSSSCG00000008501	-2.377473645	0.000569604
ENSSSCG00000005194	-2.377829995	4.46E-09
ENSSSCG00000013785	-2.378095238	0.007018723
ENSSSCG00000009655	-2.378553356	3.90E-07
ENSSSCG00000030206	-2.380330537	0.000284246
ENSSSCG00000004255	-2.383984089	0.00215553
ENSSSCG00000039883	-2.386378068	6.17E-21
ENSSSCG00000014851	-2.389769969	2.13E-07
ENSSSCG00000005352	-2.390964199	5.60E-07
ENSSSCG00000005400	-2.391782036	5.86E-25
ENSSSCG00000000663	-2.395865932	6.13E-05
ENSSSCG00000008874	-2.395959522	6.28E-10
ENSSSCG00000042736	-2.405040567	1.99E-10
ENSSSCG00000007358	-2.407248352	2.94E-06
ENSSSCG00000033204	-2.409196632	3.79E-08
ENSSSCG00000008599	-2.409459296	2.06E-05
ENSSSCG00000007181	-2.409514925	2.02E-08
ENSSSCG00000048200	-2.411732389	0.003471765
ENSSSCG00000012071	-2.413833364	0.004057879

ENSSSCG00000012150	-2.417067713	7.33E-08
ENSSSCG00000022948	-2.418992264	8.58E-12
ENSSSCG00000036846	-2.420684351	3.16E-06
ENSSSCG00000022676	-2.422754053	1.40E-12
ENSSSCG00000015083	-2.42338125	8.91E-09
ENSSSCG00000045287	-2.42354457	2.08E-10
ENSSSCG00000038801	-2.425232136	2.67E-13
ENSSSCG00000042479	-2.425666104	7.89E-05
ENSSSCG00000039633	-2.431952568	1.18E-23
ENSSSCG00000009672	-2.432600275	7.99E-27
ENSSSCG00000006160	-2.432679131	1.08E-33
ENSSSCG00000042862	-2.432879669	4.97E-08
ENSSSCG00000027941	-2.438689455	3.94E-05
ENSSSCG00000036454	-2.443310771	5.74E-06
ENSSSCG00000038569	-2.44365168	1.53E-23
ENSSSCG00000031031	-2.443830544	0.00144377
ENSSSCG00000010926	-2.444568612	6.44E-24
ENSSSCG00000012561	-2.447450876	1.92E-10
ENSSSCG00000033760	-2.447973687	3.21E-15
ENSSSCG00000015563	-2.448461893	1.50E-24
ENSSSCG00000034827	-2.449027868	2.38E-31
ENSSSCG00000045440	-2.450227356	1.37E-06
ENSSSCG00000000385	-2.45321693	0.000488005
ENSSSCG00000009486	-2.455710688	0.002740692
ENSSSCG00000044435	-2.45677255	2.56E-07
ENSSSCG00000034351	-2.457069321	0.009017332
ENSSSCG00000013074	-2.46039467	4.25E-09
ENSSSCG00000008141	-2.465588242	4.36E-06
ENSSSCG00000032660	-2.466134218	2.61E-11
ENSSSCG00000033000	-2.468520431	1.66E-05
ENSSSCG00000046798	-2.470107539	0.010399049
ENSSSCG00000009222	-2.47081184	7.95E-10
ENSSSCG00000036013	-2.471974627	1.75E-17
ENSSSCG00000016639	-2.472089749	2.23E-20
ENSSSCG00000037682	-2.472274229	9.21E-18
ENSSSCG00000002029	-2.47790251	0.011609029
ENSSSCG00000004032	-2.484013504	0.003874728
ENSSSCG00000029275	-2.485577693	0.007094498
ENSSSCG00000010252	-2.488474127	0.004510367
ENSSSCG00000011973	-2.490016987	8.98E-09
ENSSSCG00000002777	-2.504507201	5.20E-06
ENSSSCG00000044663	-2.505461351	0.003915035
ENSSSCG00000001716	-2.507294403	7.54E-09
ENSSSCG00000008326	-2.515259513	0.012795308
ENSSSCG00000002444	-2.515921008	2.36E-12
ENSSSCG00000016290	-2.51999596	0.001198767
ENSSSCG00000013989	-2.523759563	0.000362613
ENSSSCG00000007823	-2.525378798	6.52E-08
ENSSSCG00000009283	-2.527540674	2.98E-10
ENSSSCG00000026254	-2.530426536	0.00046052
ENSSSCG00000001793	-2.530742737	1.67E-07
ENSSSCG00000011747	-2.532300913	1.15E-05
ENSSSCG00000044941	-2.53314547	0.005821462
ENSSSCG00000002464	-2.534188035	1.62E-07
ENSSSCG00000051482	-2.535382135	7.01E-20
ENSSSCG00000016642	-2.535857737	0.00026626
ENSSSCG00000004150	-2.536706794	0.000654709
ENSSSCG00000005781	-2.537704735	1.12E-07

ENSSSCG00000006035	-2.537867039	2.78E-11
ENSSSCG00000040397	-2.540528992	6.99E-19
ENSSSCG00000004990	-2.552055254	2.73E-06
ENSSSCG00000000748	-2.552918954	0.002366452
ENSSSCG00000014221	-2.554172524	3.09E-07
ENSSSCG00000005487	-2.554802551	9.49E-22
ENSSSCG00000022347	-2.554973928	5.23E-12
ENSSSCG00000032503	-2.555322552	1.02E-08
ENSSSCG00000040147	-2.558438375	0.005367156
ENSSSCG00000035276	-2.558774747	1.79E-05
ENSSSCG00000026453	-2.558878237	2.40E-08
ENSSSCG00000034506	-2.55903279	2.43E-06
ENSSSCG00000046575	-2.559152192	0.001092071
ENSSSCG00000046947	-2.569492534	1.08E-18
ENSSSCG00000032971	-2.571627156	0.000370979
ENSSSCG00000012409	-2.574312376	0.001464027
ENSSSCG00000021852	-2.575065435	0.001686094
ENSSSCG00000006143	-2.576708553	6.92E-19
ENSSSCG00000012144	-2.576735349	1.36E-06
ENSSSCG00000001989	-2.579031641	7.73E-07
ENSSSCG00000046730	-2.580165747	1.88E-15
ENSSSCG00000005106	-2.583401855	1.00E-21
ENSSSCG00000002887	-2.584807463	0.000341117
ENSSSCG00000041560	-2.585105753	9.35E-05
ENSSSCG00000035859	-2.58549431	3.52E-21
ENSSSCG00000016621	-2.587113358	0.003286416
ENSSSCG00000005584	-2.588476594	1.75E-06
ENSSSCG00000010647	-2.593115675	0.012657189
ENSSSCG00000043608	-2.593875596	0.00249178
ENSSSCG00000013100	-2.594126065	9.97E-26
ENSSSCG00000008606	-2.599833787	2.49E-10
ENSSSCG00000015445	-2.600607128	1.14E-09
ENSSSCG00000042395	-2.602748504	0.000205396
ENSSSCG00000012520	-2.605010414	3.89E-29
ENSSSCG00000028341	-2.610224167	1.82E-05
ENSSSCG00000033043	-2.611029782	5.53E-09
ENSSSCG00000039261	-2.612541645	0.000135915
ENSSSCG00000001920	-2.612805509	5.55E-05
ENSSSCG00000035937	-2.614067194	1.20E-05
ENSSSCG00000050474	-2.619038802	0.007743806
ENSSSCG00000042410	-2.623060138	0.002309753
ENSSSCG00000044472	-2.625071112	1.28E-29
ENSSSCG00000050221	-2.625102395	0.000236283
ENSSSCG00000009468	-2.625995762	3.74E-08
ENSSSCG00000015045	-2.629942451	1.53E-05
ENSSSCG00000014876	-2.630176345	4.05E-16
ENSSSCG00000016437	-2.632407654	1.01E-17
ENSSSCG00000016878	-2.633197123	5.24E-16
ENSSSCG00000046607	-2.635331868	1.17E-12
ENSSSCG00000014029	-2.639291247	1.67E-23
ENSSSCG00000014224	-2.640550043	7.37E-13
ENSSSCG00000022724	-2.644867337	3.00E-05
ENSSSCG00000016032	-2.646145745	7.60E-07
ENSSSCG00000007986	-2.647433643	3.94E-06
ENSSSCG00000002811	-2.649173128	0.005111973
ENSSSCG00000048634	-2.650274859	0.003555455
ENSSSCG00000015632	-2.650722966	0.007772288
ENSSSCG00000020967	-2.654050542	1.21E-09

ENSSSCG00000031336	-2.655521827	3.76E-12
ENSSSCG00000036984	-2.657156144	2.80E-05
ENSSSCG00000035595	-2.658693562	3.46E-19
ENSSSCG00000033101	-2.659231967	0.000447807
ENSSSCG00000022309	-2.67132379	5.21E-12
ENSSSCG00000042777	-2.671381491	0.008384538
ENSSSCG00000015802	-2.674274111	1.20E-08
ENSSSCG00000022569	-2.6753044	9.28E-10
ENSSSCG00000009975	-2.676477134	1.04E-09
ENSSSCG00000006735	-2.677593239	2.80E-11
ENSSSCG00000031531	-2.682952451	8.75E-10
ENSSSCG00000001203	-2.685381808	0.002463022
ENSSSCG00000045352	-2.686194819	2.17E-07
ENSSSCG00000049061	-2.688232649	0.003865843
ENSSSCG00000050338	-2.690093282	0.002683349
ENSSSCG00000040490	-2.693619724	7.22E-17
ENSSSCG00000008240	-2.698735864	1.25E-11
ENSSSCG00000003687	-2.699967543	3.42E-38
ENSSSCG00000004907	-2.701197148	3.41E-14
ENSSSCG00000003811	-2.702808631	9.63E-11
ENSSSCG00000004218	-2.703481665	5.09E-06
ENSSSCG00000012525	-2.71065398	6.33E-12
ENSSSCG00000030565	-2.712557558	0.00033504
ENSSSCG00000026402	-2.71298683	0.006621351
ENSSSCG00000029281	-2.714282449	1.93E-49
ENSSSCG00000051156	-2.722295964	0.000140145
ENSSSCG00000007574	-2.722640247	3.45E-11
ENSSSCG00000003760	-2.723599224	3.13E-12
ENSSSCG00000040607	-2.726951971	4.67E-35
ENSSSCG00000035507	-2.731952691	3.55E-14
ENSSSCG00000000915	-2.732016292	1.22E-10
ENSSSCG00000007435	-2.732925965	4.45E-14
ENSSSCG00000033363	-2.735655357	2.54E-05
ENSSSCG00000040557	-2.737554336	1.80E-15
ENSSSCG00000005249	-2.737745692	6.13E-14
ENSSSCG00000002314	-2.740053379	3.62E-11
ENSSSCG00000024351	-2.741259051	1.07E-31
ENSSSCG00000011186	-2.74194223	1.77E-07
ENSSSCG00000036630	-2.742187178	5.02E-05
ENSSSCG00000027022	-2.748435536	2.91E-06
ENSSSCG00000050566	-2.750398315	4.99E-14
ENSSSCG00000004520	-2.75652067	9.88E-07
ENSSSCG00000001068	-2.757595905	6.32E-05
ENSSSCG00000000802	-2.761533259	2.33E-11
ENSSSCG00000017462	-2.761759399	0.003477041
ENSSSCG00000026387	-2.763394819	6.59E-13
ENSSSCG00000017738	-2.767292341	2.54E-14
ENSSSCG00000038508	-2.767351981	2.25E-14
ENSSSCG00000043115	-2.770151414	4.54E-07
ENSSSCG00000022202	-2.772253324	7.16E-12
ENSSSCG00000004093	-2.772954532	0.000488551
ENSSSCG00000007849	-2.775147962	2.54E-07
ENSSSCG00000012680	-2.777031266	1.13E-12
ENSSSCG00000000029	-2.77717182	2.52E-11
ENSSSCG00000013618	-2.777403345	2.55E-07
ENSSSCG00000012572	-2.777784542	7.21E-10
ENSSSCG00000001752	-2.786952206	6.89E-07
ENSSSCG00000004675	-2.794854022	0.000455768

ENSSSCG00000012135	-2.806664828	5.89E-07
ENSSSCG00000040829	-2.807958363	0.002736226
ENSSSCG00000044206	-2.808085342	0.005201727
ENSSSCG00000007453	-2.80842042	0.000277599
ENSSSCG00000009134	-2.809483274	1.80E-20
ENSSSCG00000042401	-2.813054601	1.33E-05
ENSSSCG00000031590	-2.813571632	0.00119209
ENSSSCG00000024191	-2.814406384	0.003531311
ENSSSCG00000009666	-2.818322849	1.90E-24
ENSSSCG00000011349	-2.821937925	0.00616812
ENSSSCG00000005502	-2.833732367	1.70E-13
ENSSSCG00000047269	-2.839528011	0.004185724
ENSSSCG00000010530	-2.841093934	1.92E-07
ENSSSCG00000006788	-2.841649567	5.47E-19
ENSSSCG00000011208	-2.84481318	3.05E-07
ENSSSCG00000006875	-2.846314716	1.35E-05
ENSSSCG00000028488	-2.848583964	4.32E-08
ENSSSCG00000012637	-2.849196946	6.04E-15
ENSSSCG00000025717	-2.850499292	0.00026451
ENSSSCG00000036516	-2.850677957	9.40E-07
ENSSSCG00000014053	-2.85310484	7.70E-06
ENSSSCG00000002315	-2.854140029	0.000477465
ENSSSCG00000046901	-2.858349567	0.000150119
ENSSSCG00000042722	-2.861430984	0.000649601
ENSSSCG00000016823	-2.863623453	0.00566324
ENSSSCG00000031465	-2.870471902	6.40E-06
ENSSSCG00000016892	-2.870560878	1.19E-09
ENSSSCG00000000916	-2.870578941	0.003887727
ENSSSCG00000010222	-2.871370522	0.005837758
ENSSSCG00000002450	-2.872148145	5.65E-08
ENSSSCG00000003951	-2.874529323	0.002470797
ENSSSCG00000011713	-2.874623916	4.99E-08
ENSSSCG00000020941	-2.875005797	3.14E-10
ENSSSCG00000015037	-2.87846228	4.22E-40
ENSSSCG00000034469	-2.878897292	1.61E-33
ENSSSCG00000039874	-2.883881293	2.22E-43
ENSSSCG00000008397	-2.886866036	9.43E-16
ENSSSCG00000002718	-2.891704355	0.00165163
ENSSSCG00000011802	-2.892060975	0.002334141
ENSSSCG00000011717	-2.892472614	2.31E-10
ENSSSCG00000007170	-2.89348102	6.51E-27
ENSSSCG00000003699	-2.893493521	1.44E-21
ENSSSCG00000021208	-2.895351255	5.70E-16
ENSSSCG00000027160	-2.900367044	0.000169826
ENSSSCG00000026259	-2.90080705	0.002946096
ENSSSCG00000032403	-2.901230809	3.87E-10
ENSSSCG00000039215	-2.906781667	0.000128251
ENSSSCG00000032480	-2.910312516	6.40E-16
ENSSSCG00000011522	-2.91050566	6.93E-06
ENSSSCG00000050458	-2.910858178	1.25E-09
ENSSSCG00000008769	-2.912500066	4.42E-11
ENSSSCG00000004013	-2.91756438	5.79E-11
ENSSSCG00000045763	-2.921362453	1.24E-05
ENSSSCG00000000515	-2.924293365	0.001055905
ENSSSCG00000016034	-2.925141926	9.46E-11
ENSSSCG00000040080	-2.932569801	7.75E-18
ENSSSCG00000038685	-2.933529859	6.56E-09
ENSSSCG00000010826	-2.936210905	1.81E-20

ENSSSCG00000036589	-2.946064088	6.14E-11
ENSSSCG00000014812	-2.948070576	1.05E-19
ENSSSCG00000030655	-2.951563697	1.01E-09
ENSSSCG00000049223	-2.951716507	0.000321325
ENSSSCG00000049042	-2.954738015	0.001512578
ENSSSCG00000000418	-2.956041259	1.34E-11
ENSSSCG00000045021	-2.961972738	0.000486417
ENSSSCG00000023871	-2.963661143	0.002188879
ENSSSCG00000020988	-2.966188677	7.26E-19
ENSSSCG00000049733	-2.966609919	0.001956779
ENSSSCG00000025783	-2.972195572	6.18E-07
ENSSSCG00000036177	-2.977426147	1.55E-14
ENSSSCG00000000660	-2.978288637	4.79E-14
ENSSSCG00000023710	-2.978430271	1.27E-14
ENSSSCG00000009361	-2.979142842	2.04E-29
ENSSSCG00000040767	-2.979759572	0.005348494
ENSSSCG00000023328	-2.98323918	1.59E-24
ENSSSCG00000015425	-2.987973219	0.008175139
ENSSSCG00000026211	-2.994873896	4.73E-05
ENSSSCG00000025672	-2.996690219	4.76E-09
ENSSSCG00000022445	-2.996950776	0.001993483
ENSSSCG00000004201	-2.997859404	6.93E-10
ENSSSCG00000041957	-3.000535861	0.004190224
ENSSSCG00000048047	-3.001219765	0.002365705
ENSSSCG00000013378	-3.012504877	0.000152441
ENSSSCG00000031831	-3.019653465	1.86E-19
ENSSSCG00000031858	-3.025945125	1.89E-14
ENSSSCG00000034660	-3.027356138	1.12E-24
ENSSSCG00000021514	-3.031420439	3.26E-14
ENSSSCG00000013433	-3.031996447	3.04E-08
ENSSSCG00000046459	-3.033744468	0.003666883
ENSSSCG00000005785	-3.038622159	2.36E-08
ENSSSCG00000024960	-3.039035145	4.90E-43
ENSSSCG00000016033	-3.044689512	1.08E-10
ENSSSCG00000023514	-3.051346353	1.53E-07
ENSSSCG00000003479	-3.062964501	5.40E-14
ENSSSCG00000043490	-3.066555306	2.36E-07
ENSSSCG00000010429	-3.068849401	1.48E-12
ENSSSCG00000044254	-3.069537095	0.000806328
ENSSSCG00000038452	-3.072149201	5.30E-14
ENSSSCG00000047491	-3.072195316	8.85E-05
ENSSSCG00000015893	-3.07678614	0.000617136
ENSSSCG00000000531	-3.077605291	1.54E-10
ENSSSCG00000006969	-3.081765196	8.80E-09
ENSSSCG00000016618	-3.084179855	1.02E-22
ENSSSCG00000025708	-3.092508081	0.01085473
ENSSSCG00000043719	-3.093305705	0.000681266
ENSSSCG00000048326	-3.095865977	3.84E-10
ENSSSCG00000038824	-3.09739202	2.24E-18
ENSSSCG00000005699	-3.111035826	2.38E-27
ENSSSCG00000050177	-3.116403748	2.13E-17
ENSSSCG00000009045	-3.124430842	1.62E-20
ENSSSCG00000041263	-3.130135049	0.00082601
ENSSSCG00000004678	-3.130784602	5.00E-14
ENSSSCG00000037360	-3.134955531	5.89E-27
ENSSSCG00000024685	-3.154923369	4.54E-07
ENSSSCG00000040306	-3.157549736	3.00E-07
ENSSSCG00000003784	-3.157989633	0.004268399

ENSSSCG00000036134	-3.160785302	0.010168401
ENSSSCG00000022105	-3.171969687	2.49E-15
ENSSSCG00000012521	-3.17402082	5.53E-22
ENSSSCG00000050742	-3.174779118	1.14E-24
ENSSSCG00000010948	-3.179045245	2.08E-24
ENSSSCG00000049418	-3.179709721	1.98E-06
ENSSSCG00000007644	-3.185870719	9.68E-11
ENSSSCG00000007678	-3.188658918	1.01E-07
ENSSSCG00000029371	-3.190834725	1.10E-12
ENSSSCG00000011286	-3.191353412	0.000228471
ENSSSCG00000036011	-3.193083736	4.94E-06
ENSSSCG00000040795	-3.195725945	5.78E-09
ENSSSCG00000027852	-3.201342643	5.44E-08
ENSSSCG00000035736	-3.205544886	1.23E-10
ENSSSCG00000031090	-3.207071207	4.28E-05
ENSSSCG00000041112	-3.20749003	0.003086433
ENSSSCG00000002835	-3.21146832	0.00137091
ENSSSCG00000023322	-3.213838825	1.05E-08
ENSSSCG00000049768	-3.214516925	0.00700775
ENSSSCG00000010210	-3.2198559	1.51E-06
ENSSSCG00000034335	-3.222886942	8.76E-10
ENSSSCG00000004835	-3.228485792	1.58E-06
ENSSSCG00000042706	-3.235156212	1.22E-08
ENSSSCG00000007079	-3.240476947	4.28E-06
ENSSSCG00000016130	-3.240886402	2.87E-06
ENSSSCG00000048660	-3.241419053	9.07E-09
ENSSSCG00000015732	-3.241464455	1.05E-09
ENSSSCG00000039358	-3.241576442	0.003111773
ENSSSCG00000025271	-3.247208663	1.59E-22
ENSSSCG00000032151	-3.253879486	3.57E-12
ENSSSCG00000008842	-3.257945007	1.88E-49
ENSSSCG00000000854	-3.271779827	2.05E-45
ENSSSCG00000009610	-3.279283722	3.48E-07
ENSSSCG00000011851	-3.279823082	0.001235934
ENSSSCG00000010377	-3.286376291	0.007010599
ENSSSCG00000032659	-3.288667508	0.006051413
ENSSSCG00000037432	-3.3016632	1.53E-11
ENSSSCG00000036499	-3.310774106	6.76E-11
ENSSSCG00000035058	-3.316416782	5.13E-37
ENSSSCG00000004143	-3.33085298	3.81E-13
ENSSSCG00000043663	-3.334962206	0.000299368
ENSSSCG00000042267	-3.340562218	6.50E-26
ENSSSCG00000016915	-3.344106652	1.35E-09
ENSSSCG00000034284	-3.3644868	1.57E-17
ENSSSCG00000047976	-3.366074295	4.04E-07
ENSSSCG00000011217	-3.36746738	0.004347562
ENSSSCG00000017956	-3.367833084	1.60E-31
ENSSSCG00000034987	-3.371087233	4.95E-05
ENSSSCG00000009526	-3.376128269	2.48E-07
ENSSSCG00000001942	-3.384952791	0.000450478
ENSSSCG00000025240	-3.38790241	1.81E-19
ENSSSCG00000003773	-3.38916564	4.17E-26
ENSSSCG00000034993	-3.389338574	1.39E-17
ENSSSCG00000009601	-3.3946918	2.84E-27
ENSSSCG00000036943	-3.396045573	1.04E-08
ENSSSCG00000049831	-3.39818281	3.88E-10
ENSSSCG00000009659	-3.398729395	7.73E-06
ENSSSCG00000015399	-3.40614217	2.17E-10

ENSSSCG00000031712	-3.418141889	7.86E-08
ENSSSCG00000011625	-3.41865457	2.35E-07
ENSSSCG00000011046	-3.421456664	0.000139325
ENSSSCG00000004939	-3.422159402	1.30E-18
ENSSSCG00000047343	-3.426618335	3.20E-10
ENSSSCG00000035124	-3.429982945	0.000248762
ENSSSCG00000007799	-3.438142104	0.006903182
ENSSSCG00000016857	-3.448348524	1.55E-75
ENSSSCG00000036378	-3.454939115	0.003473542
ENSSSCG00000016365	-3.455943801	1.75E-31
ENSSSCG00000044584	-3.458381082	3.70E-07
ENSSSCG00000035043	-3.465599885	8.04E-06
ENSSSCG00000014259	-3.469840155	0.000145325
ENSSSCG00000049139	-3.487967741	0.003910464
ENSSSCG00000009098	-3.489884866	0.004082818
ENSSSCG00000023550	-3.49014385	0.000758555
ENSSSCG00000001751	-3.495146824	3.99E-05
ENSSSCG00000025072	-3.496399737	0.004401315
ENSSSCG00000028741	-3.498140748	7.86E-12
ENSSSCG00000036461	-3.50450873	1.55E-06
ENSSSCG00000043748	-3.511698161	0.010071774
ENSSSCG00000008101	-3.517968387	9.52E-08
ENSSSCG00000048179	-3.524621832	6.62E-23
ENSSSCG00000014840	-3.535138346	2.44E-05
ENSSSCG00000013044	-3.53900865	0.000387319
ENSSSCG00000041163	-3.539776802	3.75E-21
ENSSSCG00000027903	-3.552332134	7.36E-12
ENSSSCG00000038906	-3.552360939	0.012172051
ENSSSCG00000039306	-3.554634688	2.92E-05
ENSSSCG00000010481	-3.555212015	1.08E-09
ENSSSCG00000009646	-3.565872035	3.33E-13
ENSSSCG00000013788	-3.566999074	1.17E-37
ENSSSCG00000003736	-3.569903903	0.000403186
ENSSSCG00000009748	-3.56999792	0.000515607
ENSSSCG00000014725	-3.572666737	7.07E-15
ENSSSCG00000023225	-3.573434313	4.80E-07
ENSSSCG00000026079	-3.591137132	1.06E-13
ENSSSCG00000026130	-3.595792091	2.69E-06
ENSSSCG00000013148	-3.596478015	4.32E-07
ENSSSCG00000037292	-3.610095331	3.09E-07
ENSSSCG00000041245	-3.610365751	2.21E-13
ENSSSCG00000010444	-3.614739335	2.51E-06
ENSSSCG00000016599	-3.614829588	5.33E-10
ENSSSCG00000047771	-3.616318836	6.93E-08
ENSSSCG00000032423	-3.617890192	1.73E-30
ENSSSCG00000042123	-3.618968457	0.007018917
ENSSSCG00000006138	-3.633050443	0.001528371
ENSSSCG00000017446	-3.634847767	7.98E-24
ENSSSCG00000047852	-3.634930338	0.001007185
ENSSSCG00000012571	-3.640481587	4.69E-17
ENSSSCG00000036452	-3.646957007	1.48E-20
ENSSSCG00000050979	-3.651803269	1.22E-07
ENSSSCG00000008166	-3.656489528	0.001292052
ENSSSCG00000051434	-3.65878336	0.008672856
ENSSSCG00000047296	-3.665779959	5.06E-09
ENSSSCG00000051145	-3.669514271	0.000193633
ENSSSCG00000047792	-3.67514772	0.000107172
ENSSSCG00000015353	-3.676604907	4.20E-39

ENSSSCG00000045670	-3.687084923	2.38E-21
ENSSSCG00000041646	-3.695159139	1.93E-06
ENSSSCG00000015426	-3.69602181	7.46E-46
ENSSSCG00000041964	-3.697572587	8.30E-05
ENSSSCG00000011596	-3.697955667	0.002764238
ENSSSCG00000023936	-3.703238207	0.000609906
ENSSSCG00000001863	-3.707623505	4.88E-05
ENSSSCG00000025777	-3.709488556	4.17E-13
ENSSSCG00000013934	-3.711087262	0.00018489
ENSSSCG00000036036	-3.712964424	2.91E-10
ENSSSCG00000027553	-3.716123348	0.009299061
ENSSSCG00000016423	-3.719304256	1.84E-07
ENSSSCG00000044870	-3.72408003	7.33E-07
ENSSSCG00000023806	-3.726302189	2.54E-10
ENSSSCG00000006758	-3.72920306	8.90E-06
ENSSSCG00000050025	-3.735257143	0.001792299
ENSSSCG00000040824	-3.736694725	2.07E-09
ENSSSCG00000051654	-3.737409898	0.009502169
ENSSSCG00000040123	-3.740340627	0.007015505
ENSSSCG00000008634	-3.743169599	1.71E-09
ENSSSCG00000042523	-3.751775713	1.89E-06
ENSSSCG00000009239	-3.756264306	1.56E-07
ENSSSCG00000040016	-3.768266977	1.15E-16
ENSSSCG00000009202	-3.774093617	1.12E-29
ENSSSCG00000041262	-3.781654019	3.03E-05
ENSSSCG00000015914	-3.784303241	2.68E-13
ENSSSCG00000046403	-3.792019811	4.03E-07
ENSSSCG00000025993	-3.799816658	7.15E-13
ENSSSCG00000002779	-3.800391009	1.58E-05
ENSSSCG00000051274	-3.802322488	4.07E-17
ENSSSCG00000036626	-3.812258631	9.46E-18
ENSSSCG00000002032	-3.812867491	6.75E-24
ENSSSCG00000004713	-3.838400234	3.87E-11
ENSSSCG00000006455	-3.839851682	1.84E-05
ENSSSCG00000016941	-3.841903246	1.28E-11
ENSSSCG00000042913	-3.843061028	2.65E-05
ENSSSCG00000010376	-3.851613975	0.000335832
ENSSSCG00000049232	-3.857461521	0.000167292
ENSSSCG00000005512	-3.865871224	5.65E-09
ENSSSCG00000046678	-3.877432843	9.52E-05
ENSSSCG00000021075	-3.881157254	1.04E-08
ENSSSCG00000004891	-3.891603723	0.003160636
ENSSSCG00000029260	-3.91043505	4.64E-16
ENSSSCG00000029756	-3.912112622	0.003142537
ENSSSCG00000014316	-3.912887031	4.05E-06
ENSSSCG00000032094	-3.913831415	3.41E-11
ENSSSCG00000001500	-3.940046664	0.00057314
ENSSSCG00000044207	-3.954737509	0.000252689
ENSSSCG00000046026	-3.956317687	1.75E-06
ENSSSCG00000011756	-3.962882262	9.31E-10
ENSSSCG00000034722	-3.978892633	3.01E-52
ENSSSCG00000033542	-3.984959607	1.30E-18
ENSSSCG00000004052	-3.99230728	6.21E-24
ENSSSCG00000028277	-3.995485195	2.58E-12
ENSSSCG00000024293	-4.008943586	2.62E-13
ENSSSCG00000013320	-4.014718417	0.000124843
ENSSSCG00000003777	-4.027303237	0.001826536
ENSSSCG00000051284	-4.034199481	0.012018083

ENSSSCG00000007146	-4.035694963	1.40E-23
ENSSSCG00000012238	-4.046710228	4.64E-40
ENSSSCG00000009144	-4.052869704	0.004826735
ENSSSCG00000027157	-4.056610988	1.37E-25
ENSSSCG00000003366	-4.062152244	2.02E-13
ENSSSCG00000045323	-4.086707549	0.010146766
ENSSSCG00000051645	-4.090756616	0.010566417
ENSSSCG00000045259	-4.096714204	0.010071505
ENSSSCG00000015873	-4.101469754	0.010188998
ENSSSCG00000016270	-4.14446302	0.001764245
ENSSSCG00000046645	-4.14760766	0.001549314
ENSSSCG00000050086	-4.154873273	0.000190668
ENSSSCG00000035651	-4.158784413	1.19E-17
ENSSSCG00000012548	-4.164637248	8.83E-05
ENSSSCG00000015746	-4.179615887	2.07E-07
ENSSSCG00000005296	-4.191972288	0.001660567
ENSSSCG00000031640	-4.195372467	1.36E-18
ENSSSCG00000014441	-4.197864635	2.37E-22
ENSSSCG00000049640	-4.202705631	1.12E-07
ENSSSCG00000050073	-4.209425328	4.64E-08
ENSSSCG00000031648	-4.209898409	0.00802654
ENSSSCG00000030706	-4.23339287	2.52E-15
ENSSSCG00000009754	-4.240388437	0.008032267
ENSSSCG00000048861	-4.24691861	2.40E-06
ENSSSCG00000035066	-4.288737587	1.03E-11
ENSSSCG00000041079	-4.289794207	9.93E-07
ENSSSCG00000010348	-4.323334268	0.001154043
ENSSSCG00000004903	-4.325725486	0.005083876
ENSSSCG00000046732	-4.344762899	3.68E-23
ENSSSCG00000015830	-4.350970761	0.00048981
ENSSSCG00000050412	-4.363559593	0.000599065
ENSSSCG00000015548	-4.366004596	3.40E-09
ENSSSCG00000000791	-4.368801129	2.42E-11
ENSSSCG00000048836	-4.382070081	0.000488914
ENSSSCG00000000252	-4.384796449	0.001633245
ENSSSCG00000044457	-4.407359703	0.004614073
ENSSSCG00000050971	-4.42663389	0.000353991
ENSSSCG00000038699	-4.428713214	0.000413083
ENSSSCG00000012517	-4.435562657	6.82E-30
ENSSSCG00000005131	-4.436105557	0.003497809
ENSSSCG00000021651	-4.438425145	1.06E-25
ENSSSCG00000000789	-4.447194222	0.000339851
ENSSSCG00000045433	-4.476961578	1.10E-37
ENSSSCG00000048852	-4.488742633	0.003082252
ENSSSCG00000035189	-4.492795785	6.97E-39
ENSSSCG00000033282	-4.507879668	0.000392446
ENSSSCG00000047456	-4.519687298	0.000507085
ENSSSCG00000047414	-4.529081018	8.42E-07
ENSSSCG00000018028	-4.533683898	0.00281977
ENSSSCG00000039940	-4.561001704	4.05E-07
ENSSSCG00000011069	-4.599627051	4.69E-07
ENSSSCG00000039813	-4.613779373	3.42E-16
ENSSSCG00000017109	-4.616961908	0.002351779
ENSSSCG00000017003	-4.643625072	5.54E-16
ENSSSCG00000036475	-4.646799743	3.40E-15
ENSSSCG00000042661	-4.650836398	0.000138216
ENSSSCG00000016093	-4.664340232	3.68E-06
ENSSSCG00000015711	-4.68442441	0.000119872

ENSSSCG00000038578	-4.694797573	0.010424381
ENSSSCG00000036856	-4.702756916	5.35E-09
ENSSSCG00000043383	-4.703576088	0.009457981
ENSSSCG00000002817	-4.708436157	4.84E-09
ENSSSCG00000037794	-4.719347433	0.001316665
ENSSSCG00000016064	-4.721935095	1.93E-10
ENSSSCG00000006979	-4.730199597	3.53E-28
ENSSSCG00000013358	-4.751536248	0.007420664
ENSSSCG00000049143	-4.751637129	7.08E-19
ENSSSCG00000045143	-4.765115615	0.007169814
ENSSSCG00000009842	-4.777747461	1.63E-06
ENSSSCG00000003891	-4.780408392	0.007042814
ENSSSCG00000041359	-4.841960539	0.000875634
ENSSSCG00000035798	-4.869389161	1.46E-11
ENSSSCG00000006064	-4.876092804	6.45E-05
ENSSSCG00000033413	-4.92756292	0.004716444
ENSSSCG00000047755	-4.932338519	0.005620184
ENSSSCG00000039272	-4.953406546	2.63E-10
ENSSSCG00000032019	-4.965568399	4.07E-22
ENSSSCG00000012638	-4.979063641	5.92E-16
ENSSSCG00000046270	-4.991084819	0.008882967
ENSSSCG00000002474	-4.993639171	0.003791359
ENSSSCG00000005177	-4.997901644	0.001005793
ENSSSCG00000003523	-5.029274212	0.00350945
ENSSSCG00000036833	-5.044914749	0.002854995
ENSSSCG00000040009	-5.050063274	4.20E-42
ENSSSCG00000011640	-5.051487593	0.000520558
ENSSSCG00000008770	-5.081751226	0.000383741
ENSSSCG00000041885	-5.101891142	0.002310052
ENSSSCG00000015406	-5.101974604	8.21E-16
ENSSSCG00000039685	-5.106414014	0.000122854
ENSSSCG00000004250	-5.112465907	2.68E-05
ENSSSCG00000010660	-5.129528718	0.012490737
ENSSSCG00000049731	-5.137801893	0.002207849
ENSSSCG00000028922	-5.140510444	2.24E-12
ENSSSCG00000037254	-5.145883964	2.39E-47
ENSSSCG00000029441	-5.15806209	0.00737856
ENSSSCG00000012551	-5.166763312	2.69E-07
ENSSSCG00000038706	-5.214952934	1.08E-54
ENSSSCG00000044946	-5.220742234	4.02E-07
ENSSSCG00000016676	-5.226335692	1.11E-05
ENSSSCG00000006264	-5.23062439	0.001376922
ENSSSCG00000042268	-5.249457644	0.000240814
ENSSSCG00000034946	-5.254047987	0.000173085
ENSSSCG00000031258	-5.268884888	5.73E-08
ENSSSCG00000032431	-5.276621043	8.05E-06
ENSSSCG00000003524	-5.314553331	4.06E-41
ENSSSCG00000012121	-5.314752223	7.79E-06
ENSSSCG00000007505	-5.343591623	0.000180988
ENSSSCG00000049555	-5.344698373	5.90E-06
ENSSSCG00000049571	-5.38119937	0.001528759
ENSSSCG00000046533	-5.385142472	0.000754082
ENSSSCG00000000659	-5.393055435	1.78E-45
ENSSSCG00000042998	-5.394620685	3.96E-06
ENSSSCG00000009856	-5.403834005	0.000618961
ENSSSCG00000033512	-5.407417402	1.29E-52
ENSSSCG00000028927	-5.411733683	9.34E-05
ENSSSCG00000048570	-5.412363388	3.62E-06

ENSSSCG00000039961	-5.418929516	1.80E-12
ENSSSCG00000011038	-5.421954839	1.08E-31
ENSSSCG00000041272	-5.426528014	9.01E-05
ENSSSCG00000042154	-5.460654032	0.009392493
ENSSSCG00000050630	-5.463019851	1.21E-05
ENSSSCG00000043322	-5.469346891	7.80E-05
ENSSSCG00000031352	-5.475545242	1.90E-06
ENSSSCG00000006106	-5.482247442	0.000624723
ENSSSCG00000048807	-5.493969014	0.000313456
ENSSSCG00000043581	-5.505186063	2.22E-06
ENSSSCG00000010304	-5.530266432	0.004410146
ENSSSCG00000012371	-5.534861065	1.40E-06
ENSSSCG00000016305	-5.545761005	0.000387332
ENSSSCG00000007083	-5.569024215	1.98E-31
ENSSSCG00000042754	-5.574490057	0.000378146
ENSSSCG00000040728	-5.651299199	2.75E-25
ENSSSCG00000003123	-5.661811788	2.92E-05
ENSSSCG00000042225	-5.688312144	0.000176634
ENSSSCG00000043103	-5.747336098	2.49E-05
ENSSSCG00000044752	-5.758809983	0.000122016
ENSSSCG00000032702	-5.801468728	2.44E-12
ENSSSCG00000011060	-5.831044943	1.38E-05
ENSSSCG00000040042	-5.840045841	1.17E-33
ENSSSCG00000048892	-5.85713359	1.15E-12
ENSSSCG00000001746	-5.873089176	1.58E-05
ENSSSCG00000042920	-5.910049636	0.000105976
ENSSSCG00000001085	-5.913287999	6.08E-05
ENSSSCG00000017062	-5.915292991	1.55E-118
ENSSSCG00000002781	-5.957855782	4.74E-05
ENSSSCG00000016869	-5.980229549	6.83E-06
ENSSSCG00000007185	-6.050445887	6.72E-37
ENSSSCG00000048012	-6.077342829	0.000261803
ENSSSCG00000039445	-6.091279828	5.16E-06
ENSSSCG00000009444	-6.253963176	1.75E-08
ENSSSCG00000040715	-6.262563861	1.94E-06
ENSSSCG00000032923	-6.373846826	1.12E-05
ENSSSCG00000006450	-6.378811176	1.14E-31
ENSSSCG00000012363	-6.385415592	1.28E-24
ENSSSCG00000013869	-6.387937152	7.84E-07
ENSSSCG00000015969	-6.411610465	2.41E-14
ENSSSCG00000032896	-6.460281371	1.02E-06
ENSSSCG00000045337	-6.464928907	1.17E-08
ENSSSCG00000037430	-6.515870535	2.06E-78
ENSSSCG00000033100	-6.543561287	3.53E-09
ENSSSCG00000023125	-6.573038727	1.05E-20
ENSSSCG00000016129	-6.573075993	3.52E-45
ENSSSCG00000035170	-6.607046909	1.11E-50
ENSSSCG00000046005	-6.75514038	6.49E-07
ENSSSCG00000025294	-6.823127504	4.16E-07
ENSSSCG00000015716	-6.828647842	5.06E-98
ENSSSCG00000048970	-6.858247427	4.49E-07
ENSSSCG00000013469	-6.867767562	4.31E-07
ENSSSCG00000049512	-6.903416967	3.26E-07
ENSSSCG00000023746	-6.908086239	2.69E-07
ENSSSCG00000000856	-6.959721988	1.90E-07
ENSSSCG00000046275	-6.978744493	1.20E-06
ENSSSCG00000020986	-7.011066223	7.05E-07
ENSSSCG00000051495	-7.068497356	9.50E-08

ENSSSCG00000040836	-7.072196288	1.36E-07
ENSSSCG00000040088	-7.122191187	2.37E-07
ENSSSCG00000017037	-7.135592306	1.67E-10
ENSSSCG00000033157	-7.199799625	4.37E-08
ENSSSCG00000048622	-7.277229449	3.29E-08
ENSSSCG00000020694	-7.385424235	1.58E-08
ENSSSCG00000011178	-7.469992448	9.28E-09
ENSSSCG00000013579	-7.554955027	1.45E-11
ENSSSCG00000050830	-7.61582904	4.54E-09
ENSSSCG00000041022	-7.628347773	2.93E-09
ENSSSCG00000038557	-7.784420817	6.04E-09
ENSSSCG00000031220	-7.969197302	1.77E-09
ENSSSCG00000012065	-8.133637106	3.08E-11
ENSSSCG00000049770	-8.148605362	5.90E-11
ENSSSCG00000015662	-8.225398851	3.09E-103
ENSSSCG00000018080	-8.584314505	1.64E-91
ENSSSCG00000032123	-8.63340369	1.47E-11
ENSSSCG00000008524	-8.944435753	5.48E-13
ENSSSCG00000051343	-9.019310896	6.93E-13
ENSSSCG00000046878	-9.585469995	1.55E-14
ENSSSCG00000010190	-9.790518413	0.002091896
ENSSSCG00000022701	-11.52810174	5.32E-131

Supplementary Table 5. The common upregulated differentially expressed genes from spleen and inguinal lymph node samples between the infected and mock group

GeneID
ENSSSCG00000038359
ENSSSCG00000011410
ENSSSCG00000031388
ENSSSCG00000006853
ENSSSCG00000021472
ENSSSCG00000024126
ENSSSCG00000010320
ENSSSCG00000044986
ENSSSCG00000004164
ENSSSCG00000010261
ENSSSCG00000029438
ENSSSCG00000034119
ENSSSCG00000045298
ENSSSCG00000014371
ENSSSCG00000029538
ENSSSCG00000035596
ENSSSCG00000030369
ENSSSCG00000006588
ENSSSCG00000033860
ENSSSCG00000010575
ENSSSCG00000000694
ENSSSCG00000039416
ENSSSCG00000010540
ENSSSCG00000036064
ENSSSCG00000000133
ENSSSCG00000016817
ENSSSCG00000013475
ENSSSCG00000014168
ENSSSCG00000004480
ENSSSCG00000013896
ENSSSCG00000030996
ENSSSCG00000008261
ENSSSCG00000037465
ENSSSCG00000047395
ENSSSCG00000013721
ENSSSCG00000026761
ENSSSCG00000028465
ENSSSCG00000009622
ENSSSCG00000033374
ENSSSCG00000015769
ENSSSCG00000006796
ENSSSCG00000038489
ENSSSCG00000013746
ENSSSCG00000000194
ENSSSCG00000016744
ENSSSCG00000009145
ENSSSCG00000002768
ENSSSCG00000023041
ENSSSCG00000023684
ENSSSCG00000003552
ENSSSCG00000035347
ENSSSCG00000022380
ENSSSCG00000007896
ENSSSCG00000011680
ENSSSCG00000012534
ENSSSCG00000009964
ENSSSCG00000016983

ENSSSCG00000037642
ENSSSCG00000017971
ENSSSCG00000008976
ENSSSCG00000006378
ENSSSCG00000036709
ENSSSCG00000021411
ENSSSCG00000001990
ENSSSCG00000021815
ENSSSCG00000014850
ENSSSCG00000000683
ENSSSCG00000031991
ENSSSCG00000027526
ENSSSCG00000037929
ENSSSCG00000008550
ENSSSCG00000034012
ENSSSCG00000050193
ENSSSCG00000017379
ENSSSCG00000016903
ENSSSCG00000004908
ENSSSCG00000008938
ENSSSCG00000040990
ENSSSCG00000038562
ENSSSCG00000028019
ENSSSCG00000023482
ENSSSCG00000015794
ENSSSCG00000017471
ENSSSCG00000014304
ENSSSCG00000003763
ENSSSCG00000004125
ENSSSCG00000005486
ENSSSCG00000030108
ENSSSCG00000009469
ENSSSCG00000037606
ENSSSCG00000048903
ENSSSCG00000011673
ENSSSCG00000012280
ENSSSCG00000031789
ENSSSCG00000003943
ENSSSCG00000030548
ENSSSCG00000021610
ENSSSCG00000030255
ENSSSCG00000038700
ENSSSCG00000040294
ENSSSCG00000010330
ENSSSCG00000016220
ENSSSCG00000006831
ENSSSCG00000043494
ENSSSCG00000006729
ENSSSCG00000032996
ENSSSCG00000010576
ENSSSCG00000016041
ENSSSCG00000049674
ENSSSCG00000010452
ENSSSCG00000017591
ENSSSCG00000016077
ENSSSCG00000005601
ENSSSCG00000014600
ENSSSCG00000010451

ENSSSCG00000021828
ENSSSCG00000016609
ENSSSCG00000012378
ENSSSCG00000031860
ENSSSCG00000008823
ENSSSCG00000011798
ENSSSCG00000011714
ENSSSCG00000028964
ENSSSCG00000038914
ENSSSCG00000009334
ENSSSCG00000009532
ENSSSCG00000014040
ENSSSCG00000003419
ENSSSCG00000039862
ENSSSCG00000003189
ENSSSCG00000007861
ENSSSCG00000009100
ENSSSCG00000030578
ENSSSCG00000016374
ENSSSCG00000010461
ENSSSCG00000002908
ENSSSCG00000007717
ENSSSCG00000016441
ENSSSCG00000036257
ENSSSCG00000034802
ENSSSCG00000026082
ENSSSCG00000028282
ENSSSCG00000023912
ENSSSCG00000034655
ENSSSCG00000037170
ENSSSCG00000038130
ENSSSCG00000006543
ENSSSCG00000013046
ENSSSCG00000032963
ENSSSCG00000040379
ENSSSCG00000034913
ENSSSCG00000015453
ENSSSCG00000010315
ENSSSCG00000032857
ENSSSCG00000000635
ENSSSCG00000038348
ENSSSCG00000011061
ENSSSCG00000034131
ENSSSCG00000003201
ENSSSCG00000036956
ENSSSCG00000033735
ENSSSCG00000024245
ENSSSCG00000015897
ENSSSCG00000010454
ENSSSCG00000033982
ENSSSCG00000031800
ENSSSCG00000042788
ENSSSCG00000036824
ENSSSCG00000027045
ENSSSCG00000011876
ENSSSCG00000008729
ENSSSCG00000037431
ENSSSCG00000049234

ENSSSCG00000039854
ENSSSCG00000017758
ENSSSCG00000012375
ENSSSCG00000036224
ENSSSCG00000040651
ENSSSCG00000002907
ENSSSCG00000026617
ENSSSCG00000015016
ENSSSCG00000004666
ENSSSCG00000032390
ENSSSCG00000011356
ENSSSCG00000021494
ENSSSCG00000000197
ENSSSCG00000032436
ENSSSCG00000028978
ENSSSCG00000011391
ENSSSCG00000006771
ENSSSCG00000010477
ENSSSCG00000037771
ENSSSCG00000047578
ENSSSCG00000003861
ENSSSCG00000034723
ENSSSCG00000016653
ENSSSCG00000030371
ENSSSCG00000042062
ENSSSCG00000007423
ENSSSCG00000025027
ENSSSCG00000002697
ENSSSCG00000001232
ENSSSCG00000039662
ENSSSCG00000025329
ENSSSCG00000023280
ENSSSCG00000039896
ENSSSCG00000028889
ENSSSCG00000013747
ENSSSCG00000037120
ENSSSCG00000012791
ENSSSCG00000024562
ENSSSCG00000005678
ENSSSCG00000023178
ENSSSCG00000032469
ENSSSCG00000004057
ENSSSCG00000040183
ENSSSCG00000013575
ENSSSCG00000023305
ENSSSCG00000026455
ENSSSCG00000007278
ENSSSCG00000017705
ENSSSCG00000034994
ENSSSCG00000032343
ENSSSCG00000035048
ENSSSCG00000021597
ENSSSCG00000045223
ENSSSCG00000023557
ENSSSCG00000033700
ENSSSCG00000038079
ENSSSCG00000040162
ENSSSCG00000036658

ENSSSCG00000005344
ENSSSCG00000013118
ENSSSCG00000008377
ENSSSCG00000030300
ENSSSCG00000033113
ENSSSCG00000037132
ENSSSCG00000039216
ENSSSCG00000010495
ENSSSCG00000026067
ENSSSCG00000021834
ENSSSCG00000010514
ENSSSCG00000008088
ENSSSCG00000012171
ENSSSCG00000010568
ENSSSCG00000007366
ENSSSCG00000036157
ENSSSCG00000001817
ENSSSCG00000017700
ENSSSCG00000030888
ENSSSCG00000016210
ENSSSCG00000000849
ENSSSCG00000006552
ENSSSCG00000012007
ENSSSCG00000017103
ENSSSCG00000030582
ENSSSCG00000008677
ENSSSCG00000013226
ENSSSCG00000032557
ENSSSCG00000030388
ENSSSCG00000015784
ENSSSCG00000030575
ENSSSCG00000011561
ENSSSCG00000017261
ENSSSCG00000049577
ENSSSCG00000033381
ENSSSCG00000009746
ENSSSCG00000032860
ENSSSCG00000002459
ENSSSCG00000014835
ENSSSCG00000033051
ENSSSCG00000000950
ENSSSCG00000046783
ENSSSCG00000027119
ENSSSCG00000005134
ENSSSCG00000039867
ENSSSCG00000035297
ENSSSCG00000010508
ENSSSCG00000039148
ENSSSCG00000030510
ENSSSCG00000003332
ENSSSCG00000038950
ENSSSCG00000021712
ENSSSCG00000027689
ENSSSCG00000028227
ENSSSCG00000037782
ENSSSCG00000012277
ENSSSCG00000029757
ENSSSCG00000009653

ENSSSCG00000013853
ENSSSCG00000031669
ENSSSCG00000022404
ENSSSCG00000008303
ENSSSCG00000015110
ENSSSCG00000007552
ENSSSCG00000039553
ENSSSCG00000037238
ENSSSCG00000007554
ENSSSCG00000023298
ENSSSCG00000000186
ENSSSCG00000021319
ENSSSCG00000017913
ENSSSCG00000029668
ENSSSCG00000023531
ENSSSCG00000017104
ENSSSCG00000007076
ENSSSCG00000027426
ENSSSCG00000017114
ENSSSCG00000026931
ENSSSCG00000013366
ENSSSCG00000036096
ENSSSCG00000015600
ENSSSCG00000034898
ENSSSCG00000011195
ENSSSCG00000016122
ENSSSCG00000020856
ENSSSCG00000016216
ENSSSCG00000013303
ENSSSCG00000006480
ENSSSCG00000011620
ENSSSCG00000026587
ENSSSCG00000003852
ENSSSCG00000005908
ENSSSCG00000012950
ENSSSCG00000006273
ENSSSCG00000007228
ENSSSCG00000045632
ENSSSCG00000005440
ENSSSCG00000003973
ENSSSCG00000011133
ENSSSCG00000017544
ENSSSCG00000029425
ENSSSCG00000022149
ENSSSCG00000034147
ENSSSCG00000026446
ENSSSCG00000015140
ENSSSCG00000025965
ENSSSCG00000017420
ENSSSCG00000003914
ENSSSCG00000004826
ENSSSCG00000034984
ENSSSCG00000036614
ENSSSCG00000027307
ENSSSCG00000012352
ENSSSCG00000022797
ENSSSCG00000040575
ENSSSCG00000024693

ENSSSCG00000032340
ENSSSCG00000011560
ENSSSCG00000039758
ENSSSCG00000024990
ENSSSCG00000024313
ENSSSCG00000045512
ENSSSCG00000002855
ENSSSCG00000037066
ENSSSCG00000000406
ENSSSCG00000001516
ENSSSCG00000009592
ENSSSCG00000035928
ENSSSCG00000022258
ENSSSCG00000028202
ENSSSCG00000032985
ENSSSCG00000024316
ENSSSCG00000000158
ENSSSCG00000006166
ENSSSCG00000008978
ENSSSCG00000034207
ENSSSCG00000036669
ENSSSCG00000051054
ENSSSCG00000004047
ENSSSCG00000017835
ENSSSCG00000032234
ENSSSCG00000026339
ENSSSCG00000000394
ENSSSCG00000000739
ENSSSCG00000038912
ENSSSCG00000003288
ENSSSCG00000048507
ENSSSCG00000016215
ENSSSCG00000013304
ENSSSCG00000024344
ENSSSCG00000015730
ENSSSCG00000011318
ENSSSCG00000033808
ENSSSCG00000011065
ENSSSCG00000006830
ENSSSCG00000023379
ENSSSCG00000032315
ENSSSCG00000002307
ENSSSCG00000003089
ENSSSCG00000043043
ENSSSCG00000010161
ENSSSCG00000028331
ENSSSCG00000039830
ENSSSCG00000000685
ENSSSCG00000050998
ENSSSCG00000010240
ENSSSCG00000027847
ENSSSCG00000024018
ENSSSCG00000040875
ENSSSCG00000049441
ENSSSCG00000000396
ENSSSCG00000017755
ENSSSCG00000016902
ENSSSCG00000023837

ENSSSCG00000034215
ENSSSCG00000027257
ENSSSCG00000011313
ENSSSCG00000039770
ENSSSCG00000032062
ENSSSCG00000017549
ENSSSCG00000042527
ENSSSCG00000002847
ENSSSCG00000013082
ENSSSCG00000021241
ENSSSCG00000026414
ENSSSCG00000015231
ENSSSCG00000029264
ENSSSCG00000001978
ENSSSCG00000002135
ENSSSCG00000042444
ENSSSCG00000003134
ENSSSCG00000024233
ENSSSCG00000032067
ENSSSCG00000020915
ENSSSCG00000029005
ENSSSCG00000000743
ENSSSCG00000002050
ENSSSCG00000001561
ENSSSCG00000003105
ENSSSCG00000005628
ENSSSCG00000033913
ENSSSCG00000002849
ENSSSCG00000032261
ENSSSCG00000003582
ENSSSCG00000049851
ENSSSCG00000010084
ENSSSCG00000014565
ENSSSCG00000031847
ENSSSCG00000026752
ENSSSCG00000015056
ENSSSCG00000040986
ENSSSCG00000006835
ENSSSCG00000013936
ENSSSCG00000001573
ENSSSCG00000040663
ENSSSCG00000017255
ENSSSCG00000012857
ENSSSCG00000040815
ENSSSCG00000015604
ENSSSCG00000038401
ENSSSCG00000024655
ENSSSCG00000010795
ENSSSCG00000006238
ENSSSCG00000048696
ENSSSCG00000004657
ENSSSCG00000046958
ENSSSCG00000026977
ENSSSCG00000017192
ENSSSCG00000034137
ENSSSCG00000016449
ENSSSCG00000033731
ENSSSCG00000039731

ENSSSCG00000022089
ENSSSCG00000035379
ENSSSCG00000026043
ENSSSCG00000008284
ENSSSCG00000031595
ENSSSCG00000001679
ENSSSCG00000005105
ENSSSCG00000051050
ENSSSCG00000012943
ENSSSCG00000036213
ENSSSCG00000008553
ENSSSCG00000035995
ENSSSCG00000015340
ENSSSCG00000008648
ENSSSCG00000003348
ENSSSCG00000001701
ENSSSCG00000038719
ENSSSCG00000011601
ENSSSCG00000040089
ENSSSCG00000035618
ENSSSCG00000002952
ENSSSCG00000000002
ENSSSCG00000008304
ENSSSCG00000010608
ENSSSCG00000022151
ENSSSCG00000021971
ENSSSCG00000037767
ENSSSCG00000038351
ENSSSCG00000041665
ENSSSCG00000026302
ENSSSCG00000012845
ENSSSCG00000007508
ENSSSCG00000022322
ENSSSCG00000038717
ENSSSCG00000040317
ENSSSCG00000039419
ENSSSCG00000000024
ENSSSCG00000033451
ENSSSCG00000033089
ENSSSCG00000024189
ENSSSCG00000039158
ENSSSCG00000003566
ENSSSCG00000001392
ENSSSCG00000042512
ENSSSCG00000004048
ENSSSCG00000022237
ENSSSCG00000031037
ENSSSCG00000008282
ENSSSCG00000027660
ENSSSCG00000020970
ENSSSCG00000005911
ENSSSCG00000036883
ENSSSCG00000004912
ENSSSCG00000043809
ENSSSCG00000008693
ENSSSCG00000032819
ENSSSCG00000027855
ENSSSCG00000014338

ENSSSCG00000027130
ENSSSCG00000013003
ENSSSCG00000002009
ENSSSCG00000035344
ENSSSCG00000015106
ENSSSCG00000000975
ENSSSCG00000007715
ENSSSCG00000010589
ENSSSCG00000008647
ENSSSCG00000002990
ENSSSCG00000001576
ENSSSCG00000009373
ENSSSCG00000016724
ENSSSCG00000020808
ENSSSCG00000003152
ENSSSCG00000032060
ENSSSCG00000007493
ENSSSCG00000008633
ENSSSCG00000000399
ENSSSCG00000006527
ENSSSCG00000037913
ENSSSCG00000002709
ENSSSCG00000016218
ENSSSCG00000010271
ENSSSCG00000037513
ENSSSCG00000031730
ENSSSCG00000022786
ENSSSCG00000031741
ENSSSCG00000004369
ENSSSCG00000035914
ENSSSCG00000011562
ENSSSCG00000006029
ENSSSCG00000033453
ENSSSCG00000001565
ENSSSCG00000001589
ENSSSCG00000022301
ENSSSCG00000013735
ENSSSCG00000027538
ENSSSCG00000032777
ENSSSCG00000006966
ENSSSCG00000009881
ENSSSCG00000024311
ENSSSCG00000022405
ENSSSCG00000039494
ENSSSCG00000037343
ENSSSCG00000023604
ENSSSCG00000050493
ENSSSCG00000007803
ENSSSCG00000032578
ENSSSCG00000036136
ENSSSCG00000015652
ENSSSCG00000007133
ENSSSCG00000039673
ENSSSCG00000011797
ENSSSCG00000040060
ENSSSCG00000004548
ENSSSCG00000035443
ENSSSCG00000003968

ENSSSCG00000030681
ENSSSCG00000006717
ENSSSCG00000008703
ENSSSCG00000009178
ENSSSCG00000006477
ENSSSCG00000034216
ENSSSCG00000005661
ENSSSCG00000037077
ENSSSCG00000001773
ENSSSCG00000041954
ENSSSCG00000035565
ENSSSCG00000009182
ENSSSCG00000000623
ENSSSCG00000045225
ENSSSCG00000000080
ENSSSCG00000045485
ENSSSCG00000009084
ENSSSCG00000009137
ENSSSCG00000015786
ENSSSCG00000040355
ENSSSCG00000038112
ENSSSCG00000045776
ENSSSCG00000002036
ENSSSCG00000030165
ENSSSCG00000016275
ENSSSCG00000005619
ENSSSCG00000025423
ENSSSCG00000017396
ENSSSCG00000015227
ENSSSCG00000012076
ENSSSCG00000007748
ENSSSCG00000034487
ENSSSCG00000042208
ENSSSCG00000016078
ENSSSCG00000023318
ENSSSCG00000004670
ENSSSCG00000013048
ENSSSCG00000037358
ENSSSCG00000008311
ENSSSCG00000009720
ENSSSCG00000015108
ENSSSCG00000032914
ENSSSCG00000036178
ENSSSCG00000000774
ENSSSCG00000021161
ENSSSCG00000022925
ENSSSCG00000012890
ENSSSCG00000011621
ENSSSCG00000034738
ENSSSCG00000033311
ENSSSCG00000030408
ENSSSCG00000026602
ENSSSCG00000043070
ENSSSCG00000005895
ENSSSCG00000027121
ENSSSCG00000006499
ENSSSCG00000017614
ENSSSCG00000003505

ENSSSCG00000026784
ENSSSCG00000001137
ENSSSCG00000029239
ENSSSCG00000035991
ENSSSCG00000003109
ENSSSCG00000003756
ENSSSCG00000017364
ENSSSCG00000021620
ENSSSCG00000016714
ENSSSCG00000026229
ENSSSCG00000003551
ENSSSCG00000015566
ENSSSCG00000040236
ENSSSCG00000042841
ENSSSCG00000014031
ENSSSCG00000007007
ENSSSCG00000021383
ENSSSCG00000017392
ENSSSCG00000015215
ENSSSCG00000011389
ENSSSCG00000011624
ENSSSCG00000006380
ENSSSCG00000010029
ENSSSCG00000015973
ENSSSCG00000007596
ENSSSCG00000017416
ENSSSCG00000025593
ENSSSCG00000009945
ENSSSCG00000017754
ENSSSCG00000033114
ENSSSCG00000015504
ENSSSCG00000003931
ENSSSCG00000000688
ENSSSCG00000008035
ENSSSCG00000024867
ENSSSCG00000004342
ENSSSCG00000031374
ENSSSCG00000005355
ENSSSCG00000028879
ENSSSCG00000000116
ENSSSCG00000002316
ENSSSCG00000032768
ENSSSCG00000027621
ENSSSCG00000017717
ENSSSCG00000008077
ENSSSCG00000015850
ENSSSCG00000002723
ENSSSCG00000011612
ENSSSCG00000024596
ENSSSCG00000030567
ENSSSCG00000000184
ENSSSCG00000028696
ENSSSCG00000020906
ENSSSCG00000002406
ENSSSCG00000032115
ENSSSCG00000004180
ENSSSCG00000009904
ENSSSCG00000025729

ENSSSCG00000039672
ENSSSCG00000040931
ENSSSCG00000001510
ENSSSCG00000048111
ENSSSCG00000000838
ENSSSCG00000008496
ENSSSCG00000002020
ENSSSCG00000034961
ENSSSCG00000002719
ENSSSCG00000013745
ENSSSCG00000009921
ENSSSCG00000017507
ENSSSCG00000024692
ENSSSCG00000039252
ENSSSCG00000011806
ENSSSCG00000032165
ENSSSCG00000011000
ENSSSCG00000016677
ENSSSCG00000011106
ENSSSCG00000000981
ENSSSCG00000002431
ENSSSCG00000034570
ENSSSCG00000000045
ENSSSCG00000012077
ENSSSCG00000022312
ENSSSCG00000038521
ENSSSCG00000001346
ENSSSCG00000006219
ENSSSCG00000032710
ENSSSCG00000039713
ENSSSCG00000040396
ENSSSCG00000005528
ENSSSCG00000038543
ENSSSCG00000016226
ENSSSCG00000000265
ENSSSCG00000039300
ENSSSCG00000000082
ENSSSCG00000008878
ENSSSCG00000013436
ENSSSCG00000032833
ENSSSCG00000011965
ENSSSCG00000012893
ENSSSCG00000027840
ENSSSCG00000035193
ENSSSCG00000031485
ENSSSCG00000036383
ENSSSCG00000016732
ENSSSCG00000041810
ENSSSCG00000000171
ENSSSCG00000008094
ENSSSCG00000040709
ENSSSCG00000014898
ENSSSCG00000007355
ENSSSCG00000034927
ENSSSCG00000032474
ENSSSCG00000002535
ENSSSCG00000037775
ENSSSCG00000036746

ENSSSCG00000032591
ENSSSCG00000036887
ENSSSCG00000003006
ENSSSCG00000032367
ENSSSCG00000011578
ENSSSCG00000016513
ENSSSCG00000012161
ENSSSCG00000033183
ENSSSCG00000039635
ENSSSCG00000011893
ENSSSCG00000040134
ENSSSCG00000029059
ENSSSCG00000010821
ENSSSCG00000031918
ENSSSCG00000006359
ENSSSCG00000012967
ENSSSCG00000006560
ENSSSCG00000037168
ENSSSCG00000004542
ENSSSCG00000015337
ENSSSCG00000023423
ENSSSCG00000032221
ENSSSCG00000011676
ENSSSCG00000035434
ENSSSCG00000002767
ENSSSCG00000017723
ENSSSCG00000025486
ENSSSCG00000005287
ENSSSCG00000007119
ENSSSCG00000012853
ENSSSCG00000040486
ENSSSCG00000017230
ENSSSCG00000034696
ENSSSCG00000009888
ENSSSCG00000006590
ENSSSCG00000006487
ENSSSCG00000023173
ENSSSCG00000006360
ENSSSCG00000024166
ENSSSCG00000013524
ENSSSCG00000002516
ENSSSCG00000030415
ENSSSCG00000046117
ENSSSCG00000011447
ENSSSCG00000025981
ENSSSCG00000017517

Supplementary Table 6. The common downregulated differentially expressed genes from spleen and inguinal lymph node samples between the infected and mock group

GeneID
ENSSSCG00000050979
ENSSSCG00000003123
ENSSSCG00000051266
ENSSSCG00000004401
ENSSSCG00000027124
ENSSSCG00000015862
ENSSSCG00000010559
ENSSSCG00000004931
ENSSSCG00000007181
ENSSSCG00000038844
ENSSSCG00000006160
ENSSSCG00000017062
ENSSSCG00000034440
ENSSSCG00000035983
ENSSSCG00000050742
ENSSSCG00000004520
ENSSSCG00000049048
ENSSSCG00000042379
ENSSSCG00000043867
ENSSSCG00000032403
ENSSSCG00000042607
ENSSSCG00000008147
ENSSSCG00000009144
ENSSSCG00000048047
ENSSSCG00000020988
ENSSSCG00000043201
ENSSSCG00000039261
ENSSSCG00000015766
ENSSSCG00000032660
ENSSSCG00000027157
ENSSSCG00000032852
ENSSSCG00000035445
ENSSSCG00000000867
ENSSSCG00000009672
ENSSSCG00000008835
ENSSSCG00000022724
ENSSSCG00000015866
ENSSSCG00000035371
ENSSSCG00000014441
ENSSSCG00000035507
ENSSSCG00000020941
ENSSSCG00000032503
ENSSSCG00000002424
ENSSSCG00000005699
ENSSSCG00000042225
ENSSSCG00000039161
ENSSSCG00000006390
ENSSSCG00000012944
ENSSSCG00000031356
ENSSSCG00000016756
ENSSSCG00000038801
ENSSSCG00000014034
ENSSSCG00000015879
ENSSSCG00000000433
ENSSSCG00000016444
ENSSSCG00000006456
ENSSSCG00000006452

ENSSSCG00000013351
ENSSSCG00000001458
ENSSSCG00000013865
ENSSSCG00000026098
ENSSSCG00000011186
ENSSSCG00000041957
ENSSSCG00000015368
ENSSSCG00000033268
ENSSSCG00000036190
ENSSSCG00000036134
ENSSSCG00000011892
ENSSSCG00000001582
ENSSSCG00000045152
ENSSSCG00000038296
ENSSSCG00000024403
ENSSSCG00000006213
ENSSSCG00000010665
ENSSSCG00000008593
ENSSSCG00000050719
ENSSSCG00000017003
ENSSSCG00000028338
ENSSSCG00000023806
ENSSSCG00000009245
ENSSSCG00000010359
ENSSSCG00000002341
ENSSSCG00000004628
ENSSSCG00000004250
ENSSSCG00000026517
ENSSSCG00000005203
ENSSSCG00000003147
ENSSSCG00000044254
ENSSSCG00000015211
ENSSSCG00000048970
ENSSSCG00000021255
ENSSSCG00000006810
ENSSSCG00000005832
ENSSSCG00000004402
ENSSSCG00000023746
ENSSSCG00000036822
ENSSSCG00000022202
ENSSSCG00000012362
ENSSSCG00000032151
ENSSSCG00000032433
ENSSSCG00000012238
ENSSSCG00000050888
ENSSSCG00000025711
ENSSSCG00000009833
ENSSSCG00000016676
ENSSSCG00000016618
ENSSSCG00000005762
ENSSSCG00000035147
ENSSSCG00000020858
ENSSSCG00000009221
ENSSSCG00000014933
ENSSSCG00000046275
ENSSSCG00000007849
ENSSSCG00000010745
ENSSSCG00000024000

ENSSSCG00000038452
ENSSSCG00000024800
ENSSSCG00000038374
ENSSSCG00000007170
ENSSSCG00000051710
ENSSSCG00000010256
ENSSSCG00000050958
ENSSSCG00000011023
ENSSSCG00000047343
ENSSSCG00000031716
ENSSSCG00000003715
ENSSSCG00000033059
ENSSSCG00000046730
ENSSSCG00000012153
ENSSSCG00000006862
ENSSSCG00000004275
ENSSSCG00000011522
ENSSSCG00000028144
ENSSSCG00000008888
ENSSSCG00000003604
ENSSSCG00000046097
ENSSSCG00000013295
ENSSSCG00000035218
ENSSSCG00000009486
ENSSSCG00000012911
ENSSSCG00000048314
ENSSSCG00000017282
ENSSSCG00000033760
ENSSSCG00000027331
ENSSSCG00000034879
ENSSSCG00000001500
ENSSSCG00000011056
ENSSSCG00000021374
ENSSSCG00000006357
ENSSSCG00000004586
ENSSSCG00000011928
ENSSSCG00000034827
ENSSSCG00000011538
ENSSSCG00000051601
ENSSSCG00000045950
ENSSSCG00000011060
ENSSSCG00000020758
ENSSSCG00000028148
ENSSSCG00000034630
ENSSSCG00000008606
ENSSSCG00000016290
ENSSSCG00000043581
ENSSSCG00000015426
ENSSSCG00000022390
ENSSSCG00000012637
ENSSSCG00000039813
ENSSSCG00000003821
ENSSSCG00000045622
ENSSSCG00000016652
ENSSSCG00000015445
ENSSSCG00000038811
ENSSSCG00000023273
ENSSSCG00000049170

ENSSSCG00000037766
ENSSSCG00000034727
ENSSSCG00000042021
ENSSSCG00000013010
ENSSSCG00000033394
ENSSSCG00000036446
ENSSSCG00000004661
ENSSSCG00000033093
ENSSSCG00000033043
ENSSSCG00000009666
ENSSSCG00000028341
ENSSSCG00000034178
ENSSSCG00000010169
ENSSSCG00000026699
ENSSSCG00000005178
ENSSSCG00000037360
ENSSSCG00000047491
ENSSSCG00000005249
ENSSSCG00000038557
ENSSSCG00000031713
ENSSSCG00000008169
ENSSSCG00000001403
ENSSSCG00000010340
ENSSSCG00000039874
ENSSSCG00000001716
ENSSSCG00000010447
ENSSSCG00000014766
ENSSSCG00000016557
ENSSSCG00000034987
ENSSSCG00000013584
ENSSSCG00000049586
ENSSSCG00000009627
ENSSSCG00000048556
ENSSSCG00000026180
ENSSSCG00000037015
ENSSSCG00000032923
ENSSSCG00000012001
ENSSSCG00000037987
ENSSSCG00000016925
ENSSSCG00000015037
ENSSSCG00000015522
ENSSSCG00000027745
ENSSSCG00000005122
ENSSSCG00000012595
ENSSSCG00000041180
ENSSSCG00000001068
ENSSSCG00000013788
ENSSSCG00000001242
ENSSSCG00000031336
ENSSSCG00000022429
ENSSSCG00000013618
ENSSSCG00000015662
ENSSSCG00000040607
ENSSSCG00000000146
ENSSSCG00000007185
ENSSSCG00000023992
ENSSSCG00000011014
ENSSSCG00000012150

ENSSSCG00000036475
ENSSSCG00000005235
ENSSSCG00000051156
ENSSSCG00000007239
ENSSSCG00000029395
ENSSSCG00000012278
ENSSSCG00000028529
ENSSSCG00000016130
ENSSSCG00000010222
ENSSSCG00000033175
ENSSSCG00000045069
ENSSSCG00000028101
ENSSSCG00000016033
ENSSSCG00000011811
ENSSSCG00000016117
ENSSSCG00000011579
ENSSSCG00000012638
ENSSSCG00000035595
ENSSSCG00000031531
ENSSSCG00000003722
ENSSSCG00000021309
ENSSSCG00000034607
ENSSSCG00000050073
ENSSSCG00000020657
ENSSSCG00000047456
ENSSSCG00000013335
ENSSSCG00000037970
ENSSSCG00000025238
ENSSSCG00000049042
ENSSSCG00000011521
ENSSSCG00000013018
ENSSSCG00000030565
ENSSSCG00000024552
ENSSSCG00000010926
ENSSSCG00000003760
ENSSSCG00000044437
ENSSSCG00000046798
ENSSSCG00000015383
ENSSSCG00000035189
ENSSSCG00000017367
ENSSSCG00000029860
ENSSSCG00000022318
ENSSSCG00000006781
ENSSSCG00000031465
ENSSSCG00000010071
ENSSSCG00000050540
ENSSSCG00000007949
ENSSSCG00000007572
ENSSSCG00000037539
ENSSSCG00000001698
ENSSSCG00000016442
ENSSSCG00000031337
ENSSSCG00000016574
ENSSSCG00000040366
ENSSSCG00000011477
ENSSSCG00000012551
ENSSSCG00000048896
ENSSSCG00000000435

ENSSSCG00000004672
ENSSSCG00000024022
ENSSSCG00000040838
ENSSSCG00000015878
ENSSSCG00000038993
ENSSSCG00000007644
ENSSSCG00000002026
ENSSSCG00000029331
ENSSSCG00000050772
ENSSSCG00000013393
ENSSSCG00000016705
ENSSSCG00000034308
ENSSSCG00000036933
ENSSSCG00000013978
ENSSSCG00000036846
ENSSSCG00000042146
ENSSSCG00000033001
ENSSSCG00000003146
ENSSSCG00000042541
ENSSSCG00000026547
ENSSSCG00000025271
ENSSSCG00000023848
ENSSSCG00000017036
ENSSSCG00000009222
ENSSSCG00000041715
ENSSSCG00000050870
ENSSSCG00000009966
ENSSSCG00000009429
ENSSSCG00000016034
ENSSSCG00000003148
ENSSSCG00000012112
ENSSSCG00000002932
ENSSSCG00000023924
ENSSSCG00000008185
ENSSSCG00000037334
ENSSSCG00000044610
ENSSSCG00000021105
ENSSSCG00000024396
ENSSSCG00000011915
ENSSSCG00000036452
ENSSSCG00000014725
ENSSSCG00000047595
ENSSSCG00000030511
ENSSSCG00000016665
ENSSSCG00000022701
ENSSSCG00000014224
ENSSSCG00000000680
ENSSSCG00000002817
ENSSSCG00000034191
ENSSSCG00000012818
ENSSSCG00000039368
ENSSSCG00000016599
ENSSSCG00000049185
ENSSSCG00000031831
ENSSSCG00000002863
ENSSSCG00000014851
ENSSSCG00000046685
ENSSSCG00000031149

ENSSSCG00000014097
ENSSSCG00000046947
ENSSSCG00000025243
ENSSSCG00000001572
ENSSSCG00000037432
ENSSSCG00000050566
ENSSSCG00000049232
ENSSSCG00000032019
ENSSSCG00000026583
ENSSSCG00000046429
ENSSSCG00000032702
ENSSSCG00000015357
ENSSSCG00000016554
ENSSSCG00000011878
ENSSSCG00000000516
ENSSSCG00000008096
ENSSSCG00000005487
ENSSSCG00000038706
ENSSSCG00000013286
ENSSSCG00000037997
ENSSSCG00000037821
ENSSSCG00000038693
ENSSSCG00000003351
ENSSSCG00000017873
ENSSSCG00000041144
ENSSSCG00000009011
ENSSSCG00000036549
ENSSSCG00000023710
ENSSSCG00000014943
ENSSSCG00000035617
ENSSSCG00000002292
ENSSSCG00000029281
ENSSSCG00000037682
ENSSSCG00000014316
ENSSSCG00000006183
ENSSSCG00000005997
ENSSSCG00000032423
ENSSSCG00000000665
ENSSSCG00000005828
ENSSSCG00000000857
ENSSSCG00000003524
ENSSSCG00000000848
ENSSSCG00000033937
ENSSSCG00000003088
ENSSSCG00000051365
ENSSSCG00000033624
ENSSSCG00000029815
ENSSSCG00000032058
ENSSSCG00000050618
ENSSSCG00000039007
ENSSSCG00000040822
ENSSSCG00000015716
ENSSSCG00000015083
ENSSSCG00000040850
ENSSSCG00000004347
ENSSSCG00000041370
ENSSSCG00000007542
ENSSSCG00000031858

ENSSSCG00000040728
ENSSSCG00000005190
ENSSSCG00000016083
ENSSSCG00000039215
ENSSSCG00000015756
ENSSSCG00000041490
ENSSSCG00000012512
ENSSSCG00000004678
ENSSSCG00000046945
ENSSSCG00000011766
ENSSSCG00000015545
ENSSSCG00000013145
ENSSSCG00000015353
ENSSSCG00000014672
ENSSSCG00000017257
ENSSSCG00000004478
ENSSSCG00000023788
ENSSSCG00000024351
ENSSSCG00000015780
ENSSSCG00000004744
ENSSSCG00000048120
ENSSSCG00000021651
ENSSSCG00000029606
ENSSSCG00000015548
ENSSSCG00000008488
ENSSSCG00000038598
ENSSSCG00000000531
ENSSSCG00000004898
ENSSSCG00000003881
ENSSSCG00000005194
ENSSSCG00000046546
ENSSSCG00000035554
ENSSSCG00000025005
ENSSSCG00000001901
ENSSSCG00000030655
ENSSSCG00000007100
ENSSSCG00000021793
ENSSSCG00000003950
ENSSSCG00000008799
ENSSSCG00000013658
ENSSSCG00000022569
ENSSSCG00000039961
ENSSSCG00000034776
ENSSSCG00000030821
ENSSSCG00000012548
ENSSSCG00000006344
ENSSSCG00000004973
ENSSSCG00000046607
ENSSSCG00000006143
ENSSSCG00000009230
ENSSSCG00000007727
ENSSSCG00000025294
ENSSSCG00000015969
ENSSSCG00000035477
ENSSSCG00000010212
ENSSSCG00000021601
ENSSSCG00000039272
ENSSSCG00000046005

ENSSSCG00000022506
ENSSSCG00000016843
ENSSSCG00000010046
ENSSSCG00000003986
ENSSSCG00000003731
ENSSSCG00000010426
ENSSSCG00000049494
ENSSSCG00000013934
ENSSSCG00000035849
ENSSSCG00000016831
ENSSSCG00000039271
ENSSSCG00000015901
ENSSSCG00000006958
ENSSSCG00000002718
ENSSSCG00000040795
ENSSSCG00000004143
ENSSSCG00000036626
ENSSSCG00000000216
ENSSSCG00000012789
ENSSSCG00000017818
ENSSSCG00000000916
ENSSSCG00000049462
ENSSSCG00000044695
ENSSSCG00000002507
ENSSSCG00000027466
ENSSSCG00000021612
ENSSSCG00000040053
ENSSSCG00000026473
ENSSSCG00000010142
ENSSSCG00000040088
ENSSSCG00000031640
ENSSSCG00000036589
ENSSSCG00000009765
ENSSSCG00000017990
ENSSSCG00000004962
ENSSSCG00000026130
ENSSSCG00000027198
ENSSSCG00000037832
ENSSSCG00000026387
ENSSSCG00000023627
ENSSSCG00000015584
ENSSSCG00000004199
ENSSSCG00000032971
ENSSSCG00000013273
ENSSSCG00000032162
ENSSSCG00000004008
ENSSSCG00000023498
ENSSSCG00000027053
ENSSSCG00000016129
ENSSSCG00000044206
ENSSSCG00000023434
ENSSSCG00000006788
ENSSSCG00000017204
ENSSSCG00000042618
ENSSSCG00000017511
ENSSSCG00000030303
ENSSSCG00000022676
ENSSSCG00000047770

ENSSSCG00000023001
ENSSSCG00000040746
ENSSSCG00000011216
ENSSSCG00000041248
ENSSSCG00000036052
ENSSSCG00000049587
ENSSSCG00000036013
ENSSSCG00000030206
ENSSSCG00000008051
ENSSSCG00000002520
ENSSSCG00000037697
ENSSSCG00000033512
ENSSSCG00000045440
ENSSSCG00000003366
ENSSSCG00000032341
ENSSSCG00000025176
ENSSSCG00000033542
ENSSSCG00000000455
ENSSSCG00000012667
ENSSSCG00000031694
ENSSSCG00000013358
ENSSSCG00000039193
ENSSSCG00000017063
ENSSSCG00000042706
ENSSSCG00000015045
ENSSSCG00000005224
ENSSSCG00000001478
ENSSSCG00000005352
ENSSSCG00000026991
ENSSSCG00000028488
ENSSSCG00000000789
ENSSSCG00000039245
ENSSSCG00000013073
ENSSSCG00000014029
ENSSSCG00000015144
ENSSSCG00000041324
ENSSSCG00000000029
ENSSSCG00000049475
ENSSSCG00000001567
ENSSSCG00000014047
ENSSSCG00000025993
ENSSSCG00000032659
ENSSSCG00000025644
ENSSSCG00000023225
ENSSSCG00000048240
ENSSSCG00000016164
ENSSSCG00000000660
ENSSSCG00000022309
ENSSSCG00000050177
ENSSSCG00000032522
ENSSSCG00000009326
ENSSSCG00000007926
ENSSSCG00000035887
ENSSSCG00000005512
ENSSSCG00000004570
ENSSSCG00000033185
ENSSSCG00000039883
ENSSSCG00000037430

ENSSSCG00000004207
ENSSSCG00000025687
ENSSSCG00000045353
ENSSSCG00000035152
ENSSSCG00000000854
ENSSSCG00000039633
ENSSSCG00000001849
ENSSSCG00000004052
ENSSSCG00000032984
ENSSSCG00000037852
ENSSSCG00000007823
ENSSSCG00000011400
ENSSSCG00000004441
ENSSSCG00000016093
ENSSSCG00000010640
ENSSSCG00000015802
ENSSSCG00000028741
ENSSSCG00000000418
ENSSSCG00000010746
ENSSSCG00000007678
ENSSSCG00000008771
ENSSSCG00000015294
ENSSSCG00000044010
ENSSSCG00000040147
ENSSSCG00000028996
ENSSSCG00000041163
ENSSSCG00000015937
ENSSSCG00000049223
ENSSSCG00000016992
ENSSSCG00000003709
ENSSSCG00000045050
ENSSSCG00000010862
ENSSSCG00000042267
ENSSSCG00000030065
ENSSSCG00000048909
ENSSSCG00000034993
ENSSSCG00000025208
ENSSSCG00000012572
ENSSSCG00000038508
ENSSSCG00000010925
ENSSSCG00000026478
ENSSSCG00000036499
ENSSSCG00000024293
ENSSSCG00000034660
ENSSSCG00000026516
ENSSSCG00000011208
ENSSSCG00000012571
ENSSSCG00000015556
ENSSSCG00000038180
ENSSSCG00000007094
ENSSSCG00000011443
ENSSSCG00000012838
ENSSSCG00000000062
ENSSSCG00000035043
ENSSSCG00000011717
ENSSSCG00000008721
ENSSSCG00000024191
ENSSSCG00000005360

ENSSSCG00000011397
ENSSSCG00000045352
ENSSSCG00000011681
ENSSSCG00000026564
ENSSSCG00000011527
ENSSSCG00000002314
ENSSSCG00000016437
ENSSSCG00000023014
ENSSSCG00000046678
ENSSSCG00000045763
ENSSSCG00000004795
ENSSSCG00000016784
ENSSSCG00000016892
ENSSSCG00000027941
ENSSSCG00000022989
ENSSSCG00000010494
ENSSSCG00000022445
ENSSSCG00000010593
ENSSSCG00000002788
ENSSSCG00000012002
ENSSSCG00000015882
ENSSSCG00000046599
ENSSSCG00000024818
ENSSSCG00000047414
ENSSSCG00000007909
ENSSSCG00000040275
ENSSSCG00000040109
ENSSSCG00000007986
ENSSSCG00000009489
ENSSSCG00000040377
ENSSSCG00000016639
ENSSSCG00000006082
ENSSSCG00000008991
ENSSSCG00000037257
ENSSSCG00000043322
ENSSSCG00000027093
ENSSSCG00000004209
ENSSSCG00000029227
ENSSSCG00000033232
ENSSSCG00000035400
ENSSSCG00000005609
ENSSSCG00000010647
ENSSSCG00000015619
ENSSSCG00000023028
ENSSSCG00000006415
ENSSSCG00000024088
ENSSSCG00000044472
ENSSSCG00000008646
ENSSSCG00000034335
ENSSSCG00000025924
ENSSSCG00000034633
ENSSSCG00000022105
ENSSSCG00000006458
ENSSSCG00000048660
ENSSSCG00000032896
ENSSSCG00000028549
ENSSSCG00000005502
ENSSSCG00000004714

ENSSSCG00000011355
ENSSSCG00000027505
ENSSSCG00000015747
ENSSSCG00000046901
ENSSSCG00000009060
ENSSSCG00000011885
ENSSSCG00000023229
ENSSSCG00000013236
ENSSSCG00000016131
ENSSSCG00000021075
ENSSSCG00000012759
ENSSSCG00000003768
ENSSSCG00000035025
ENSSSCG00000012680
ENSSSCG00000049770
ENSSSCG00000011782
ENSSSCG00000047463
ENSSSCG00000009379
ENSSSCG00000009322
ENSSSCG00000036123
ENSSSCG00000005284
ENSSSCG00000051680
ENSSSCG00000031031
ENSSSCG00000030172
ENSSSCG00000003708
ENSSSCG00000001499
ENSSSCG00000024960
ENSSSCG00000007155
ENSSSCG00000010210
ENSSSCG00000005593
ENSSSCG00000009361
ENSSSCG00000016567
ENSSSCG00000008535
ENSSSCG00000005106
ENSSSCG00000015770
ENSSSCG00000009152
ENSSSCG00000010209
ENSSSCG00000002276
ENSSSCG00000011495
ENSSSCG00000047852
ENSSSCG00000021222
ENSSSCG00000026592
ENSSSCG00000011663
ENSSSCG00000016285
ENSSSCG00000038162
ENSSSCG00000006171
ENSSSCG00000048861
ENSSSCG00000033260
ENSSSCG00000015795
ENSSSCG00000028327
ENSSSCG00000006947
ENSSSCG00000032612
ENSSSCG00000025686
ENSSSCG00000013720
ENSSSCG00000036223
ENSSSCG00000008294
ENSSSCG00000048537
ENSSSCG00000023862

ENSSSCG00000013248
ENSSSCG00000040416
ENSSSCG00000015810
ENSSSCG00000023451
ENSSSCG00000037905
ENSSSCG00000003702
